# Chemospecific Heterostructure and Heteromaterial Assembly of Metal–Organic Framework Nanoparticles

**DOI:** 10.1021/jacs.4c15261

**Published:** 2025-01-30

**Authors:** Ailsa K. Edward, Romy Ettlinger, Zuzanna Z. Janczuk, Guoxiong Hua, Russell E. Morris, Euan R. Kay

**Affiliations:** 1 EaStCHEM School of Chemistry, University of St Andrews, St Andrews KY16 9ST, U.K.; 2 TUM School of Natural Sciences, Technical University of Munich, Lichtenbergstr. 4, Garching b. München 85748, Germany

## Abstract

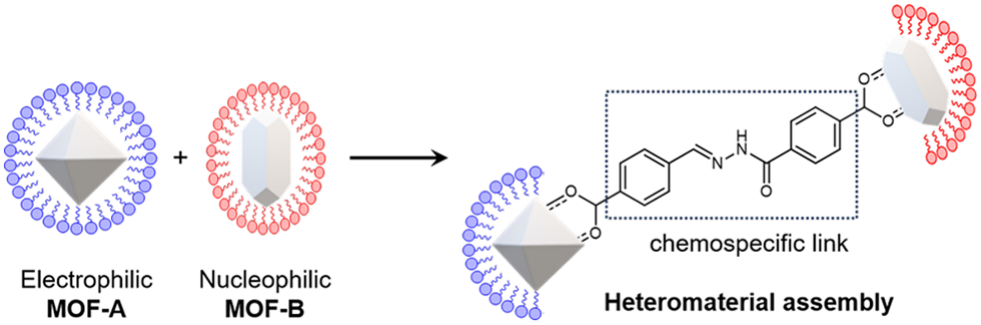

Nanoparticles of highly porous metal–organic frameworks (MOFs) are some of the most exciting nanomaterials under development, with potential applications that range from biomedicine and catalysis to adsorption technologies. However, our synthetic methodologies to functionalize and manipulate MOF nanoparticles (NPs) are less well developed than they might be. Here we create MOF NPs derivatized with hydrazone units on their exterior, enabling chemospecific reversible dynamic covalent modification of structures on the external surface. Pairwise combinations of nanometer-sized building blocks with complementary dynamic covalent surface units can be used to prepare heterostructure assemblies (i.e., two MOFs with different structures and morphologies) and heteromaterial assemblies (a MOF with a nanoparticle of another kind, in this case gold) in which the directional molecular-level dynamic covalent links demand intimate mixing of the two nanoscale components. Crucially, the defining characteristic of the MOF components—their porosity—is minimally affected by the external functionalization and interparticle linking. The development of atomically precise dynamic covalent functionalization on the external surface of MOF NPs opens up new avenues for programmable frameworks with responsive behaviors and modular assembly of porous materials with precise control over the spatial organization of multiple nanoscale building blocks.

## Introduction

Metal–organic frameworks (MOFs) are among the most intensively investigated material classes because of their unique combination of properties and extensive structural variability. Comprising metal nodes/oxoclusters connected by multitopic organic linkers, MOFs that exhibit exceptionally high porosity and accessible surface areas prepared using reticular design principles have led to many examples of their use in gas storage and separation.^1−4^ Additionally, MOFs are proposed for numerous other applications including catalysis,^5^ sensing,^6^ and biomedicine.^7−9^

MOF Nanoparticles (NPs)^10,11^ with crystallite sizes less than 100 nm exhibit enhanced or novel properties compared to their bulk counterparts^11^ such as unexpected memory effects on framework flexibility after crystal downsizing.^12^ Furthermore, solution processability makes MOF NPs practically useful for incorporating the attractive properties of porous frameworks into composite and hybrid materials.^13^ As MOF particle size is reduced, the importance of the external surface chemistry increases^14^ and there emerges an opportunity to modify or augment the intrinsic framework properties through surface chemical functionalization. However, reduction in particle size also raises significant challenges as MOF structures are often labile and relatively fragile,^15^ especially when compared to more established colloidal nanomaterials such as noble metal or semiconductor NPs. This means that manipulating and characterizing MOF NP surface chemistry with atomic precision is even more challenging–but at least as crucial–than it is for other types of NP.

Here we describe the surface functionalization of MOF NPs with functional units capable of dynamic covalent reactions.^16,17^ This facilitates the reversible manipulation of molecular structure on the external surface of MOF NPs and the controlled construction of local assemblies combining two different MOF structures (which we call heterostructure assemblies) or the preparation of heteromaterial assemblies that combine MOF NPs with NPs of another material entirely (e.g., gold). This approach demonstrates a general synthetic methodology for designing reactive nanoscale building blocks through which otherwise crystallographically incompatible nanomaterials can be intimately connected at the local level. Small abiotic covalent linking structures formed through chemospecific reactions define the connectivity between any two nanoscale building blocks, ensuring close contact between nanoparticles of different types and circumventing phase segregation–crucial factors for achieving synergistic effects in hybrid nanosystems.^18,19^ Importantly, the surface functionalization does not significantly affect access to the internal porosity of the MOF structures, which is critical in being able to exploit the unique properties of the MOF framework.

Surface modification of MOF NPs has recently seen rapid developments: examples include lipid,^20^ peptide,^21^ or oligonucleotide coatings,^22,23^ to enhance biocompatibility and cell uptake; phosphine ligands,^24^ which can support catalytic palladium NPs; and polymers for sensing applications.^25^ Conferred by the lability of metal–ligand interactions throughout the extended framework, the notion of reversibility is inherent in MOF chemistry–from their crystallization to their postsynthetic functionalization.^26−30^ Introducing reversible covalent functionality that is exclusively confined to the MOF NP exterior surface opens a new window through which the properties of framework materials can be modified, augmented and combined.

There are only a handful of examples that explore reversible chemistry on MOF NP surfaces independent of the framework coordination chemistry. In one,^31^ MOF NPs were functionalized with alkoxyamines, with the goal of nitroxide-mediated polymerization and covalent exchange reactions at the surface. Recently, reversible Diels–Alder reactions have been used to modify both the external and internal surfaces simultaneously.^29^ Crystalline arrays of polymer-passivated oligonucleotide-functionalized MOFs have also been prepared by exploiting reversible noncovalent DNA hybridization interactions.^32^

**Figure 1:**
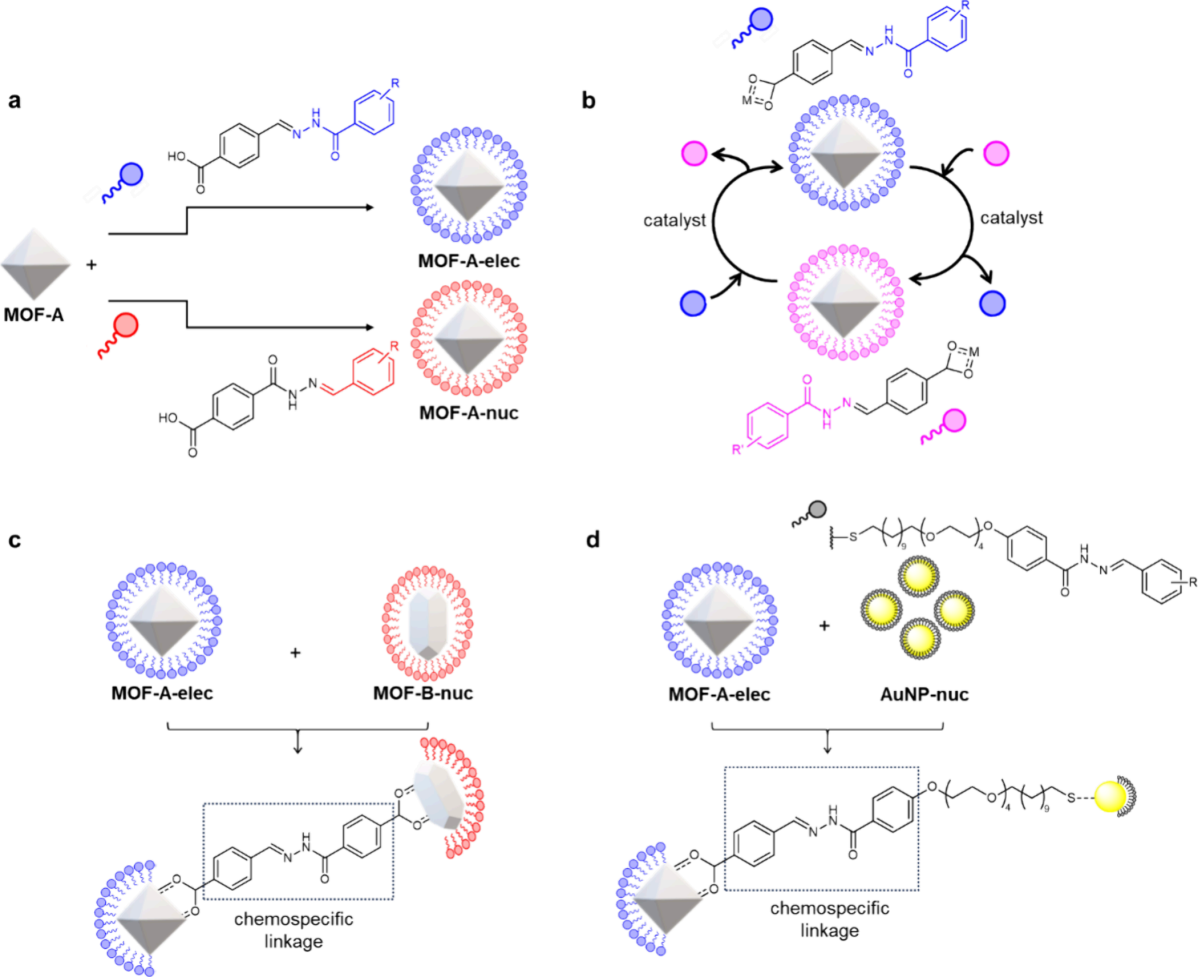
**Summary of synthetic strategy. a.** Postsynthetic modification of MOF NPs with either electrophilic (blue) or nucleophilic (red) hydrazone surface functionality, forming dynamic covalent MOF NPs with mutually complementary reactivity. **b.** Postsynthetic modification of covalent structure on the external surface of MOF NPs via dynamic covalent hydrazone exchange on application of specific catalytic signals. **c.** Assembly of heterostructure aggregates through chemospecific formation of dynamic covalent linkages between two MOF NP structures bearing mutually complementary surface functionality. **d.** Assembly of heteromaterial aggregates through chemospecific formation of dynamic covalent linkages between mutually complementary dynamic covalent MOF NPs and AuNPs.

On noble metal NPs, dynamic covalent reactions have been used to alter the molecular structure of surface-stabilizing ligands post synthesis,^33^ to switch and tune NP physicochemical properties,^34−36^ and to create programmable NP building blocks for size-, composition- and connectivity-selective covalently linked NP assemblies.^37−39^ Notwithstanding the significantly greater challenge presented by working with large, chemically labile organic-rich porous cores, we proposed that hydrazone-based dynamic covalent reactivity^40^ could be installed on the external surface of MOF NPs using simple, small-molecule modifiers. The ensuing surface-confined reversible chemical transformations will enable on-demand manipulation of MOF NP surface chemistry and chemospecific interparticle linking to create heterostructure and heteromaterial MOF hybrids (Figure 1).

## Results and Discussion

### Synthesis and Characterization of Functionalized MOF Nanoparticles

The general synthetic strategy is shown in Figure 1. Two mutually complementary ligand designs each combine a carboxylic acid for anchoring to vacant metal sites on the MOF NP surface with an *N*-acyl hydrazone for dynamic covalent exchange reactivity. Depending on the arrangement of the hydrazone bond, surface attachment produces either “electrophilic” (**elec**, Figure 1a, blue) or “nucleophilic” (**nuc**, Figure 1a, red) reaction-enabled MOF NPs that can undergo on-demand dynamic covalent exchange with complementary nucleophilic (Figure 1b) or electrophilic modifiers, respectively.

We chose to exemplify this strategy on two important MOF materials that have each been proposed for multiple applications and are known for their relative stability: UiO-66,^41^ and aluminum fumarate (Al-fum).^42^ UiO-66 NPs were prepared using a solvothermal method adapted from previously established protocols.^43^ Analysis by powder X-ray diffraction (PXRD), transmission electron microscopy (TEM), dynamic light scattering (DLS), and N_2_ adsorption (Figures S1–S4) confirmed the crystallinity and established size/morphology, colloidal stability, and porosity of the NPs, respectively. On comparison of the PXRD pattern with the literature,^41^ the structure of the UiO-66 was confirmed, and broadening of the reflections was observed, consistent with nanometre-sized crystalline domains. Electron microscopy revealed monodisperse, spherical nanometre-sized crystallites of <*d*_TEM_> = 17 (3) nm, which could be dispersed in ethanol to give particles with a solvodynamic diameter of <*d*_EtOH_> = 71 (2) nm. N_2_ adsorption (77 K) analysis revealed a Brunauer–Emmett–Teller (BET) apparent surface area of 1170 m^2^ g^–1^_,_ which matches the expected value for bulk UiO-66.^43^ This confirms that down-sizing of the MOF did not negatively affect the integrity of the framework.

**Figure 2:**
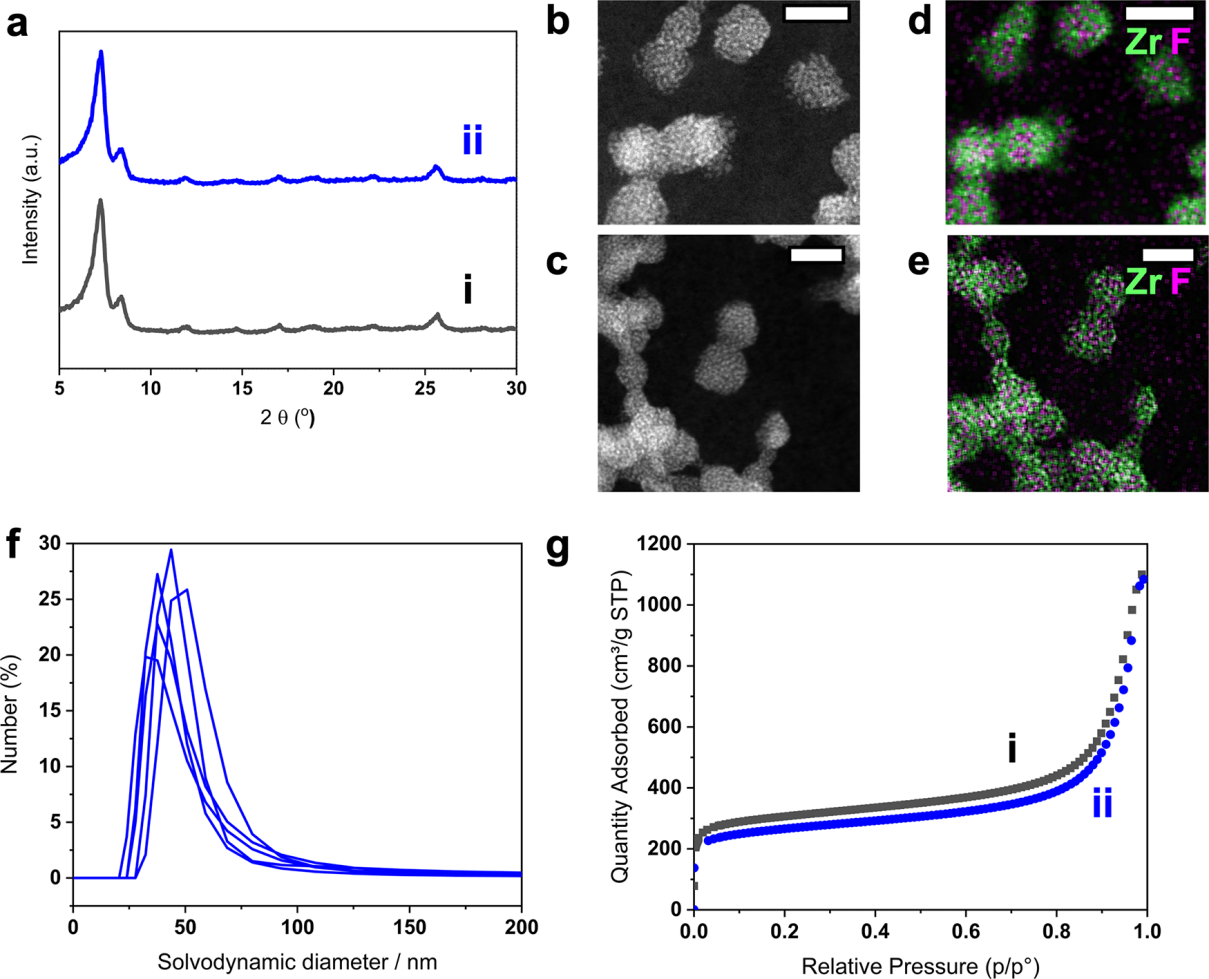
**Nanoscale characterization of UiO-66-elec-4F. a.** PXRD patterns of (**i**) unfunctionalized UiO-66 and (**ii**) **UiO-66-elec-4F** (blue). **b-c.** Representative HAADF STEM images (scale bars = 20 nm). **d-e**. Representative STEM-EDX maps (scale bars = 20 nm; green = Zr; pink = F). See Figure S6 for additional images. **f.** Solvodynamic size measured by DLS in ethanol, plotted as % number of particles (<*d*_EtOH_> = 50 (4) nm). **g.** N_2_ adsorption (77 K) isotherms of (**i**) unfunctionalized UiO-66 (black) and (**ii**) **UiO-66-elec-4F** (blue).

**Figure 3:**
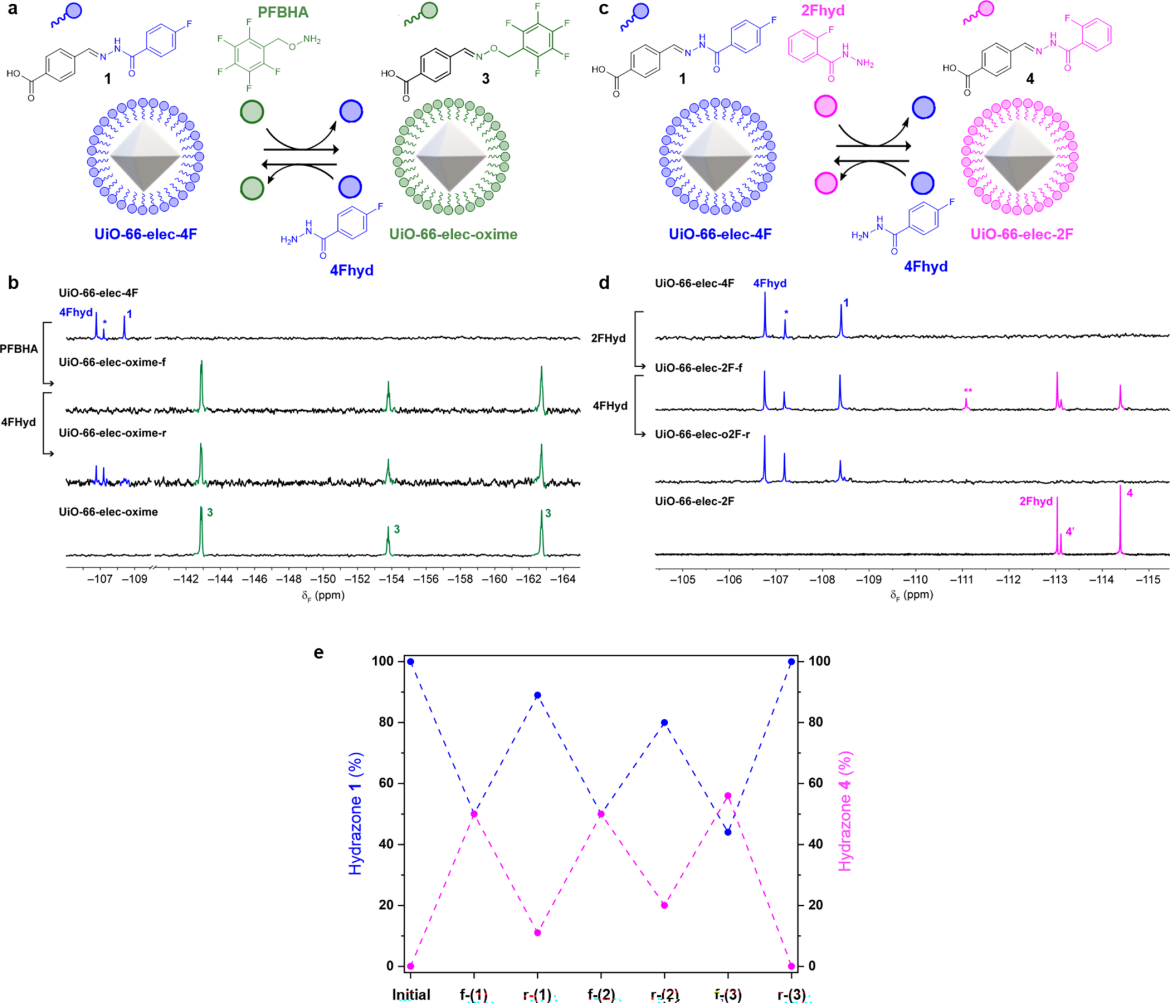
**Exchange of electrophilic UiO-66-elec-4F with nucleophilic modifiers. a**. Schematic representation of on-MOF exchange between **UiO-66-elec-4F** and **UiO-66-elec-oxime**. **b.**^19^F NMR spectra (376.7 MHz, DMSO-*d*_6_) after digestion with conc. H_2_SO_4_. Top to bottom: **UiO-66-elec-4F** (blue), **UiO-66-elec-oxime-f**, **UiO-66-elec-oxime-r**, authentic **UiO-66-elec-oxime** (green). See Tables S8 and S10 for quantitative analyses. **c.** Schematic representation of the on-MOF exchange between **UiO-66-elec-4F** and **UiO-66-elec-2F-f**. **d.**^19^F NMR spectra (376.7 MHz, DMSO-*d*_6_) after digestion with conc. H_2_SO_4_. Top to bottom: **UiO-66-elec-4F** (blue), **UiO-66-elec-2F-f**, **UiO-66-elec-2F-r**, authentic **UiO-66-elec-2F** (pink, signal doubling observed as a consequence of conformational isomers). See Tables S11 and S12 for quantitative analyses. **e.** Proportion of NP-bound hydrazones on repeated back-and-forth exchanges with **2Fhyd** then **4Fhyd**, starting from **UiO-66-elec-4F** as determined by acid digestion ^19^F NMR spectroscopy (Table S13). Blue trace: hydrazone **1**, pink trace: hydrazone **4**. * = 4-fluorobenzoic acid; ** = 2-fluorobenzoic acid. Note that exchange products are labeled as **UiO-66-elec-x-f/r** where **x** is replaced with a short abbreviation to describe the product formed in the exchange, and **f** or **r** indicates “forward” exchange from pristine material or “reverse” exchange.

**Figure 4:**
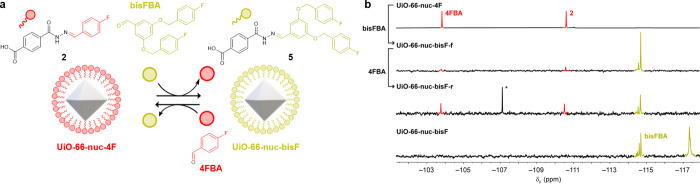
**Exchange of nucleophilic UiO-66-nuc-4F with electrophilic modifiers.** a) Schematic representation of on-MOF exchange between **UiO-66-nuc-4F** and **UiO-66-nuc-bisF**. **b.**^19^F NMR spectra (376.7 MHz, DMSO-*d*_6_) after digestion with conc. H_2_SO_4_. Top to bottom: **UiO-66-nuc-4F** (red), **UiO-66-nuc-bisF-f**, **UiO-66-nuc-bisF-r**, **UiO-66-nuc-bisF** (yellow). * = 4-fluorobenzoic acid.

UiO-66 was then modified with either hydrazone **1** (Figures 1a and 3, blue) or hydrazone **2** (Figures 1a and 4, red) via coordinative attachment of the terminal carboxylate groups to accessible Zr(IV)-oxoclusters present on the external surface of the UiO-66-NPs to give **UiO-66-elec-4F** and **UiO-66-nuc-4F**, respectively. The nanoscale characterization of **UiO-66-elec-4F** is described here (Figure 2) with equivalent data for **UiO-66-nuc-4F** reported in the Supporting Information (Section 10). PXRD measurements (Figure 2a) showed no difference between pristine UiO-66 and functionalized **UiO-66-elec-4F** indicating that the framework structure was unaffected by surface functionalization.^44^ Visualization by TEM confirmed that the size and shape of the particles were well preserved (Figure 2b,c). Energy dispersive X-ray (EDX) spectrometry mapping of **UiO-66-elec-4F** (Figure 2d,e) revealed colocalization of fluorine and zirconium, coincident with uniform functionalization of the particles with fluorine-labeled **1**. Detection of fluorine by X-ray photoelectron spectroscopy (XPS), which strictly probes the surface of the MOF particle, indicated a clear signal for fluorine 1s electrons which is not present in unfunctionalized MOF and so further confirmed that fluorine-labeled functionality was positioned at or near the particle surface (Figure S7). Carbon 1s and zirconium 3d XPS also showed some evidence of change compared to the unfunctionalized controls (Figure S7). The functionalized particles formed dispersions in ethanol. DLS measurements showed a slight reduction in solvodynamic diameter (<*d*_EtOH_> = 50 (4) nm, Figure 2f) compared to the unfunctionalized material, consistent with improved colloidal stability. Thermogravimetric analysis (TGA) showed a similar thermal stability for pristine UiO-66 and functionalized **UiO-66-elec-4F** (Figure S8).

Characterizing the molecular length-scale structures present on the nanoparticle exterior is critical to being able to rationally manipulate surface chemistry and interparticle connectivity. The molecular structure of surface-attached species was determined by liquid state ^19^F and ^1^H NMR spectroscopy after digestion of the functionalized MOF NPs in acid (Figures S9, S10, S20, S21), confirming that the benzoic acid anchored hydrazones **1** or **2** had been incorporated intact. Subjecting authentic samples of **1** or **2** to the acid digestion conditions confirmed that all observable species corresponded to the parent hydrazone or degradation products produced under the strongly acidic conditions (Figures S9, S10, S20, S21). Corroborating evidence of the structure and mode of attachment was provided by treating the MOF NPs with excess trifluoroacetic acid (TFA), which acted as a competitive carboxylic acid to displace metal-coordinated species while leaving the NPs intact. Liquid state ^19^F NMR analysis of the desorbed species again indicated attachment of hydrazone **1** (Figure S15) or **2** (Figure S25) by coordination of the benzoic acid functionality. Acid digestion analysis also proved that the ligand remains intact after activating the modified UiO-66 at 150 °C for 16 h (Figure S11).

Both digestion of the MOF (fully destroying the NPs) and stripping the metal-coordinated species from the surface using either TFA or citric acid as a competitive ligand^45^ (maintaining the particle structural integrity) was used in combination with quantitative ^19^F NMR analysis to determine the density of surface-coordinated hydrazones (Supporting Information Section 7). All methods gave consistent results. Similar average values of 0.34 ± 0.01 μmol hydrazone **1** per mg of **UiO-66-elec-4F** (Supporting Information Section 8) and 0.32 ± 0.02 μmol hydrazone **2** per mg of **UiO-66-nuc-4F** (Supporting Information Section 10.2) were determined for the electrophilic and nucleophilic surface functionalizations, respectively. For each construct, close agreement between the acid digestion and competitive binding methods implies that all hydrazones present are incorporated through specific coordination to Zr sites on the particle surface, with no significant nonspecific adsorption within the pores. Molecular-level quantification of surface functionalization density, in combination with the nanoscale particle size information, leads to an estimation of approximately 720 hydrazones attached to an average spherical **UiO-66-elec-4F** NP of 17 nm in diameter. Considering the available surface area and chemical makeup of the UiO-66 crystal structure, this corresponds to a surface density of 0.80 hydrazones nm^–2^ or 1.7 hydrazones per surface-accessible Zr_6_ oxocluster (Supporting Information Section 9). Given that for each Zr_6_ oxocluster only half the Zr atoms are exposed at the surface (the other three point into the bulk structure and therefore not accessible) the expected maximum functionalization is three hydrazones per cluster. This may be reduced by local kink or step defects at the surface of the MOF and by any steric effects between hydrazones or between hydrazone and the linker molecules also present. It is notable that the surface density of reactive ligands we observe here is significantly higher (by an order of magnitude) than that measured for oligonucleotide-functionalized MOF NPs,^23^ but close to an order of magnitude lower than typical functionalization densities on reaction-enabled noble metal nanoparticles,^39^ underlining the challenge associated with instituting and characterizing controlled surface modification on MOF NPs where coordination sites are relatively sparse.

Localization of the modification to only the external surface was evidenced by N_2_ adsorption measurements (Figure 2g). The measured BET surface area for **UiO-66-elec-4F** of 998 m^2^ g^–1^ corresponds to a value of 1109 m^2^ g^–1^ when normalized to account for the additional mass of **1** (Supporting Information Section 9), confirming that the porosity of the MOF was unaffected by the functionalization.

Further confirmation that the hydrazones are incorporated through specific coordination of the benzoic acid units to vacant Zr sites at the surface of the NPs was shown by treating pristine UiO-66 with control hydrazones lacking the carboxylate coordinating group. Analysis by ^19^F NMR spectroscopy after acid digestion indicated that the carboxylate-free control hydrazones were neither adsorbed within the pores nor attached to the surface (Figures S12, S22). This is important as it demonstrates that internal (bulk) “missing-linker” defects, which are common in UiO-66, are not accessible to the hydrazone modifiers and so play no part in the chemistry of the system.

### Dynamic Covalent Exchange On Electrophilic Reactive MOF NPs

Dynamic covalent exchange of electrophilic MOF-bound hydrazones with nucleophilic modifiers was explored using fluorine-labeled alkoxyamine **PFBHA** and hydrazide **2FHyd** (Figure 3a,c). From the range of possible catalytic systems for hydrazone exchange,^46,47^ effective conditions for each nucleophile were determined by bulk solution studies using small-molecule model hydrazones (Supporting Information Section 12). Stability tests of functionalized **UiO-66-elec-4F** in the absence of dynamic exchange modifier were used to determine reaction conditions under which the surface functionality remained intact and catalyst species were not incorporated in the porous framework (Supporting Information Section 11). Reference nanoparticles were also prepared by functionalizing **UiO-66** with oxime **3** or hydrazone **4**, the products of the putative on-particle dynamic covalent exchange processes (Supporting Information Section 13)

Exchange to give **UiO-66-elec-oxime-f** (Figure 3a) was achieved by redispersing **UiO-66-elec-4F** in 10% *v*/*v* D_2_O/DMF then treating with **PFBHA** (10 mol equiv) in the presence of aniline as catalyst. After 48 h at room temperature, the nanoparticles were separated from all unbound molecular species by cycles of washing then centrifugation (see Supporting Information Section 14 for full procedures). Analysis by ^19^F NMR spectroscopy following acid digestion revealed complete conversion of hydrazone **1** to oxime **3** (Figure 3b), confirmed by comparison to the spectrum generated from authentic **UiO-66-elec-oxime**.

The PXRD pattern of the recovered **UiO-66-elec-oxime-f** (Figure S45) revealed no major structural changes to the crystalline framework, confirming that the structural integrity of the MOF NPs was maintained. Quantitative assessment of functionalization density per particle (Table S8) indicated a reduction in reactive surface functionality after exchange. Desorption of a proportion of the reactive surface groups during the exchange process was confirmed by analysis of the reaction supernatant (Figure S44). Control experiments revealed that high excesses of the strongly nucleophilic **PFBHA** were responsible for triggering desorption of hydrazone **1**, or its exchange product **3**, from the NP surface (Supporting Information Section 14.2). Further controls also confirmed that no **PFBHA** or other species lacking a carboxylate anchor became bound to the nanoparticles or adsorbed in the MOF pores of the MOF during exchange (Supporting Information Section 14.3).

The reverse exchange reaction is disfavored on account of the thermodynamic stability of oximes relative to hydrazones.^48^ Nevertheless, treating **UiO-66-elec-oxime-f** with **4Fhyd** (50 mol equiv) produced **UiO-66-elec-oxime-r** on which 30% of surface-bound reactive units had been returned to hydrazone **1** (Figure 3b, Table S10).

Exchange between structures of similar thermodynamic stability would be expected to allow for more efficient back-and-forth cycling of NP-bound molecular structure. Exchange of **UiO-66-elec-4F** with **2Fhyd** (10 mol equiv) using a low concentration of TFA as catalyst (Figure 3c) produced a mixture of NP-bound hydrazones on **UiO-66-elec-2F-f** after 7 days at 35 °C (Figure 3d). Again, PXRD analysis of the purified product revealed that the porous structure was unaffected by the surface-bound covalent exchange (Figure S51). Quantitative analysis of **UiO-66-elec-2F-f** (Table S11) revealed that ca. 50% of the NP-bound hydrazones had been converted to **4**. Only a moderate loss of reactive units (ca. 24%) to solution was observed, consistent with the nucleophilicity of the exchange modifier being responsible for the higher losses on exchange with **PFBHA**. Pleasingly, treating **UiO-66-elec-2F-f** with 25 mol equiv of **4Fhyd** returned 100% of the NP-bound hydrazones to **1**, with no further loss of reactive units to solution (**UiO-66-elec-2F-r**, Figure 3d, Table S12), demonstrating the reversibility of the on-NP exchange reactions.

The repeatability and stability of dynamic covalent MOF NPs was investigated over multiple cycles of back-and-forth exchange (Figure 3e). Starting from **UiO-66-elec-4F**, treatment with **2Fhyd** (10 mol eq) to produce **UiO-66-elec-2F-f-(n)**, followed by **4Fhyd** (25 mol eq) to return **UiO-66-elec-2F-r-(n)** was repeated over three cycles (n = 1–3) corresponding to six individual exchange reactions. Quantitative analysis of the NP surface functionality after each reaction (Table S13) revealed that the forward cycles using 10 mol eq modifier achieved a roughly 50:50 mix of hydrazones **1** and **4** on the NP surface which could be returned to almost exclusively **1** on the reverse exchange using 25 mol eq modifier. After an initial net surface loss of ca. 30% on the first exchange reaction, all subsequent cycles proceeded with negligible surface losses. Notably, reduction in functionalization density to a plateau value of ∼0.23 μmol mg^–1^ corresponds to approximately 1 functional unit per surface oxocluster (Supporting Information Section 9). Furthermore, no change in framework crystallinity or particle morphology was observed after all three cycles (Figures S54–S55). This demonstrates the exciting potential of dynamic covalent chemistry to allow repeated modification of NP-bound covalent structure over multiple cycles without affecting the underlying porous framework.

### Dynamic Covalent Exchange on Nucleophilic Reactive MOF NPs

Reversing the direction of attachment of the hydrazone units gives dynamic covalent nanoparticles with surface functionality that should exchange with electrophilic molecular modifiers in a hydrolysis–recondensation process (Figure 1a, red). **UiO-66-nuc-4F** was treated with fluorine-labeled benzaldehyde **bisFBA** (10 mol equiv) in the presence of water and an acetic acid/aniline catalyst mixture (Figure 4a). After 7 d at 35 °C, the particles were recovered and purified from molecular species by washing–centrifugation cycles (see Supporting Information Section 17 for full procedures). ^19^F NMR spectroscopy after acid digestion and comparison to authentic **UiO-66-nuc-4F** and **UiO-66-nuc-bisF** (Figure 4b) revealed a mixture of hydrazones **2** and **5** on **UiO-66-nuc-bisF-f**. Quantitative analysis revealed 81% conversion to hydrazone **5** and a ca. 40% loss of functional species to desorption (Table S14). The reverse exchange was achieved on addition of 4-fluorobenzaldehyde (**4FBA**, 40 mol equiv) to **UiO-66-nuc-bisF-f** to give **UiO-66-nuc-bisF-r** with a 63:37 ratio of on-NP **2**:**5** and no further desorption of functional units (Figure 4b, Table S15).

### Heterostructure Assembly through Chemospecific Dynamic Molecular Linking

Combining dynamic covalent nanoparticles that possess mutually complementary reactive surface functionality presents an attractive strategy to assemble nanoscale building blocks directed by chemospecific molecular linkages.^38,39^ Hence, if MOF NPs of two different structures are functionalized with complementary dynamic covalent hydrazone surface units, then heterostructure aggregates that combine two (or more) porous frameworks can be created (Figure 1c). Crucially, the nature of the molecular-level linkages will be conferred on the heterostructure material. Chemospecific linking between “nucleophilic” and “electrophilic” surfaces demands intimate mixing of heterostructure NPs, avoiding phase segregation.

Nucleophilic hydrazone **2** was installed on ellipsoidal Al-fumarate MOF (Al-fum) NPs (<*w*_TEM_> = 9 (1) nm, <*l*_TEM_> = 28 (4) nm), using similar procedures as for UiO-66, to form **Al-fum-nuc-4F**. The functionalized MOF NPs were then characterized in the same manner as the UiO-66 NPs (Supporting Information Section 19) and surface species quantified through ^19^F NMR studies, revealing a surface loading of 0.1 μmol of **2** per milligram of **Al-fum-nuc-4F**.

**Figure 5:**
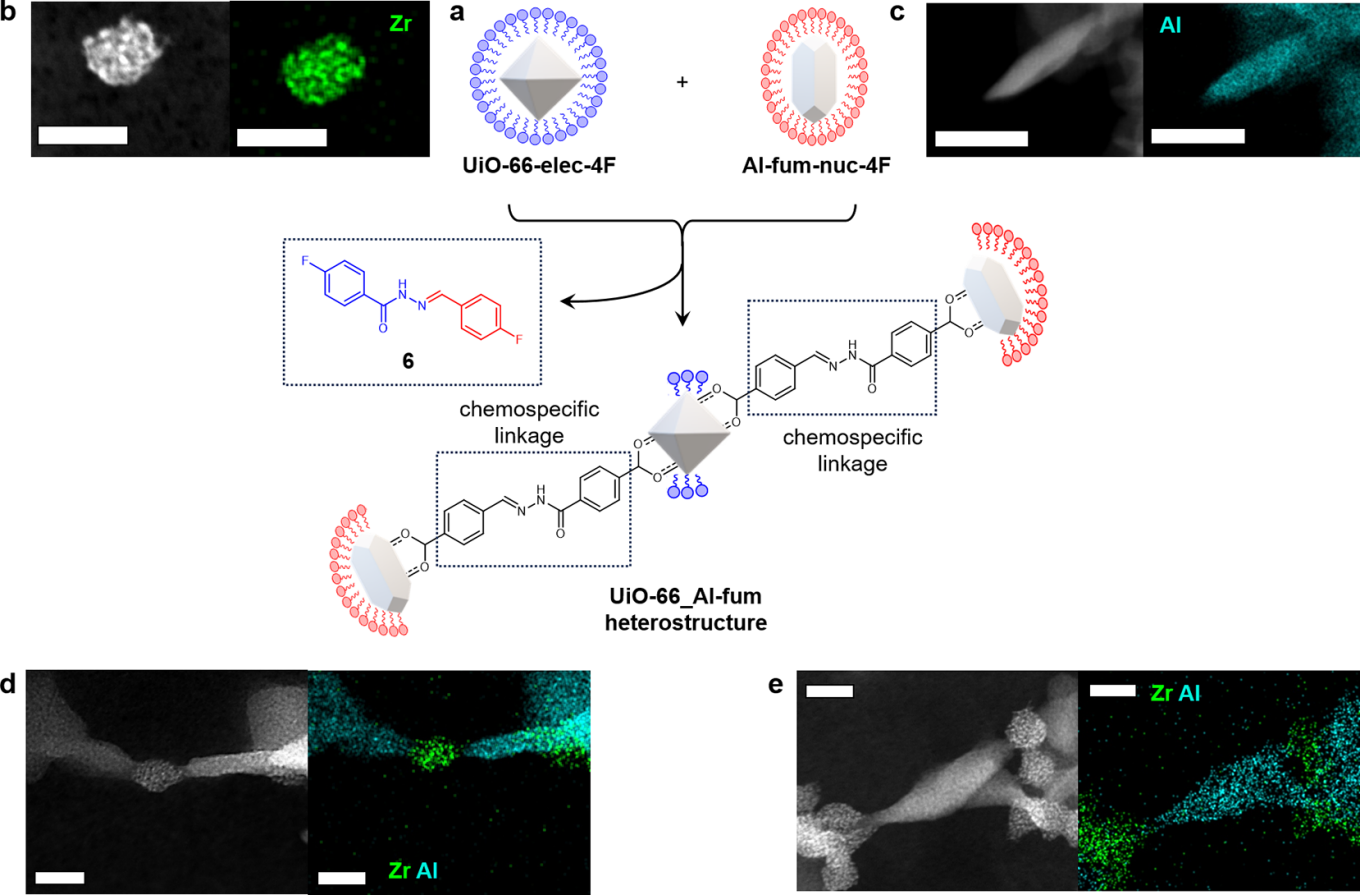
**UiO-66_Al-fum heterostructure assembly**. **a.** Schematic representation of heterostructure assembly directed by chemospecific hydrazone links between mutually complementary MOF NPs of differing framework structures. **b.** HAADF STEM-EDX images of **UiO-66-elec-4F**: scale bar = 20 nm; green = Zr. For additional images, see Figure S6. **c.** STEM-EDX images of Al-fum-nuc-4F; scale bar = 50 nm; cyan = Al. For additional images, see Figure S67**d**, **e** HAADF STEM-EDX images of heterostructure assemblies from two independent batches. Green = Zr; cyan = Al; scale bars: 20 nm. For additional images, see Figures S78–S79.

Heterostructure assemblies were formed by combining **UiO-66-elec-4F** and **Al-fum-nuc-4F** (Figure 5a). The two mutually complementary dynamic covalent MOF NPs were dispersed in solvent to give equimolar concentrations of hydrazone units **1** and **2**, then TFA added to initiate exchange. After 7 days under gentle heating, the assemblies were separated from the reaction supernatant by centrifugation and subsequent washing. On analysis of a dried aliquot of the purified assembly by TEM, the two NP types could be differentiated visually by their morphological differences (spherical UiO-66 vs ellipsoidal Al-fum). EDX analysis confirmed consistently selective heterostructure links between the two different material types (Figure 5 d,e). Interestingly Al-fum particles appeared to preferentially engage in links at the regions of highest curvature suggesting some degree of shape-dependent directional connectivity, which is consistent with the anisotropic crystal structure of the material. The crystal structure of Al-fum consists of one-dimensional chains of aluminum atoms linked parallel to the direction of the pores in the material. This anisotropy manifests itself as an elongated morphology with the metal chains exposed at the ends of the particles. This means that there is a natural point at which the hydrazone functionalization can take place, leading to the anisotropic linking to the UiO-66 particles that we observe in all the experiments. This is in contrast to the UiO-66 particles themselves where, given the high symmetry of the underlying crystal structure and the isotropic nature of the pseudo-spherical NPs, there is no expectation of any localization of the hydrazone functionalization in any one position. This is confirmed by the EDX studies that show fluorine distributed across the whole particle.

Consistent with the formation of the hydrazone dynamic covalent links, byproduct **6** was observed by ^19^F NMR spectroscopy on the reaction supernatant (Figure S80). Crucially, N_2_ adsorption (77 K) measurement (1062 m^2^g^–1^) revealed no significant loss of porosity in the heterostructured material compared to the **Al-fum-nuc-4F** (946 m^2^g^–1^) and **UiO-66-elec-4F** (998 m^2^g^–1^) constituents (Figure S81), indicating that this strategy is a route to hybrid porous materials that retain the structural and functional properties of their constituent parts. On comparing the PXRD pattern of the recovered material to the patterns for component materials (Figure S82), reflections corresponding to Al-fum predominate, alongside a broad peak consistent with the dominant (111) reflection in the PXRD pattern for UiO-66.^49^ This is a consequence of the fact that a higher mass of Al-fum was required to achieve an equimolar concentration of NP-bound hydrazones.

Control reactions confirmed that the hydrazone exchange mechanism is essential for formation of the heterostructure aggregates. On incubating **Al-fum-nuc-4F** with **UiO-66-elec-4F** in the absence of TFA catalyst, no aggregates were formed – TEM analysis of a dried aliquot of the mixture after 7 d showed only self-sorting of the two different particle types (Figure S83). Combining the pristine, unfunctionalized particles under the exchange conditions that included the acid catalyst also did not produce any linking between Al-fum and UiO-66 MOFs (Figure S84).

### Heteromaterial Assembly through Chemospecific Dynamic Molecular Linking

In previous work, we have exploited hydrazone dynamic covalent exchange for manipulating surface chemistry and connectivity of monolayer-stabilized noble metal nanoparticles.^33,34,38,39^ We therefore hypothesized that, despite the considerable structural differences, mutually complementary hydrazone MOF NPs and AuNPs should react to generate heteromaterial hybrid aggregates with intimate mixing of the two nanoscale building blocks.

**Figure 6:**
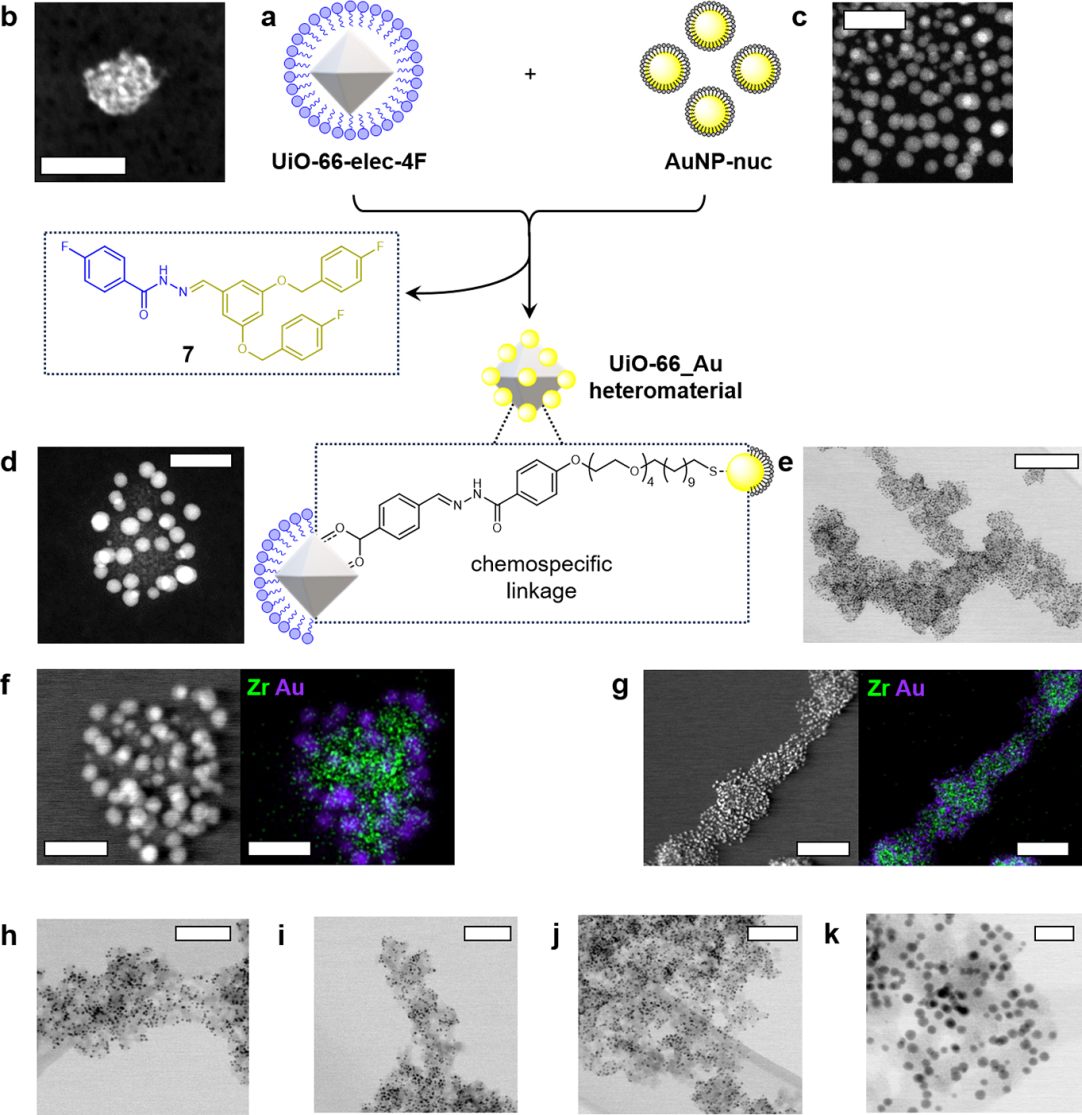
**UiO-66_Au heteromaterial assembly. a.** Schematic representation of heteromaterial assembly driven by chemospecific hydrazone links between mutually complementary reactive nanoparticle building blocks of differing core materials. **b.** STEM image of **UiO-66-elec-4F**: scale bar = 20 nm; **c.** TEM image of **AuNP-nuc**; scale bar = 20 nm. **d-g.** STEM-HAADF images and STEM-EDX images of **UiO-66**_**Au** heteromaterial assemblies produced at high stoichiometric excess of AuNP building blocks (ca. 14:1 AuNP:UiO-66-NP). Green = Zr, purple = Au. Scale bars: **d.** 20 nm, **e.** 200 nm, **f.** 20 nm, **g.** 100 nm. For additional images, see Figures S92–S93. **h-k.** STEM-HAADF images of **UiO-66_Au** heteromaterial assemblies produced at roughly equimolar ratio of building blocks (ca. 1.4:1 AuNP:UiO-66-NP). Scale bars: **h.** 100 nm, **i.** 100 nm, **j.** 100 nm, **k.** 20 nm. For additional images, see Figure S94.

For this approach, we combined electrophilic **UiO-66-elec-4F** with complementary **AuNP-nuc** (Figure 6, see Supporting Information sections 4 and 21 for preparation and characterization of **AuNP-nuc**). To create discrete assemblies, an excess of the smaller **AuNP-nuc** particles was employed; the exchange reaction was accelerated by TFA and gentle heating as before (see Supporting Information Section 22 for full procedures). After 7 days, analysis of an aliquot of the supernatant by ^19^F NMR spectroscopy revealed formation of the molecular byproduct, hydrazone **7**, as well as excess isolated **AuNP-nuc** (Figure S91). At this point, the nanoparticle material was separated from supernatant by centrifugation, then subsequent washing steps removed the excess of small AuNPs from the precipitate. TEM analysis of the solid residue revealed planet–satellite assemblies of the larger MOF NPs decorated with small AuNPs, confirming chemospecific linking of the two reactive nanoparticle building blocks (Figure 6d–g).

The adaptability of the dynamic covalent linking approach could be demonstrated by repeating the same protocol using a new batch of **UiO-66-elec-4F** at a close to equimolar ratio of building blocks. In this case, the MOF NPs were more sparsely decorated with AuNPs, resulting in extended aggregates when dried, in which AuNPs and MOF NPs each link between more than one partner (Figure 6h–k). The PXRD pattern of the heteromaterial aggregate (Figure S95) confirmed that the crystalline structure of UiO-66 remained intact, with all major reflections observable. Additional strong, broad reflections at 2θ values of 38° and 44° were also observed, corresponding to (111) and (200) reflections of crystalline gold originating from the AuNPs.^50^ N_2_ adsorption (77 K) measurements (Figure S96) revealed that the porosity of the MOF is maintained. The measured BET surface area of 553 m^2^ g^–1^ normalizes to ∼690 m^2^ g^–1^ in terms of MOF material after accounting for 19 wt % AuNPs as determined by TGA analysis (Figure S97). The modest reduction in surface area might originate from blocking of the pore entrances by the surface-attached AuNPs, suggesting intriguing potential for nanoscale gating of access to the MOF internal surface area.

In a control experiment, incubating chemically noncomplementary building blocks **AuNP-nuc** and **UiO-66-nuc-4F** under the dynamic covalent exchange conditions failed to produce well-defined assemblies (Figure S98), demonstrating the essential role of the chemospecific molecular linking chemistry for heteromaterial assembly.

Heteromaterial assembly of nanoscale building blocks directed by molecular length scale surface-confined processes is independent of the underlying nanomaterial constitution. Consequently, the same strategy could be used to create intimately mixed Al-fum_Au heteromaterials by combining mutually complementary **Al-fum-elec-4F** and **AuNP-nuc** building blocks (See Supporting Information Section 19 for full procedures). Imaging of the Al-fum_Au aggregates (Figures S99–S100) again revealed that the interfaces between components were preferentially heteromaterial in nature–the hallmark of chemospecific molecular length scale hydrazone links. Interestingly, AuNP components were mostly attached to Al-fum at regions of highest surface curvature, in line with our observations from heterostructure MOF hybrids (see above) that suggest preferential functionalization of the Al-fum surface at these regions, consistent with the anisotropic crystal structure.

## Conclusions

Presenting large external surface areas and amenable to solvent-based processing, colloidal nanomaterials can combine the features of extended structures with size-dependent nanoscale properties and molecular length scale interactions. We have shown that introducing dynamic covalent reactive functionality on the exterior of MOF NPs allows surface-bound covalent structure to be repeatedly modified under mild conditions without affecting the underlying porous architecture. The resulting reaction-enabled MOF NPs present a platform for augmenting and modulating physicochemical properties and for manipulating how framework materials interact with their surroundings postsynthesis using familiar solvent-based processing and programmable chemical transformations.

Using hydrazone dynamic covalent bonds with reaction kinetics that are switchable between locked and labile states according to the presence of simple molecular catalysts allowed us to prepare and characterize stable functionalized MOF NPs that react only on demand. This approach allowed us to surmount the challenges associated with comparatively low surface to volume ratios and sparse surface coordination sites to achieve higher surface density of functionalization (by an order of magnitude) than has previously been shown for MOF NPs, to characterize the surface-confined molecular structure with atomic precision, and to quantitatively assess on-surface covalent transformations. It was therefore possible to establish that either partial modification or exhaustive covalent exchange of surface-bound structures can be achieved depending on exchange unit reactivity and reaction conditions. This level of understanding is critical for informing rational synthetic planning when exploiting reaction-enabled MOF NPs as building blocks.

Chemospecific covalent reactions allow the synthetic principles of complementarity and orthogonality to be applied to the modification and linking of reaction-enabled MOF NPs. By combining MOF NP building blocks bearing chemically complementary reactive surface functional groups but crystallographically incompatible core frameworks, we have assembled unprecedented heterostructure MOF aggregates. Likewise, reaction-enabled MOF NPs can be integrated with complementary dynamic covalent NPs of other types to create heteromaterial assemblies. By defining the interparticle linkages, the directional hydrazone bond is critical to the programmable assembly of heterostructure and heteromaterial aggregates in which phase segregation of differing nanomaterials is not possible and aggregate composition is sensitive to the stoichiometry of reactive building blocks.

In contrast to other emerging methods for connecting structurally different frameworks, such as core–shell (MOF@MOF) NPs^51,52^ and heterointerpenetration,^53,54^ our strategy does not hinder access to the porous structure of either constituent. Complementary to approaches that apply oligonucleotide hybridization to assemble discrete and crystalline heteromaterial arrays,^23,32^ using dynamic covalent reactions between abiotic surface-bound molecules draws upon the rich diversity of synthetic chemistry, which can be compatible with a wide range of both aqueous and nonaqueous environmental conditions. Although the oligonucleotide-directed approach can achieve beautiful structural control, this approach requires a thick NP surface coating, adding significantly to the size of the particles. This puts limitations on the structures that can be created and means that the closest approach between MOF NPs is >50 nm. By contrast, our approach uses small molecules of <1 nm in length, allowing almost intimate contact between the NPs without fusing. The significant steric effects of large biomolecules means that the degree of functionalization on the exterior is of the MOF is an order of magnitude greater in our work than in any previous oligonucleotide-based approach. Importantly and despite this extra degree of functionalization, our approach does not limit access to the interior of the MOFs.

Short transport (diffusion) pathways and close contact between components will be critical to achieving synergistic interactions in heteromaterial structures so that the properties of two different nanoscale constituents can be harnessed for applications such as sequential or tandem catalytic processes^55^ and chemical logic gates.^56^ Independent of the underlying nanomaterial structure, this strategy should be generalizable to any number of framework materials or heteromaterial combinations with other solution-processable components. Together with an ever-expanding palette of dynamic covalent chemistries that operate under mild conditions and applied in chemospecific complementary and orthogonal combinations,^57−59^ our methods lay the foundations of a synthetic strategy for hierarchical assemblies on mesoscopic and macroscopic length scales composed of nanosized building blocks, directed by the precision of covalent interactions.^60^

## Data Availability

The research data supporting this publication can be accessed at https://doi.org/10.17630/682adaf9-c8a7-47e6-b1da-a7c4b994faef.

## Supporting Information Available

Full experimental details of synthetic procedures and characterization of nanoparticles, including all control reactions (PDF)
