# Supplementary material for: Chemospecific Heterostructure and Heteromaterial Assembly of Metal–Organic Framework Nanoparticles
Source: J Am Chem Soc. 2025 Jan 30;147(6):5114–24. doi: 10.1021/jacs.4c15261 (PMC11826876; doi:10.1021/jacs.4c15261)
Supplement: Supplementary file 1 — ja4c15261_si_001.pdf [file ja4c15261_si_001.pdf]

Supporting Information

Chemospecific Heterostructure and Heteromaterial Assembly of Metal–Organic Framework Nanoparticles

Ailsa K. Edward,[1] Romy Ettlinger,[1,2] Zuzanna Z. Janczuk,[1] Guoxiong Hua,[1] Russell E. Morris*[1] and Euan R. Kay*[1]

[1]*EaStCHEM School of Chemistry, University of St Andrews, St Andrews KY16 9ST, UK*
[2]*TUM School of Natural Sciences, Technical University of Munich, Lichtenbergstr. 4, Garching b. München 85748, Germany*

**Corresponding author emails: rem1@st-andrews.ac.uk, ek28@st-andrews.ac.uk.*

1. Materials and methods....4
2. Synthesis of organic compounds....6
3. Synthesis of MOFs....21
4. Synthesis of **AuNP-nuc**....23
5. Characterisation of unfunctionalized UiO-66....24
6. Characterization of **UiO-66-elec-4F**....27
7. Quantitative NMR procedures....31
   7.1 Acid digestion ^{1}H NMR spectroscopy....31
   7.2 Acid digestion ^{19}F NMR spectroscopy....31
   7.3 TFA stripping ^{19}F NMR spectroscopy....31
   7.4 Citric acid stripping ^{19}F NMR spectroscopy....32
8. Quantitative assessment of **UiO-66-elec-4F** surface functionalisation....33
   8.1 Acid digestion ^{1}H NMR spectroscopy....33
   8.2 Acid digestion ^{19}F NMR spectroscopy....33
   8.3 TFA stripping ^{19}F NMR spectroscopy....34
   8.4 Citric Acid stripping ^{19}F NMR spectroscopy....35
9. Estimation of surface functionalisation density....36
10. Characterisation of **UiO-66-nuc-4F**....38
   10.1 Structural characterisation of **UiO-66-nuc-4F**....38
   10.2 Quantitative assessment of **UiO-66-nuc-4F** surface functionalisation....40
11. Stability tests of **UiO-66-elec-4F** under dynamic covalent exchange conditions....43
   11.1 Stability test procedures....43
   11.2 Analysis of stability studies on **UiO-66-elec-4F**....45
12. Model exchange reactions on electrophilic molecular substrate (**S3**)....49

12.1 Exchange with *o*-(2,3,4,5,6-pentafluorobenzyl)hydroxylamine hydrochloride (**PFBHA**) 49
12.2 Exchange with 2-fluorobenzoic hydrazide (**2Fhyd**) 49
13. Synthesis and characterisation of reference electrophilic MOF NPs 51
13.1 Synthesis of UiO-66 functionalized with oxime **3** 51
13.2 Characterisation of UiO-66 functionalized with oxime **3** 51
13.3 Synthesis of UiO-66 functionalized with hydrazone **4** 52
13.4 Characterization of UiO-66 functionalized with hydrazone **4** 52
14. Exchange between **UiO-66-elec-4**F and **UiO-66-elec-oxime** 54
14.1 Modification of **UiO-66-elec-4F** by exchange with **PFBHA** 54
14.2 Test reactions to explain desorption of functional units 56
14.3 Control exchange reactions 58
14.4 Reverse exchange of **UiO-66-elec-oxime** with **4Fhyd** 59
15. Exchange between **UiO-66-elec-4F** and **UiO-66-elec-2F** 61
15.1 Modification of **UiO-66-elec-4F** by exchange with **2Fhyd** 61
15.2 Reverse exchange of **UiO-66-elec-2F-f** with **4Fhyd** 63
15.3 Back-and-forth exchange of **UiO-66-elec-4F** with **2Fhyd** and **4Fhyd** 64
16. Synthesis and characterisation of reference nucleophilic MOF NPs 67
16.1 Synthesis of UiO-66 functionalized with nucleophilic hydrazone **5** 67
16.2 Characterisation of UiO-66 functionalized with **5** 67
17. Exchange between **UiO-66-nuc-4F** and **UiO-66-nuc-bisF** 69
17.1 Modification of **UiO-66-nuc-4F** by exchange with **bisFBA** 69
17.2 Reverse exchange of **UiO-66-nuc-bisF-f** with **4FBA** 71
18. Characterisation of unfunctionalized Al-fum particles 72
19. Characterisation of functionalized Al-fum particles 74
19.1 Structural characterisation of **Al-fum-nuc-4F** 74
19.2 Quantitative analysis of **Al-fum-nuc-4F** 76
19.3 Structural characterisation of **Al-fum-elec-4F** 77
19.4 Quantitative analysis of **Al-fum-elec-4F** 78
20. Heterostructure assembly 80
20.1 Formation of heterostructure assemblies 80
20.2 Characterisation of heterostructure assemblies 81
20.3 Heterostructure assembly control experiments 84
21. Characterisation of **AuNP-nuc** 86
21.1 Nanoscale structural analysis 86
21.2 Molecular structural analysis 87
22. Heteromaterial assemblies 90
22.1 Assembly of UiO-66_Au heteromaterial 90
22.2 Characterisation UiO-66_Au heteromaterials 91
22.3 Heteromaterial assembly control – non-complementary building blocks 96

22.4 Al-fum_Au heteromaterial 96
23. ^{1}H, ^{19}F and ^{13}C NMR spectra and mass spectra of organic compounds 98
23.1 Spectral data for **1** 98
23.2 Spectral data for **2** 99
23.3 Spectral data for **S3** 101
23.4 Spectral data for **S4** 102
23.5 Spectral data for **S5** 104
23.6 Spectral data for **3** 106
23.7 Spectral data for **S6** 107
23.8 Spectral data for **4** 109
23.9 Spectral data for **S10** 111
23.10 Spectral data for **S11** 113
23.11 Spectral data for **5** 115
23.12 Spectral data for **7** 116
23.13 Spectral data for **S12** 118
23.14 Spectral data for **S14** 120
23.15 Spectral data for **S15** 122
23.16 Spectral data for **S16**$_2$ 124
24. References 126

1. Materials and methods

All reagents were purchased from commercial sources and used without further purification unless stated otherwise. Centrifugation was carried out on sample volumes 2–50 mL using a Heraeus Multifuge X1R fitted with Fiberlite F15-8X50C rotor operating at either 20 °C or at 5 °C to enhance sedimentation when necessary at the quoted speed (up to 14500 rpm); for sample volumes < 2 mL using a Sigma 1- 14 Microfuge with fixed angle polypropylene rotor at 14800 rpm; or for low-speed centrifugation, a Centurion Scientific C2 series was used with a fixed angle rotor operating at cycles of 6000 rpm for 15-20 minutes. Powder X-ray Diffraction was measured using a STOE STADI/P diffractometer using Mo $K_{\alpha 1}$ radiation (0.70930 Å) operated in capillary Debye-Scherrer mode. The 2θ values were translated to allow for comparison with those obtained using Cu $K_{\alpha 1}$ x-rays. This was calculated by the following equation.

$$2\theta_{CuK\alpha 1} = 2\,sin^{-1}\left(\frac{\lambda_{CuK\alpha 1}}{\lambda_{MoK\alpha 1}}\sin\left(\frac{2\theta_{MoK\alpha 1}}{2}\right)\right)$$

BET-specific surface area determination from N_2 isotherms was carried out according to the Rouquerol theory,[1] using the Microactive Software Kit v4.03.04. Data were recorded on a Micromeritics Tristar ii Surface Area and Porosity Instrument. Samples were added to a frit tube and activated *in vacuo* (150 °C, ~3×10^{-5} mbar, 16 h) prior to the measurement. TEM micrographs were obtained using a FEI Titan Themis operated at 200 kV on samples prepared by deposition of one drop of nanoparticle suspension on holey carbon films supported on a 300 mesh Cu grid (Agar Scientific®). Nanoparticle diameters were measured either automatically or manually using the software ImageJ. The images were first converted to black and white images using the "Threshold" function. The area of each nanoparticle was measured using the "Analyse particles" function. Particles on the edges were excluded, otherwise a scale bar was set and individual particle diameters were measured. Dynamic light scattering experiments were performed on a Malvern Zetasizer Pro instrument using a glass cuvette of 10 mm path length. The mean solvodynamic diameters reported are an average from 5 independent measurements where each measurement involves 15 sequential scans. ^{1}H, ^{13}C and ^{19}F NMR spectra were recorded on Bruker AVII 400 (operating at 400.1 MHz) and Bruker AVIII-HD 400 (operating at 400.3 MHz) fitted with a BBFO probe, Bruker AVIII-HD 500 (operating at 499.9 MHz) fitted with SmartProbe BBFO+ or a Bruker AVIII 500 (operating at 500.1 MHz) fitted with CryoProbe Prodigy BBO instruments, at a constant temperature of 25 °C. ^{1}H and ^{13}C NMR spectra were referenced to literature values of residual solvent peaks, and ^{19}F NMR chemical shifts were referenced to added 4-fluorotoluene (–118.68 ppm) internal standard. Spectra were analysed and *J* coupling constants were calculated using MestReNova (Version 14.0.1) software. Quantitative NMR spectra were run with a recycle delay time (d_1) equal to 5 x T_1 measured for the internal standard. All spectra were analysed

using MestReNova (Version 14.0.1). Mass spectrometry data were acquired through the University of St Andrews School of Chemistry mass spectrometry service using a Thermo Exactive Orbitrap mass spectrometer. Thermal gravimetric analysis (TGA) was performed on a Staton Redcroft STA-780. Dried nanoparticle samples (ca. 3–5 mg) were weighed into a blanked alumina crucible. The sample was then heated under a stream of air at a ramp rate of 10 °C/min from room temperature to 700 °C for the MOF NPs or at a ramp rate of 10 °C/min from room temperature to 900 °C for Au NPs. X-ray Photoelectron Spectroscopy (XPS) was measured using a Scienta 300 with SPECS monochromated AlKα source (photon energy 1486.6 eV) operating at or below a base pressure of 1 x 10^{-9} mbar at approximately 12 kV and 200 watts power. The instrument maintains a pass energy set to 150 eV for all spectra. Survey scans were collected at a dwell 225 time of 133 ms, step size 200 meV and 2 or 3 scans were added. Data was analysed and fitted using CasaXPS (version 2.1.0.1) and plotted using Origin software.

2. Synthesis of organic compounds

(*E*)-4-((2-(4-Fluorobenzoyl)hydrazineylidene)methyl)benzoic acid (1)

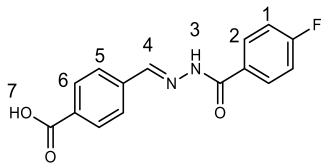

4-Formylbenzoic acid (0.429 g, 2.60 mmol) and 4-fluorobenzhydrazide (0.40 g, 2.86 mmol) were dissolved in 1:1 DCM/methanol (40 mL). Then, glacial acetic acid (6% v/v) was added and the mixture was stirred at room temperature for 7 hours. The solvent was then removed under reduced pressure to recover the crude product. The solid was then dissolved in the minimum volume of DMF and drops of methanol were added until solid precipitate was formed. The solution was left overnight and then the product was filtered and washed with ice-cold methanol to isolate a yellow powder (0.368 g, 1.29 mmol, 49%).

^{1}H NMR (500.1 MHz, DMSO-d$_6$): δ 12.04 (s, 1H, H-3), 8.50 (s, 1H, H-4), 8.06-7.95 (m, 4H, H-2,6), 7.90-7.80 (m, 2H, H-5), 7.39 (*apparent* t, 2H, J = 8.7 Hz, H-1) ppm.

^{13}C NMR (125.8 MHz, DMSO-d$_6$): δ 166.9 (COOH), 164.3 (d, $J_{C\text{-}F}$ = 249.1 Hz, Ar-F), 162.2 (C=O), 146.7 (C$_4$), 138.3 (C$_q$-Ar), 131.8 (C$_q$-Ar), 130.5 (d, $J_{C\text{-}F}$ = 9.0 Hz, C$_2$), 129.8 (C$_6$), 129.7 (d, $J_{C\text{-}F}$ = 2.8 Hz, Ar-F), 127.2 (C$_5$), 115.6 (d, $J_{C\text{-}F}$ = 21.9 Hz, C$_1$) ppm.

^{19}F NMR (376.5 MHz, DMSO-d$_6$): δ -108.11 (s, 1F) ppm.

HRMS (ES-) calculated *m/z* for $C_{15}H_{10}FN_2O_3$ $[M\text{-}H]^-$ 285.0681, found 285.0686.

(*E*)-4-(2-(4-Fluorobenzylidene)hydrazine-1-carbonyl)benzoic acid (2)

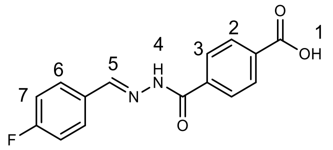

4-(Hydrazinocarbonyl) benzoic acid (0.360 g, 2.00 mmol) was dissolved in 1:1 DCM/methanol (80 mL), and 4-fluorobenzaldehyde (0.273 g, 2.20 mmol, 0.236 mL) was added. To this solution, acetic acid (6% v/v) was also added and then mixture left to stir at room temperature for 22 hours. The solvent was then removed under reduced pressure to recover the crude product. The crude was then recrystallized in toluene and washed with ice-cold toluene. This solid was then further purified via recrystallization with methanol and washed with ice-cold methanol to afford the product as a white powder (0.028 g, 0.097 mmol, 12 %).

^{1}H NMR (499.9 MHz, DMSO-d$_6$): δ 12.02 (s, 1H, H-4), 8.46 (s, 1H, H-5), 8.11-7.98 (m, 4H, H-2,3), 7.81 (dd, 2H, J = 8.6, 5.7 Hz, H-6), 7.32 (*apparent* t, 2H, J = 8.6, 5.7 Hz, H-7) ppm.

^{13}C NMR (125.8 MHz, DMSO-d$_6$): δ 166.8 (COOH), 163.2 (d, J_{C-F} = 251.3 Hz, Ar-F), 162.5 (C=O), 147.2 (C_5), 137.1 (C_q-Ar), 133.5 (C_q-Ar), 130.9 (d, J_{C-F} = 2.4 Hz, C_q-Ar), 129.4 (d, J_{C-F} = 8.4 Hz, C_6), 129.4 (C_2), 127.9 (C_3), 116.0 (d, J_{C-F} = 22.2 Hz, C_7) ppm.
^{19}F NMR (470.4 MHz, DMSO-d$_6$): δ -110.35 (s, 1F) ppm.
HRMS (ES-) calculated *m/z* for $C_{15}H_{10}FN_2O_3$ $[M-H]^-$ 285.0681, found 285.0678.

(*E*)-*N*'-benzylidene-4-fluorobenzohydrazide (S1)[2]

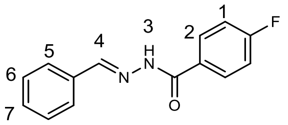

Benzaldehyde (0.304 g, 2.86 mmol, 0.29 mL) and 4-fluorobenzhydrazide (0.40 g, 2.60 mmol) were dissolved in 1:1 DCM/methanol (40 mL). Then, glacial acetic acid (6% v/v) was added and the mixture was stirred at room temperature for 24 hours. The solvent was then removed under reduced pressure to recover the crude product. The solid was then recrystallized from the minimum volume of aqueous ethanol to recover off-white crystals (0.346 g, 1.43 mmol, 55%). Spectral data in agreement with the literature.[2]
^{1}H NMR (500.1 MHz, DMSO-d$_6$): δ 11.89 (s, 1H, H-3), 8.46 (s, 1H, H-4), 8.04 – 7.97 (m, 2H, H-2), 7.74 (d, *J* = 6.8 Hz, 2H, H-5), 7.50 – 7.42 (m, 3H, H-6,7), 7.38 (*apparent* t, *J* = 8.7 Hz, 2H, H-1) ppm.
^{13}C NMR (125.8 MHz, DMSO-d$_6$): δ 164.2 (d, J_{C-F} = 249.3 Hz, Ar-F), 162.1 (C=O), 147.9 (C-4), 134.3 (C_q-Ar), 130.4 (d, J_{C-F} = 9.0 Hz, C-2), 130.2 (C-7), 129.9 (d, J_{C-F} = 2.5 Hz, C_q-Ar), 128.9 (C-6), 127.1 (C-5), 115.5 (d, J_{C-F} = 21.8 Hz, C-1) ppm.
^{19}F NMR (470.4 MHz, DMSO-d$_6$): δ -108.37 (s, 1F) ppm.
HRMS (ES+) calculated *m/z* for $C_{14}H_{11}FN_2NaO$ $[M+Na]^+$ 265.0748, found 265.0741.

(*E*)-*N*'-(4-Fluorobenzylidene)benzohydrazide (S2)[3]

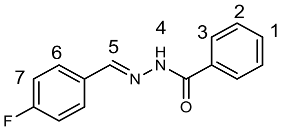

Benzhydrazide (0.327 g, 2.50 mmol) was dissolved in 1:1 DCM/methanol (50 mL) and 4-fluorobenzaldehyde (0.313 g, 2.52 mmol, 0.27 mL) was added followed by glacial acetic acid (6% v/v) and the mixture was stirred at room temperature for 24 hours. The solvent was then removed under reduced pressure to recover the crude product. The solid was then recrystallized from the minimum volume of aqueous ethanol and the solution filtered under vacuum and solid washed with ice-cold ethanol to recover a white powder (0.302 g,1.25 mmol, 52%). Spectral data in agreement with the literature.[3]

^{1}H NMR (499.9 MHz, DMSO-d$_6$): δ 11.86 (s, 1H, H-4), 8.47 (s, 1H, H-5), 7.92 (d, *J* =7.6 Hz, 2H, H-3), 7.83-7.76 (m, 2H, H-6), 7.62-7.57 (m, 1H, H-1), 7.56-7.48 (m, 2H, H-2), 7.30 (*apparent* t, *J* =8.5 Hz, 2H, H-7) ppm.
^{13}C NMR (125.8 MHz, DMSO-d$_6$): δ 163.2 (d, J_{C-F} = 249.6 Hz, Ar-F), 163.2 (C=O), 146.7 (C-5), 133.4 (C$_q$-Ar), 131.8 (C-1), 131.0 (d, J_{C-F} = 2.6 Hz, C$_q$-Ar), 129.3 (d, J_{C-F} = 8.6 Hz, C-6), 128.5 (C-2), 127.7 (C-3), 116.0 (d, J_{C-F} = 21.9 Hz, C-7)
^{19}F NMR (470.4 MHz, DMSO-d$_6$): δ -110.58 (s, 1F) ppm.
HRMS (ES+) calculated *m/z* for $C_{14}H_{11}FN_2NaO$ [M+Na]$^+$ 265.0748 found 265.0740.

Methyl (*E*)-4-((2-(4-Fluorobenzoyl)hydrazineylidene)methyl)benzoate (S3)

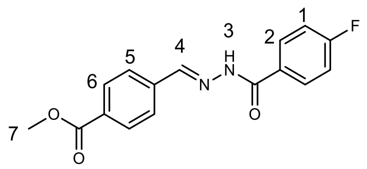

Methyl-4-formyl benzoate (0.469 g, 2.86 mmol) and 4-fluorobenzhydrazide (0.40 g, 2.60 mmol) were dissolved in 1:1 DCM/methanol (40 mL). Then, glacial acetic acid (6% v/v) was added and the mixture was stirred at room temperature for 24 hours. The solvent was then removed under reduced pressure to recover the crude product. The solid was then recrystallized from the minimum volume of aqueous methanol to recover an off-white powder (0.583 g, 1.94 mmol, 75%).
^{1}H NMR (500.1 MHz, DMSO-d$_6$): δ 12.06 (s, 1H, H-3), 8.50 (s, 1H, H-4), 8.08-7.95 (m, 4H, H-6,2), 7.93-7.83 (m, 2H, H-5), 7.39 (*apparent* t, 2H, *J* = 8.7 Hz, H-1), 3.87 (s, 3H, H-7) ppm.
^{13}C NMR (125.8 MHz, DMSO-d$_6$): δ 165.9 (COOMe), 164.3 (d, J_{C-F} = 249.3 Hz, Ar-F), 162.3 (C=O), 146.4 (C$_4$), 138.8 (C$_q$-Ar), 130.5 (d, J_{C-F} = 9.2 Hz, C$_2$), 129.7 (C$_6$), 127.3(C$_5$), 115.6 (d, J_{C-F} = 21.8 Hz, C$_1$), 52.3 (C$_7$) ppm
^{19}F NMR (470.4 MHz, DMSO-d$_6$): δ -108.07 (s, 1F) ppm.
HRMS (ES+) calculated *m/z* for $C_{16}H_{14}FN_2O_3$ [M+H]$^+$ 301.0983, found 301.0979.

Methyl (*E*)-4-((((Perfluorophenyl)methoxy)imino)methyl)benzoate (S4)

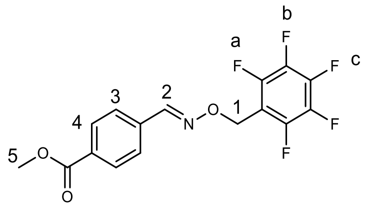

O-(2,3,4,5,6-Pentafluorobenzyl) hydroxylamine hydrochloride (0.10 g, 0.40 mmol) was dissolved in 1:1 DCM/methanol (10 mL) and methyl-4-formylbenzoate (0.072 g, 0.44 mmol) was added followed by glacial acetic acid (6% v/v) and the mixture was stirred at room temperature for 24 hours. The solvent was then removed under reduced pressure to recover

the crude product. The solid was then recrystallized from the minimum volume of aqueous ethanol to recover white crystals (0.108 g, 0.30 mmol, 75%).

^{1}H NMR (500.1 MHz, DMSO-d$_6$): δ 8.39 (s, 1H, H-2), 7.99 (d, 2H, *J* = 8.4 Hz, H-4), 7.71 (d, 2H, *J* = 8.4 Hz, H-3), 5.31 (s, 2H, H-1), 3.86 (s, 2H, H-5) ppm.

^{13}C NMR (125.8 MHz, DMSO-d$_6$): δ 165.7 (C=O), 149.7 (C$_2$), 145.2 (d, $J_{C\text{-}F}$ = 249.7 Hz, Ar-F), 140.9 (d, $J_{C\text{-}F}$ = 251.6 Hz, Ar-F), 137.0 (d, $J_{C\text{-}F}$ = 252.0 Hz, Ar-F), 135.8 (C$_q$-Ar), 130.8 (C$_q$-Ar), 129.7 (C$_4$), 127.3 (C$_3$), 111.4 (t, $J_{C\text{-}F}$ = 22.2 Hz, C$_q$-Ar), 62.8 (C$_1$), 52.3 (C$_5$) ppm.

^{19}F NMR (376.7 MHz, DMSO-d$_6$): δ -142.58 (dd, 2F, *J* = 23.4, 7.6 Hz, F$_a$), -153.35 (t, 1F, *J* = 22.2 Hz, F$_c$), -162.37 (td, 2F, *J* = 23.8, 8.3 Hz, F$_b$) ppm.

HRMS (ES+) calculated *m/z* for $C_{16}H_{11}F_5NO_3^+$ [M+H]$^+$ 360.0654, found 360.0654.

Methyl (*E*)-4-((2-(2-Fluorobenzoyl)hydrazineylidene)methyl)benzoate (S5)

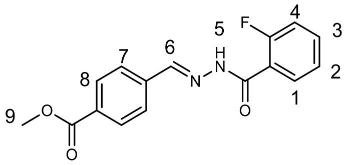

Methyl 4-formylbenzoate (0.138 g, 0.84 mmol) and 2-fluorobenzhydrazide (0.123 g, 0.80 mmol) were dissolved in 1:1 DCM/methanol (20 mL). Then, glacial acetic acid (6% v/v) was added and the mixture was stirred at room temperature for 22 hours. The solvent was then removed under reduced pressure to recover the crude product. The solid was then recrystallized from methanol to recover a white powder (0.164 g, 68%).

^{1}H NMR (499.9 MHz, DMSO-d$_6$): δ 12.05 (bs, 2H, H-5 major and minor conformers), 8.39 (s, 1H, H-6, major conformer), 8.14 (s, 1H, H-6, minor conformer), 8.03 (d, *J* = 8.1 Hz, 2H, H-8, major conformer), 7.92 (d, *J* = 8.1 Hz, 2H, H-8, minor conformer), 7.87 (d, *J* = 8.2 Hz, 2H, H-7, major conformer), 7.69 (td, *J* = 7.4, 1.8 Hz, 1H, H-1 major conformer), 7.64 – 7.50 (m, 4H, H-7 minor conformer, H-1 minor conformer, H-3 major conformer, H-3 minor conformer), 7.40 – 7.28 (m, 4H, H-4 major conformer, H-4 minor conformer, H-2 minor conformer, H-2 major conformer), 3.87 (s, 3H, H-9, major conformer), 3.83 (s, 3H, H-9, minor conformer) ppm.

^{13}C NMR (125.8 MHz, DMSO-d$_6$): δ 167.4 (C=O, minor conformer), 165.9 (COOMe, major conformer), 165.8 (COOMe, minor conformer), 160.7 (C=O, major conformer), 159.2 (d, $J_{C\text{-}F}$ = 249.3 Hz, Ar-F, major conformer), 158.5 (d, $J_{C\text{-}F}$ = 247.6 Hz, Ar-F, minor conformer), 146.7 (C$_6$, major conformer), 143.0 (C$_6$, minor conformer), 138.6 (C$_q$-Ar, major conformer), 138.4 (C$_q$-Ar, minor conformer), 133.1 (d, $J_{C\text{-}F}$ = 8.4 Hz, H-3, major conformer), 132.2 (d, $J_{C\text{-}F}$ = 8.3 Hz, H-3, minor conformer), 130.6 (C$_q$-Ar, major conformer), 130.3 (C$_q$-Ar, minor conformer), 130.2 (d, $J_{C\text{-}F}$ = 2.1 Hz, C$_1$, major conformer), 129.9 (d, $J_{C\text{-}F}$ = 2.0Hz, C$_1$, minor conformer), 129.7 (C$_8$, major conformer), 129.6 (C$_8$, minor conformer), 127.4 (C$_7$, major conformer), 126.8 (C$_7$, minor conformer), 124.7 (d, $J_{C\text{-}F}$ = 2.9 Hz, C$_2$, major conformer), 124.3 (d, $J_{C\text{-}F}$ = 2.4 Hz,C$_2$,

minor conformer), 123.7 (d, $J_{C\text{-}F}$ = 16.8 Hz, C_q-Ar, minor conformer), 123.1 (d, $J_{C\text{-}F}$ = 15.0 Hz, C_q-Ar, major conformer), 116.3 (d, $J_{C\text{-}F}$ = 21.7 Hz, C_4, major conformer), 115.4 (d, $J_{C\text{-}F}$ = 21.8 Hz, C_4, minor conformer), 52.3 (O-CH_3, major conformer), 52.3 (O-CH_3, minor conformer) ppm.
^{19}F NMR (470.4 MHz, DMSO-d$_6$): δ -112.69 (s, 1F, minor conformer), -113.94 (s, 1F, major conformer) ppm.
HRMS (ES+) calculated *m/z* for $C_{16}H_{13}FN_2NaO_3$ $[M+Na]^+$ 323.0802 found 323.0793.

(*E*)-4-((((Perfluorophenyl)methoxy)imino)methyl)benzoic acid (3)

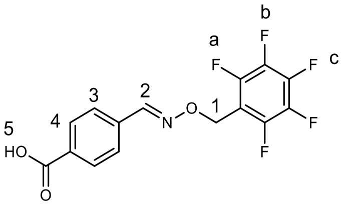

O-(2,3,4,5,6-Pentafluorobenzyl) hydroxylamine hydrochloride (0.20 g, 0.80 mmol) was dissolved in 1:1 DCM/methanol (20 mL) and 4-formylbenzoic acid (0.132 g, 0.88 mmol) was added followed by glacial acetic acid (6% v/v) and the mixture was stirred at room temperature for 24 hours. The solvent was then removed under reduced pressure to recover the crude product. The solid was then recrystallized from the minimum volume of aqueous ethanol to recover white crystals (0.131 g, 0.38 mmol, 48%).
^{1}H NMR (499.9 MHz, DMSO-d$_6$): δ 13.12 (s, 1H, H-5), 8.38 (s, 1H, H-2), 7.96 (d, 2H, *J* = 8.4 Hz, H-4), 7.69 (d, 2H, *J* = 8.3 Hz, H-3), 5.30 (s, 2H, H-1) ppm.
^{13}C NMR (125.8 MHz, DMSO-d$_6$): 166.8 (C=O), 149.8 (C_2), 145.23 (d, $J_{C\text{-}F}$ = 239.1 Hz, Ar-F), 140.92 (d, $J_{C\text{-}F}$ = 251.3 Hz, Ar-F), 137.0 (d, $J_{C\text{-}F}$ = 248 Hz, Ar-F), 135.4 (C_q-Ar), 132.1(C_q-Ar), 129.8 (C_4), 127.1 (C_3), 111.1 (t, $J_{C\text{-}F}$ = 21.0 Hz, C_q-Ar), 62.7 (C_1) ppm.
^{19}F NMR (470.4 MHz, DMSO-d$_6$): δ -142.58 (dd, 2F, *J* = 23.7, 8.1 Hz, F_a), -153.44 (t, 1F, *J* = 22.2 Hz, F_c), -162.41 (td, 2F, *J* = 23.2, 7.9 Hz, F_b) ppm.
HRMS (ES-) calculated *m/z* for $C_{15}H_8F_5NO_3$ $[M-H]^-$ 344.0352, found 344.0352.

(*E*)-benzaldehyde *O*-((perfluorophenyl)methyl) oxime (S6)

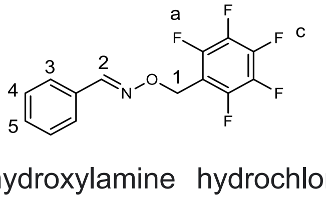

O-(2,3,4,5,6-Pentafluorobenzyl) hydroxylamine hydrochloride (0.20 g, 0.80 mmol) was dissolved in 1:1 DCM/methanol (20 mL) and benzaldehyde (0.085 mL, 0.84 mmol) was added followed by glacial acetic acid (6% v/v) and the mixture was stirred at room temperature for 22 hours. The solvent was then removed under reduced pressure to recover the crude

product. The solid was then recrystallized from the minimum volume of ethanol to recover a white powder (0.143 g, 0.47 mmol, 59%).

^{1}H NMR (500.1 MHz, DMSO-d_6): δ 8.28 (s, 1H, H-2), 7.59-7.56 (m, 2H, H-3), 7.43-7.41 (m, 3H, H-4,5), 5.26 (s, 2H, H-1) ppm.

^{13}C NMR (125.8 MHz, DMSO-d_6): δ 150.4 (C_2), 145.2 (d, J_{C-F} = 249.6 Hz, Ar-F), 140.9 (d, J_{C-F} = 256.8 Hz, Ar-F), 137.0 (d, J_{C-F} = 231.8 Hz, Ar-F), 131.4 (C_q-Ar), 130.4 (C_4),128.9 (C_5), 127.0 (C_3), 111.2 (C_q-Ar), 62.4 (C_1) ppm.

^{19}F NMR (470.4 MHz, DMSO-d_6): δ -140.82- -143.71 (m, 2F, F_a), -153.54 (t, 1F, J_{C-F} = 22.2 Hz, F_c), -162.49 (td, J_{C-F} = 23.7, 8.2 Hz, F_b) ppm.

HRMS (ES+) calculated *m/z* for $C_{14}H_9ONF_5^+$ [M+H]$^+$ 302.0599, found 302.0595.

(*E*)-4-((2-(2-Fluorobenzoyl)hydrazineylidene)methyl)benzoic acid (4)

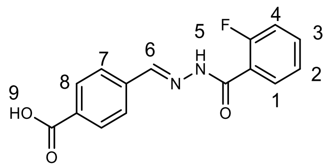

4-Formylbenzoic acid (0.215 g, 1.43 mmol) and 2-fluorobenzhydrazide (0.20 g, 1.3 mmol) were dissolved in 1:1 DCM/methanol (20 mL). Then, glacial acetic acid (6% v/v) was added and the mixture was stirred at room temperature for 22 hours. The solvent was then removed under reduced pressure to recover the crude product. The solid was then recrystallized from aqueous methanol to recover a white powder (0.266 g, 72%).

^{1}H NMR (500.1 MHz, DMSO-d_6): δ 12.13 (s, 1H, H-5, minor conformer), 12.03 (s, 1H, H-5, major conformer), 8.38 (s, 1H, H-6, major conformer), 8.13 (s, 1H, H-6, minor conformer), 8.01 (d, *J* = 8.4 Hz, 2H, H-8, major conformer), 7.90 (d, *J* = 8.2 Hz, 2H, H-8, minor conformer), 7.85 (d, *J* = 8.4 Hz, 2H, H-7, major conformer), 7.69 (td, *J* = 7.4, 1.8 Hz, 1H, H-2, major conformer), 7.65-7.53 (m, 5H, H-1, major and minor conformers, H-2 minor conformer), 7.52 (d, *J* = 8.2 Hz, 2H, H-7, minor conformer), 7.41-7.28 (m, 4H, H-4,3 major and H-4,3 minor conformers) ppm.

^{13}C NMR (125.8 MHz, DMSO-d_6): δ 167.4 (C=O minor), 166.9 (COOH major), 166.9 (COOH minor), 160.6 (C=O major), 159.2 (d, J_{C-F} = 249.3 Hz, Ar-F, major conformer), 158.5 (d, J_{C-F} =247.5 Hz, Ar-F, minor conformer), 146.8 (C_6, major conformer), 143.1 (C_6, minor conformer), 138.1 (C_q-Ar, major conformer), 138.0 (C_q-Ar, minor conformer), 133.0 (d, J_{C-F} = 8.5 Hz, C_1 major conformer), 132.2 (d, J_{C-F} = 8.5 Hz, C_1, minor conformer), 131.9 (C_q-Ar, major conformer), 131.6 (C_q-Ar, minor conformer), 130.2 (d, J_{C-F} = 2.8 Hz, C_2 major conformer), 129.8 (C_8, major conformer), 129.8 (C_8, minor conformer), 129.7 (d, J_{C-F} = 3.4 Hz, C_2 minor conformer), 127.3 (C_7, major conformer), 126.7 (C_7, minor conformer), 124.8 (d, J_{C-F} = 3.6 Hz, C_3 major conformer), 124.3 (d, J_{C-F} = 2.4 Hz, C_3 minor conformer), 123.8 (d, J_{C-F} = 17.1 Hz,

C_q-Ar), 123.1 (d, $J_{C\text{-}F}$ = 15.1 Hz, C_q-Ar), 116.3 (d, $J_{C\text{-}F}$ = 21.8 Hz, C_4 major conformer), 115.4 (d, $J_{C\text{-}F}$ = 21.7 Hz, C_4 minor conformer) ppm.

^{19}F NMR (376.5 MHz, DMSO-d_6): δ -112.72 (s, 1F, minor conformer), -113.96 (s, 1F, major conformer) ppm.

HRMS (ES-) calculated *m/z* for $C_{15}H_{10}FN_2O_3^-$ $[M-H]^-$ 285.0681, found 285.0676.

(*E*)-*N'*-Benzylidene-2-fluorobenzohydrazide (S7)[4]

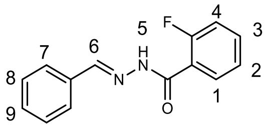

2-Fluorobenzhydrazide (0.200 g, 1.30 mmol) was dissolved in 1:1 DCM/methanol (20 mL), and then benzaldehyde (0.152 g, 145 µL, 1.43 mmol) was added followed by, glacial acetic acid (6% v/v) and then the mixture was stirred at room temperature for 24 hours. The solvent was then removed under reduced pressure to recover the crude product. The solid was then recrystallized from aqueous methanol to recover a white powder (0.115 g, 37%). Spectral data in agreement with the literature.[4]

^{1}H NMR (500.1 MHz, DMSO-d_6): δ 11.96 (s, 1H, H-5, minor conformer), 11.85 (s, 1H, H-5, major conformer), 8.33 (s, 1H, H-6, major conformer), 8.07 (s, 1H, H-6, minor conformer), 7.76 – 7.71 (m, 2H, H-7 major conformer), 7.67 (td, *J* = 7.4, 1.8 Hz, 1H, H-1 major conformer), 7.64 – 7.53 (m, 2H, H-3 major conformer, H-3 minor conformer), 7.52-7.44 (m, 7H , H-4 major conformer, H-4 minor conformer, H-8 major conformer, H-1 minor conformer, H-7 minor conformer), 7.44–7.26 (m, 6H, H-9 major conformer, H-9 minor conformer, H-8 minor conformer, H-2 major conformer, H-2 minor conformer) ppm.

^{13}C NMR (125.8 MHz, DMSO-d_6): δ 167.2 (C=O, minor conformer), 160.5 (C=O, major conformer), 159.2 (d, $J_{C\text{-}F}$ = 248.9 Hz, Ar-F, major conformer), 158.4 (d, $J_{C\text{-}F}$ = 247.5 Hz, Ar-F, minor conformer), 148.0 (C_6, major conformer), 144.0 (C_6, minor conformer), 134.1 (C_q-Ar), 134.0 (C_q-Ar), 132.9 (d, $J_{C\text{-}F}$ = 8.4 Hz, C_3, major conformer), 132.0 (d, $J_{C\text{-}F}$ = 8.4 Hz, C_3, minor conformer), 130.3 (C_8, major conformer), 130.2 (d, $J_{C\text{-}F}$ = 2.4 Hz, C_1, major conformer), 129.9 (C_8, minor conformer), 129.6 (d, $J_{C\text{-}F}$ = 3.5 Hz, C_1, minor conformer), 128.9 (C_9, major conformer), 128.8 (C_9, minor conformer), 127.2 (C_7, major conformer), 126.6 (C_7, minor conformer), 124.7 (d, $J_{C\text{-}F}$ = 3.5 Hz, C_2, major conformer), 124.2 (d, $J_{C\text{-}F}$ =3.1 Hz, C_2, minor conformer), 124.0 (d, $J_{C\text{-}F}$ = 18.2 Hz, C_q-Ar, minor conformer), 123.3 (d , $J_{C\text{-}F}$ = 15.4 Hz, C_q-Ar, major conformer), 116.2 (d, $J_{C\text{-}F}$ = 21.8 Hz, C_4, major conformer), 115.3 (d , $J_{C\text{-}F}$ = 21.7 Hz, C_4, minor conformer) ppm.

^{19}F NMR (376.7 MHz, DMSO-d_6): δ -112.78 (s, 1F, minor conformer), -114.06 (s, 1F, major conformer) ppm.

HRMS (ES+) calculated *m/z* for $C_{14}H_{12}FN_2O$ $[M+H]^+$ 243.0934 found 243.0923 (–4.5 ppm).

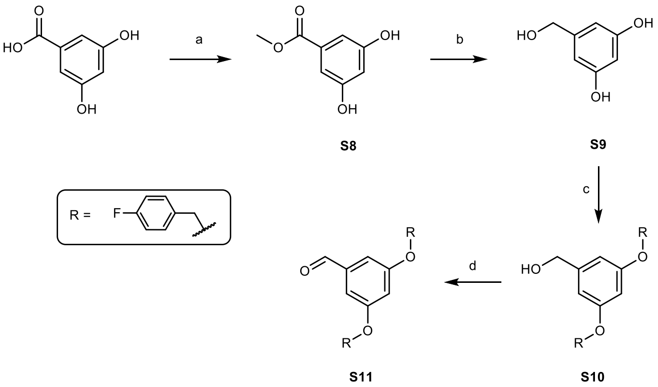

Scheme S1 Synthesis of aldehyde **S11**. Reagents and conditions: (a) c. H_2SO_4, MeOH, 80 °C, overnight, 82%; (b) i) $LiAlH_4$ ii) H^+, THF, 60 °C, 6 h, 82%; (c) 4-fluorobenzyl bromide, K_2CO_3, 18-crown-6, acetone, reflux, overnight, 32%; (d) i) CO_2Cl_2, DMSO ii) Et_3N, DCM, –78 °C, 90%.

Methyl 3,5-dihydroxybenzoate (S8) [5]

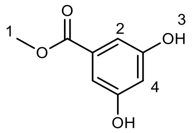

Concentrated sulfuric acid (228 µL) was added to a solution of 3,5-dihydroxybenzoic acid (11.0 g, 71.4 mmol) in methanol (290 mL) and stirred at 60 °C for 24 h. After cooling to room temperature, brine (100 mL) was added and product extracted with ethyl acetate (3 x 100 mL). The combined organic layers were then washed with brine (4 x 200 mL), dried over $MgSO_4$, and the solvent was removed under reduced pressure to give an off-white solid (9.85 g, 58.6 mmol, 82%). Spectral data in agreement with the literature.[5]

^{1}H NMR (400.1 MHz, DMSO-d$_6$): δ 9.67 (s, 2H, H-3), 6.82 (d, *J*=2.2 Hz, 2H, H-2), 6.44 (t, *J*=2.3, 1H, H-4), 3.79 (s, 3H, H-1) ppm.

5-(Hydroxymethyl)benzene-1,3-diol (S9) [6]

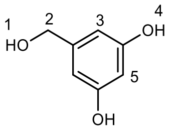

Lithium aluminium hydride (2.07 g, 54.5 mmol) was slowly suspended in dry THF (200 mL) with stirring under an atmosphere of N_2. Compound **S8** (1.84 g, 10.9 mmol) was dissolved in dry THF (50 mL) and slowly added via dropping funnel. Then, the reaction mixture was heated to reflux for 6 h, and then stirring continued at room temperature overnight. Particular care was taken to wash any lithium aluminium hydride residues formed on the flask back into solution. Next, the reaction mixture was cooled down to 0 °C with an ice/water bath, and saturated sodium sulfate solution (100 mL) was added dropwise. Afterwards, 1 M HCl was added until all solid residues were dissolved. The product was extracted with ethyl acetate (3 x 200 mL), then the combined organic layers were concentrated and washed with brine (3 x 200 mL), dried over $MgSO_4$, and the solvent was removed under reduced pressure. The crude

product was purified by column chromatography (SiO_2, DCM/acetone, 6:4, *v/v*) to give a white solid (0.54 g, 3.9 mmol, 35 %). Spectral data in agreement with the literature.[6]

^{1}H NMR (400.1 MHz, DMSO-d$_6$): δ 9.08 (s, 2H, H-4) 6.16 (d, *J*= 2.2 Hz, 2H, H-3), 6.04 (t, *J*= 2.2 Hz, 1H, H-5), 5.00 (t, *J*= 5.7 Hz, 1H, H-1), 4.29 (d, *J*=5.7, 2H, H-2) ppm.

(3,5-bis((4-Fluorobenzyl)oxy)phenyl)methanol (S10)

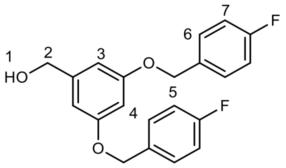

Alcohol **S9** was dissolved in acetone (50 mL) and 18-crown-6 (0.20 g, 0.8 mmol) and K_2CO_3 (1.33 g, 9.6 mmol) were added. Next, 4-fluorobenzyl bromide (0.96 mL, 1.46 g, 7.7 mmol) was added dropwise and the reaction mixture was stirred at reflux overnight. After cooling down to room temperature, solvent was removed under reduced pressure and ethyl acetate was added (100 mL). The organic phase was washed with water (2 x 100 mL) and brine (2 x 100 mL). After drying over $MgSO_4$, the solvent was removed under reduced pressure and the crude product was purified by column chromatography (SiO_2, petroleum ether/ethyl acetate, 2:1, *v/v*) to give a white solid (0.44 g,1.2 mmol, 32%).

^{1}H NMR (400.1 MHz, $CDCl_3$): δ 7.41 – 7.36 (m, 4H, H-6/7), 7.10 – 7.04 (m, 4H, H-6/7), 6.62 (d, *J* = 2.3 Hz, 2H, H-3), 6.51 (t, *J* = 2.3 Hz, 1H, H-4), 4.99 (s, 4H, H-5), 4.63 (d, *J* = 4.6 Hz, 2H, H-2) ppm.

^{19}F NMR (376.5 MHz, $CDCl_3$): δ −114.1 ppm.

^{13}C NMR (100.6 MHz, $CDCl_3$): δ 163.8, 160.1, 143.7, 132.7, 129.5, 115.6, 105.9, 101.4, 69.5, 65.4 ppm.

HRMS (ES$^+$) calculated *m/z* for $C_{21}H_{18}F_2NaO_3$ $[M+Na]^+$ 379.1116, found 379.1108.

3,5-bis((4-Fluorobenzyl)oxy)benzaldehyde (S11)

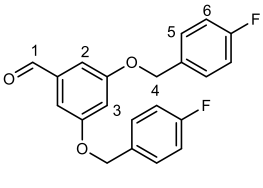

Activated molecular sieves and dry DCM (80 mL) were placed in a reaction vessel under Ar atmosphere and cooled to –78 °C using a dry ice/acetone bath. Then, oxalyl chloride (3.45 mL, 5.18 g, 40.8 mmol) was added. Next, DMSO (5.72 ml, 6.29 g, 80.5 mmol) was added dropwise and the reaction was stirred for 15 min. Afterwards, a solution of **S10** (2.90 g, 8.1 mmol) in dry DCM (20 mL) was added dropwise and the reaction was stirred for 30 min. Lastly, triethylamine (17.0 mL, 12.4 g, 122 mmol) was added dropwise and the reaction was stirred for 30 min. Then, the cooling bath was removed, and the reaction

mixture was allowed to warm up to room temperature while DMS was expelled under air flow. The molecular sieves were filtered off and the solvent was removed under reduced pressure. The dry residue was redissolved in DCM (200 mL), washed with 0.1 M HCl (3 x 200 mL) and with brine (2 x 200 mL). The organic layer was dried over $MgSO_4$, then the solvent was removed under reduced pressure to give a yellow solid (2.59 g, 7.3mmol, 90%).

^{1}H NMR (400.1 MHz, $CDCl_3$): δ 9.90 (s, 1H, H-1), 7.42 – 7.38 (m, 4H, H-5/6), 7.11– 7.06 (m, 6H, H-5/6 and H-2), 6.83 (t, *J* = 2.3 Hz, 1H, H-3), 5.05 (s, 4H, H-4) ppm.
^{19}F NMR (376.5 MHz, $CDCl_3$): δ −113.7 ppm.
^{13}C NMR (100.6 MHz, $CDCl_3$): δ 191.8, 164.0, 160.4, 138.6, 132.1, 129.6, 115.9, 108.8, 108.5, 69.9 ppm.
HRMS (ES$^-$) calculated *m/z* for $C_{21}H_{15}F_2O_3$ [M–H]$^-$ 353.0995, found 353.0993.

(*E*)-4-(2-(3,5-bis((4-Fluorobenzyl)oxy)benzylidene)hydrazine-1-carbonyl)benzoic acid (5)

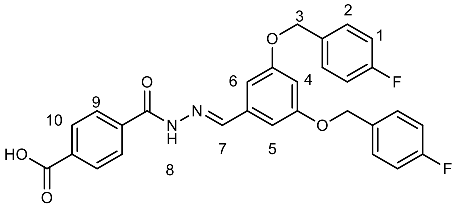

Aldehyde **S11** (0.298 g, 0.84 mmol) and 4-(hydrazinocarbonyl) benzoic acid (0.144 g, 0.8 mmol) were dissolved in methanol (50 mL). To this solution, acetic acid (6% v/v) was added and mixture left to stir at room temperature for 23 hours. The solvent was then removed under reduced pressure to recover the crude product. The product was dissolved in ethanol, then filtered to remove an insoluble yellow by-product. After removing the solvent under reduced pressure, recrystallisation in toluene afforded the pure product as a white powder (0.075 g, 18%).
^{1}H NMR (499.9 MHz, DMSO-d_6): δ 12.04 (s, 1H, H-8), 8.38 (s, 1H, H-7), 8.07 (d, 2H, *J*= 8.4 Hz, H-10), 8.01 (d, 2H, *J*= 8.2 Hz, H-9), 7.56-7.47 (m, 4H, H-2), 7.27-7.19 (*apparent* t, *J*= 8.9 Hz, 4H, H-1), 7.00 (d, 2H, *J*= 2.3 Hz, H-5,6), 6.77 (t, 1H, *J*= 2.3 Hz, H-4), 5.14 (s, 4H, H-3) ppm.
^{13}C NMR (125.8 MHz, DMSO-d_6): δ 166.8 (C=O), 162.8 (d, $J_{C\text{-}F}$ = 243.7Hz, Ar-F), 162.5 (HNC=O), 159.7 (Ar-O), 148.1(C-7), 137.1 (C_q), 136.3 (C_q), 134.0 (C_q), 133.1 (d, $J_{C\text{-}F}$ = 2.6 Hz, C_q), 130.1 (d, $J_{C\text{-}F}$ = 8.5 Hz, C-2), 129.4 (C-10), 127.9 (C-9), 115.3 (d, $J_{C\text{-}F}$ = 21.6 Hz,C-1), 106.1 (C-5), 104.0 (C-4), 68.7 (C-3) ppm.
^{19}F NMR (470.4 MHz, DMSO-d_6): δ -114.38 (s, 2F) ppm.
HRMS (ES-) calculated *m/z* for $C_{29}H_{21}F_2N_2O_5$ [M-H]$^-$ 515.1424 found 515.1426.

(*E*)-*N'*-(3,5-bis((4-Fluorobenzyl)oxy)benzylidene)-4-fluorobenzohydrazide (7)

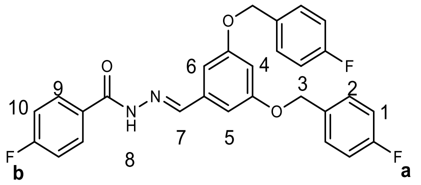

Aldehyde **S11** (0.298 g, 0.84 mmol) and 4-fluorobenzhydrazide (0.123 g, 0.8 mmol) were dissolved in 1:1 DCM/methanol (50 mL). To this solution, acetic acid (6% v/v) was added and the mixture left to stir at room temperature for 22 hours. The solvent was then removed under reduced pressure to recover the crude product. The solid was then recrystallized from methanol to recover a white powder (0.319 g, 0.65 mmol, 80%).

^{1}H NMR (500.1 MHz, DMSO-d_6): δ) 11.92 (s, 1H, H-7), 8.36 (s, 1H, H-6), 8.03-7.94 (m, 2H, H-8), 7.55-7.47 (m, 4H, H-2), 7.37 (t, 2H, *J*= 8.7 Hz, H-9), 7.23 (t, 4H, *J*= 8.8 Hz, H-1), 6.99 (d, 2H, *J*= 1.7 Hz, H-5), 6.76 (t, 1H, *J*= 2.4 Hz, H-4), 5.13 (s, 4H, H-3) ppm.

^{13}C NMR (125.8 MHz, DMSO-d_6): δ 164.2 (d, $J_{C\text{-}F}$ = 249.3 Hz, Ar-F_b), 162.8 (C=O), 161.8 (d, $J_{C\text{-}F}$ = 243.6 Hz, Ar-F_a), 159.7 (C_q), 147.6 (C_q), 136.4 (C_q), 133.1 (d, $J_{C\text{-}F}$ = 3.0 Hz, C_q), 130.4 (d, $J_{C\text{-}F}$ = 9.0 Hz, C-8), 130.1 (d, $J_{C\text{-}F}$ = 8.2 Hz, C-2), 129.8 (C_q), 115.5 (d, $J_{C\text{-}F}$ = 21.7 Hz, C-9), 115.3 (d, $J_{C\text{-}F}$ = 21.4 Hz, C-1), 106.1 (C-5), 103.9 (C-4), 68.7 (C-3) ppm.

^{19}F NMR (470.4 MHz, DMSO-d_6): δ -108.31 (s, 1F, F_b), -114.37 (s, 2F, F_a) ppm.

HRMS (ES+) calculated *m/z* for $C_{28}H_{21}F_3N_2NaO_3$ $[M+Na]^+$ 513.1396 found 513.1387.

3,5-bis((4-Fluorobenzyl)oxy)benzoic acid (S12)

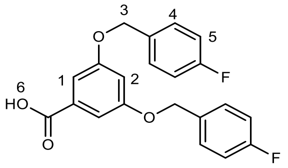

Aldehyde **S11** (0.308 g, 0.80 mmol) and potassium hydroxide (0.216 g, 3.85 mmol) were dissolved in methanol (20 mL). The solution was then heated under reflux for 19 hours. After cooling, 2M hydrochloric acid (20 mL) was added, causing a white precipitate to form. The solid was then collected via vacuum filtration and washed with copious amounts of water, to isolate the product as a white solid (0.264 g, 89%).

^{1}H NMR (500.1 MHz, DMSO-d_6) δ 7.53-7.46 (m, 4H, H-4), 7.25-7.18 (m, 4H, H-5), 7.15 (d, *J* = 2.3 Hz, 2H, H-1), 6.91 (t, *J* = 2.3 Hz, 1H, H-2), 5.12 (s, 4H, H-3) ppm.

^{13}C NMR (125.8 MHz, DMSO-d_6): 166.9 (C=O), 161.8 (d, $J_{C\text{-}F}$ = 243.8 Hz, Ar-F), 159.3 (Ar-C_q), 133.0 (d, $J_{C\text{-}F}$ = 3.0 Hz, Ar-C_q), 132.9 (C_q), 130.0 (d, $J_{C\text{-}F}$ = 8.3 Hz, C_4), 115.33 (d, $J_{C\text{-}F}$ = 21.3 Hz, C_5), 108.1 (C_1), 106.6 (C_2), 68.8 (C_3) ppm.

^{19}F NMR (376.5 MHz, DMSO-d_6): δ -114.35 (s, 2F) ppm.

HRMS (ES-) calculated *m/z* for $C_{21}H_{16}F_2O_4^-$ $[M-H]^-$ 369.0944 found 369.0933.

(E)-4-Fluoro-*N'*-(4-fluorobenzylidene)benzohydrazide (6) [7]

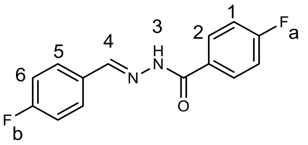

4-Fluorobenzhydrazide (0.70 g, 4.5 mmol) was dissolved in methanol (70 mL) and 4-benzaldehyde (0.73 mL, 6.8 mmol) was added followed by glacial acetic acid (6% v/v) and the mixture was stirred at room temperature for 21 hours. The solvent was then removed under reduced pressure to recover the crude product. The solid was then recrystallized from the minimum volume of DCM/MeOH (25:1). Et_2O was then added over ice to precipitate and the solution filtered under vacuum and solid washed with Et_2O to recover a white powder (0.859 g, 3.30 mmol, 73%). Spectral data in agreement with the literature.[7]

^{1}H NMR (500.1 MHz, DMSO-d$_6$): δ 11.90 (s, 1H, H-3), 8.45 (s, 1H, H-4), 8.06-7.95 (m, 2H, H-5), 7.84-7.74 (m, 2H, H-2), 7.37 (*apparent* t, 2H, J = 8.7 Hz, H-6), 7.31 (*apparent* t, 2H, J = 8.8 Hz, H-1) ppm.

^{13}C NMR (125.8 MHz, DMSO-d$_6$): δ 164.6 (d, $J_{C\text{-}F}$ = 249.5 Hz, Ar-F$_b$), 163.6 (d, $J_{C\text{-}F}$ = 247.8 Hz, Ar-F$_a$), 162.5 (C=O), 147.2 (C$_4$), 131.4 (C$_q$-Ar), 130.9 (d, $J_{C\text{-}F}$ = 9.1 Hz, C$_5$), 130.3 (C$_q$-Ar), 129.8 (d, $J_{C\text{-}F}$ = 8.6 Hz, C$_2$), 116.4 (d, J = 22.0 Hz, C$_1$), 116.0 (d, J= 21.8 Hz, C$_6$) ppm.

^{19}F NMR (376.5 MHz, DMSO-d$_6$): δ -108.34 (s, 1F, F$_a$), -110.50 (s, 1F, F$_b$) ppm.

HRMS (ES+) calculated *m/z* for $C_{14}H_{10}F_2N_2O$ $[M+H]^+$ 261.0839, found 261.0828 (–4.2 ppm).

AuNP-nuc ligand synthesis

Compound **S13** was prepared as previously described.[8]

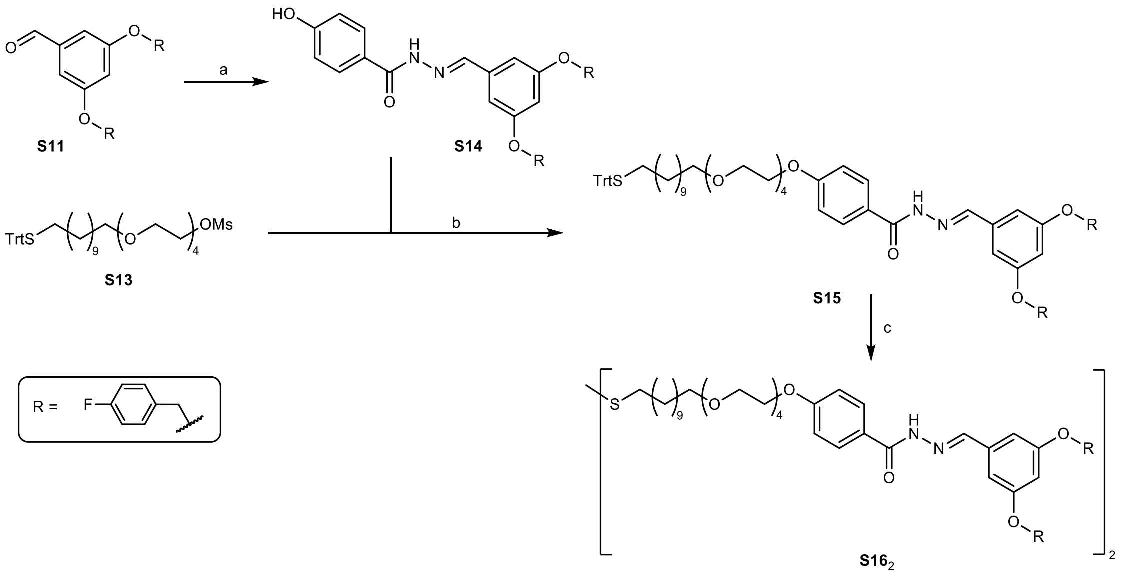

Scheme S2 Synthesis of disulfide pro-ligand **S16$_2$**. Reagents and conditions: (a) 4-hydroxybenzhydrazide, AcOH, MeOH, RT, overnight, 96%; (b) K_2CO_3, KI, DMF, 80 °C, overnight, 55%; (c) I_2, MeOH, RT, 15 min, 38%.

(*E*)-*N'*-(3,5-Bis((4-fluorobenzyl)oxy)benzylidene)-4-hydroxybenzohydrazide (S14)

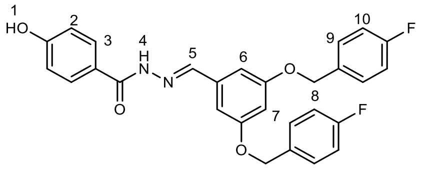

4-Hydroxybenzhydrazide (0.43 g, 2.8 mmol) and aldehyde **S11** (1.00 g, 2.8 mmol) were dissolved in MeOH/DCM (2:1, *v/v*, 75 mL) with 6% *v/v* (4.5 mL) of acetic acid. The reaction mixture was stirred overnight at room temperature. Next, the solvent was removed under reduced pressure and residue was redissolved in ethyl acetate (100 mL). Then the organic phase was washed with a saturated solution of $NaHCO_3$ (3 x 100 mL) and brine (2 x 100 mL). The organic layer was dried over $MgSO_4$, and the solvent was removed under reduced pressure to give a beige, waxy solid (1.33 g, 2.7 mmol, 96%).

^{1}H NMR (400.1 MHz, DMSO-d_6): δ 11.67 (s, 1H, H-4), 10.12 (s, 1H, H-1), 8.35 (bs, 1H, H-5), 7.81 (d, J = 8.8 Hz, 2H, H-3), 7.53 – 7.50 (m, 4H, H-9/10), 7.25 – 7.19 (m, 4H, H-9/10), 6.96 (bs, 2H, H-6), 6.86 (d, J = 8.8 Hz, 2H, H-2), 6.75 – 6.74 (m, 1H, H-7), 5.12 (s, 4H, H-8) ppm.

^{19}F NMR (376.5 MHz, DMSO-d_6): δ −114.4 ppm.

^{13}C NMR (100.6 MHz, DMSO-d_6): δ 163.0, 160.7, 160.6, 159.6, 146.6, 136.7, 133.1, 130.0, 129.7, 123.8, 115.4, 115.0, 105.9, 103.6, 68.7 ppm.

HRMS (ES$^+$) calculated *m/z* for $C_{28}H_{22}F_2N_2NaO_4$ $[M+Na]^+$ 511.1440, found 511.1436.

(*E*)-*N'*-(3,5-Bis((4-fluorobenzyl)oxy)benzylidene)-4-((1,1,1-triphenyl-14,17,20,23-tetraoxa-2-thiapentacosan-25-yl)oxy)benzohydrazide (S15)

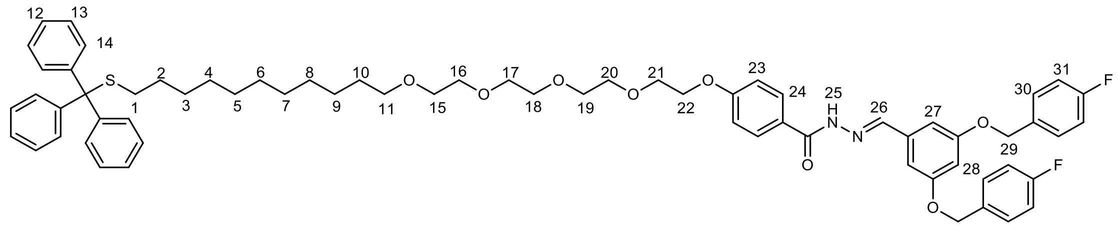

Hydrazone **S14** (1.90 g, 3.9 mmol) was dissolved in DMF (40 mL), then K_2CO_3 (1.61 g, 11.7 mmol) and KI (0.45 g, 2.7 mmol) were added and the mixture heated to 80 °C. Compound **S13** (3.27 g, 4.7 mmol) in DMF (20 mL) was then added dropwise and heating at 80 °C continued overnight. After cooling to room temperature, distilled water (50 mL) and ethyl acetate (100 mL) were added. The organic layer separated then washed with distilled water (2 x 100 mL) and with brine (3 x 100 mL). After drying over $MgSO_4$, the solvent was removed under reduced pressure and the crude product purified by column chromatography (SiO_2, DCM/acetonitrile, 6:1 → 3:1, *v/v*) to afford **S15** as a colourless oil (2.83 g, 2.6 mmol, 55%).

^{1}H NMR (400.1 MHz, $CDCl_3$): δ 9.96 (bs, 1H, H-25), 8.23 (bs, 1H, H-26), 7.86 (bs, 2H, H-24), 7.42 – 7.35 (m, 12H, H-13/14), 7.29-7.24 (m, 3H, H-12), 7.21 – 7.17 (m, 4H, H-30), 7.07 –

7.03 (m, 4H, H-31), 6.93 (bs, 2H, H-23), 6.91 (bs, 2H, H-27), 6.58 (t, 1H, H-28), 4.95 (bs, 4H, H-29), 4.15 – 4.13 (m, 2H, H-22), 3.86 – 3.83 (m, 2H, H-21), 3.76 – 3.55 (m, 12H, H-15-20), 3.42 (t, *J* = 7.3 Hz, 2H, H-11), 2.12 (t, *J* = 7.3 Hz, 2H, H-1), 1.87-1.83 (m, 4H, H-2/10),1.39 – 1.35 (m, 2H, H-9), 1.28 - 1.14 (m, 12H, H-3-8) ppm.

^{19}F NMR (376.5 MHz, $CDCl_3$): δ −114.0 ppm.

^{13}C NMR (100.6 MHz $CDCl_3$): δ 163.9, 162.0, 161.4, 160.1, 148.1, 145.2, 136.1, 132.5, 132.3, 129.7, 129.6, 129.5, 127.9, 126.6, 115.7, 115.5, 114.6, 106.6, 104.6, 71.7, 71.0, 70.8, 70.7, 70.2, 69.7, 69.6, 68.1, 67.7, 66.5, 32.2, 29.8, 29.7, 29.6, 29.3, 29.2, 29.1, 28.7, 26.2, 25.7 ppm.

HRMS (ES+) calculated *m/z* for $C_{66}H_{74}F_2N_2O_8SNa$ $[M+Na]^+$ 1115.5026, found 1115.5020.

4,4'-((((Disulfanediylbis(undecane-11,1-diyl))bis(oxy))bis(octane-8,1-diyl))bis(oxy))bis(*N'*-((*E*)-3,5-bis((4-fluorobenzyl)oxy)benzylidene)benzohydrazide) (S16$_2$)

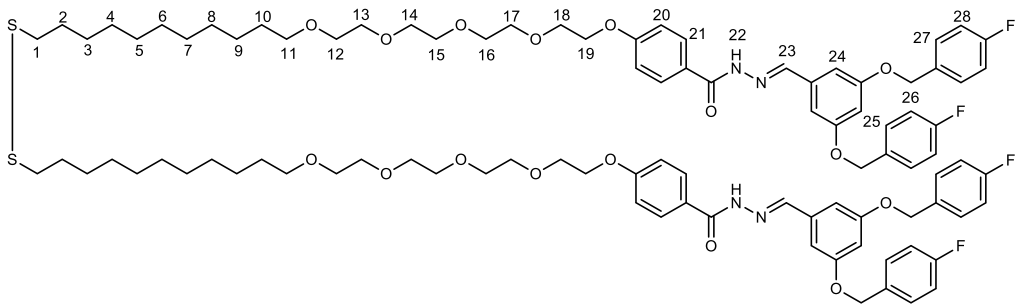

Thioether **S15** (2.83 g, 2.59 mmol) was dissolved in MeOH/DCM (45 mL, 2:1, *v/v*), then iodine (1.31 g, 5.18 mmol) was added. The reaction mixture was stirred for 15 min at room temperature, then quenched by adding a saturated solution of $NaHSO_3$ until the colour changed from brown to pale yellow. Next, the product was extracted into DCM (3 x 100 mL) and the combined organic layers were washed with brine (3 x 200 mL). After drying over $MgSO_4$, the solvent was removed under reduced pressure and the crude product was purified by column chromatography (SiO_2, DCM/acetonitrile, 4:1, *v/v*) to give **S16**$_2$ as a pale-yellow oil (1.65 g, 0.97 mmol, 38%).

^{1}H NMR (400.1 MHz, $CDCl_3$): δ 10.23 (bs, 2H, H-22), 8.27 (bs, 2H, H-23), 7.86 (bs, 4H, H-21), 7.39 – 7.34 (m, 8H, H-27), 7.05 (t, *J* = 8.8 Hz, 8H, H-28), 6.91 (m, *J* = 8.7 Hz, 8H, H-20/24), 6.57 (bs, 2H, H-25), 4.92 (bs, 8H, H-26), 4,14 – 4.12 (m, 4H, H-19), 3.84 (t, *J* = 7.3 Hz, 4H, H-18), 3.76 – 3.61 (m, 24H, H-12-17), 3.57 – 3.55 (m, 4H, H-11), 3.41 (t, *J* = 7.3 Hz 4H, H-1), 1.68 – 1.63 (m, 4H, H-2), 1.57 – 1.51 (m, 4H, H-10), 1.37 – 1.23 (m, 28H, H-3-9) ppm.

^{19}F NMR (376.5 MHz, $CDCl_3$): δ −114.1 ppm.

^{13}C NMR (100.6 MHz, $CDCl_3$): δ 163.6, 162.0, 161.7, 160.1, 147.9, 136.2, 132.5, 129.7, 128.9, 127.9, 115.6, 114.6, 106.6, 104.6, 71.7, 71.0, 70.7, 70.6, 70.2, 69.7, 69.6, 68.1, 67.7, 39.3, 29.8, 29.7, 29.6, 29.5, 29.3, 29.2, 28.6, 26.2, 25.8 ppm.

HRMS (ES+) calculated *m/z* for $C_{94}H_{119}F_4N_4O_{16}S_2$ $[M+H]^+$ 1699.7993, found 1699.7955.

3. Synthesis of MOFs

UiO-66-nanoparticles

UiO-66 was prepared via an adapted previously reported protocol.[9]

$ZrOCl_2$•$8H_2O$ (0.630 g, 1.96 mmol) was dissolved in DMF (90 mL) via ultrasonication. Then in a separate vial, terephthalic acid (1.50 g, 9.03 mmol) was dissolved in DMF (30 mL) via ultrasonication. The two solutions were then mixed in a 250 mL screw cap pyrex vial and glacial acetic acid (10.5 mL) was added. The solution was then heated to 90 °C in an oven for 18 hours. The solution was then removed from heating once the oven was cooled to 65 °C and allowed to further cool to room temperature. The MOF produce was then collected via centrifugation. Initially, the MOF was collected via centrifugation (14,500 rpm, 1 hour), followed by washing-dispersion cycles (3x DMF, 2x ethanol at 14,500 rpm for 30-45 min each). The solid was stored in an ethanolic solution at ~15 mg/mL until further use.

Aluminium fumarate nanoparticles

Sodium aluminate (0.45 g, 5.50 mmol) and fumaric acid (0.90 g, 7.75 mmol) were dissolved in water (50 mL) and stirred at 90 °C for 1 hour. After cooling to room temperature, the resulting solid was collected by collected via centrifugation (14,500 rpm, 10 min), followed by washing-dispersion cycles (2x water, 2x ethanol at 14,500 rpm for 10 minutes each). The solid was stored in an ethanolic solution at ~25 mg/mL until further use.

Functionalized UiO-66 MOF NPs

An aliquot of the sample stored in ethanolic solution was removed and dried to determine the mass concentration of suspended particles before functionalisation. 500 mg of UiO-66 (28.4 mL of a 17.6 mg/mL solution in ethanol) was added to a solution of hydrazone **1** (121 mg, 0.42 mmol) in DMF (21.6 mL). The two solutions were mixed and then left to stir at room temperature for 7 days. Initially, the MOF was collected via centrifugation (14,500 rpm, 40 min), followed by washing-dispersion cycles (3x DMF, 2x ethanol at 14,500 rpm for 30-45 min each). The solid was stored in an ethanolic solution, ~10 mg/mL until further use.

The same procedure was used for functionalisation with **2, 3**, **4**, **5**.

Functionalized Al-fum MOF NPs

An aliquot of the sample stored in ethanolic solution was removed and dried to determine the mass concentration of suspended particles before functionalisation. 250 mg of Al-fum (6.4 mL of a 39 mg/mL solution in ethanol) was diluted with additional portion of ethanol (12.4 mL), before addition of a solution of hydrazone **2** (155 mg, 0.54 mmol) in DMF (23.7 mL). The two solutions were mixed and then left to stir at room temperature for 7 days. Initially, the MOF was collected via centrifugation (14,500 rpm, 30 min), followed by washing-dispersion cycles

(3x DMF, 2x ethanol at 14,500 rpm for 30-45 min each). The solid was stored in an ethanolic solution, ~20 mg/mL until further use.
The same procedure was used for functionalisation with **1**, using 300 mg of Al-fum (11.2 of a 26.8 mg/mL solution in ethanol) and **1** (372 mg, 1.30 mmol)

Handling and storage of functionalized MOF NPs
MOF NPs were stored in ethanolic solution until further use.
Concentrations of solution were determined by drying 1 mL of MOF solution on a watch glass at 60 °C in an oven at atmospheric pressure.
For further use, aliquots of known concentration were removed and transferred to a centrifuge vial, then collect via 1 cycle at 14,800 rpm for 20 min. The ethanol supernatant was removed and MOF redispersed into fresh reaction solvent.
Alternatively, an aliquot of known concentration was removed and dried on a watch glass at 60 °C in an oven at atmospheric pressure.

4. Synthesis of AuNP-nuc

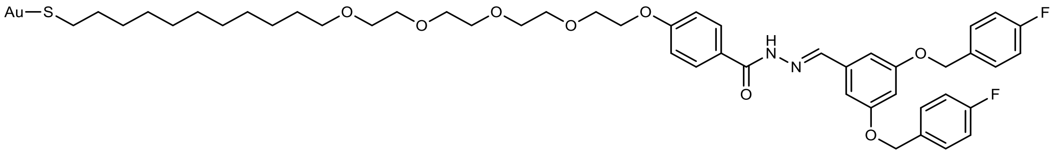

Disulfide pro-ligand S16$_2$ (212 mg, 0.125 mmol) was dissolved in THF (20 mL) in a flask previously washed with aqua regia. Next, $AuPPh_3Cl$ (202 mg, 0.410 mmol) was added, and the reaction mixture was heated to 50 °C while stirring at 1500 rpm. Finally, borane *tert*-butylamine complex (356 mg, 4.10 mmol) in THF (5 mL) was added rapidly. After 3 hours, heating was turned off and the reaction was allowed to cool to room temperature and stir for a further 1 hour. Nanoparticle precipitation was then achieved by addition of methanol (80 mL) followed by sonication (40 °C, 15 min) before sitting overnight in a fridge producing a black precipitate. The clear supernatant was discarded and this process was repeated a further twice with methanol and once with a DCM/hexane mix. Traces of volatile solvents were removed under a stream of compressed air to isolate 110 mg of **AuNP-nuc** as a black solid. For characterization see Section 21.

5. Characterisation of unfunctionalized UiO-66

UiO-66 was synthesised following procedure described in Section 3.

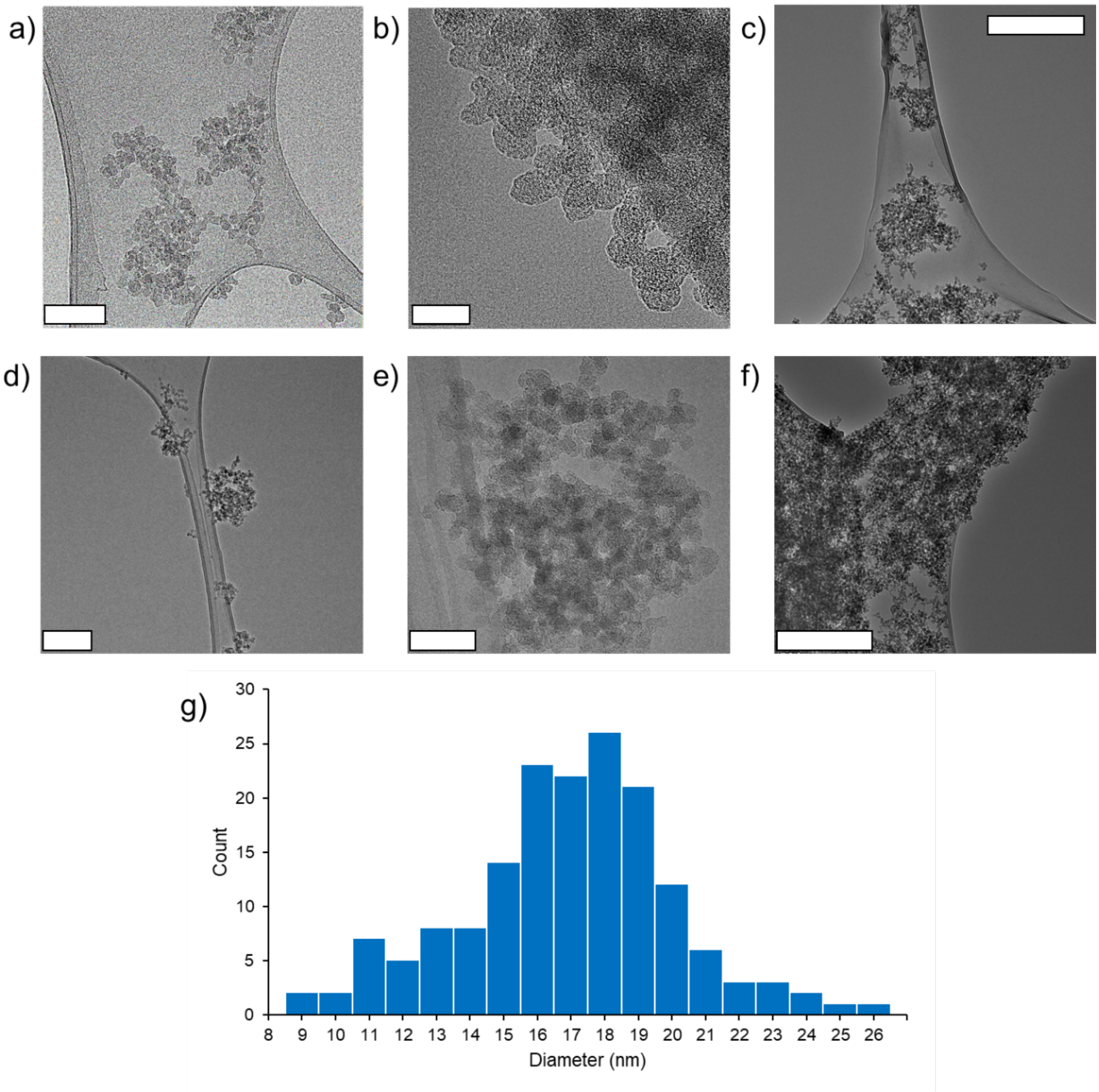

Figure S1 Representative transmission electron microscopy (TEM) images (a–f) and size distribution of the unfunctionalized UiO-66 <d_{TEM}> = 16.9 (3.1) nm (total count: 166 particles). Scale bars a) 100 nm, b) 20 nm, c) 500 nm, d) 200 nm, e) 50 nm, f) 500 nm.

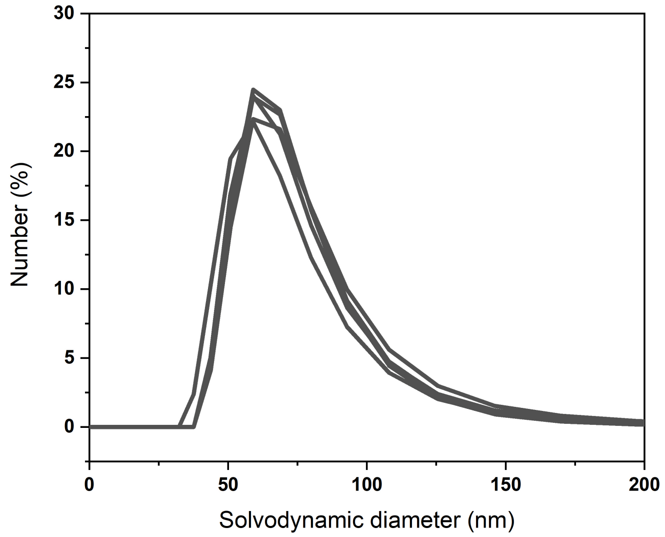

Figure S2 Dynamic light scattering (DLS) of unfunctionalized UiO-66 nanoparticles dispersed in ethanol (0.25 mg/mL) <d_{EtOH}> = 71 (2) nm.

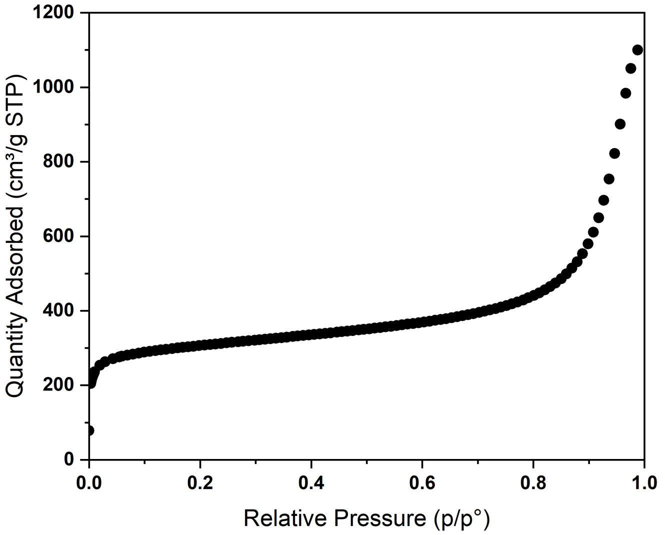

Figure S3 N_2 adsorption (77 K) plot of the unfunctionlized UiO-66 resulting in a BET surface area of 1170 m^2 g^{-1}.

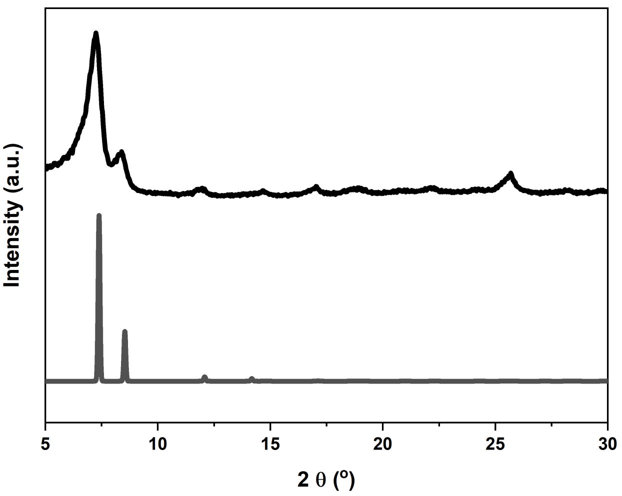

Figure S4 Powder X-ray diffraction of the as-synthesised unfunctionalized UiO-66 (top) against 'ideal' UiO-66 diffraction pattern reported (bottom).[10]

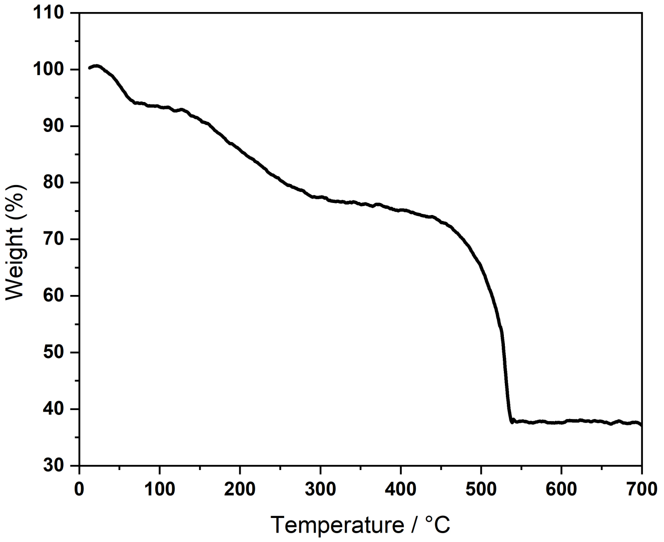

Figure S5 Thermogravimetric analysis (TGA) plot of UiO-66 displaying thermal stability up to 450 °C.

6. Characterisation of UiO-66-elec-4F

UiO-66-elec-4F was synthesised following the procedure described in Section 3.

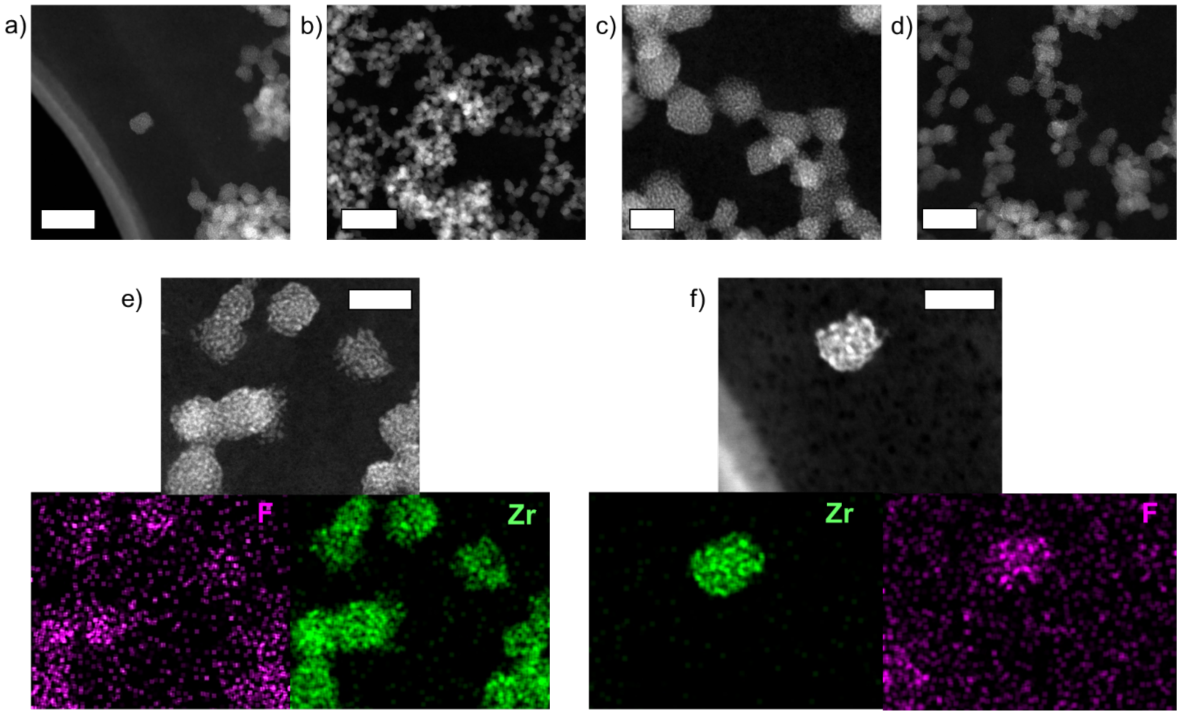

Figure S6. a–d) Additional HAADF STEM images of **UiO-66-elec-4F**. Scale bars: a) 50 nm, b) 100 nm, c) 20 nm and d) 50 nm. e–f) Additional STEM-EDX mappings of **UiO-66-elec-4F** (scale bars = 20 nm; green = Zr; pink = F)

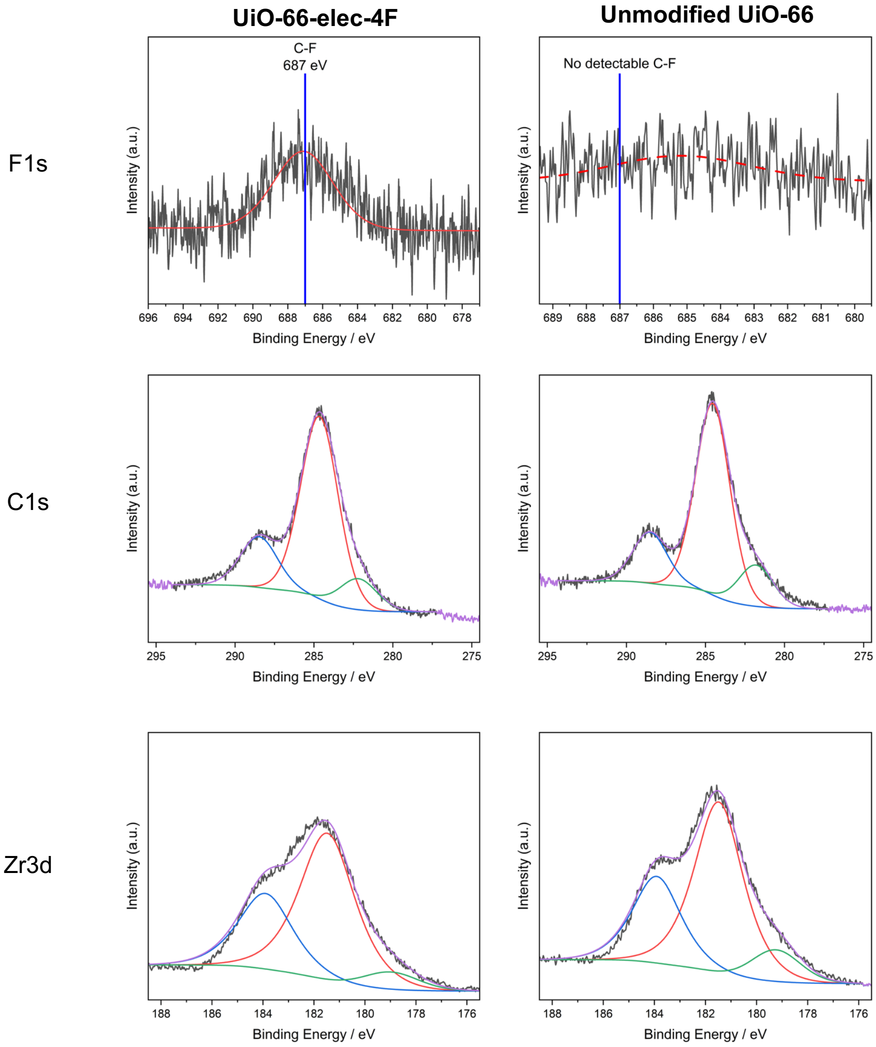

Figure S7 X-Ray photoelectron spectra showing the fluorine 1s, carbon 1s and Zr 3d regions for **UiO-66-elec-4F** (left-hand column) compared to the unmodified UiO-66 MOF (right-hand hand column).

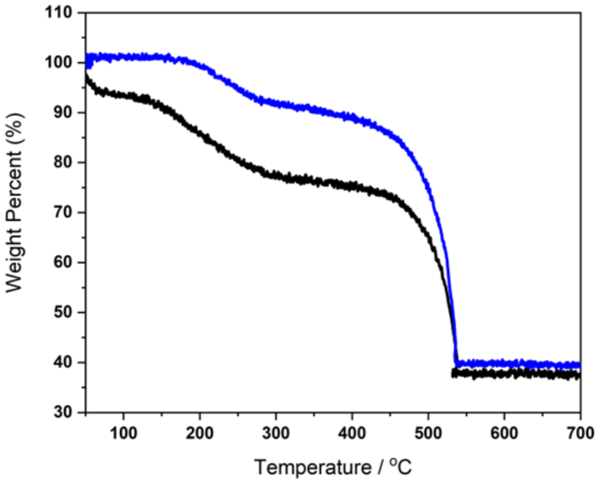

Figure S8 TGA plot of unfunctionalized UiO-66 (black) and **UiO-66-elec-4F** (blue).

Under acid digestion conditions, hydrazone **1** decomposed into components **4FHyd** and 4-fluorobenzoic acid (**S17**). In quantitative experiments (Section 7), the concentrations of all three species were summed to estimate the total concentration of **1** present in the initial sample.

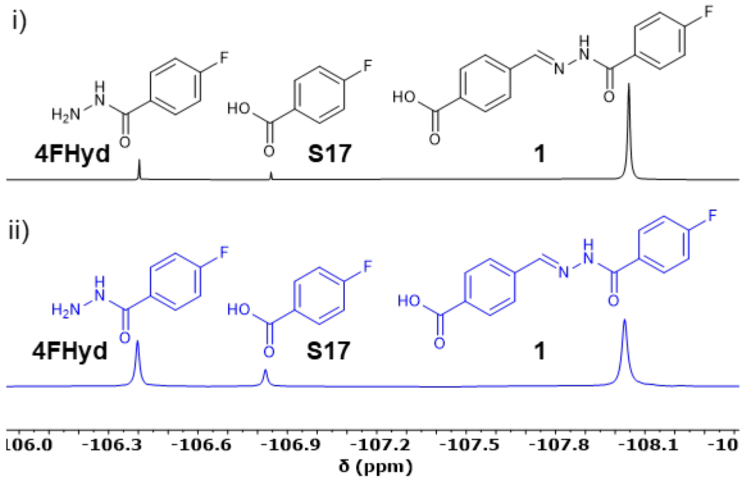

Figure S9 Acid digestion ^{19}F NMR spectra (376.7 MHz, DMSO-d_6), in which ~3 mg of MOF or molecule, is digested with conc. H_2SO_4 (30 μL) in DMSO-d_6. (i) authentic hydrazone **1**, (ii) **UiO-66-elec-4F**.

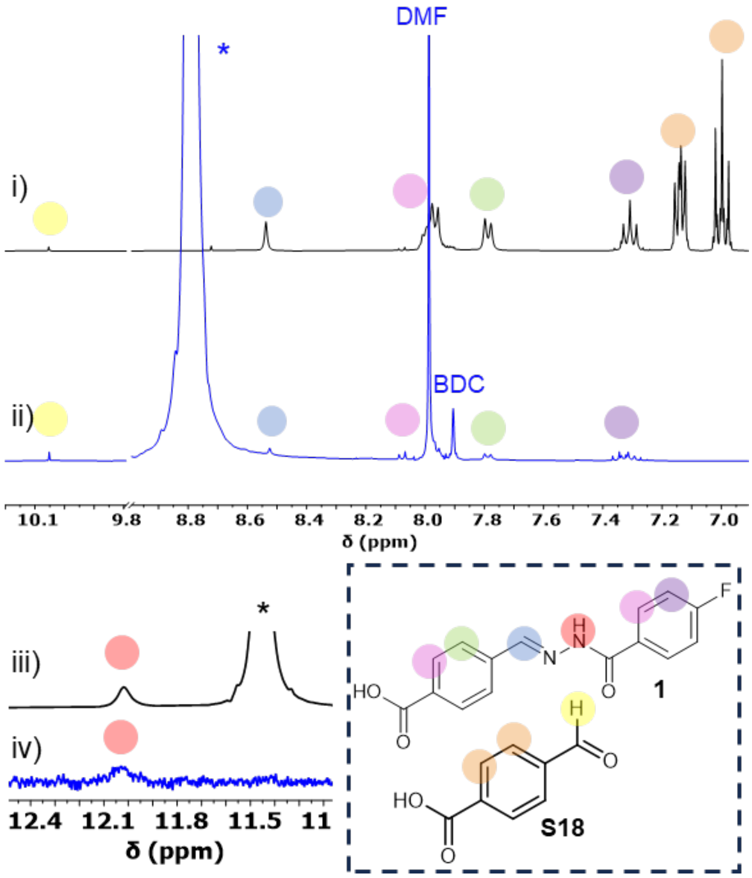

Figure S10 Acid digestion ^{1}H NMR spectra (DMSO-d_6) in which ~3 mg of MOF or molecule, is digested with conc. H_2SO_4 (30 μL) in DMSO-d_6, (i) authentic hydrazone **1** (400.3 MHz), (ii) **UiO-66-elec-4F** (400.1 MHz). Magnifications of high chemical shift regions for (iii) authentic hydrazone **1** (400.3 MHz), (iv) **UiO-66-elec-4F** (400.1 MHz). Spectra are labelled with protons corresponding to **1** and digestion product **S18**. 1,4-

Benzenedicarboxylic acid (BDC) linker from the MOF, and *N*,*N*-dimethylformamide (DMF) solvent used are also labelled. * = H_2SO_4.

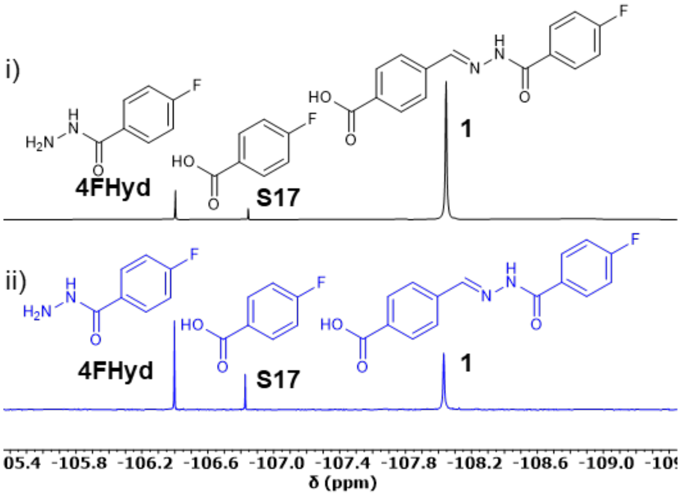

Figure S11 Acid digestion ^{19}F NMR spectra (376.7 MHz, DMSO-d_6) in which ~3 mg of MOF/molecule is digested with conc. H_2SO_4 (30 µL) in DMSO-d_6 of (i) authentic hydrazone **1** and (ii) **UiO-66-elec-4F** after activation at 150 °C for 16 h.

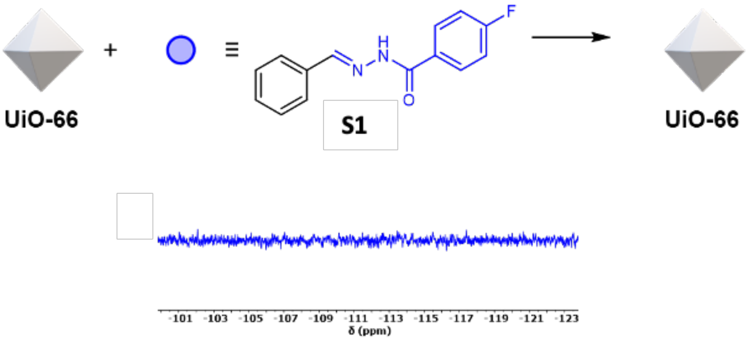

Figure S12 Control modification of UiO-66 using analogue hydrazone **S1** lacking a carboxylate binding site showing no coordination to the MOF surface. Conditions: UiO-66 (0.023 mmol $C_{48}H_{28}O_{32}Zr_6$), **S1** (0.033 mmol) in DMF (4 mL), RT, 7 d. ^{19}F NMR spectrum (376.7 MHz, DMSO-d_6) in which ~3 mg of MOF, is digested with conc. H_2SO_4 (30 µL) in DMSO-d_6 The spectrum shows no fluorine signal.

7. Quantitative NMR procedures

7.1 Acid digestion ^{1}H NMR spectroscopy

A 50 mM stock solution of maleic acid internal standard was prepared volumetrically in DMSO-d_6.

A precisely known mass of MOF (between 2–4 mg) was weighed and to this DMSO-d_6 (510 µL), maleic acid stock solution (60 µL) and concentrated H_2SO_4 (30 µL) were added. The solution was then sonicated until no solid was visible. A quantitative ^{1}H NMR spectrum was then recorded (d_1 = 30 s).

Line fitting was carried out on the processed spectrum and concentrations were determined relative to the signal for the maleic acid IS (5 mM). Since the sulfuric acid caused not only digestion of the MOF but also of the organic species, the concentrations of all species originating from the parent moiety were combined to estimate the total amount of hydrazone present in the sample.

7.2 Acid digestion ^{19}F NMR spectroscopy

A 100 mM stock solution of 4-fluorotoluene (4FT) internal standard was prepared volumetrically in DMSO-d_6.

A precisely known mass of MOF (between 2–4 mg) was weighed and to this DMSO-d_6 (510 µL), 4FT stock solution (60 µL) and concentrated H_2SO_4 (30 µL) were added. The solution was then sonicated until no solid was visible. A quantitative ^{19}F NMR spectrum was measured (d_1 = 25 s), with between 8–64 scans dependent upon the amount and type of MOF sample.

Line fitting was carried out on the processed spectrum and concentrations were determined relative to the signal for the 4FT internal standard (10 mM). Since the sulphuric acid caused not only digestion of the MOF but also of the organic species, the concentrations of all species originating from the parent moiety were combined to estimate the total amount of hydrazone present in the sample.

7.3 TFA stripping ^{19}F NMR spectroscopy

A stock solution of 10 mM 4FT was prepared volumetrically in a 10% *v*/*v* D_2O/DMF. This solution was used as a solvent to prepare a 100 mM TFA stock solution volumetrically.

A precisely known mass of MOF (between 2–4 mg) was weighed and added to the TFA stock solution (600 µL). The solution was then sonicated until fully dispersed. Quantitative ^{19}F NMR spectra were then measured (d_1 = 25 s), with between 8–64 scans dependent upon the amount and type MOF sample.

Line fitting was carried out on the processed spectrum and concentrations were determined relative to the signal for the 4FT internal standard (10 mM). In cases where hydrazone hydrolysis was observed, concentrations of all species originating from the parent moiety were combined to estimate the total amount of hydrazone present in the sample.

Repeated spectra were recorded over a period of 1 week to establish when all surface-coordinated material had been released.

7.4 Citric acid stripping ^{19}F NMR spectroscopy

A stock solution of 10 mM 4FT was prepared volumetrically in a 10% v/v D_2O/DMF solution. This solution was used as a solvent to prepare a 100 mM citric acid (CA) stock solution volumetrically.

A precisely known mass of MOF (between 2–4 mg) was weighed and added to the CA stock solution (600 µL). The solution was then sonicated until fully dispersed. Quantitative ^{19}F NMR spectra were measured (d_1 = 25 s), with between 8–64 scans dependent upon the amount and type MOF sample.

Line fitting was carried out on the processed spectrum and concentrations were determined relative to the signal for the 4FT internal standard (10 mM). In cases where hydrazone hydrolysis was observed, concentrations of all species originating from the parent moiety were combined to estimate the total amount of hydrazone present in the sample.

Repeated spectra were recorded over a period of 1 week to establish when all surface-coordinated material had been released.

8. Quantitative assessment of UiO-66-elec-4F surface functionalisation

8.1 Acid digestion ^{1}H NMR spectroscopy

Using the procedure outlined in Section 7.1.

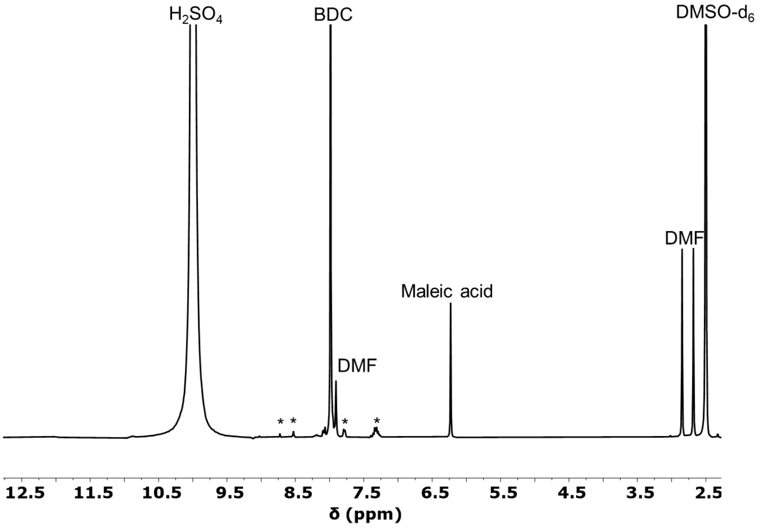

Figure S13 ^{19}F qNMR spectrum (400.3 MHz, DMSO-d$_6$) of **UiO-66-elec-4F** acid digestion (H_2SO_4). IS is maleic acid (5.12 mM). * = signals for hydrazone **1**.

Table S1 Processed data for the ^{1}H qNMR acid digestion spectrum of **UiO-66-elec-4F**

[**1**] / mM	Total quantity **1** present / μmol	Mass concentration **1** / μmol mg^{-1}
1.39	0.83	0.29

8.2 Acid digestion ^{19}F NMR spectroscopy

Using the procedure outlined in Section 7.2.

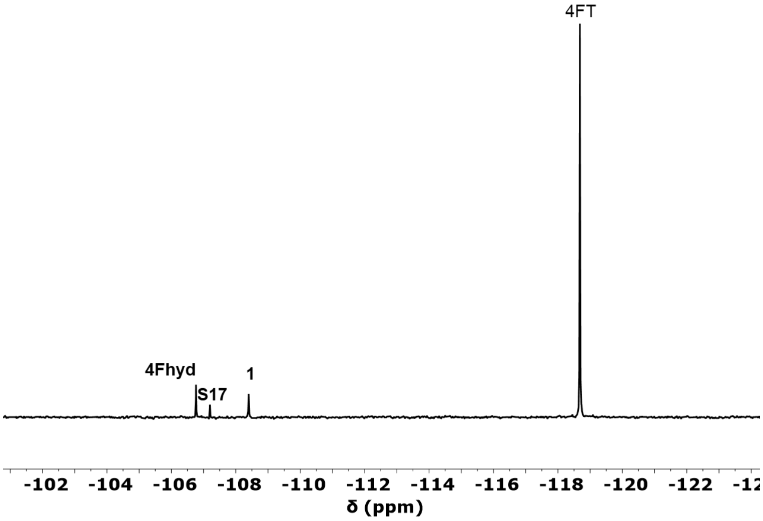

Figure S14 ^{19}F qNMR spectrum (376.7 MHz, DMSO-*d*$_6$) of **UiO-66-elec-4F** acid digestion (H_2SO_4). IS is 4FT (10 mM)

Table S2 Quantitative analysis of the acid digestion ^{19}F NMR spectrum of **UiO-66-elec-4F.**

[**1**] / mM	[**4Fhyd**] / mM	[**S17**] / mM	[**1**] + [**4Fhyd**] + [**S17**]/ mM	Total quantity **1** initially present / µmol	Mass concentration **1** / µmol mg^{-1}
1.709	0.242	0.024	1.975	1.185	0.352

8.3 TFA stripping ^{19}F NMR spectroscopy

Using the procedure outlined in Section 7.3.

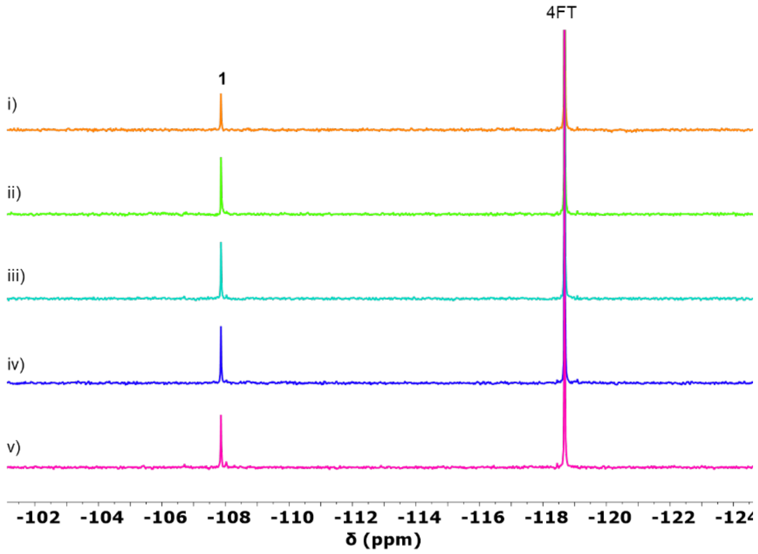

Figure S15 ^{19}F qNMR spectra (376.7 MHz, D_2O) of **UiO-66-elec-4F** TFA (100 mM in 10% *v*/*v* D_2O/DMF) stripping over time after (i) < 1 h, (ii) 24 h, (iii) 48 h and (iv) 7 d. IS =4FT (10 mM)

Table S3 Quantitative analysis of the TFA stripping ^{19}F NMR Spectra of **UiO-66-elec-4F.**

Time	[**1**] / mM	[**4Fhyd**] / mM	[**S17**] / mM	[**1**] + [**4Fhyd**] + [**S17**] / mM	Total quantity **1** initially present / µmol	Mass concentration **1** / µmol mg^{-1}
< 1 h [a]	1.000	0	0	1.000	0.600	0.195
24 h	1.659	0	0	1.659	0.996	0.324
48 h	1.705	0	0	1.705	1.023	0.333
72 h	1.757	0	0	1.757	1.054	0.343
1 week	1.365	0.350	0.094	1.809	1.857	0.354

[a] The first spectrum recorded immediately following sample preparation after a waiting time dependent on spectrometer availability.

8.4 Citric Acid stripping ^{19}F NMR spectroscopy

Using the procedure outlined in Section 7.4.

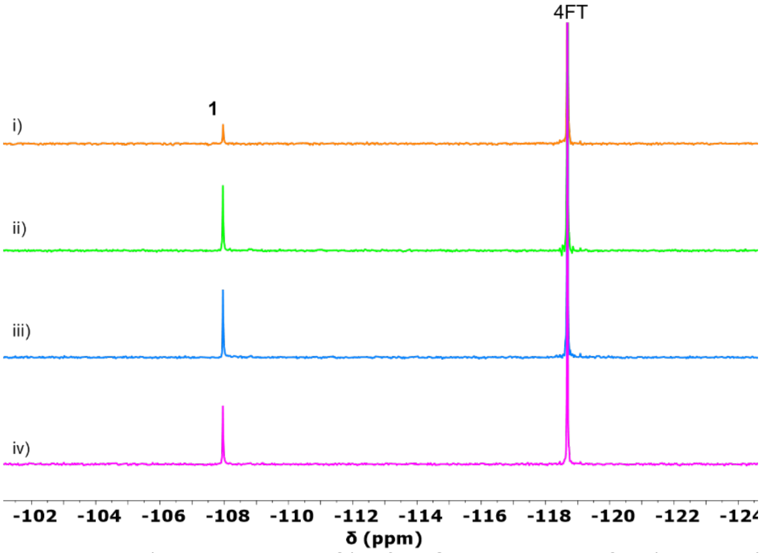

Figure S16 ^{19}F qNMR spectra (376.7 MHz, D_2O) of **UiO-66-elec-4F.** CA (100 mM) stripping 10% v/v D_2O/DMF over time after a) < 1 h, b) 24 h, c) 48 h and d) 7 d. IS is 4FT (10 mM)

Table S4 Quantitative analysis of the CA stripping ^{19}F NMR Spectra of **UiO-66-elec-4F.**

Time	[**1**] / mM	[**4Fhyd**] / mM	[**S17**] / mM	[**1**] + [**4Fhyd**] + [**S30**] / mM	Total quantity **1** initially present / µmol	Mass concentration **1** / µmol mg^{-1}
< 1 h [a]	0.543	0	0	0.543	0.326	0.105
24 h	1.721	0	0	1.721	1.032	0.333
48 h	1.731	0	0	1.731	1.038	0.335
8 days	1.709	0	0	1.709	1.025	0.331

[a] The first spectrum recorded immediately following sample preparation after a wait time dependant on spectrometer availability.

9. Estimation of surface functionalisation density

Assuming an average particle is pseudo spherical in shape with diameter equal to the mean size observed by TEM imaging (Figure S1).

NP radius = 8.45 nm

$\Rightarrow$ NP core volume = 2530 nm^3

UiO-66 density[11] ρ = 1.24 g cm^{-3} $\equiv$ 1.24 $\times$ 10^{-21} g nm^{-3}

$\therefore$ NP core mass = 3.1 $\times$ 10^{-18} g ($\equiv$ 1.9 $\times$ 10^{6} g mol^{-1})

Hydrazone **1** molar mass = 285 g mol^{-1}

Mass concentration of **1** = 0.34 μmol mg^{-1} (Section S8)

$\Rightarrow$ 9.7 $\times$ 10^{-5} g of **1** per mg sample

$\Rightarrow$ 9.0 $\times$ 10^{-4} g of UiO-66 per mg sample

$\equiv$ 4.7 $\times$ 10^{-10} mol of UiO-66 NPs per mg sample

$\therefore$ Copies of **1** per NP = (0.34 $\times$ 10^{-6} / 4.7 $\times$ 10^{-10}) = 720

UiO-66 unit cell size = 2.07 nm

$\Rightarrow$ surface area of unit cell face = 4.14 nm^2

Unit cell face contains 2 clusters (1$\times$ centre + 4$\times$ ¼ at each corner)

$\therefore$ Surface area per cluster = 2.07 nm^2

NP core surface area = 897 nm^2

$\Rightarrow$ Number of clusters on NP surface $\approx$ 433

$\therefore$ Copies **1** per cluster = 720 / 433 = 1.7

Post-exchange functionalisation density

On repeated back-and-forth dynamic covalent exchange, functionalisation density falls on the first exchange process to a plateau value of 0.23 μmol mg–1 (Table S13, Section 15.3).

$\Rightarrow$ 6.6 $\times$ 10^{-5} g of **1** per mg sample

$\Rightarrow$ 9.3 $\times$ 10^{-4} g of UiO-66 per mg sample

$\equiv$ 4.9 $\times$ 10^{-10} mol of UiO-66 NPs per mg sample

$\therefore$ Copies of **1** per NP = (0.23 $\times$ 10^{-6} / 4.9 $\times$ 10^{-10}) = 470

$\therefore$ Copies **1** per cluster = 470 / 433 = 1.1

Normalisation of BET surface area

For **UiO-66-elec-4F**

Mass % of surface active UiO-66 = $(9.0 \times 10^{-4} / 1 \times 10^{-3}) \times 100$

$= 90\%$

$\Rightarrow$ BET surface area normalized to UiO-66 content = 998 / 0.9

$= \underline{1109\ m^2\ g^{-1}}$

10. Characterisation of UiO-66-nuc-4F

10.1 Structural characterisation of UiO-66-nuc-4F

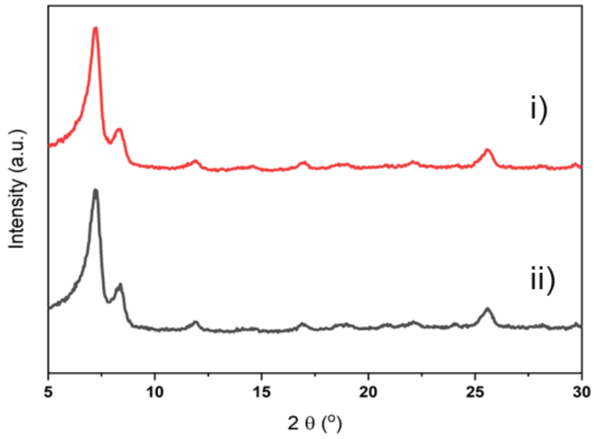

Figure S17 PXRD patterns of i) **UiO-66-nuc-4F** and (ii) pristine UiO-66.

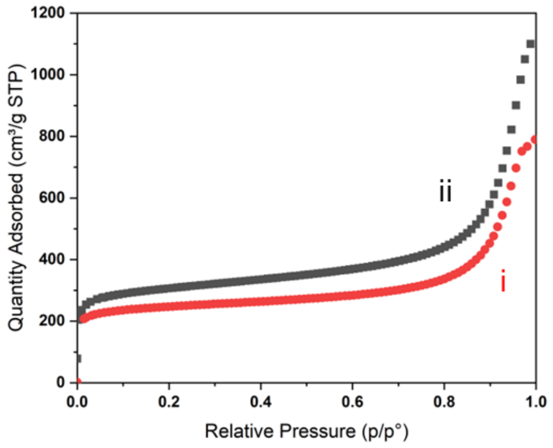

Figure S18 N_2 adsorption (77 K) isotherms of i) **UiO-66-nuc-4F** (red) and ii) pristine UiO-66 (black). BET surface areas of 950 m^2 g^{-1} for **UiO-66-nuc-4F** and 1170 m^2 g^{-1} for pristine UiO-66.

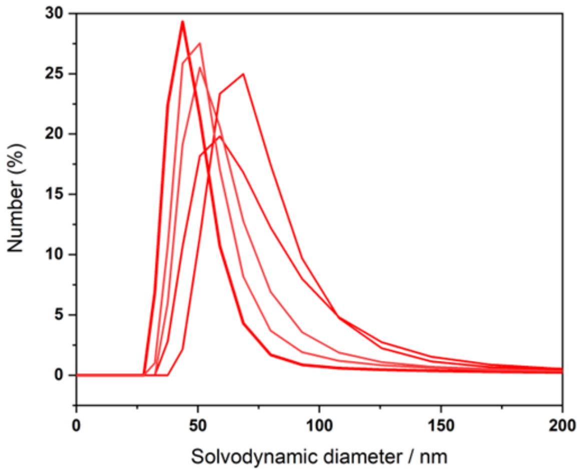

Figure S19 DLS measurements of **UiO-66-nuc-4F** with respect to number (%) <d_{EtOH}> = 64 (10) nm.

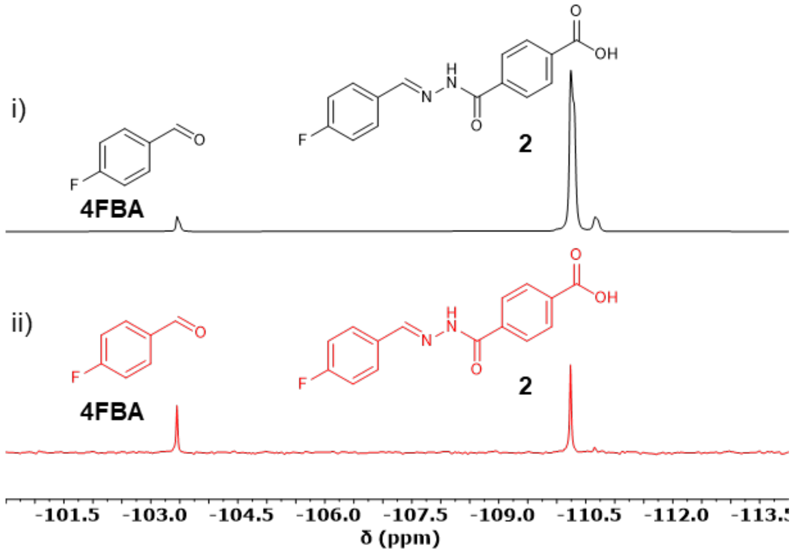

Figure S20 Acid digestion NMR spectra in which ~3 mg of MOF or molecule, is digested with conc. H_2SO_4 (30 μL) in DMSO-d6. ^{19}F NMR spectra (376.7 MHz, DMSO-d_6), (i) authentic hydrazone **2**, (ii) **UiO-66-nuc-4F**.

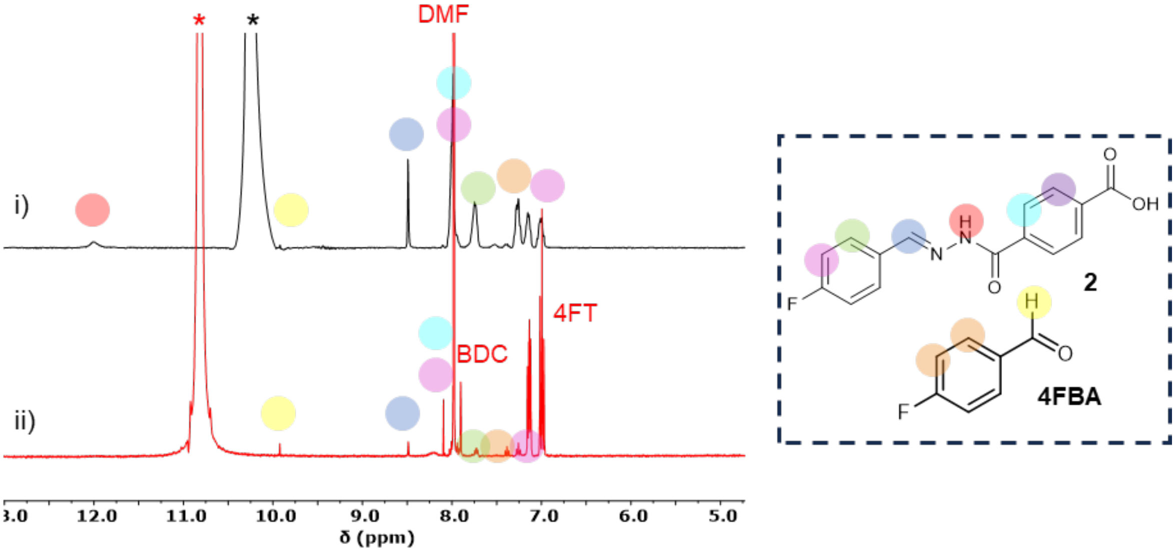

Figure S21 Acid digestion NMR spectra in which ~3 mg of MOF or molecule, is digested with conc. H_2SO_4 (30 μL) in DMSO-d_6. ^1H NMR spectra (400.3 MHz, DMSO-d_6), (i) authentic hydrazone **2**, (ii) **UiO-66-nuc-4F**. Spectra are labelled with protons corresponding to **2** and digestion product **4FBA**. 1,4-Benzenedicarboxylic acid (BDC) linker from the MOF, and *N*,*N*-dimethylformamide (DMF) solvent used are also labelled. * = H_2SO_4.

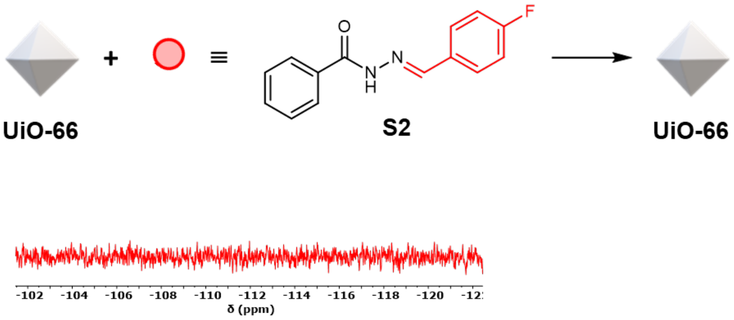

Figure S22 Control modification of UiO-66 using analogue hydrazone **S2** lacking a carboxylate binding site. Conditions: UiO-66 (0.03 mmol $C_{48}H_{28}O_{32}Zr_6$), **S2** (0.03 mmol) in DMF (20 mL), RT, 7 d. Acid digestion ^{19}F NMR spectrum (376.7 MHz, DMSO-d_6). ~3 mg of MOF digested with conc. H_2SO_4 (30 μL) in DMSO-d_6.

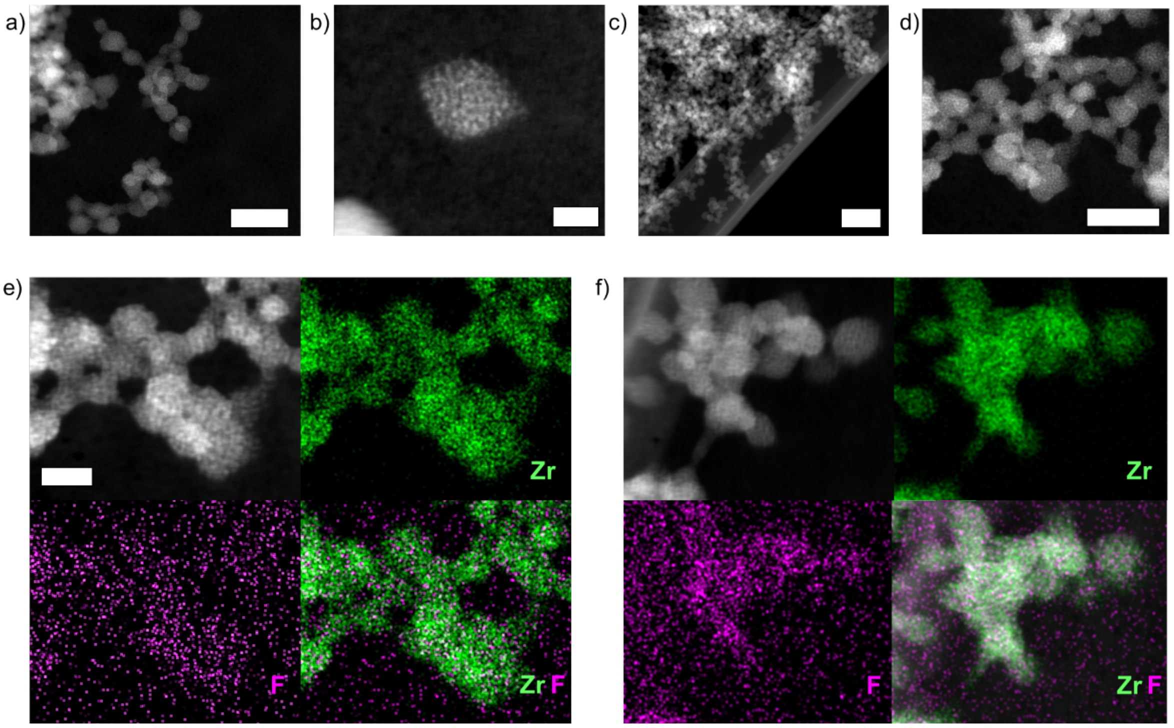

Figure S23 HAADF-STEM images of **UiO-66-nuc-4F**; scale bars: a) 50 nm, b) 10 nm, c) 100 nm, and d) 50 nm. e)-f) STEM-EDX images of **UiO-66-nuc-4F**; Scale bars = 20 nm green= Zr atoms, pink = F atoms.

10.2 Quantitative assessment of UiO-66-nuc-4F surface functionalisation

Acid digestion ^{19}F NMR spectroscopy

Using the procedure outlined in Section 7.2.

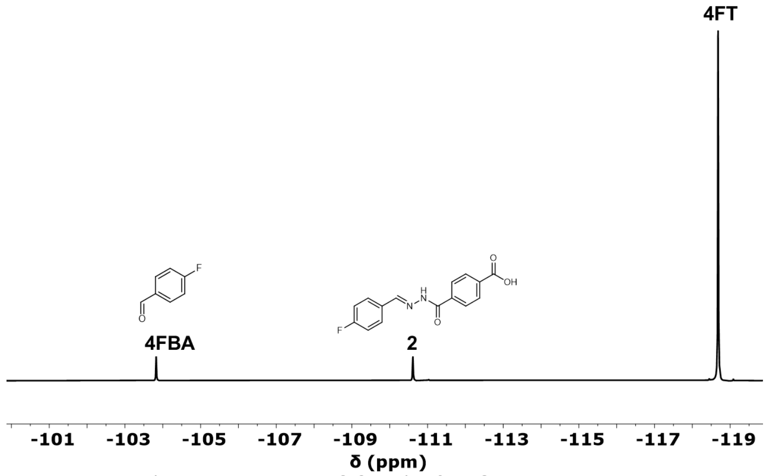

Figure S24 ^{19}F qNMR spectrum (376.7 MHz, DMSO-d_6) of **UiO-66-nuc-4F** acid digestion (H_2SO_4).

Table S5 Quantitative analysis of the acid digestion ^{19}F NMR spectrum of **UiO-66-nuc-4F**.

[**4FBA**] / mM	[**2**] / mM	[**4FBA**] + [**2**] / mM	Total quantity **2** initially present / μmol	Mass concentration **2** / μmol mg^{-1}
0.703	0.844	1.547	0.898	0.301

TFA stripping ^{19}F NMR spectroscopy

Using the procedure outlined in Section 7.3.

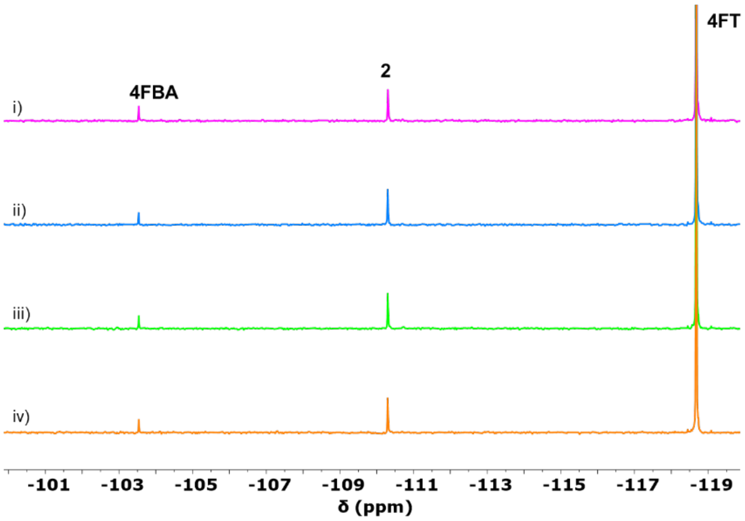

Figure S25 ^{19}F qNMR spectra (376.7 MHz, 10% *v*/*v* D_2O/DMF) of **UiO-66-nuc-4F**. TFA (100 mM in 10% *v*/*v* D_2O/DMF) after i) < 1 h, ii) 24 h, iii) 48 h and iv) 7 d. Referenced to IS = 4FT (10 mM).

Table S6 Quantitative analysis of the TFA stripping ^{19}F qNMR Spectra of **UiO-66-nuc-4F.**

Time	[**4FBA**] / mM	[**2**] / mM	[**4FBA**] + [**2**] / mM	Total quantity **2** present / μmol	Mass concentration **2** / μmol per mg
< 1 h	0.242	0.767	1.009	0.605	0.320
24 h	0.267	0.816	1.083	0.650	0.343
48 h	0.252	0.865	1.117	0.670	0.355
7 d	0.308	0.778	1.085	0.651	0.345

[a] The first spectrum recorded immediately following sample preparation after a wait time dependent on spectrometer availability.

CA stripping ^{19}F NMR spectroscopy

Using the procedure outlined in Section 7.4.

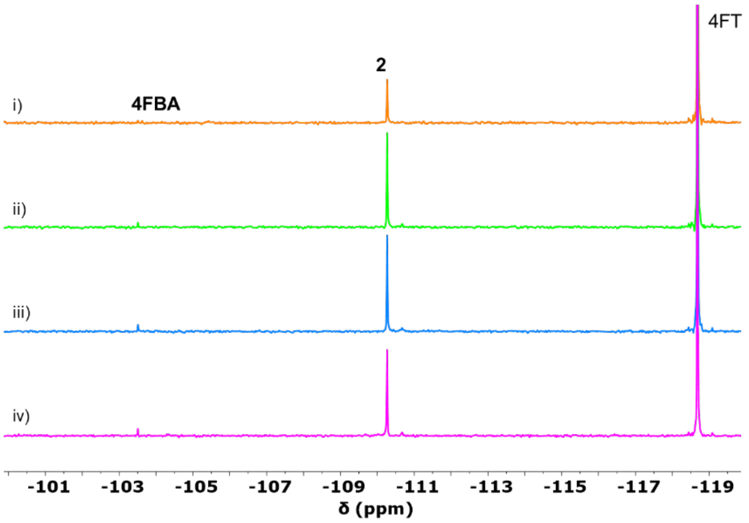

Figure S26 ^{19}F qNMR spectra (376.7 MHz, D_2O) of **UiO-66-nuc-4F** CA (100 mM in 10% v/v D_2O/DMF) stripping over time after i) < 1 h, ii) 24 h, iii) 48 h and iv) 8 d. Referenced to IS = 4FT (10 mM).

Table S7 Quantitative analysis of the CA stripping ^{19}F qNMR Spectra of **UiO-66-nuc-4F.**

Time	[**4FBA**] / mM	[**2**] / mM	[**4FBA**] + [**2**] / mM	Total quantity **2** present / μmol	Mass concentration **1** / μmol per mg
< 1 h [a]	0	0.769	0.769	0.462	0.135
24 h	0.048	1.593	1.640	0.984	0.288
48 h	0.106	1.642	1.748	1.049	0.307
8 d	0.0905	1.726	1.817	1.090	0.319

[a] The first spectrum recorded immediately following sample preparation after a wait time dependant on spectrometer availability.

11. Stability tests of UiO-66-elec-4F under dynamic covalent exchange conditions

11.1 Stability test procedures

The stability of the MOF framework, particle morphology and surface functionalisation was assessed in the presence of a variety of acid or nucleophile catalysts (Scheme S3) at a range of concentrations and environmental conditions. Concentrations refer to overall concentration in the final reaction mixture.

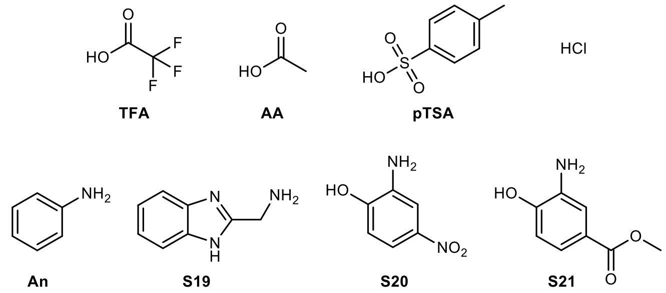

Scheme S3 Brønsted acid and nucelophilic catalysts that were investigated in stability tests with **UiO-66-elec-4F**. All catalysts were sourced commercially.

Trifluoroacetic acid (TFA)

UiO-66-elec-4F (20–25 mg) was dispersed in DMF (8 mL) and TFA was added (0–20 mM overall). The suspension was stirred for 1–7 days at RT or at 35 °C. Then the MOF was collected via centrifugation (13000 rpm, 10 min). The supernatant was separated and solvent removed under reduced pressure for analysis by NMR spectroscopy. The recovered MOF was then washed 3x DMF, 2x MeOH (collecting the solid by centrifugation at 13000 rpm, 10 min each time). The MOF was then dried in an oven at 60 °C.

Acetic acid (AA)

UiO-66-elec-4F (25 mg) was dispersed in DMF (8 mL) and AA was added (20–40 mM). The suspension was stirred for 1–7 days at RT or at 35 °C. Then the MOF was collected via centrifugation (13000 rpm, 10 min). The supernatant was separated and solvent removed under reduced pressure for analysis by NMR spectroscopy. The recovered MOF was then washed 3x DMF, 2x MeOH or EtOH (collecting the solid by centrifugation at 13000 rpm, 10 min each time). The MOF was then dried in an oven at 60 °C.

Hydrochloric acid and p-toluene sulfonic acid (pTSA)

UiO-66-elec-4F (20 mg) was dispersed in DMF (8 mL) and either HCl or *p*TSA (0.2 or 2 mM) were added. The suspension was stirred for 72 h at RT. Then the MOF was collected via centrifugation (14500 rpm, 10 min). The supernatant was separated and solvent removed under reduced pressure for analysis by NMR spectroscopy. The recovered MOF was then

washed 2x DMF, 3x EtOH (collecting the solid by centrifugation at 14500 rpm, 10 min each time). The MOF was then dried in an oven at 60 °C.

Aniline (An)

UiO-66-elec-4F (25 mg) was dispersed in DMF (8 mL) and aniline was added (20 mM). The suspension was stirred for 3 days at RT or at 35 °C. Then the MOF was collected via centrifugation (13000 rpm, 10 min). The supernatant was separated and solvent removed under reduced pressure for analysis by NMR spectroscopy. The recovered solid was then washed 1x DMF, 1x EtOH (collecting the solid by centrifugation at 14500 rpm, 10 min each time). The MOF was then dried in an oven at 60 °C.

Alternative nucleophilic catalysts

UiO-66-elec-4F (15 mg) was dispersed in DMF (6 mL) and one of catalysts **S31–S33** (20 mM) was added. The suspension was stirred for 7 days at RT. Then the MOF was collected via centrifugation (14500 rpm, 10 min). The supernatant separated and solvent removed under reduced pressure for analysis by NMR spectroscopy. The recovered soldi was then washed 2x DMF, 3x EtOH (collecting the solid by centrifugation at 14500 rpm, 10 min each time). The MOF was then dried in an oven at 60 °C.

11.2 Analysis of stability studies on UiO-66-elec-4F

To study the stability of the functionalized MOF NP under dynamic covalent exchange reaction conditions, the recovered MOF material was subjected to acid digestion (following the procedure outlined in Section 7.2) then ^{19}F NMR spectra were collected to observe whether hydrazone **1** was preserved and if any catalyst or degradation products had become associated with the NPs, either by surface binding or adsorption in the porous framework. Additionally, a ^{19}F NMR spectrum of the test reaction supernatant was collected to reveal any desorption of hydrazone **1** from the MOF.

Trifluoroacetic acid (TFA) catalyst

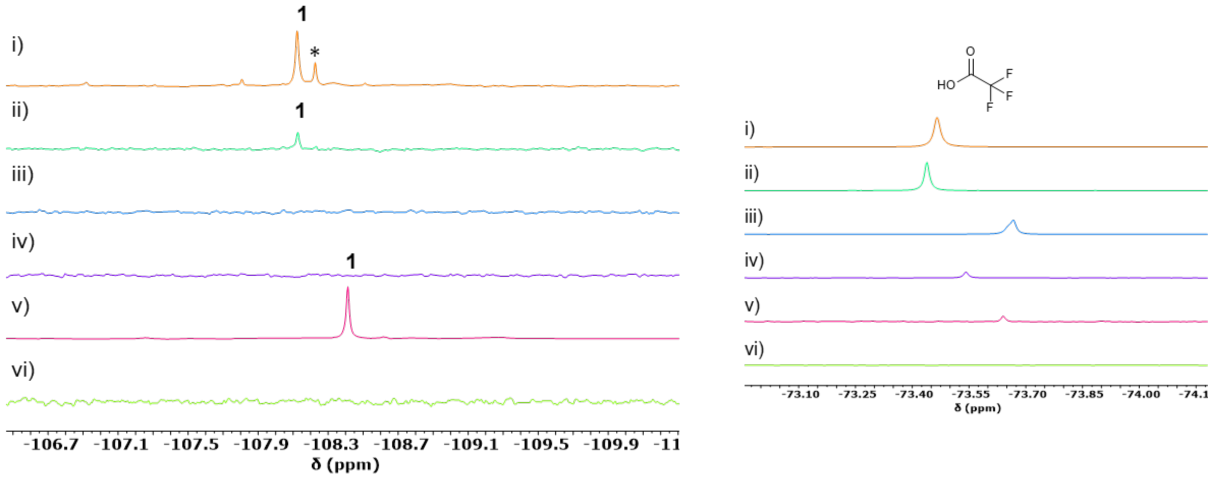

Figure S27 ^{19}F NMR spectra (DMSO-d_6) of supernatants collected after dispersing **UiO-66-elec-4F** in DMF with i) 20 mM TFA, 1 d, RT (376.7 MHz); ii) 2 mM TFA, 1 d, RT (470.4 MHz); iii) 0.2 mM TFA, RT, 1 d (376.7 MHz); iv) 0.2 mM TFA, RT, 7 d (376.7 MHz); v) 0.2 mM TFA, 35 °C, 3 d (470.4 MHz); vi) no TFA, RT, 1 d (376.7 MHz). Left: δ –105.0 to –110.0 ppm. Right: magnification of region δ –74.0 to –76.5 ppm showing resonance corresponding to TFA. * tentatively assigned to amide rotamer of **1** stabilized by interaction with TFA.

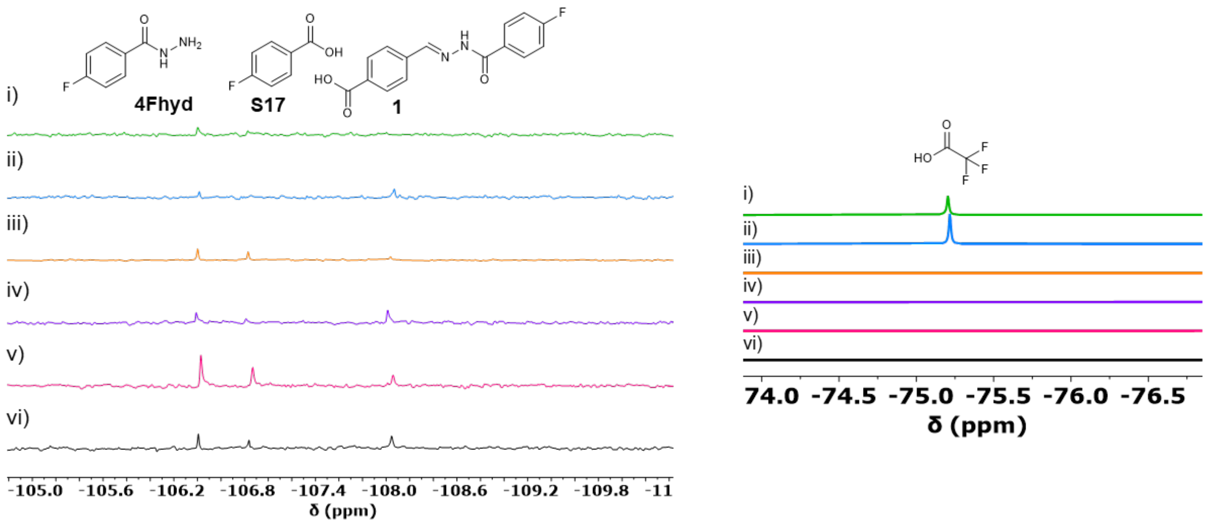

Figure S28 ^{19}F NMR spectra (376.7 MHz, DMSO-d_6) of acid digested **UiO-66-elec-4F** (~ 3 mg with conc. H_2SO_4) following incubation under test dynamic covalent exchange reaction conditions, followed by multiple rounds of washing. Conditions: i) 20 mM TFA, 1 d, RT; ii) 2 mM TFA, 1 d, RT; iii) 0.2 mM TFA, RT, 1 d; iv) 0.2 mM TFA, RT, 7 d; v) 0.2 mM TFA, 35 °C, 3 d; vi) no TFA, RT, 1 d. Left: δ –105.0 to –110.0 ppm. Right:

magnification of region δ –74.0 to –76.5 ppm indicating conditions under which TFA was not removed by the work-up procedure, suggesting incorporation in the framework.

Acetic acid (AA) catalyst

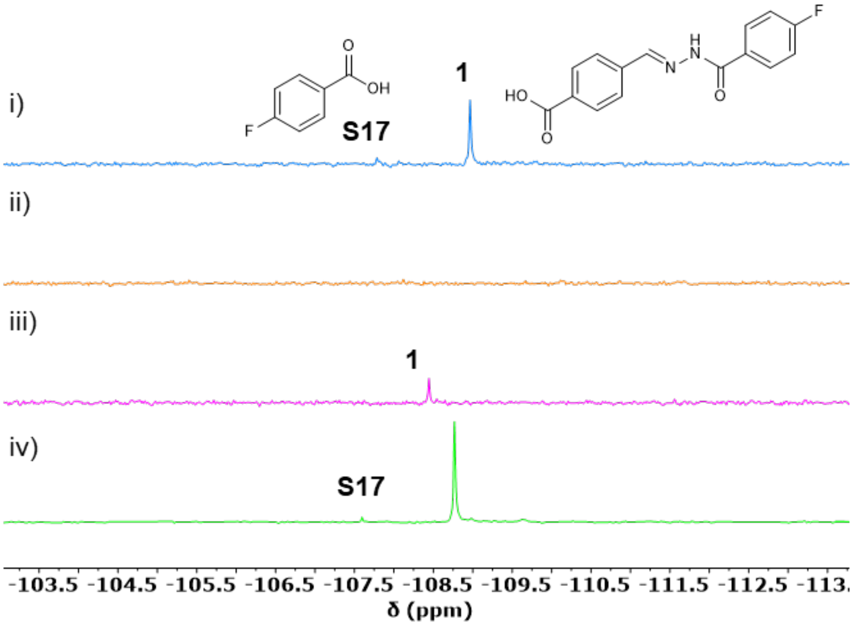

Figure S29 ^{19}F NMR spectra (376.7 MHz, DMSO-d_6) of supernatants collected after dispersing **UiO-66-elec-4F** in DMF with i) 20 mM AA, 1 d, RT; ii) 40 mM AA, 1 d, RT; iii) 20 mM AA, RT, 7 d; iv) 20 mM AA, 35 °C, 3 d.

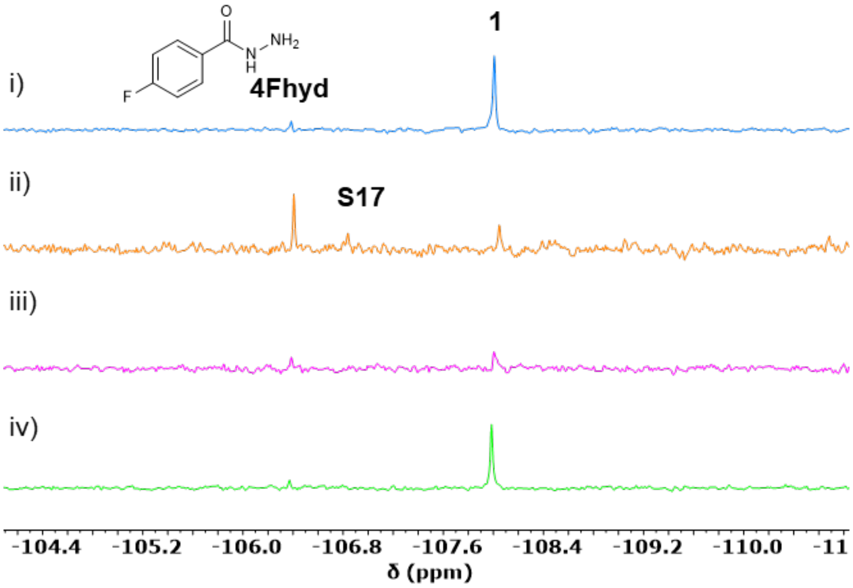

Figure S30 ^{19}F NMR spectra (DMSO-d_6) of acid digested **UiO-66-elec-4F** (~ 3 mg with conc. H_2SO_4) following incubation under test dynamic covalent exchange reaction conditions, followed by multiple rounds of washing. Conditions: i) 20 mM AA, 1 d, RT (376.7 MHz); ii) 40 mM AA, 1 d, RT (376.5 MHz); iii) 20 mM AA, RT, 7 d (376.7 MHz); iv) 20 mM AA, 35 °C, 3 d (376.7 MHz).

Aniline (An) catalyst

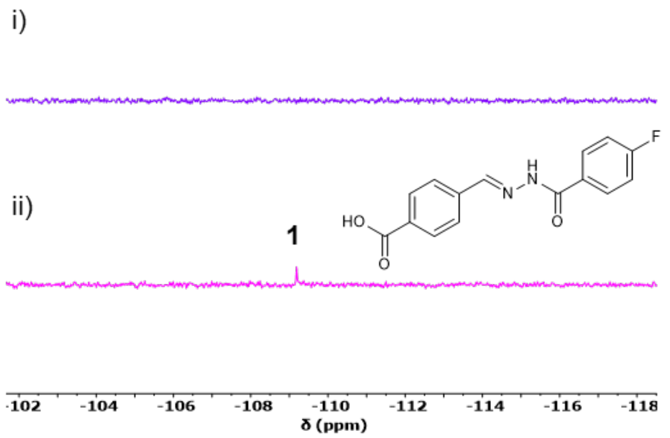

Figure S31 ^{19}F NMR spectra (376.7 MHz, DMSO-d_6) of supernatants collected after dispersing **UiO-66-elec-4F** in DMF with i) 20 mM An, 1 d, RT; ii) 20 mM An, 35 °C, 3 d.

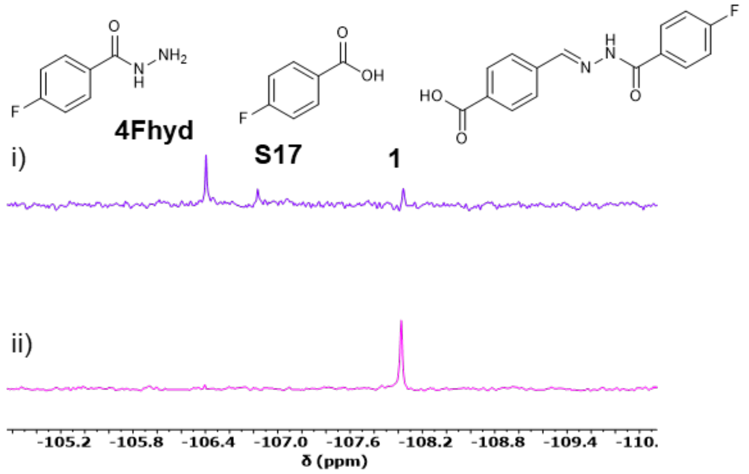

Figure S32 **[19]**F NMR spectra (376.7 MHz, DMSO-d_6) of acid digested **UiO-66-elec-4F** (~ 3 mg with conc. H_2SO_4) following incubation under test dynamic covalent exchange reaction conditions, followed by multiple rounds of washing. Conditions: i) 20 mM An, 1 d, RT; ii) 20 mM An, 35 °C, 3 d.

Hydrochloric acid and p-toluene sulfonic acid (pTSA) catalysts

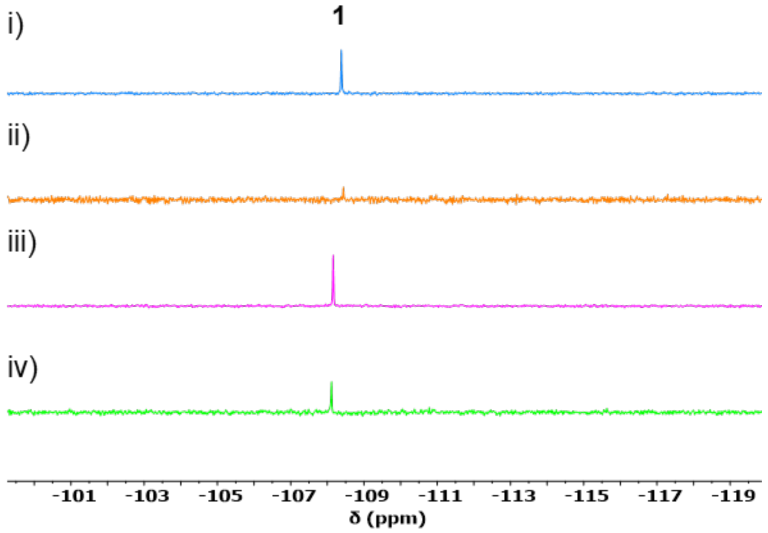

Figure S33 ^{19}F NMR spectra (DMSO-d_6) of supernatants collected after dispersing **UiO-66-elec-4F** in DMF with i) 2 mM *p*TSA, 3 d, RT (376.7 MHz); ii) 0.2 mM *p*TSA, 3 d, RT (470.4 MHz). ^{19}F NMR spectra (DMSO-

d_6) of supernatants collected after dispersing **UiO-66-elec-4F** in iii) 2 mM HCl, 3 d, RT (470.4 MHz); iv) 0.2 mM HCl, 3 d, RT (376.7 MHz).

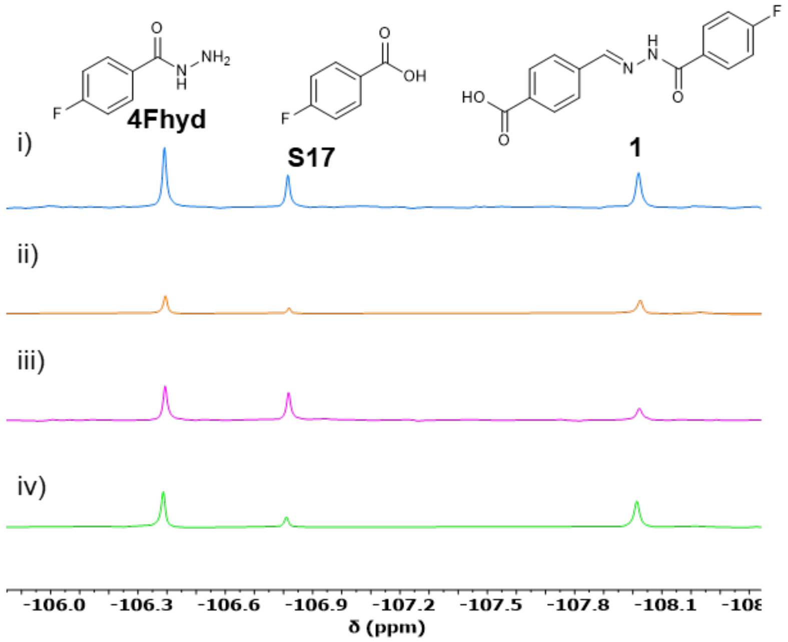

Figure S34 ^{19}F NMR spectra (376.7 MHz, DMSO-d_6) of the acid digested **UiO-66-elec-4F** (~ 3 mg with conc. H_2SO_4) following incubation under test dynamic covalent exchange reaction conditions, followed by multiple rounds of washing. Conditions: i) 2 mM pTSA, 3 d, RT; ii) 0.2 mM pTSA, 3 d, RT; iii) 2 mM HCl,3 d, RT; iv) 0.2 mM HCl, 3 d, RT.

Nucleophilic catalysts

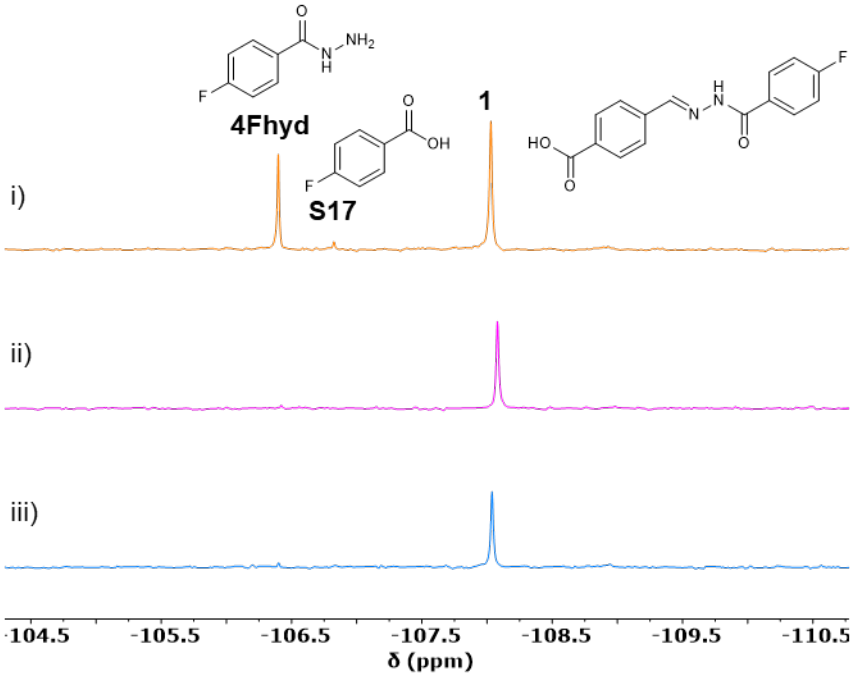

Figure S35 ^{19}F NMR spectra (376.7 MHz, DMSO-d_6) of acid digested **UiO-66-elec-4F** (~ 3 mg with conc. H_2SO_4) following incubation under test dynamic covalent exchange reaction conditions, followed by multiple rounds of washing. Conditions: i) 20 mM **S19**, 7 d, RT; ii) 20 mM **S20**, 7 d, RT; iii) 20 mM **S21**, 7 d, RT.

12. Model exchange reactions on electrophilic molecular substrate (S3)

12.1 Exchange with o-(2,3,4,5,6-pentafluorobenzyl)hydroxylamine hydrochloride (PFBHA)

The exchange of model hydrazone **S3** with an equimolar quantity of nucleophilic modifier **PFBHA** (Scheme S4) was investigated in the presence of Brønsted acid and nucleophilic catalysts. Reactions were performed in either 10% or 20% *v*/*v* D_2O in DMF. Reaction progress was monitored by ^{19}F qNMR spectroscopy, monitoring the concentration of **4Fhyd** released into solution over time (Figure S36).

General Method

Stock solutions of **S3** (50 mM), **PFBHA** (50 mM) and catalysts (200 mM) were made up in 9:1 DMF/D_2O containing 10 mM of 4FT. Reactions were then carried out in an NMR tube by mixing the stock solutions (final concentration of **S3** and **PFBHA** both 5 mM) and recording quantitative ^{19}F NMR spectra (d_1 = 25 s) at regular time intervals.

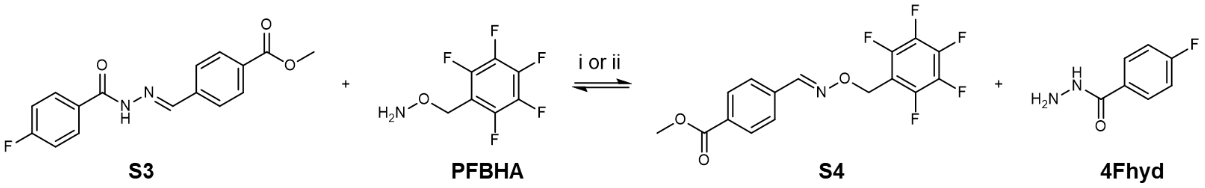

Scheme S4 Exchange of model hydrazone **S3** with **PFBHA**. Reaction conditions: (i) **S3** (5 mM), **PFBHA** (5 mM), choice of catalyst: 0.2 mM pTSA, 0.2 mM HCl, 20 mM An, None, 0.2 mM TFA, 2 mM HCl, 2 mM pTSA, 20 mM **S19, S20** or **S21** in 10% *v*/*v* D_2O/DMF, RT. (ii) **S3** (5 mM), **PFBHA** (5 mM), choice of catalyst: None, 0.2 mM TFA, 20 mM AA or 20 mM An in 20% D_2O/DMF solution, RT.

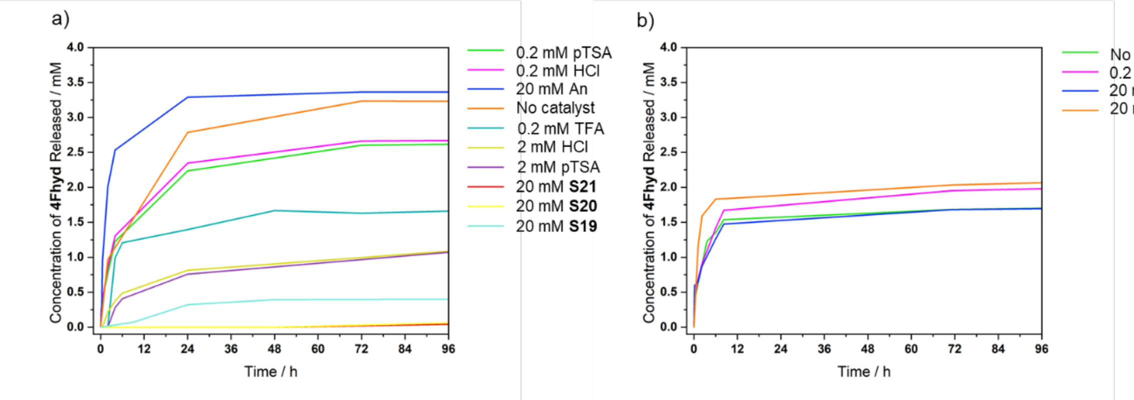

Figure S36 Plots of the concentration of **4Fhyd** released into solution over time in the molecular model exchange of **S3** with **PFBHA**, calculated from ^{19}F qNMR data. a) Exchanges in 10% *v*/*v* D_2O/DMF; b) exchanges in 20% *v*/*v* D_2O/DMF.

12.2 Exchange with 2-fluorobenzoic hydrazide (2Fhyd)

The exchange of model hydrazone **S3** with an equimolar quantity of nucleophilic modifier **2Fhyd** (Scheme S5) was investigated in the presence of Brønsted acid and nucleophilic catalysts. Reactions were performed in 10% *v*/*v* D_2O in DMF. Reaction progress was monitored by ^{19}F qNMR spectroscopy, monitoring the concentration of **4Fhyd** released into solution over time (Figure S37).

General Method

Stock solutions of **S3** (50 mM), **2Fhyd** (50 mM) and catalysts (200 mM) were made up in 10% *v*/*v* D_2O/DMF containing 10 mM of 4FT. Reactions were then carried out in an NMR tube by mixing the stock solutions (final concentration of **S3** and **2Fhyd** both 5 mM) and recording quantitative ^{19}F NMR spectra (d_1 = 25 s) at regular time intervals.

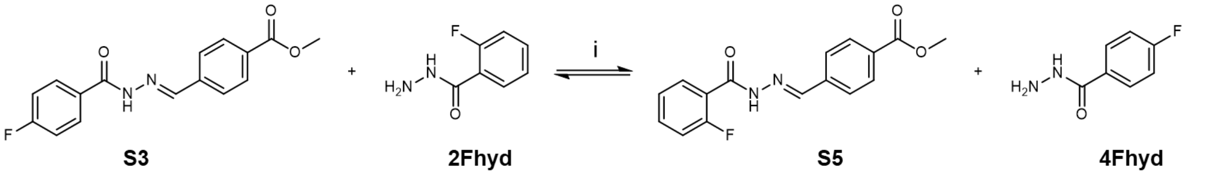

Scheme S5 Exchange of model hydrazone **S3** with **2Fhyd**. Reaction conditions: (i) **S3** (5 mM), **2Fhyd** (5 mM), choice of catalyst; None, 20 mM An, 2 mM HCl, 2 mM *p*TSA, 0.2 mM TFA, 0.2 mM *p*TSA, or 0.2 mM HCl, in 10% v/v D2O/DMF, RT, 14–21 d.

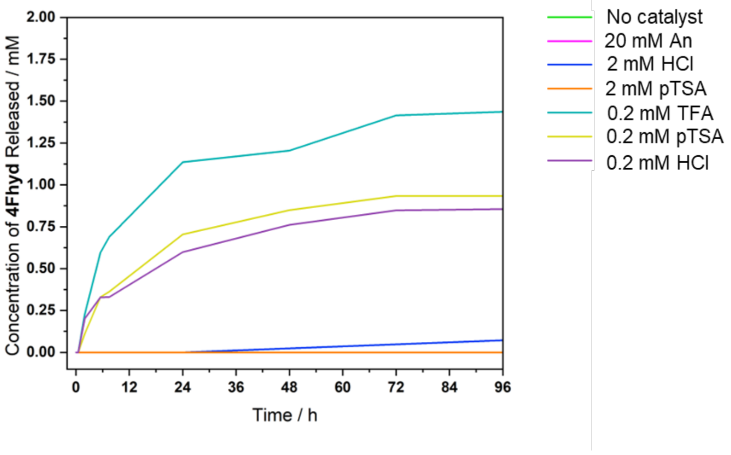

Figure S37 Plot of the concentration of hydrazide **4Fhyd** released into solution over time in the molecular model exchange of **S3** with **2Fhyd**, calculated from ^{19}F qNMR data.

13. Synthesis and characterisation of reference electrophilic MOF NPs

13.1 Synthesis of UiO-66 functionalized with oxime 3

UiO-66 was functionalized with **3** to form **UiO-66-elec-oxime** using the procedure described in Section 3.

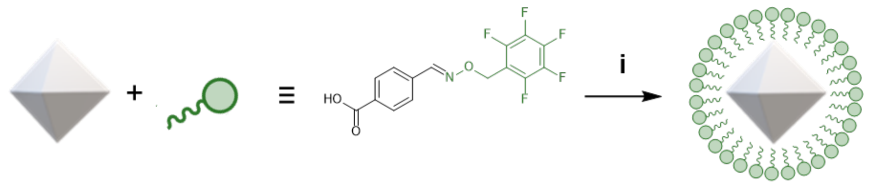

Scheme S6 Post-synthetic functionalisation of UiO-66 with oxime **3** via coordination of the carboxylate to Zr nodes on the surface to form **UiO-66-elec-oxime.** Conditions: (i) UiO-66 (0.126 mmol $C_{48}H_{28}O_{32}Zr_6$), **3** (0.180 mmol), dispersed in EtOH/DMF (3:2 *v*/*v*, ~10 mg/mL of MOF) at RT for 7 d.

13.2 Characterisation of UiO-66 functionalized with oxime 3

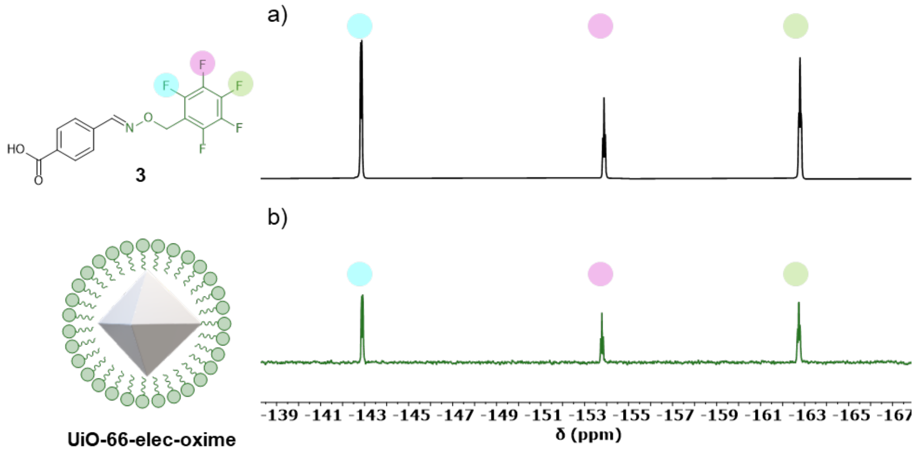

Figure S38 Acid digestion ^{19}F NMR spectra (376.7 MHz, DMSO-d_6) ~3 mg of MOF or molecule was digested with conc. H_2SO_4 (30 μL) in DMSO-d_6. a) Oxime **3**, b) **UiO-66-elec-oxime**.

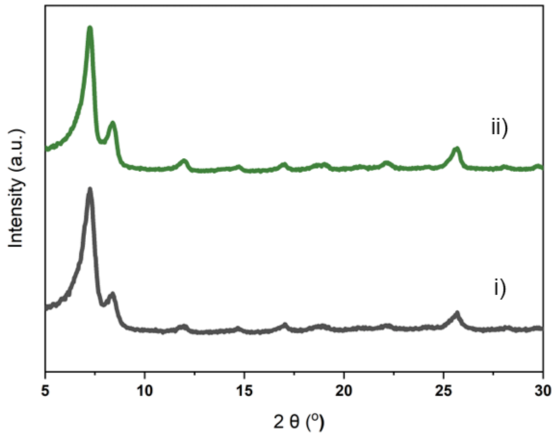

Figure S39 PXRD patterns of i) pristine UiO-66; ii) **UiO-66-elec-oxime.**

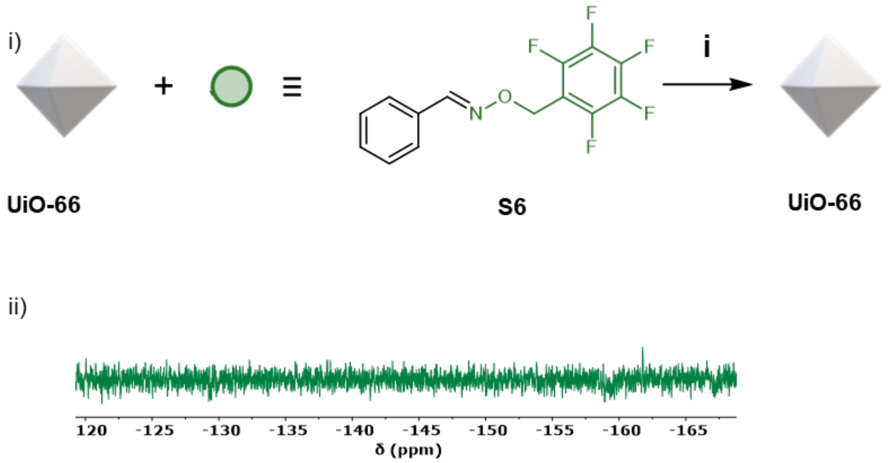

Figure S40 Control modification of UiO-66 using analogue oxime **S6** lacking a carboxylate binding site. Reaction conditions: i) UiO-66 (0.0301 mmol) dispersed in EtOH/DMF (3:2 *v*/*v*, ~10 mg/mL) at RT for 7 d, **S6** (0.0402 mmol). Acid digestion ^{19}F NMR spectrum (376.7 MHz, DMSO-d_6). ~3 mg of MOF digested with conc. H_2SO_4 (30 µL) in DMSO-d_6.

13.3 Synthesis of UiO-66 functionalized with hydrazone 4

UiO-66 was functionalized with **4** to form **UiO-66-elec-2Fhyd** using the procedure described in Section 3.

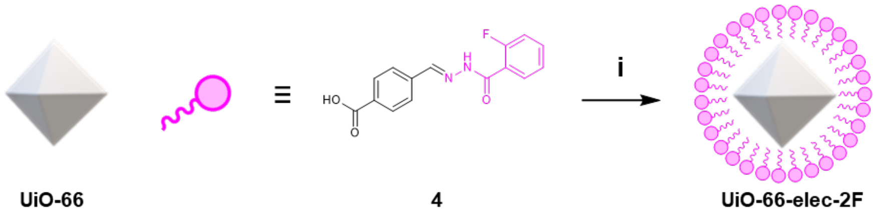

Scheme S7 Post-synthetic functionalization of UiO-66 with hydrazone **4** via coordination of the carboxylate to Zr nodes on the surface to form **UiO-66-elec-2F.** UiO-66 (0.03 mmol $C_{48}H_{28}O_{32}Zr_6$), **4** (0.042 mmol), dispersed in EtOH/DMF (3:2 *v*/*v*, ~10 mg/mL of MOF) at RT for 7 d.

13.4 Characterization of UiO-66 functionalized with hydrazone 4

Signal doubling is observed for hydrazone **5** due to two conformations related by rotation around the amide bond.[4] Under acid digestion conditions, hydrazone **2** decomposed into components **2FHyd** and 2-fluorobenzoic acid (**2FBA**). In quantitative experiments (Section 7) the concentrations of all three species were summed to estimate the total concentration of **2** present in the initial sample.

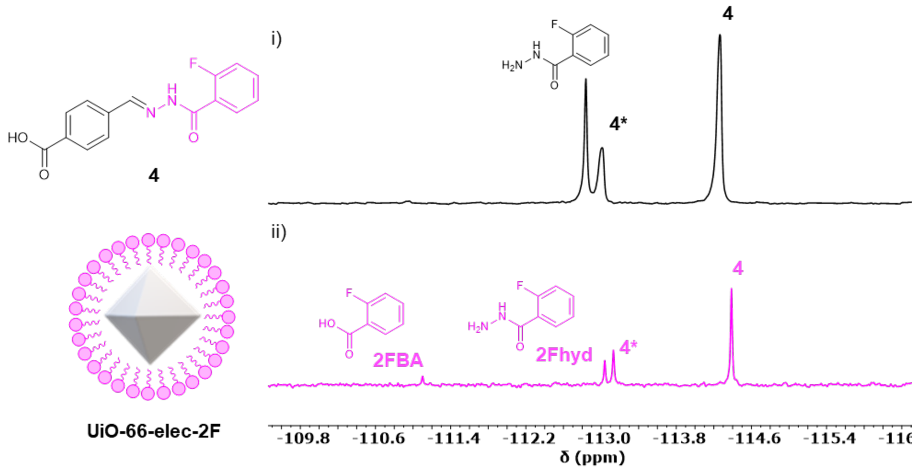

Figure S41 Acid digestion ^{19}F NMR spectra (376.7 MHz, DMSO-d_6) ~3 mg of MOF or molecule was digested with conc. H_2SO_4 (30 μL) in DMSO-d_6. a) Authentic hydrazone **4**, b) **UiO-66-elec-2F**.

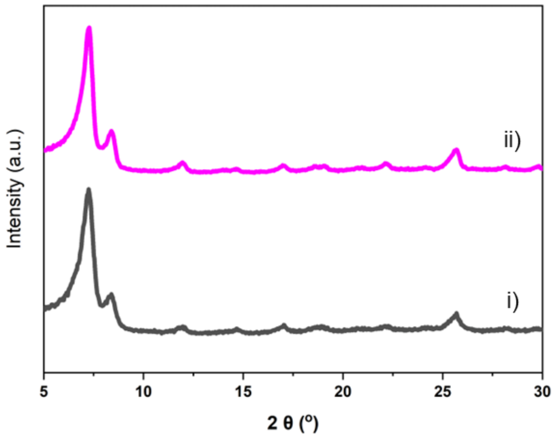

Figure S42 PXRD patterns of i) pristine UiO-66; ii) **UiO-66-elec-2F.**

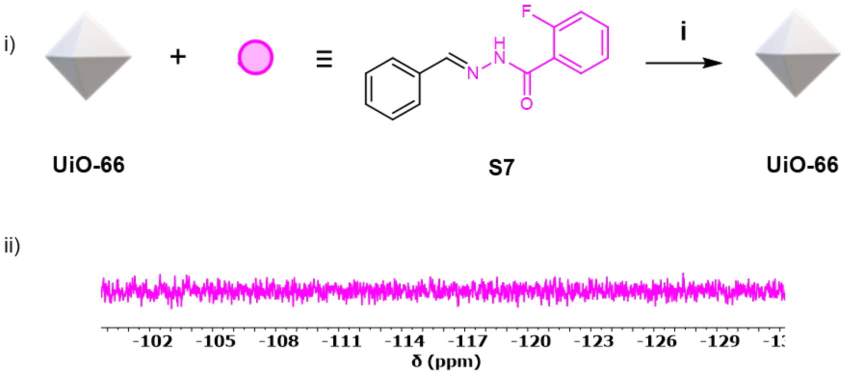

Figure S43 Control modification of UiO-66 using analogue hydrazone **S7** lacking a carboxylate binding site. Conditions: i) UiO-66 (0.0301 mmol $C_{48}H_{28}O_{32}Zr_6$) dispersed in EtOH/DMF (3:2 *v*/*v*, ~10 mg/mL) at RT for 7 d, **S7** (0.0402 mmol). Acid digestion ^{19}F NMR spectrum (376.7 MHz, DMSO-d_6). ~3 mg of MOF digested with conc. H_2SO_4 (30 μL) in DMSO-d_6.

14. Exchange between UiO-66-elec-4F and UiO-66-elec-oxime

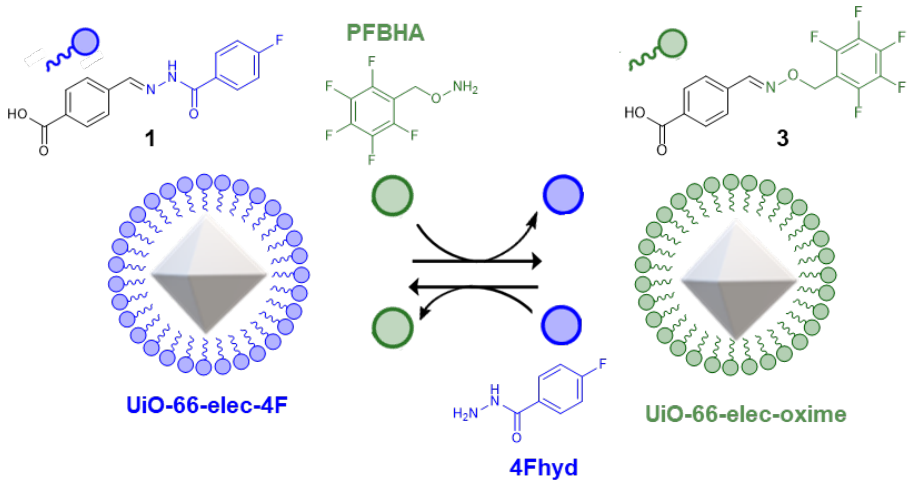

Scheme S8 Schematic representation of the exchange between **UiO-66-elec-4F** and **UiO-66-elec-oxime.**

14.1 Modification of UiO-66-elec-4F by exchange with PFBHA

Procedure

UiO-66-elec-4F (28.6 mg) was recovered from its ethanolic storage solution by evaporation of ethanol in a drying oven at 60 °C and atmospheric pressure.

A stock solution of 10 mM 4-fluorotoluene in 10% *v*/*v* D_2O/DMF was prepared and this was used as the solvent mixture for all further stock solutions.

Stock solutions of modifiers were first prepared volumetrically using the internal standard solvent mixture.

Recovered dried **UiO-66-elec-4F** was suspended in the solvent stock solution (1.6 mL) and dispersed via ultrasonication. The dispersion was then treated with **PFBHA** modifier stock solution (2 mL, 50 mM) and finally aniline (0.40 mL, 0.41 g, 4.4 mmol) was added, giving final concentrations: [**1**] = 2.5 mM, [**PFBHA**] = 25 mM and [aniline] = 1.1 M.

The solution was left to stir at room temperature for 48 h, and then the MOF separated from the reaction solution by centrifugation (4,500 rpm, 20 min).

The reaction solution was collected for analysis and the MOF was then purified by washing-dispersion cycles (1x DMF, 1x ethanol; recovery by centrifugation at 4,500 rpm for 20 min each time). The purified MOF was dried in an oven at 60 °C and atmospheric pressure and then analysed.

Analysis of exchange supernatant

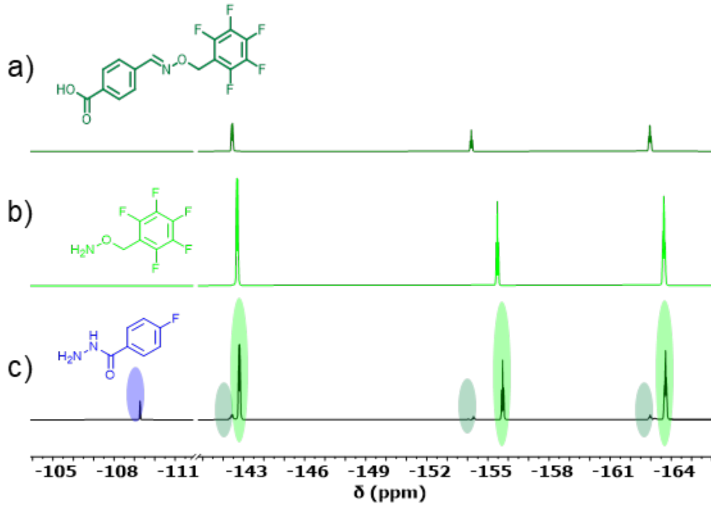

Figure S44 ^{19}F NMR spectra (376.7 MHz, 10% *v*/*v* D_2O/DMF) of a) **3**; b) **PFBHA**; c) the supernatant from exchange of **UiO-66-elec-4F** with **PFBHA**. Desorbed **3** (dark green) is observed in the supernatant along with expected exchanged **4Fhyd** (blue), and excess **PFBHA** (light green). No desorbed **1** is observed, consistent with quantitative dynamic covalent exchange to thermodynamically more stable **3**.

*Quantification of exchange product **UiO-66-elec-oxime-f***

Table S8 Quantification of exchange from **UiO-66-elec-4F** with **PFBHA**. Values derived from ^{19}F qNMR spectroscopy analysis of **UiO-66-elec-oxime-f** (following TFA stripping or acid digestion) and of reaction supernatant. All molar values quoted in μmol per milligram of solid. Percentages calculated relative to initial surface quantity of **1**.

Analysis method	Initial on-NP **1** / μmol mg^{-1}	Post-exchange on-NP **1** / μmol mg^{-1}	Post-exchange on-NP **3** / μmol mg^{-1}	Post-exchange on-NP **1**:**3**	Recovered on-NP material (%)	Desorbed functional units in supernatant(%) [a]
TFA stripping	0.34	0	0.13	0:100	38	76
Acid digestion	0.35	0	0.13	0:100	37	74

[a] Values from supernatant analysis. All desorbed material in supernatant was present as oxime **3**; hydrazone **1** was not detected. See Figure S44.

*PXRD analysis of **UiO-66-elec-oxime-f***

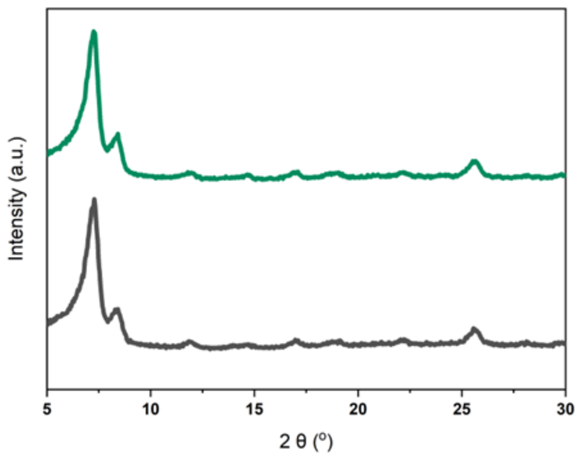

Figure S45 PXRD pattern of **UiO-66-elec-4F** (black, bottom) and exchange product **UiO-66-elec-oxime-f** (green, top).

14.2 Test reactions to explain desorption of functional units

To examine the causes for the desorption of surface species observed during the exchange reaction, the influence of the aniline catalyst and PFBHA modifier were probed in separate experiments.

*Effect of **PFBHA** modifier*

Dried **UiO-66-elec-4F** ([**1**] ≈ 1.5 mM) was dispersed in 10% D_2O/DMF (0.6 µL) containing 4FT internal standard (10 mM) and **PFBHA** modifier (5 mM, 3.3 mol equiv.) in the absence of any catalyst. Quantitative ^{19}F NMR spectra were recorded at intervals over a period of 7 days to monitor the loss of hydrazone **1** desorbed into solution (Figure S46, Table S9).

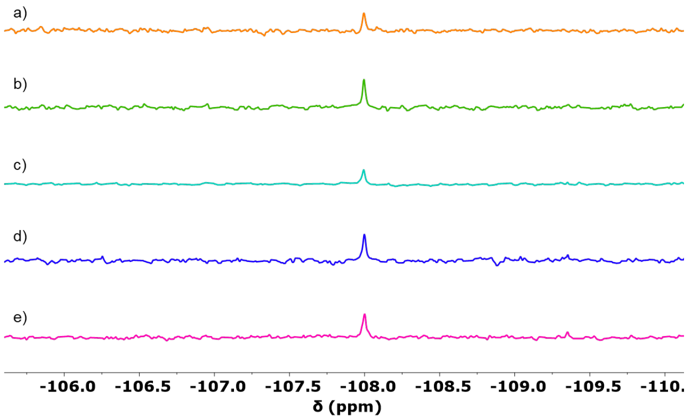

Figure S46 ^{19}F NMR spectra (376.7 MHz, 10% *v*/*v* D_2O/DMF) of **UiO-66-elec-4F** ([**1**] ≈ 1.5 mM) treated with **PFBHA** (5 mM, 3.3 mol equiv.). Spectra recorded after a) 0 h, b) 24 h, c) 48 h, d) 72 h, e) 168 h. Referenced to 4FT (10 mM).

Table S9 Concentration of hydrazone **1** released over time after treatment of **UiO-66-elec-4F** ([**1**] ≈ 1.5 mM) treated with **PFBHA** (5 mM, 3.3 mol equiv.) in 9:1 *v*/*v* DMF/D_2O.

Time / h	Hydrazone **1** / mM
0	0.18
24	0.38
48	0.36
72	0.40
168	0.40

After 7 days, the amount of **1** leached into solution plateaued at 0.09 µmol per milligram of **UiO-66-elec-4F**, or 26% of the initial surface-attached **1**.

After **UiO-66-elec-4F** was incubated with a larger excess of **PFBHA** modifier (25 mol equiv. relative to **1**), analysis of the supernatant revealed a significant quantity of oxime **3** in solution as well as desorbed **1** (Figure S47). Additional signals were also observed corresponding to products of side-reactions of **PFBHA**.

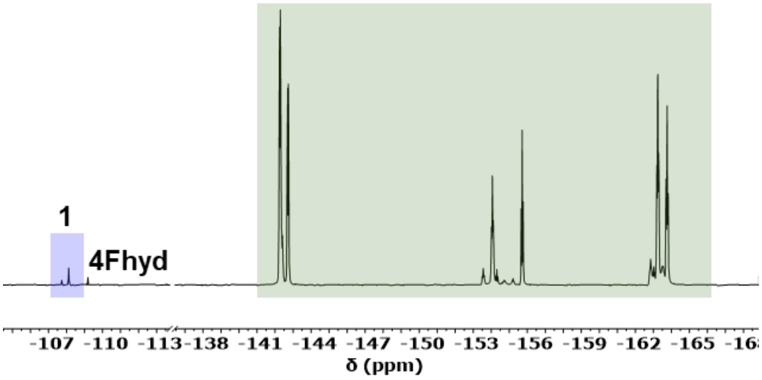

Figure S47 ^{19}F NMR spectrum (376.7 MHz, 10% *v*/*v* D_2O/DMF) of the supernatant of **UiO-66-elec-4F** ([**1**] ≈ 1.5 mM) treated with **PFBHA** (38 mM, 25 mol equiv.) showing desorbed hydrazone **1** (blue box), **4Fhyd** and multiple signals corresponding products of side reactions of **PFBHA** (green box).

Aniline catalyst

To extend the earlier investigation on the effects of excess aniline (Section 11), **UiO-66-elec-4F** was treated with a large excess of aniline (1 M) in the absence of modifier. The quantity of hydrazone **1** desorbed into the supernatant (0.03 µmol per milligram of **UiO-66-elec-4F**, ca. 30% of the initial surface-bound concentration, Figure S48) was not increased over experiments in the presence of lower concentrations of aniline (Section 11).

It could therefore be concluded that the nucleophilic **PFBHA** modifier is responsible for the observed desorption of carboxylate-coordinated surface functionality during dynamic covalent exchange.

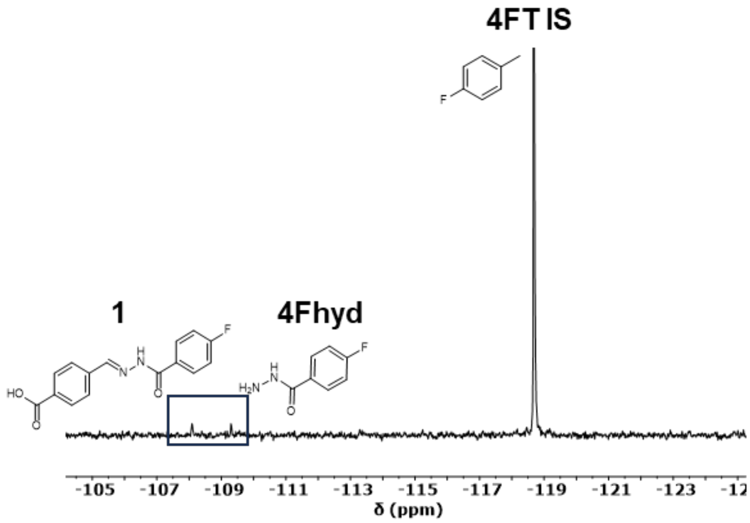

Figure S48 ^{19}F NMR spectrum (376.7 MHz, 10% v/v D_2O/DMF) of the supernatant solution after incubation with 1 M aniline.

14.3 Control exchange reactions

*Control 1: Interaction of pristine UiO-66 with **PFBHA***

To ensure that there was no confounding coordination or absorption of **PFBHA** on the MOF framework, unfunctionalized pristine UiO-66 was treated with **PFBHA** modifier under exchange conditions (Scheme S9).

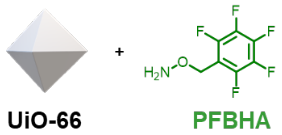

Scheme S9 Unfunctionalized UiO-66 was treated with PFBHA modifier.

Procedure

Pristine **UiO-66** (13.4 mg) powder was dispersed in 10% *v/v* D_2O/DMF (600 mL final volume) with 10 mM 4FT as internal standard along with **PFBHA** modifier (5 mM). After leaving to stir for 48 h at room temperature, the MOF was separated from the supernatant via centrifugation, then washed (1x DMF; recovery by centrifugation at 4500 rpm for 10 min at 5°C) then dried in an oven at 60 °C.

Analysis of the purified MOF by ^{19}F NMR spectroscopy after acid digestion revealed no **PFBHA** was coordinated to the MOF nor adsorbed into the pores, with only a signal corresponding to the IS detected (Figure S49a).

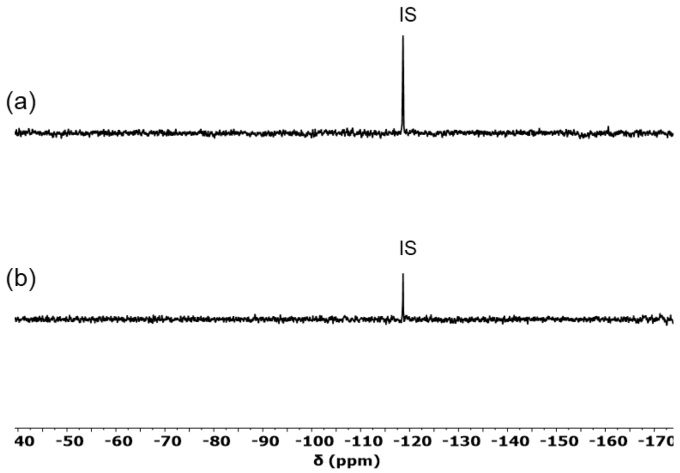

Figure S49 Analysis of control reactions 1 and 2. Acid digestion ^{19}F NMR spectra (376.7 MHz, DMSO-d_6) ~3 mg of MOF is digested with conc. H_2SO_4 (30 µL) in DMSO-d_6. a) Control 1 and b) Control 2. Referenced to IS, 4FT (10 mM)

Control 2: Interaction of pristine UiO-66 with exchange intermediates

To ensure no interaction between the hydrazone, oxime or exchange intermediates and the MOF, a bulk solution exchange reaction of model hydrazone **S2** with **PFBHA** was performed in the presence of pristine unfunctionalized UiO-66 (Scheme S10).

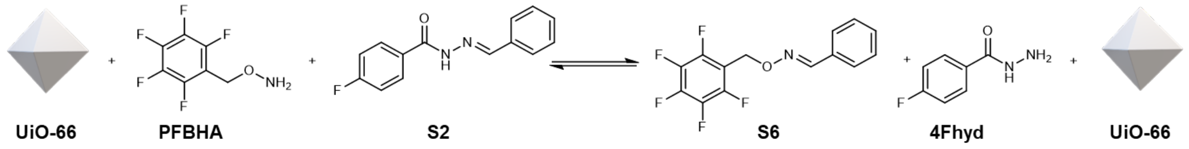

Scheme S10 Model compound exchange between **S2** and **S6** in the presence of pristine UiO-66**.**

Procedure

Pristine UiO-66 (13.4 mg, 8.06 µmol) powder was redispersed in 10% *v*/*v* D_2O/DMF (600 µL) along with **PFBHA** modifier (5 mM), **S2** (5 mM) and aniline (20 mM). After leaving to stir for 48 h at room temperature, the MOF was separated from the supernatant via centrifugation then washed (1x DMF; recovery by centrifugation at 4500 rpm for 10 min at 5°C) than dried in an oven at 60 °C.

Analysis of the purified MOF by ^{19}F NMR spectroscopy after acid digestion confirmed no uptake of exchange species to the surface or pores (Figure S49b).

14.4 Reverse exchange of UiO-66-elec-oxime with 4Fhyd

Procedure

Dried **UiO-66-elec-oxime-f** (0.93 mM in terms of **3**) was dispersed via sonication in 10% *v*/*v* D_2O/DMF (1.4 mL) containing **4Fhyd** (46.5 mM), acetic acid (20 mM), and 4FT IS (10 mM).

The solution was left to stir at 50 °C for 48 h, and then the MOF separated from reaction mixture by centrifugation (4,500 rpm, 20 min).

The reaction supernatant was collected for analysis and the solid was purified by washing-dispersion cycles (1x DMF, 1x ethanol; recovery by centrifugation at 4,500 rpm for 20 min

each time). The purified MOF was dried in an oven at 60 °C and atmospheric pressure and then analysed.

*Quantitative analysis of exchange product **UiO-66-elec-oxime-r***

Table S10 Quantification of exchange from **UiO-66-elec-oxime-f** with **4Fhyd**. Values derived from ^{19}F qNMR spectra of **UiO-66-elec-oxime-r** (values following acid digestion and TFA stripping were identical). All molar values quoted in μmol per milligram of solid. Percentages calculated relative to initial surface quantity of **3**.

Initial on-NP **3** / μmol mg^{-1}	Post-exchange on-NP **1** / μmol mg^{-1}	Post-exchange on-NP **4** / μmol mg^{-1}	On-NP conversion (%)	Recovered on-NP material (%)
0.13	0.04	0.07	31	85

15. Exchange between UiO-66-elec-4F and UiO-66-elec-2F

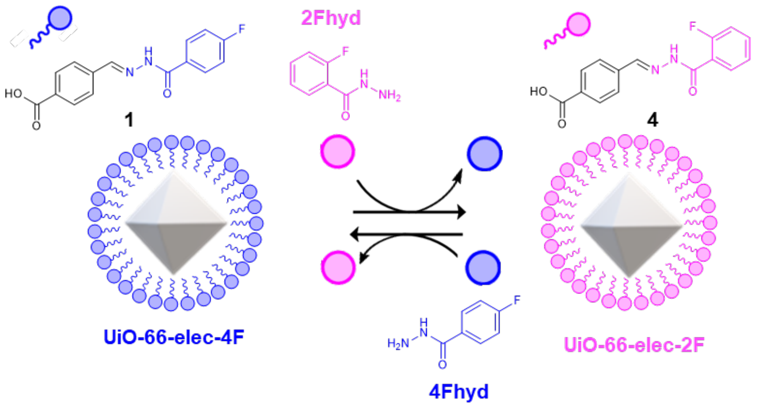

Scheme S11 Schematic representation of the exchange between **UiO-66-elec-4F** and **UiO-66-elec-2F.**

15.1 Modification of UiO-66-elec-4F by exchange with 2Fhyd

Procedure

UiO-66-elec-4F (28.6 mg) was recovered from its ethanolic storage solution by evaporation of ethanol in a drying oven at 60 °C and atmospheric pressure.

A stock solution of 10 mM 4-fluorotoluene in 10% *v*/*v* D_2O/DMF was prepared and this was used as the solvent mixture for all further stock solutions.

Stock solutions of all modifiers and catalysts were first prepared volumetrically using the internal standard solvent mixture.

Recovered dried **UiO-66-elec-4F** was suspended in the solvent stock solution (1.992 mL) and dispersed via ultrasonication. The dispersion was then treated with **2Fhyd** modifier stock solution (2 mL, 50 mM) and finally TFA (8 µL, 100 mM) was added, giving final concentrations: [**1**] = 2.5 mM, [**2Fhyd**] = 25 mM, [TFA] = 0.2 mM.

The solution was left to stir at 35 °C for 7 days, and then the MOF separated from the reaction mixture by centrifugation (4,500 rpm, 20 min).

The reaction solution was collected for analysis and the MOF was then purified by washing-dispersion cycles (1x DMF, 1x ethanol; recovery by centrifugation at 4,500 rpm for 20 min each time). The purified MOF was dried in an oven at 60 °C and atmospheric pressure and then analysed.

Analysis of exchange supernatant

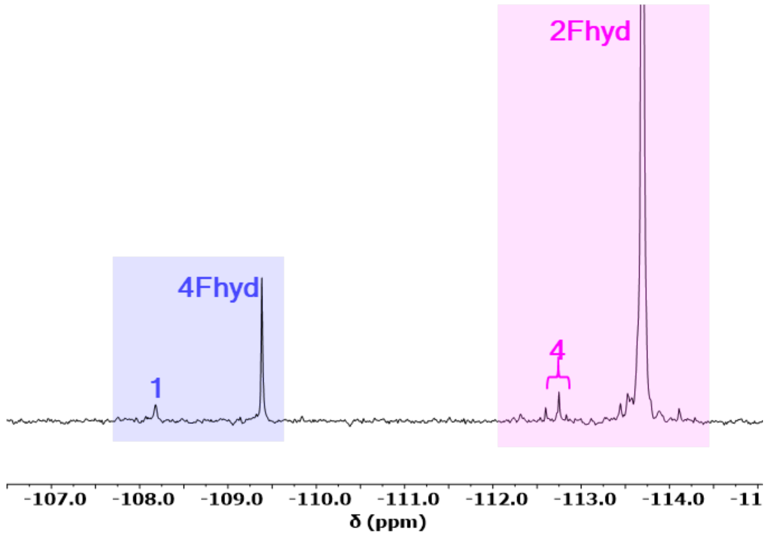

Figure S50 ^{19}F NMR spectrum (376.7 MHz, 10% *v*/*v* D_2O/DMF) of the supernatant after **2Fhyd** exchange with **UiO-66-elec-4F** to form **UiO-66-elec-2F-f**. Pink box highlights signals originating from the **2Fhyd** derivatives. Blue box highlights signals deriving from **4Fhyd** derivatives.

The quantity of hydrazone released into solution could only be reliably quantified for hydrazone **1** because the chemical shift region for the 2-fluorobenzylidine derivatives was obscured by excess 2-fluorobenzoyl hydrazide and side products (Figure S50). Approximately 0.04 μmol mg^{-1} of the initial surface species was released as **1**. Assuming the same conversion from **1** to **4** as observed from the on NP analysis (50%, Table S11), it can be estimated that there will be 0.04 μmol mg^{-1} **4** desorbed. The total amount of desorbed functional units would therefore be ~0.08 μmol mg^{-1}, corresponding to ~24% of the initial on-NP material.

*Quantification of exchange product **UiO-66-elec-2F-f***

Table S11 Quantification of exchange from **UiO-66-elec-4F** with **2Fhyd**. Values derived from ^{19}F qNMR spectra of **UiO-66-elec-2F-f** (following TFA stripping or acid digestion) and of reaction supernatant. All molar values quoted in μmol per milligram of solid. Percentages calculated relative to initial surface quantity of **1**.

Analysis method	Initial on-NP **1** / μmol mg^{-1}	Post-exchange on-NP **4** / μmol mg^{-1}	Post-exchange on-NP **1** / μmol mg^{-1}	Post-exchange on-NP **1**:**4**	Recovered on-NP material (%)	Desorbed **1** + **4** measured in supernatant (%) [a]
TFA stripping	0.34	0.10	0.10	50:50	59	24
Acid digestion	0.35	0.11	0.12	52:48	65	24

[a] Values from supernatant analysis (see Figure S50 and accompanying discussion above).

*PXRD analysis of **UiO-66-elec-2F-f***

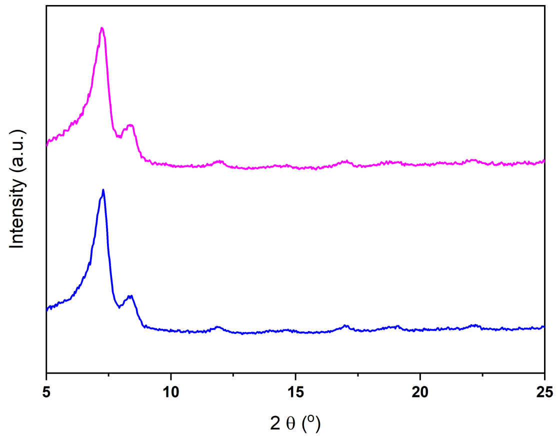

Figure S51 PXRD pattern of initial **UiO-66-elec-4F** (blue, bottom) and exchange product **UiO-66-elec-2F-f** (pink, top).

15.2 Reverse exchange of UiO-66-elec-2F-f with 4Fhyd

Procedure

Dried **UiO-66-elec-2F-f** was dispersed via sonication in 10% *v*/*v* D_2O/DMF (0.8 mL) containing **4Fhyd** (39 mM), TFA (0.2 mM), and 4FT IS (10 mM), giving a final concentration of 0.78 mM in terms of **4** (1.56 mM total hydrazone).

The solution was left to stir at 35 °C for 7 days, and then the MOF separated from reaction mixture by centrifugation (14,800 rpm, 10 min).

The reaction supernatant was collected for analysis and the solid was purified by washing-dispersion cycles (1x DMF, 1x ethanol; recovery by centrifugation at 14,800 rpm for 10 min each time). The purified MOF was dried in an oven at 60 °C and atmospheric pressure and then analysed.

Analysis of exchange supernatant

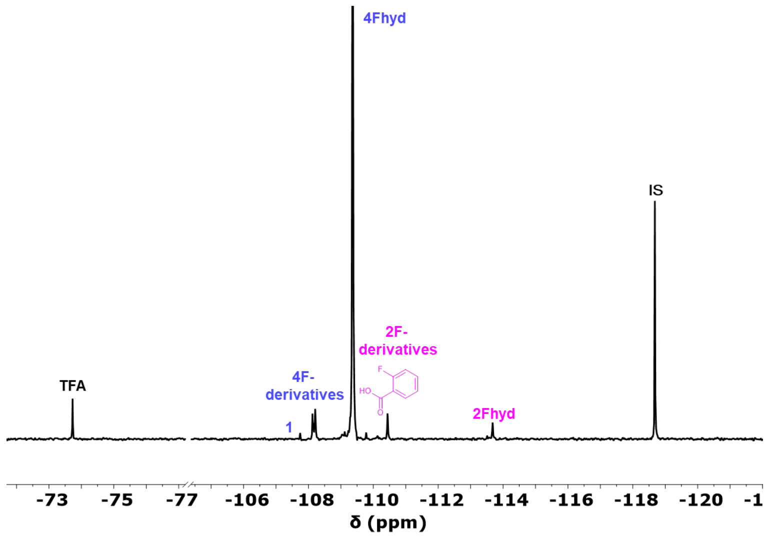

Figure S52 ^{19}F NMR spectrum (376.7 MHz, 10% *v*/*v* D_2O/DMF) of the supernatant after **4Fhyd** exchange with **UiO-66-elec-2F-f** to form **UiO-66-elec-2F-f-r**.

*Quantitative analysis of exchange product **UiO-66-elec-2F-r***

Table S12 Quantification of exchange from **UiO-66-elec-2F-f** with **4Fhyd**.Values derived from ^{19}F qNMR spectra of **UiO-66-elec-2F-r** following TFA stripping or acid digestion. All molar values quoted in μmol per milligram of solid. Conversion calculated relative to initial surface quantity of **4**; recovered surface material relative to total initial **4** + **1**.

Analysis Method	Initial on-NP **1** / μmol mg^{-1}	Initial on-NP **4** / μmol mg^{-1}	Post-exchange on-NP **1** / μmol mg^{-1}	Post-exchange on-NP **4** / μmol mg^{-1}	Post-exchange on-NP **1**:**4**	Recovered on-NP material (%)
TFA stripping	0.10	0.10	0.19	0.01	95:5	100
Acid digestion	0.12	0.11	0.22	0	100:1	96

15.3 Back-and-forth exchange of UiO-66-elec-4F with 2Fhyd and 4Fhyd

Starting from **UiO-66-elec-4F**, sequential treatment with **2Fhyd** (10 eq.) to produce **UiO-66-elec-2F-f-1**, then treatment with **4Fhyd** (25 eq.) to return **UiO-66-elec-2F-r-1** was repeated over three cycles (corresponding to six individual exchange processes).

Procedure

Cycling experiments were prepared as described in Section 15.1. After each cycle, the MOF was stored in ethanol to prevent aggregation and an aliquot of the sample was removed and dried for analysis.

Quantitative Analysis of Cycling Products

The dried MOF samples were characterised using ^{19}F qNMR spectroscopy under both acid digestion and TFA stripping treatments (procedures according to Sections 7.2 and 7.3, respectively). The data from TFA stripping and acid digestion treatments were also mutually consistent over all cycles (Table S13).

The forward cycles achieved a roughly 50:50 mix of products **1** and **4** on the surface which could be exchanged in the reverse cycles to an almost 100:0 mix of hydrazones **1** to **4**.

Table S13 Quantification of exchange products from back-and-forth exchange of **UiO-66-elec-4F** with **2Fhyd** (forward) and **4Fhyd** (reverse). Values derived from ^{19}F qNMR spectra following TFA stripping or acid digestion. All values quoted in μmol per milligram of solid. The total number of moles of surface species before exchange is n_i. n_{2F} are the number of moles of hydrazone **4** and n_{4F} are the number of moles of hydrazone **1** post-exchange.

UiO-66-elec-2F-x	n_i (TFA strip)	n_{2F} (TFA strip)	n_{4F} (TFA strip)	Ratio **4:1** (TFA strip)	n_i (digest)	n_{2F} (digest)	n_{4F} (digest)	Ratio **4: 1** (digest)
Initial	N/A	--	0.31	0:100	N/A	--	0.30	0:100
f-(1)	0.31	0.11	0.11	50:50	0.30	0.10	0.10	50:50
r-(1)	0.22	0.02	0.23	8:92	0.20	0.02	0.19	10:90
f-(2)	0.25	0.13	0.08	62:38	0.21	0.10	0.10	50:50
r-(2)	0.21	0.03	0.20	13:87	0.20	0.05	0.18	22:78
f-(3)	0.23	0.11	0.07	61:39	0.23	0.09	0.07	56:44
r-(3) (Final)	0.18	0.01	0.20	4:96	0.16	0	0.14	0:100

Supernatants of back-and-forth products

The net surface desorption compared to the starting **UiO-66-elec-4F** was also monitored. After an initial net surface loss of ca. 30% on the first exchange reaction, all subsequent cycles proceeded with negligible surface losses.

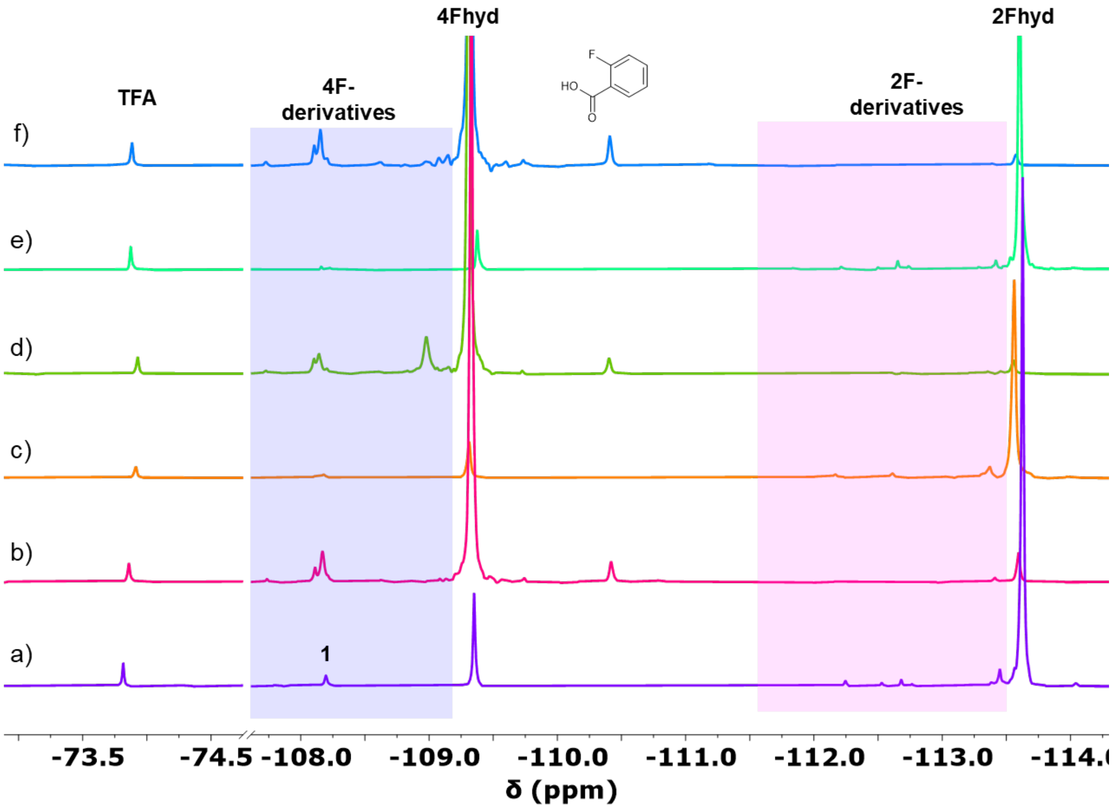

Figure S53 Analysis of supernatants from back-and-forth exchange reactions. ^{19}F NMR spectra (376.6 MHz, 10% *v*/*v* D_2O/DMF) after exchanges to produce products: a) **UiO-66-elec-2F-F1**; b) **UiO-66-elec-2F-R1**; c) **UiO-66-elec-2F-F2**; d) **UiO-66-elec-2F-R2**; e) **UiO-66-elec-2F-F3**; f) **UiO-66-elec-2F-R3**.

*Characterisation of final **UiO-66-elec-2F-r-(3)***

The structure of the MOF framework remained unchanged, as evidenced by identical PXRD patterns for the final isolated **UiO-66-elec-2F-r-(3)** product and initial **UiO-66-elec-4F** (Figure S54). Furthermore, the morphology of the NPs remained the unchanged on inspection by electron microscopy (Figure S55).

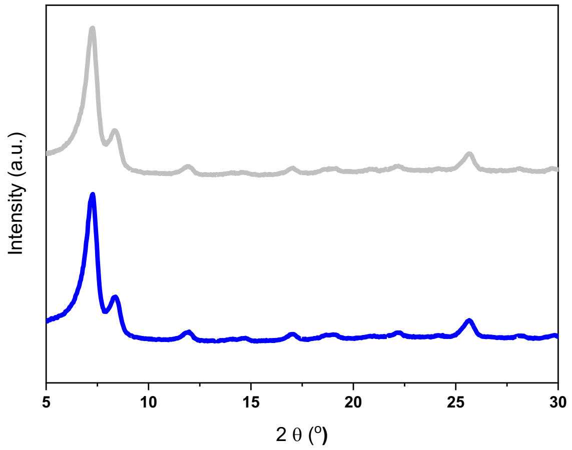

Figure S54 PXRD patterns of initial **UiO-66-elec-4F** (blue, bottom) and final product **UiO-66-elec-2F-r-(3)** (grey, top).

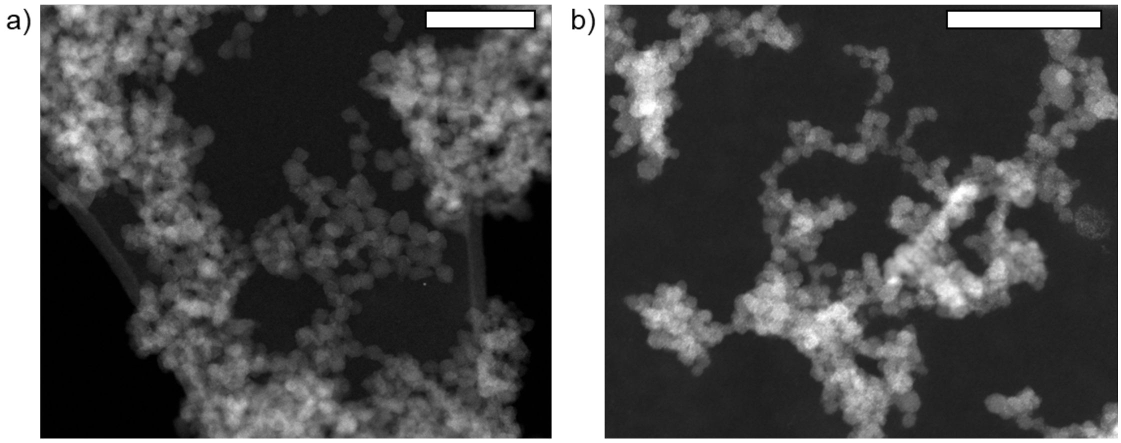

Figure S55 STEM-HAADF images of i) initial **UiO-66-elec-4F**, scale bar = 100 nm, ii) **UiO-66-elec-2F-r-(3)** exchange product, scale bar = 200 nm.

16. Synthesis and characterisation of reference nucleophilic MOF NPs

16.1 Synthesis of UiO-66 functionalized with nucleophilic hydrazone 5

UiO-66 was functionalized with **5** to form **UiO-66-nuc-bisF** as described in section 3.

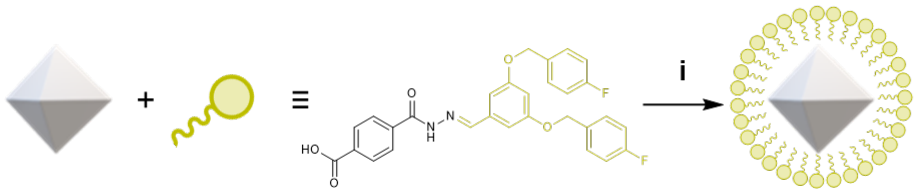

Scheme S12 Post-synthetic functionalization of UiO-66 with hydrazone **5** via coordination of the carboxylate to Zr nodes on the surface to form **UiO-66-nuc-bisF.** UiO-66 (0.096 mmol $C_{48}H_{28}O_{32}Zr_6$), **5** (0.134 mmol), dispersed in EtOH/DMF (3:2 *v*/*v*, ~10 mg/mL of MOF) at RT for 7 d.

16.2 Characterisation of UiO-66 functionalized with 5

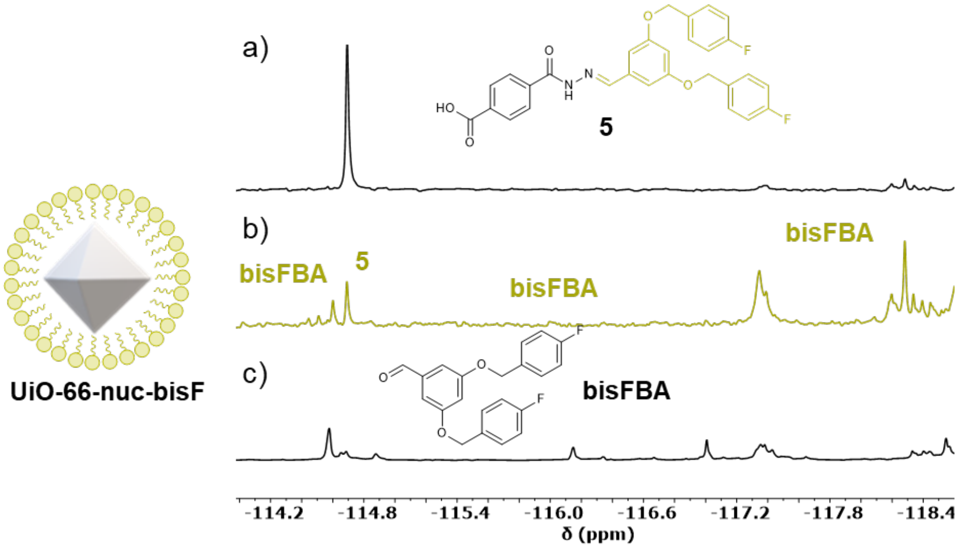

Figure S56 Acid digestion ^{19}F NMR spectra (376.7 MHz, DMSO-d_6) ~3 mg of MOF or molecule was digested with conc. H_2SO_4 (30 µL) in DMSO-d_6. a) Hydrazone **5**; b) **UiO-66-nuc-bisF**; iii) **bisFBA**.

The nucleophilic functionalized MOF reference sample was characterised by acid digestion ^{19}F NMR spectroscopy (Figure S56b). A signal at δ –114.69 ppm corresponding to hydrazone **5** was observed. Multiple additional signals were also observed, one was assigned to aldehyde **bisFBA** (δ –114.60 ppm, Figure S57b). It was established that the remaining signals are degradation products produced from aldehyde **bisFBA** under the acid digestion conditions (Figure S56c). Apparently, the presence of Lewis acidic MOF components accelerates the hydrolysis of **5** to produce **bisFBA** and subsequent degradation products, compared to **5** under acid digestion conditions alone (Figure S57a).

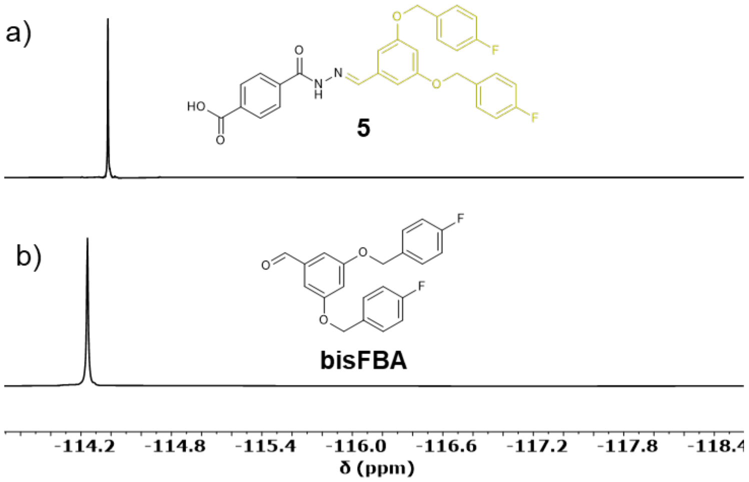

Figure S57 ^{19}F NMR spectra (470.4 MHz, DMSO-d_6) of i) hydrazone **5** and ii) **bisFBA**.

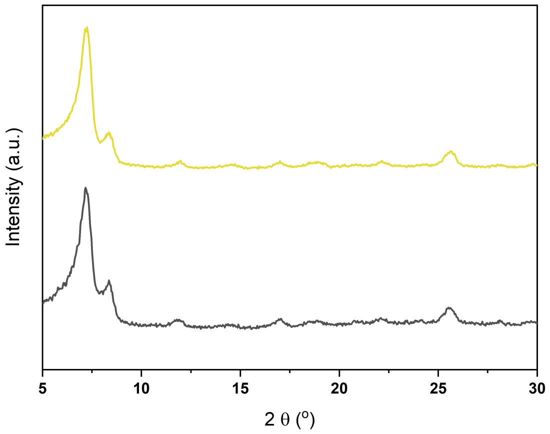

Figure S58 PXRD patterns of pristine UiO-66 (bottom, black) and **UiO-66-nuc-bisF** (top, yellow).

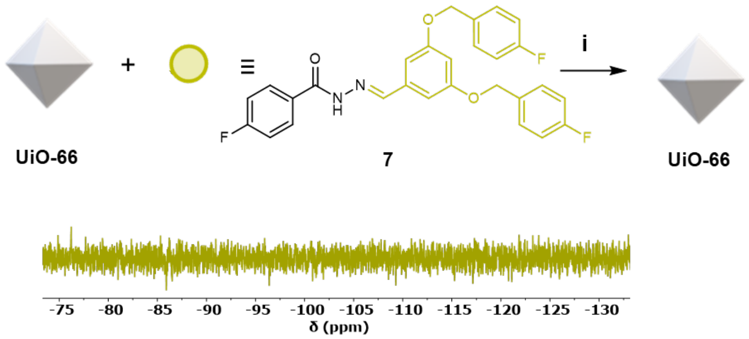

Figure S59 Control modification of UiO-66 using analogue hydrazone **7** lacking a carboxylate binding site. Reaction conditions: i) UiO-66 (0.064 mmol $C_{48}H_{28}O_{32}Zr_6$) dispersed in EtOH/DMF (3:2 *v*/*v*, ~10 mg/mL) at RT for 7 d, **7** (0.089 mmol). Acid digestion ^{19}F NMR spectrum (376.7 MHz, DMSO-d_6). ~3 mg of MOF digested with conc. H_2SO_4 (30 μL) in DMSO-d_6.

17. Exchange between UiO-66-nuc-4F and UiO-66-nuc-bisF

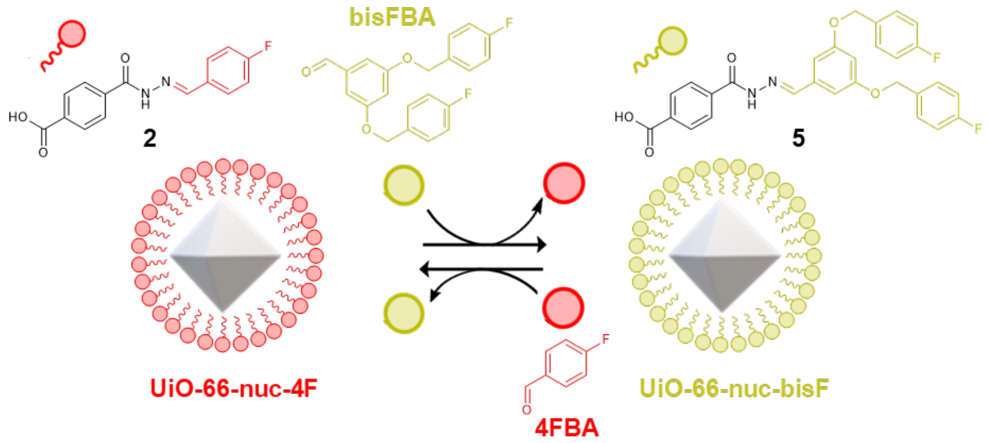

Scheme S13 Schematic representation of exchange between **UiO-66-nuc-4F** and **UiO-66-bisF.**

17.1 Modification of UiO-66-nuc-4F by exchange with bisFBA

Procedure

UiO-66-nuc-4F (13 mg) was recovered from its ethanolic storage solution by evaporation of ethanol in a drying oven at 60 °C and atmospheric pressure.

A stock solution of 10 mM 4-fluorotoluene in 10% *v*/*v* D_2O/DMF was prepared and this was used as the solvent mixture for all further stock solutions.

Stock solutions of all modifiers and catalysts were first prepared volumetrically using the internal standard solvent mixture.

Recovered dried **UiO-66-nuc-4F** was suspended in the solvent stock solution (1.28 mL) and dispersed via ultrasonication. The dispersion was then treated with **bisFBA** modifier stock solution (0.5 mL, 100 mM) then a mixture of acetic acid (0.02 mL, 200 mM) and aniline (0.2 mL, 200 mM) was added, giving final concentrations: [**2**] = 2.5 mM, [**bis-FBA**] = 25 mM, [acetic acid] = 2.0 mM, [aniline] = 20 mM.

The solution was left to stir at 35 °C for 7 d, and then the MOF separated from the reaction mixture by centrifugation (14,800 rpm, 15 min).

The reaction solution was collected for analysis and the MOF was then purified by washing-dispersion cycles (1x DMF, 1x ethanol; recovery by centrifugation at 14,800 rpm for 15 min each time). The purified MOF was dried in an oven at 60 °C and atmospheric pressure and then analysed.

Analysis of exchange supernatant

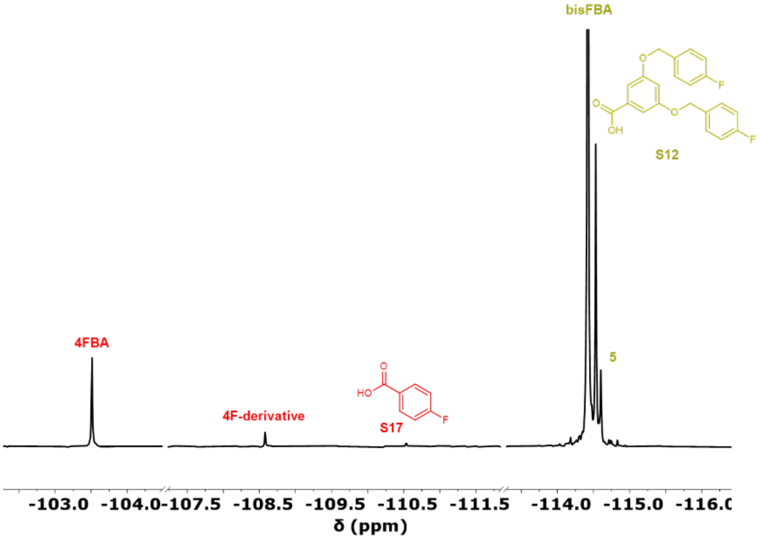

Figure S60 ^{19}F NMR spectrum (376.7 MHz, 10% v/v D_2O/DMF) of the supernatant after **bisFBA** exchange with **UiO-66-nuc-4F**.

The supernatant of the exchange was analysed (Figure S60). Some hydrazone **5** was observed but could not be quantified owing to overlapping signals from other **bisFBA** derivatives. A sharp signal at δ_F = –108.6 ppm was attributed to a side product of the 4-fluorobenzylidine unit. Additionally, a signal corresponding to acid **S12**, the oxidation product of **bisFBA**, was observed. No evidence of **S12** binding to the MOFs under the exchange conditions was identified (Figure 4b).

*Quantification of exchange product **UiO-66-nuc-bisF-f***

Table S14 Quantification of exchange from **UiO-66-nuc-4F** with **bisFBA**. Values derived from ^{19}F qNMR spectra of **UiO-66-nuc-bisF-f** (following TFA stripping or acid digestion) and of reaction supernatant. All molar values quoted in μmol per milligram of solid. Percentages calculated relative to initial surface quantity of **2**.

Analysis method	Initial on-NP **2** / μmol mg^{-1}	Post-exchange on-NP **5** / μmol mg^{-1}	Post-exchange on-NP **2** / μmol mg^{-1}	Post-exchange on-NP **2**:**5**	Recovered on-NP material (%)
TFA stripping	0.35	0.18	0.03	14:86	60
Acid digestion	0.30	0.13	0.03	19:81	53

PXRD analysis of UiO-66-nuc-bisF-f

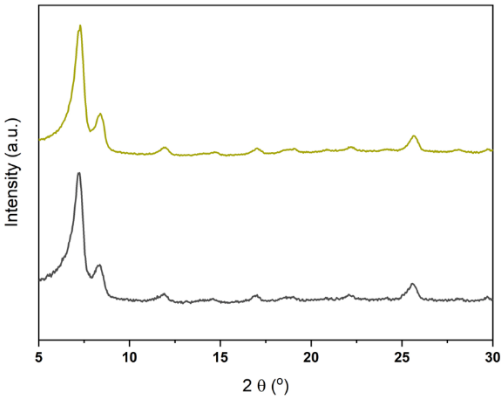

Figure S61 PXRD pattern of initial **UiO-66-nuc-4F** (black, bottom) and exchange product **UiO-66-nuc-bisF-f** (yellow, top).

17.2 Reverse exchange of UiO-66-nuc-bisF-f with 4FBA

Procedure

Dried **UiO-66-nuc-bisF-f** (0.89 mM in terms of **5**; [total hydrazone] ≈ 1.1 mM) was dispersed via sonication in 10% *v*/*v* D_2O/DMF (0.8 mL) containing **4FBA** (45 mM), acetic acid (2 mM), aniline (20 mM) and 4FT IS (10 mM).

The solution was left to stir at 35 °C for 7 days, and then the MOF separated from reaction mixture by centrifugation (14,800 rpm, 20 min).

The solid was then purified by washing-dispersion cycles (1x DMF, 1x ethanol; recovery by centrifugation at 14,800 rpm for 20 min each time). The purified MOF was dried in an oven at 60 °C and atmospheric pressure and then analysed.

Quantitative analysis of exchange product ***UiO-66-nuc-bisF-r***

Table S15 Quantification of exchange from **UiO-66-nuc-bisF-f** with **4FBA**.Values derived from ^{19}F qNMR spectra of **UiO-66-nuc-bisF-r** following acid digestion. All molar values quoted in μmol per milligram of solid.

Analysis method	Initial on-NP **2** / μmol mg^{-1}	Initial on-NP **5** / μmol mg^{-1}	Post-exchange on-NP **2** / μmol mg^{-1}	Post-exchange on-NP **5** / μmol mg^{-1}	Post-exchange on-NP **2**:**5**	Recovered on-NP material (%)
Acid digestion	0.03	0.13	0.10	0.06	63:37	100

A quantity of 4-fluorobenzoic acid (**S17**) was also observed associated with the MOF post-exchange. We attribute this to oxidation of excess **4FBA** over the course of the exchange reaction. By contrast, during the forward exchange reaction (Section 17.1) **bisFBA** is less susceptible to aerobic oxidation and the steric bulk of its oxidation product **S12** inhibits association with the MOF.

18. Characterisation of unfunctionalized Al-fum particles

Al-fum synthesised following procedure described in Section 3.

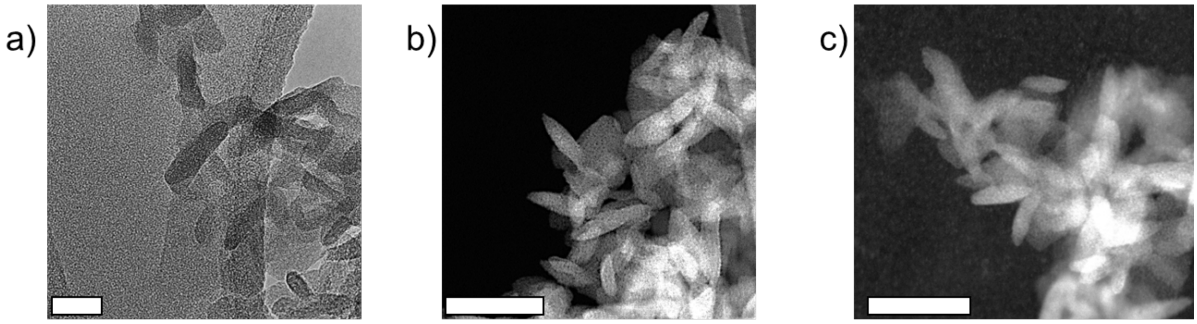

Figure S62 Transmission Electron Microscopy (TEM) images of the unfunctionalized Al-fum <w_{TEM}> = 9 (1) nm, <l_{TEM}> = 28 (4) nm. Scale bars a) 20 nm, b) 50 nm, and c) 50 nm.

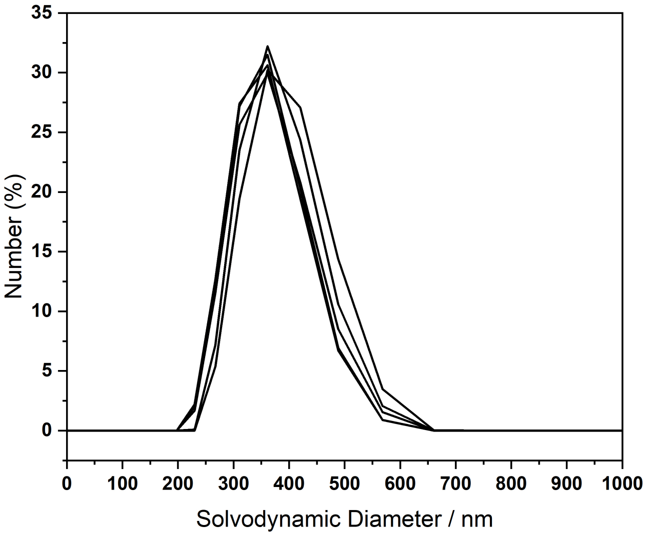

Figure S63 Dynamic light scattering of unfunctionalized Al-fum nanoparticles dispersed in ethanol (0.25 mg/mL) <d_{EtOH}> = 367 (13) nm.

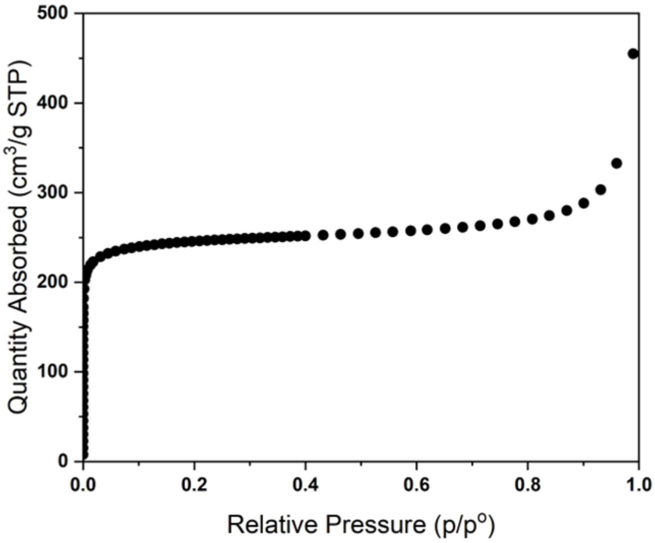

Figure S64 N_2 adsorption (77 K) plot of the unfunctionalized Al-fum resulting in a BET surface area of 976 m^2 g^{-1}.

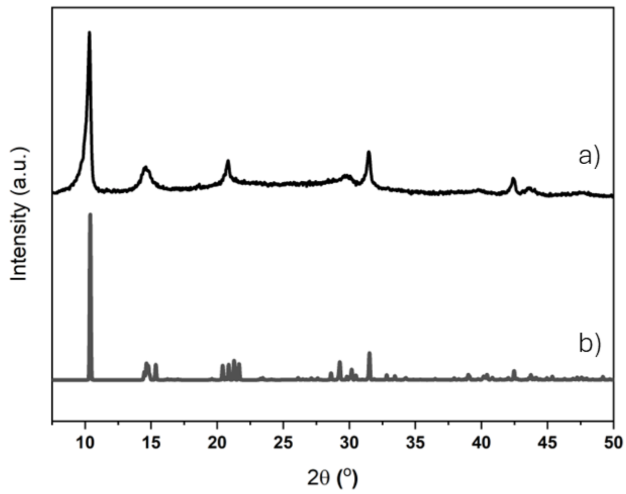

Figure S65 Powder X-ray diffraction patterns of a) ‘ideal’ Al-fum,[12] and b) as-synthesised unfunctionalized Al-fum.

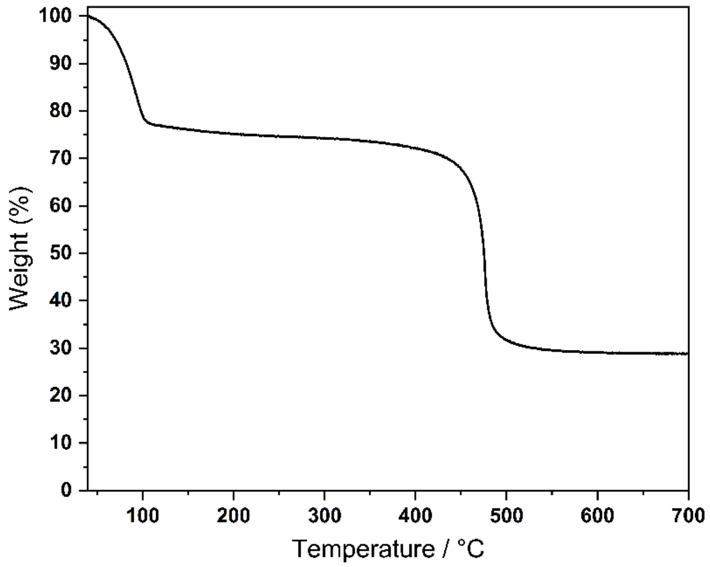

Figure S66 Thermogravimetric Analysis (TGA) plot of Al-fum indicating thermal stability up to 400 °C.

19. Characterisation of functionalized Al-fum particles

Al-fum synthesised and functionalized following procedures described in Section 3.

19.1 Structural characterisation of Al-fum-nuc-4F

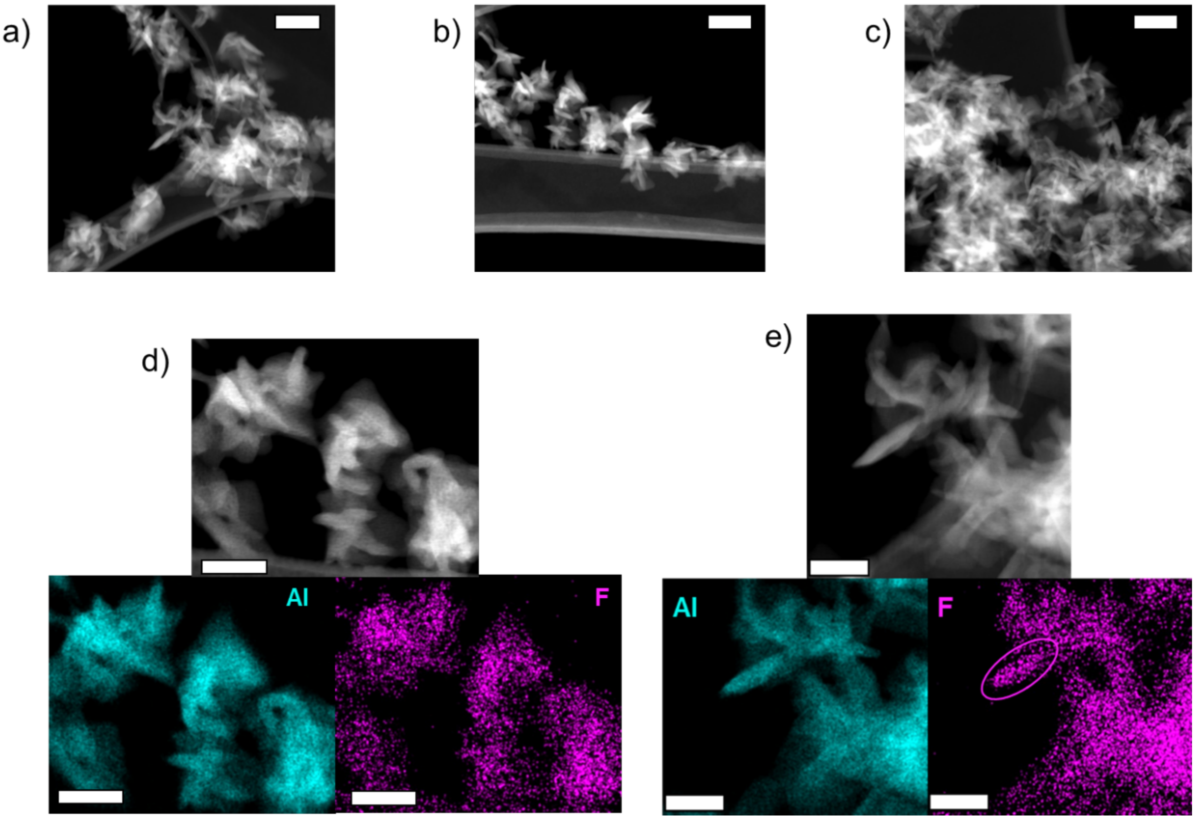

Figure S67 HAADF STEM images of **Al-fum-nuc-4F**. Scale bars: a)-c) 100 nm. d)-e) STEM-EDX mappings of **Al-fum-nuc-4F** (scale bars = 50 nm; cyan = Al; pink = F)

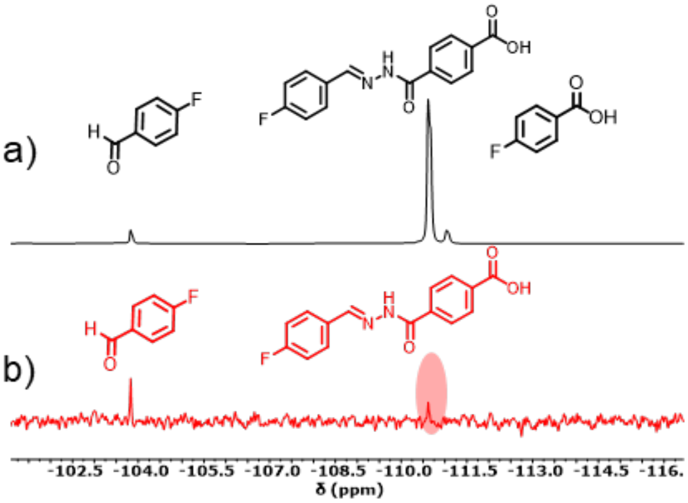

Figure S68 Acid digestion ^{19}F NMR spectra (376.7 MHz, DMSO-d_6), in which ~3 mg of MOF or molecule, is digested with conc. H_2SO_4 (30 µL) in DMSO-d_6. (i) Hydrazone **2**, (ii) **Al-fum-nuc-4F**.

a)

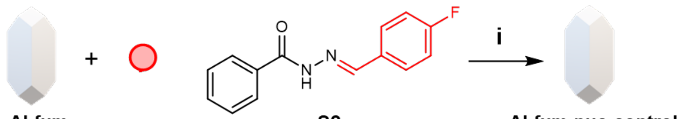

b)

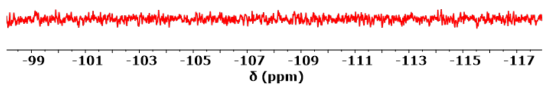

Figure S69 (a) Control modification of Al-fum with hydrazone **S2** lacking a carboxylate binding site. Reaction conditions: i) Al-fum (0.217 mmol $Al(OH)(O_2C(CH)_2CO_2)$), **S2** (0.304 mmol), EtOH/DMF (3:2 *v*/*v*, 5 mL), RT, 7 d. (b) Acid digestion ^{19}F NMR spectrum (376.7 MHz, DMSO-d_6) in which ~3 mg of MOF, is digested with conc. H_2SO_4 (30 μL).

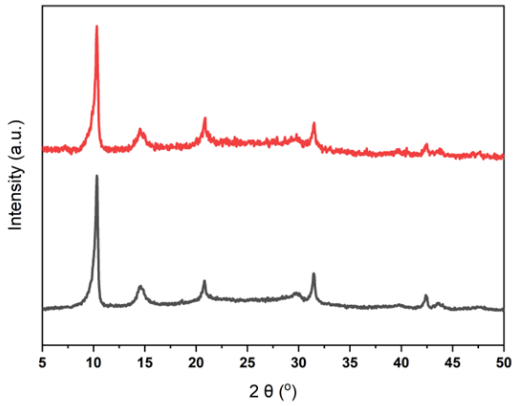

Figure S70 PXRD patterns of unfunctionalized Al-fum (black, bottom) and **Al-fum-nuc-4F** (red, top).

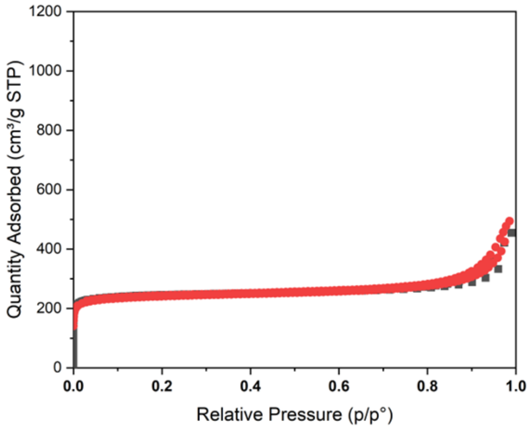

Figure S71 N_2 adsorption (77 K) plot of the unfunctionlized Al-fum resulting a BET surface area of 976 m^2 g^{-1} (black) and functionalized **UiO-66-4Fhyd-nuc** of 946 m^2 g^{-1}.

19.2 Quantitative analysis of Al-fum-nuc-4F

It was found that TFA stripping (Section 7.3) gave the most consistent results for quantitative analysis of Al-fum MOFs.

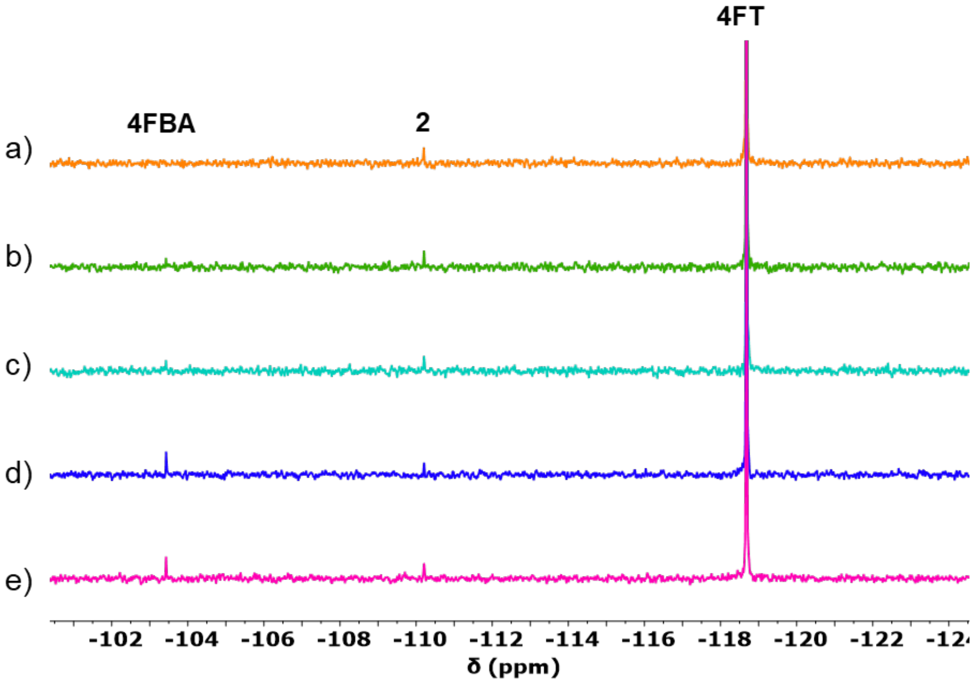

Figure S72 ^{19}F qNMR spectra (376.7 MHz, D_2O) of **Al-fum-nuc-4F**. TFA (100 mM in 10% *v*/*v* D_2O/DMF) after a) < 1 h, b) 24 h, c) 48 h, d) 8 d, and e) 3 weeks.

Table S16 Quantitative analysis of the TFA stripping ^{19}F qNMR spectra of **Al-fum-nuc-4F.**

Time	[**4FBA**] / mM	[**2**] / mM	[**4FBA**] + [**2**] / mM	Total quantity **2** initially present / µmol	Mass concentration **2** / µmol mg^{-1}
< 1 h [a]	0	0.26	0.26	0.154	0.052
24 h	0	0.26	0.26	0.159	0.054
48 h	0.14	0.21	0.35	0.213	0.072
8 d	0.22	0.20	0.42	0.251	0.085
3 weeks	0.28	0.19	0.47	0.283	0.096

[a] The first spectrum recorded immediately following sample preparation after a wait time dependant on spectrometer availability.

19.3 Structural characterisation of Al-fum-elec-4F

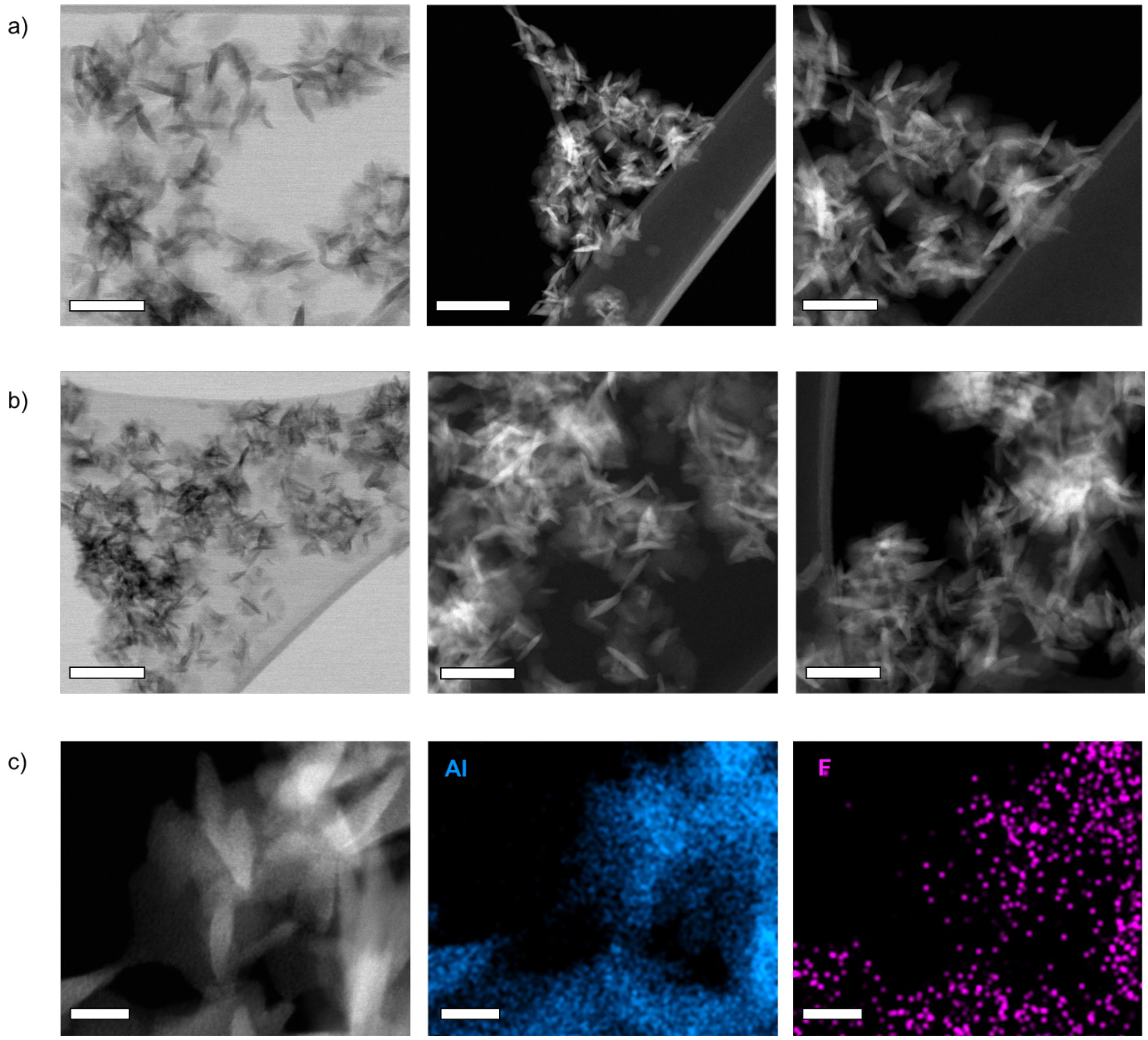

Figure S73 STEM-EDX images of **Al-fum-elec-4F**. Scale bars: a) 100 nm; b) 200 nm; c) 100 nm; d) 200 nm; e) 100 nm; f) 100 nm; g); . Blue = Al; pink = F.

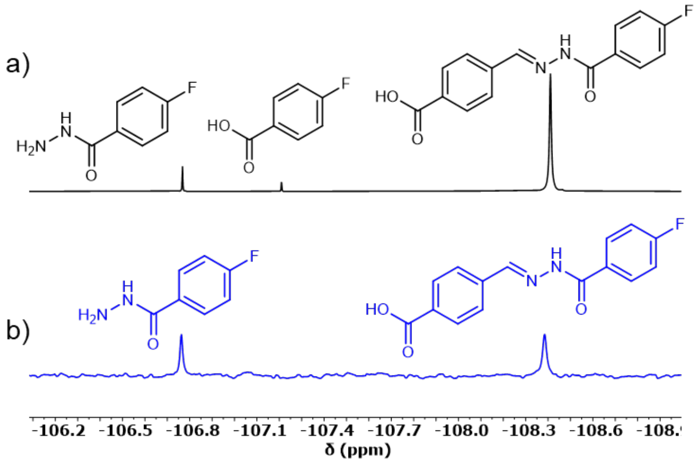

Figure S74 Acid digestion ^{19}F NMR spectra (376.7 MHz, DMSO-*d*$_6$), in which ~3 mg of MOF or molecule, is digested with conc. H_2SO_4 (30 μL) in DMSO-d$_6$. (i) authentic hydrazone **1**, (ii) **Al-fum-elec-4F** .

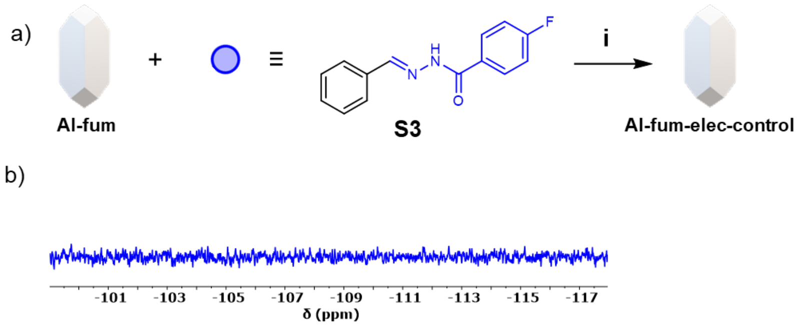

Figure S75 (a) Control modification of Al-fum with hydrazone **S1** to form **Al-fum-elec-control**. Conditions: i) Al-fum (0.174 mmol $Al(OH)(O_2C(CH)_2CO_2)$), **S1** (0.244 mmol) in (4:1) EtOH/DMF (5 mL), RT, 7 d. (b) Acid digestion ^{19}F NMR spectrum (376.7 MHz, DMSO-d_6) in which ~3 mg of **Al-fum-elec-control** is digested with conc. H_2SO_4 (30 µL).

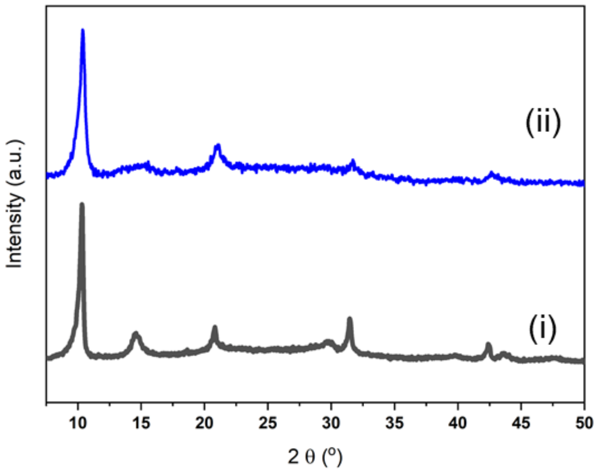

Figure S76 PXRD patterns of (i) unfunctionalized Al-fum; (ii) **Al-fum-elec-4F**.

19.4 Quantitative analysis of Al-fum-elec-4F

Surface functionalization of **Al-fum-elec 4F** was quantitatively assessed using the TFA stripping method (Section 7.3).

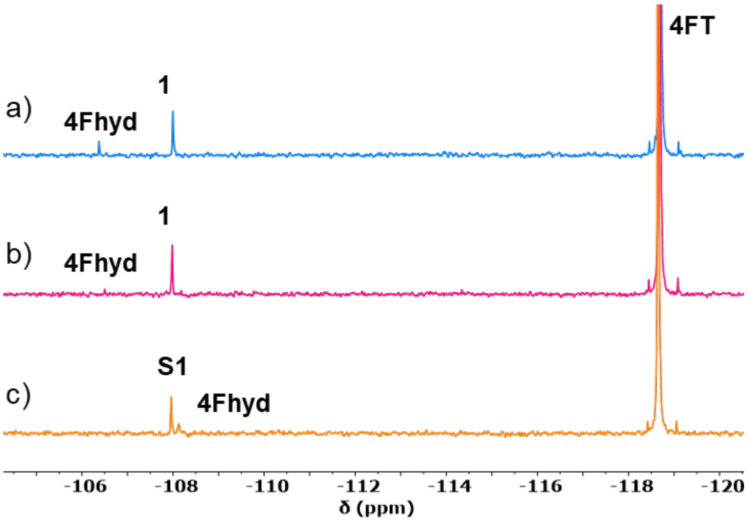

Figure S77 ^{19}F qNMR spectra (376.7 MHz, 10%v/v D_2O/DMF) of **Al-fum-elec-4F**. TFA (100 mM) in 10% v/v D_2O/DMF over time after a) < 1 h, b) 24 h, c) 1 week.

Table S17 Quantitative analysis of the TFA stripping ^{19}F qNMR spectra of **Al-fum-elec-4F.**

Time	[**4Fhyd**] / mM	[**1**] / mM	[**4Fhyd**] + [**1**] / mM	Total quantity **1** initially present / μmol	Mass concentration **1** / μmol mg^{-1}
< 1 h [a]	0.06	0.28	0.34	0.204	0.062
24 h	0.02	0.25	0.27	0.162	0.050
7 d	0.09	0.23	0.32	0.189	0.058

[a] The first spectrum recorded immediately following sample preparation after a wait time dependant on spectrometer availability.

20. Heterostructure assembly

20.1 Formation of heterostructure assemblies

General procedure

A stock solution of 10 mM 4FT in 10% *v*/*v* D_2O/DMF was prepared and this was used as the solvent mixture for all further stock solutions.

A stock solution of TFA (100 mM) was prepared volumetrically by diluting with the internal standard solvent stock.

Recovered dried **UiO-66-elec-4F** (1.43 mg) (~2.5 mM in terms of **1**) and **Al-fum-nuc-4F** (7.2 mg) (~2.5 mM in terms of **2**) were redispersed into the reaction solvent mixture (1996 µL) via ultrasonication. An aliquot of TFA stock (4 µL) was then added and the mixture was stirred at 35 °C for 1 week. The supernatant was collected via centrifugation at 14,800 rpm for 20 min and analysed by ^{19}F NMR spectroscopy. The solid residue was washed with DMF followed by EtOH (collection by centrifugation at 14,800 rpm, 20 min each time). The assembly product was kept in suspension until further analysis.

20.2 Characterisation of heterostructure assemblies

Electron microscopy images, batch 1

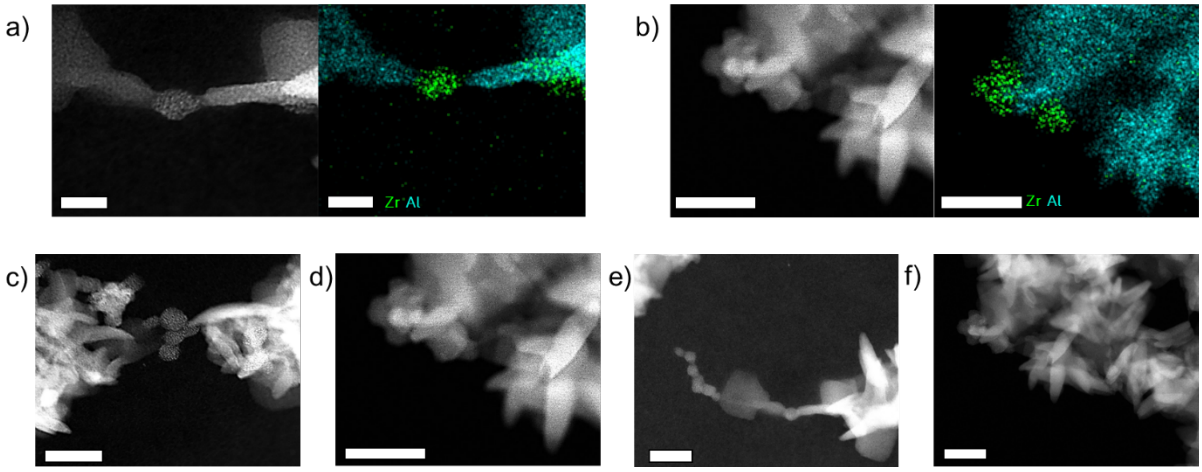

Figure S78 STEM-EDX images of heterostructure assemblies from **UiO-66-elec-4F** and **Al-fum-nuc-4F** (batch 1). Green = Zr and cyan = Al; scale bars: a) 20 nm; b)-f) 50 nm.

Electron Microscopy Images for batch 2

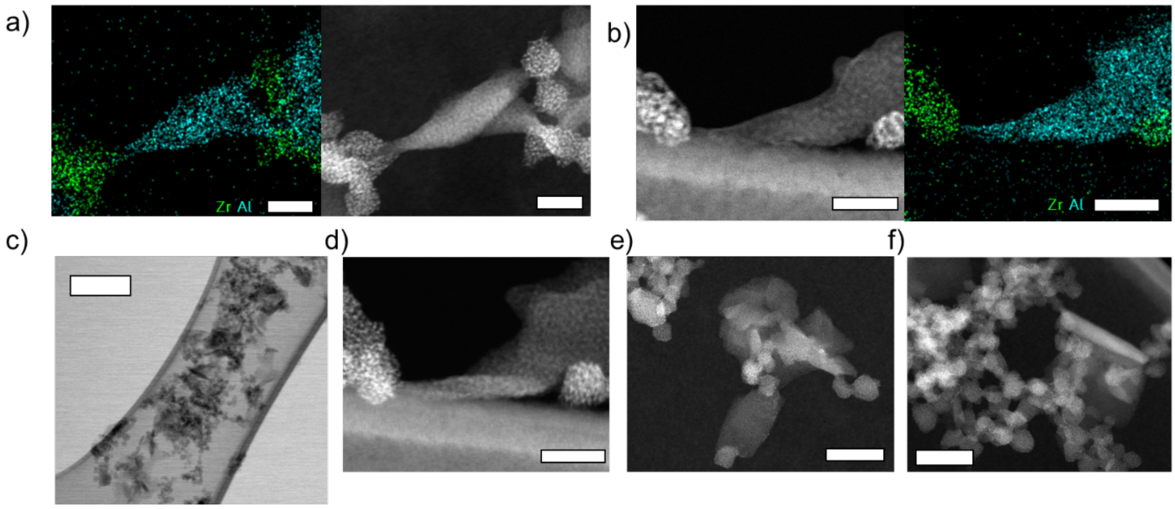

Figure S79 STEM-EDX images of heterostructure assemblies from **UiO-66-elec-4F** and **Al-fum-nuc-4F** (batch 2). Green = Zr and cyan = Al; scale bars: a) 20 nm; b) 20 nm; c) 200 nm; d) 20 nm; e) 50 nm; f) 50 nm.

Supernatant analysis

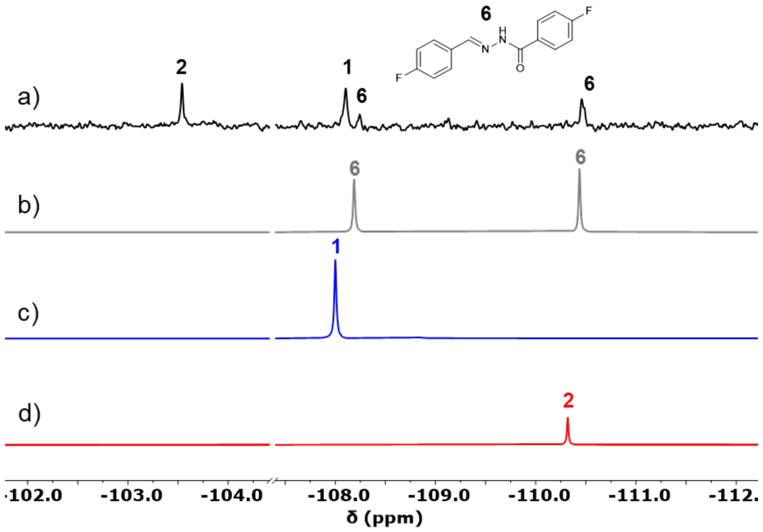

Figure S80 ^{19}F NMR spectra (376.7 MHz, 10% *v*/*v* D_2O/DMF) of a) supernatant after heterostructure assembly batch 1, b) authentic hydrazone **6**, c) authentic hydrazone **1**, and d) authentic hydrazone **2**.

BET Measurements

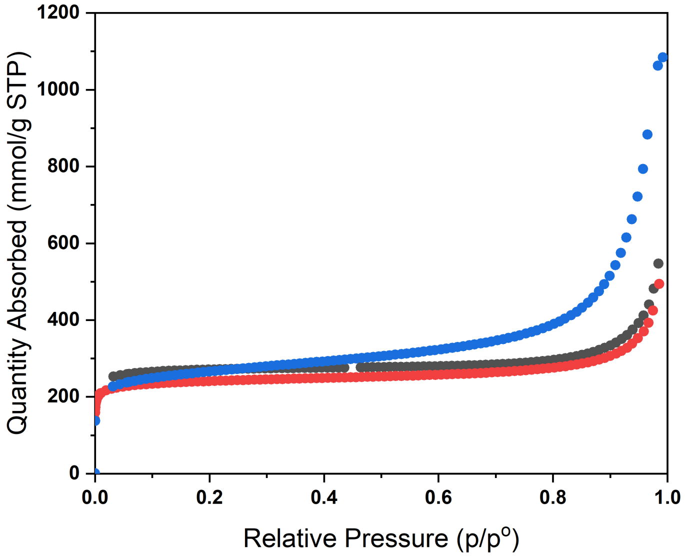

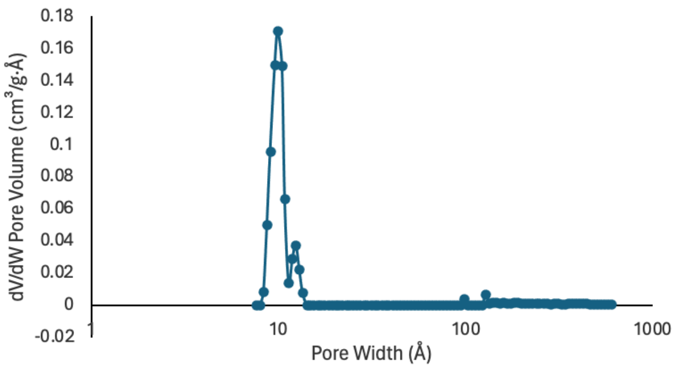

Figure S81 (Top) N_2 adsorption (77 K) isotherms after activation at 150 °C for 16 hours of **Al-fum-nuc-4F** (red, 946 m^2 g^{-1}), **UiO-66-elec-4F** (blue, 998 m^2 g^{-1}) and heterostructure assembly batch 1 (black, 1062 m^2 g^{-1}). (Bottom) the pore size distribution for the heterostructure assembly (NLDFT with carbon slit pores)

PXRD

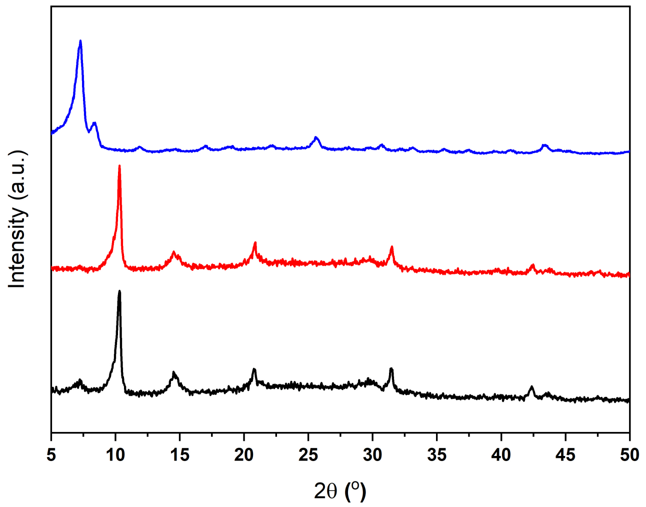

Figure S82 PXRD patterns of **UiO-66-elec-4F** (blue, top), **Al-fum-nuc-4F** (red, middle), and heterostructure assembly batch 1 (black, bottom).

20.3 Heterostructure assembly control experiments

Control 1

Control 1 was performed in the same manner as described for heteromaterial assembly in Section 20.1 but replacing the TFA with blank reaction solvent mixture. Representative TEM images (Figure S83) show particle self-sorting with distinct regions of exclusively spherical UiO-66 particles or ellipsoidal Al-fum particles.

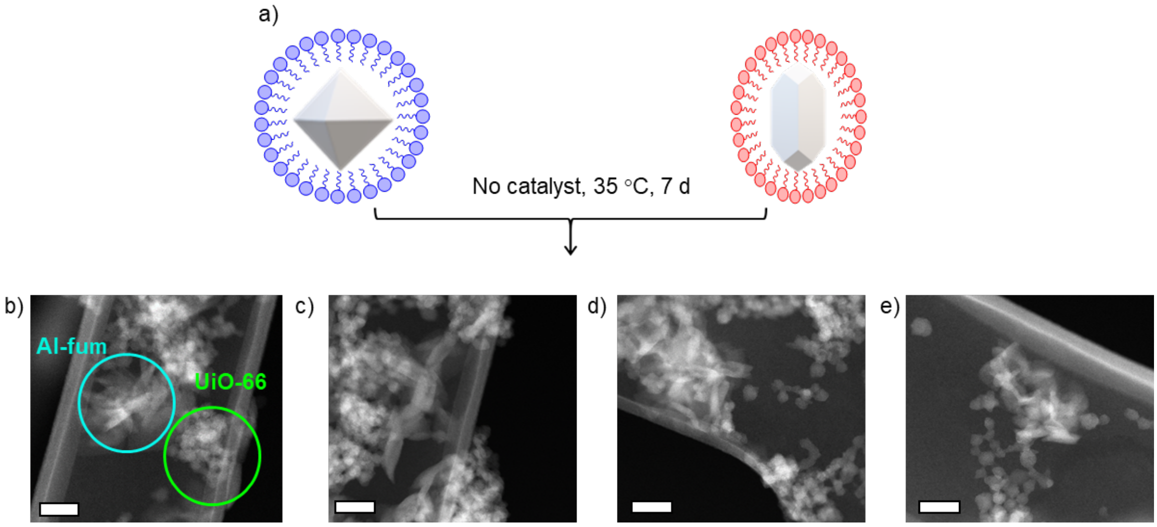

Figure S83 a) Schematic representation of heterostructure assembly control 1 (omitting acid catalyst). b)–e) Representative TEM images showing discrete aggregates of Al-fum (cyan) and UiO-66 NPs (green). Scale bars: 50 nm.

Control 2

Control 2 was performed in the same manner as described for heteromaterial assembly in Section 20.1 but using unfunctionalized UiO-66 (1.43 mg) and Al-fum (7.3 mg) in place of the functionalized dynamic covalent NPs.

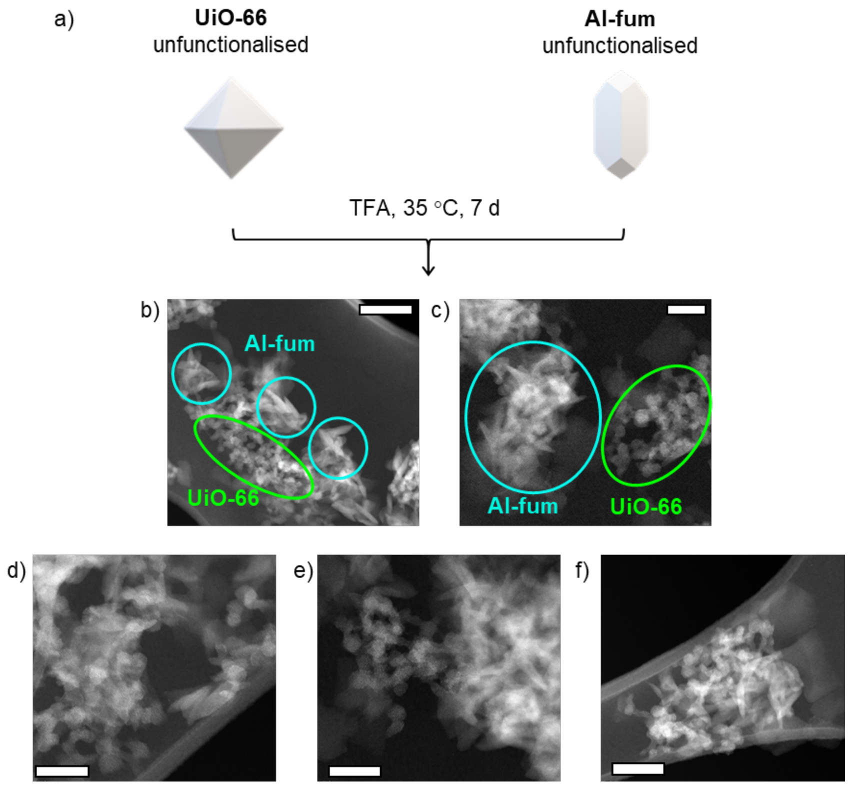

Figure S84 a) Schematic representation of heterostructure assembly control 2 (omitting reactive hydrazone functionality). b)–f) Representative TEM images showing discrete self-sorted areas of Al-fum (cyan) and UiO-66 (green). Scale bars: (b) 100 nm; (c)–(f) 50 nm.

21. Characterisation of AuNP-nuc

Synthesis described in Section 4.

21.1 Nanoscale structural analysis

TEM analysis revealed spherical AuNPs of <d_{TEM}> = 5.2 ± 0.9 nm (Figures S85–S86).

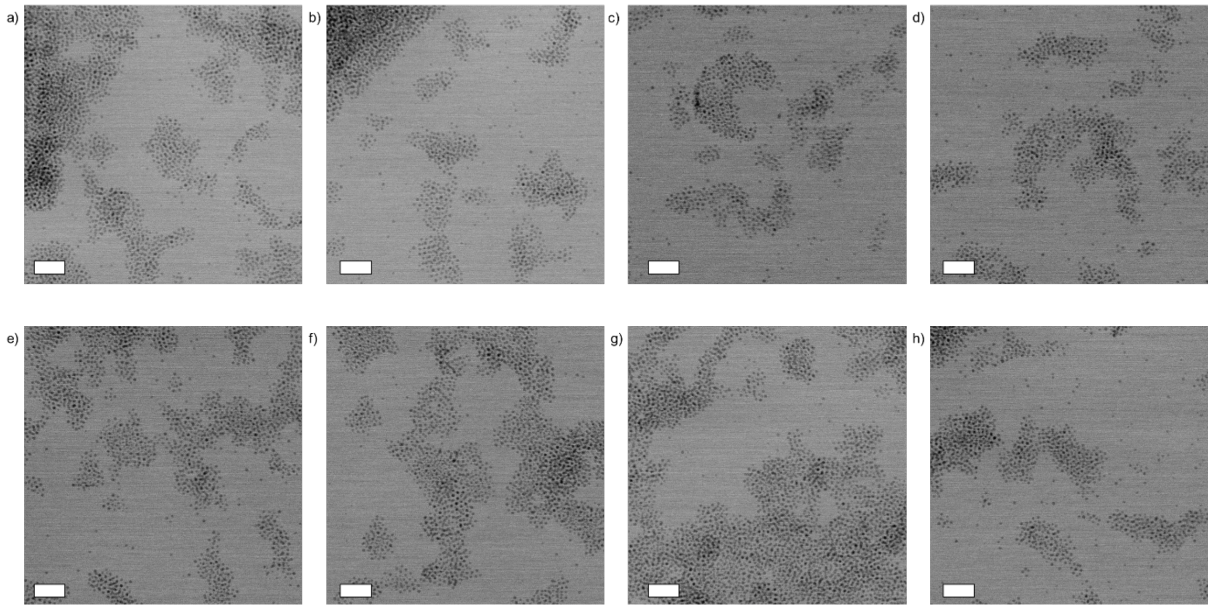

Figure S85 Representative TEM images of **AuNP-nuc**; scale bars = 50 nm.

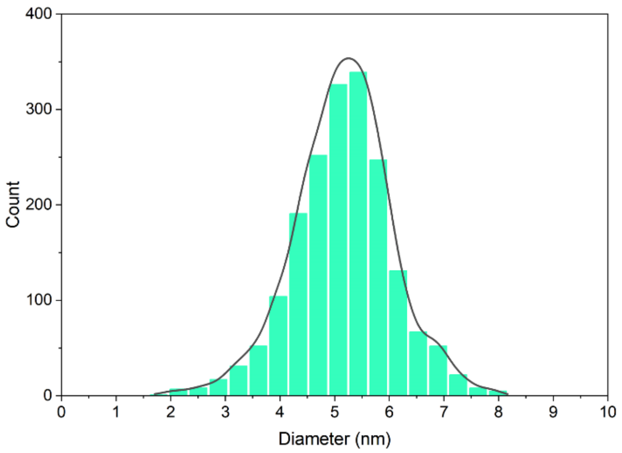

Figure S86 Histogram of **AuNP-nuc** size distribution as determined from analysis of multiple TEM images <d_{TEM}> = 5.2 ± 0.9 nm.

TGA analysis (Figure S87) was used to estimate the molar weight and number of ligands per nanoparticle (Table S18), assuming an isotropic gold core (d = <d_{TEM}> = 5.2 nm, Figure S86)

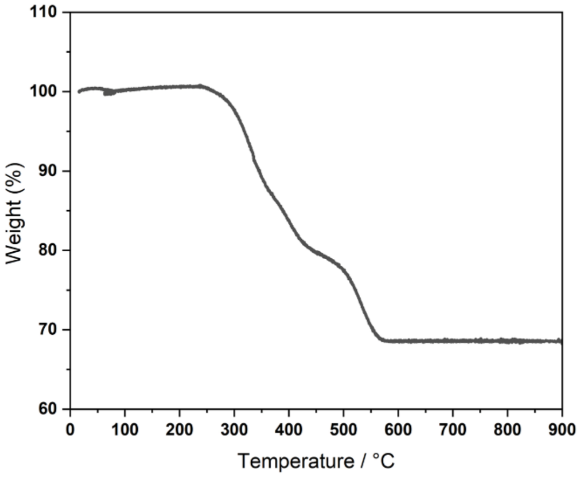

Figure S87 TGA plot for **AuNP-nuc**.

Table S18 Quantitative nanoscale and molecular scale characterisation of **AuNP-nuc.**

<d_{TEM}> / nm	**AuNP-nuc** surface area / nm^2	Au wt%	**S16** wt%	Surface area per **S16** / nm^2	**AuNP-nuc** M.W. / g mol^{-1}	Copies **S16** per **AuNP-nuc**	Mass concentration **S16** in **AuNP-nuc** / μmol mg^{-1}
5.2	84.9	32	68	0.18	1.26×10^6	475	0.38

21.2 Molecular structural analysis

In situ NMR spectroscopic analysis indicated that ligand **S16** was present on the surface of **AuNP-nuc** (Figure S88b, e). A T_2-filtered ^{1}H NMR spectrum recorded using the CPMG-z pulse sequence[13] showed signals corresponding only to residual non-deuterated solvents and grease (Figure S88c) confirming that **AuNP-nuc** had been successfully isolated free of all unbound molecular species.

Ligand desorption was achieved by treating a sample of **AuNP-nuc** in CD_2Cl_2 with a small quantity of iodine, allowing *ex situ* analysis of the molecular compoents released into solution. Both ^{1}H and ^{19}F NMR spectra (Figure 89b, d) indicate that ligand **S16** was the sole surface-bound component on **AuNP-nuc**.

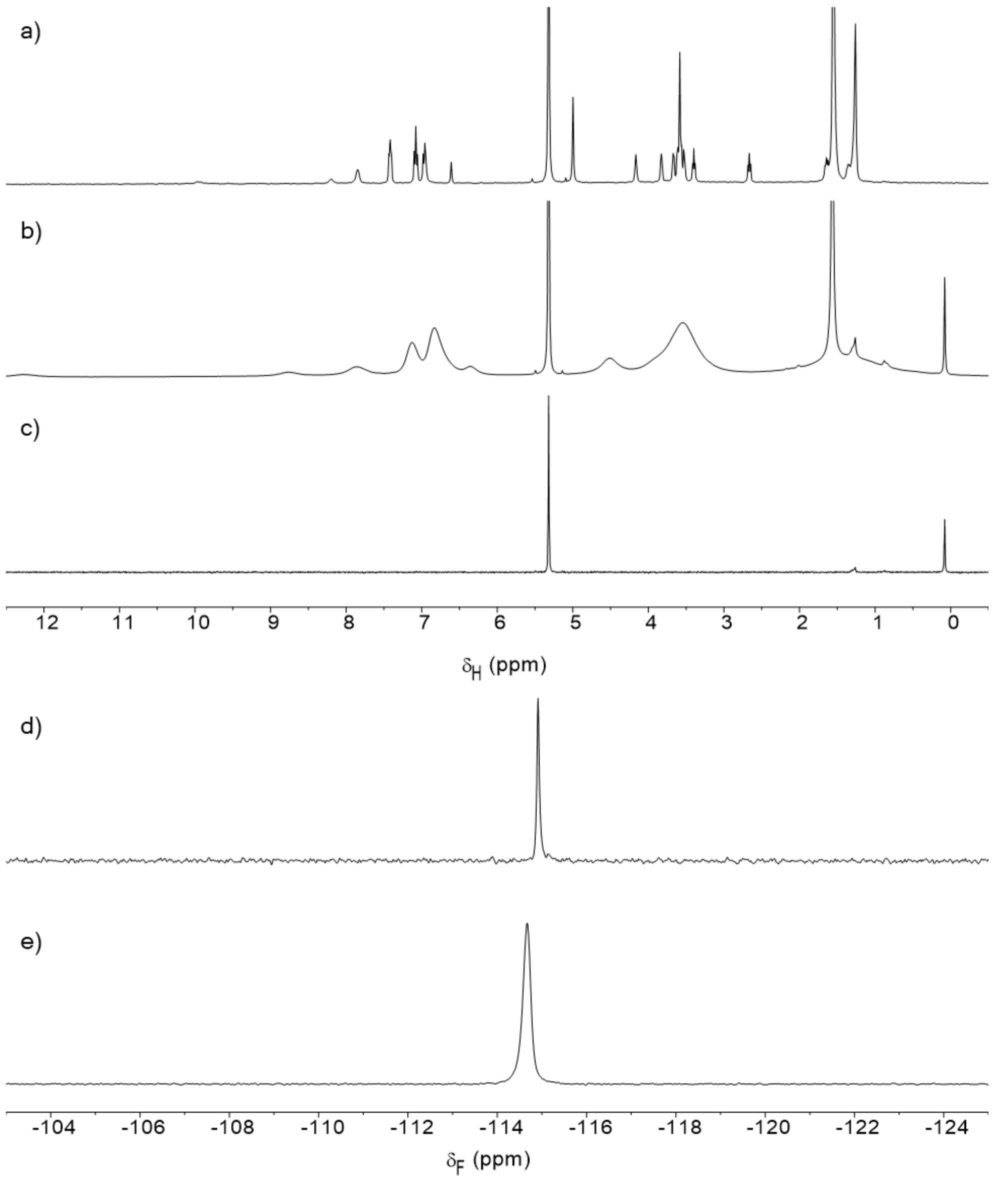

Figure S88 *In situ* NMR spectroscopic characterization of **AuNP-nuc**. (a–c) ^{1}H NMR spectra (CD_2Cl_2, a: 400.3 MHz; b, c: 500.1 MHz) of (a) pro-ligand reference **S16**$_2$; (b) **AuNP-nuc**; (c) T_2-filtered ^{1}H NMR spectrum of **AuNP-nuc** acquired using the CPMG-z pulse sequence. All sharp peaks can be assigned to residual non-deuterated solvents or grease. (d–e) ^{19}F NMR (CD_2Cl_2, 376.7 MHz) spectra of (d) pro-ligand reference **S16**$_2$; (e) **AuNP-nuc**.

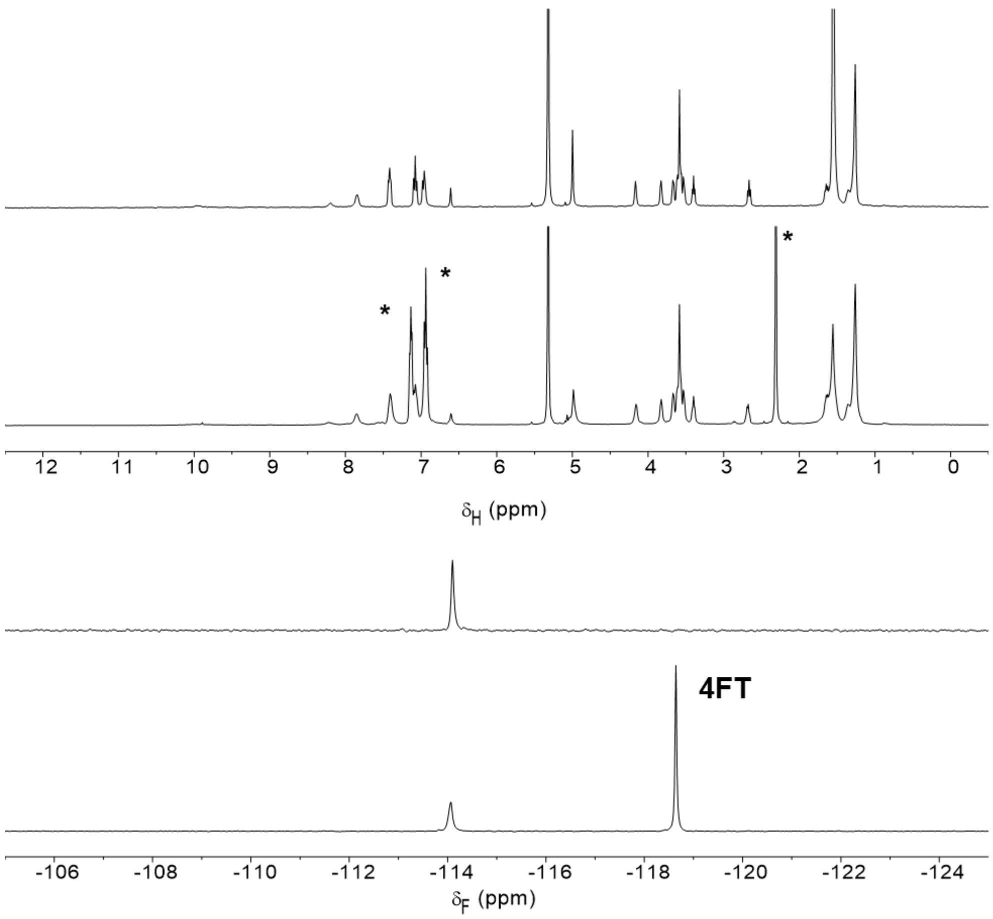

Figure S89 *Ex situ* NMR spectroscopic characterization of **AuNP-nuc**. (a–b) ^{1}H NMR spectra (CD_2Cl_2, a: 400.3 MHz) of (a) pro-ligand reference **S16**$_2$; (b) **AuNP-nuc** + I_2, 3 h; (c–d) ^{19}F NMR (CD_2Cl_2, 376.7 MHz) spectra of (c) pro-ligand reference **S16**$_2$; (d) **AuNP-nuc** + I_2, 3 h. Signals marked (*) correspond to contamination with toluene during sample handling.

Quantitative NMR characterisation

A loading of 0.38 µmol of ligand **S16** per milligram of AuNP was determined from the ^{19}F qNMR spectrum (Figure S90), in agreement with the quantitative results from TGA analysis (Table S18).

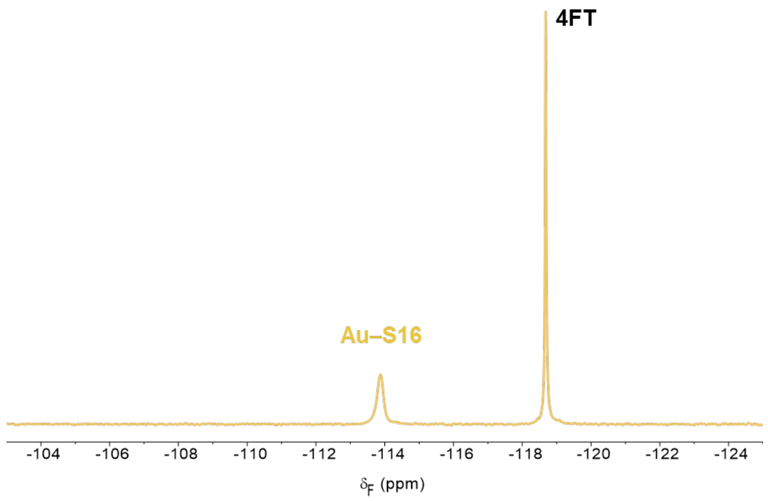

Figure S90 a) *In situ* quantitative ^{19}F NMR spectrum (376.7 MHz, CD_2Cl_2) of **AuNP-nuc**.

22. Heteromaterial assemblies

22.1 Assembly of UiO-66_Au heteromaterial

General procedure

A stock solution of 10 mM 4FT in 10% *v*/*v* D_2O/DMF was prepared and this was used as the solvent mixture for all further stock solutions.

A stock solution of TFA catalyst (100 mM) was prepared volumetrically by diluting with the internal standard solvent stock.

UiO-66-elec-4F was recovered and dried from its ethanolic storage solution then redispersed into the reaction solvent mixture via ultrasonication. **AuNP-nuc** and an aliquot of the TFA stock solution were then added and the mixture again subjected to ultrasonication. The mixture was stirred at 35 °C for 1 week. The coloured supernatant was collected via centrifugation at 14,800 rpm for 20 min and analysed by ^{19}F NMR spectroscopy. The solid residue was washed with DMF until colourless, indicating that excess isolated **AuNP-nuc** had been removed, followed by EtOH (collection by centrifugation at 14,800 rpm, 20 min each time). The assembly product was kept in solution until further analysis.

Table S19 Molecular and nanoparticle stoichiometries used in forming heteromaterial assemblies of **UiO-66-elec-4F** and **AuNP-nuc.**

Exp.	Total volume / mL	Mass **UiO-66-elec-4F** / mg	[**UiO-66-elec-4F**] / μM	[**1**] / mM	Mass **AuNP-nuc** / mg	[**AuNP-nuc**] / μM	[**S16**] / mM	NP molar ratio (**NP-nuc** : **NP-elec**)	Molecular molar ratio (**S16**:**1**)
**A**	2.5	0.2	0.045	0.028	2	0.64	0.3	14:1	11:1
**B**	18.75	15	0.45	0.25	15	0.64	0.3	1.4:1	1.2:1

Analysis of supernatants from UiO-66_Au heteromaterial assembly

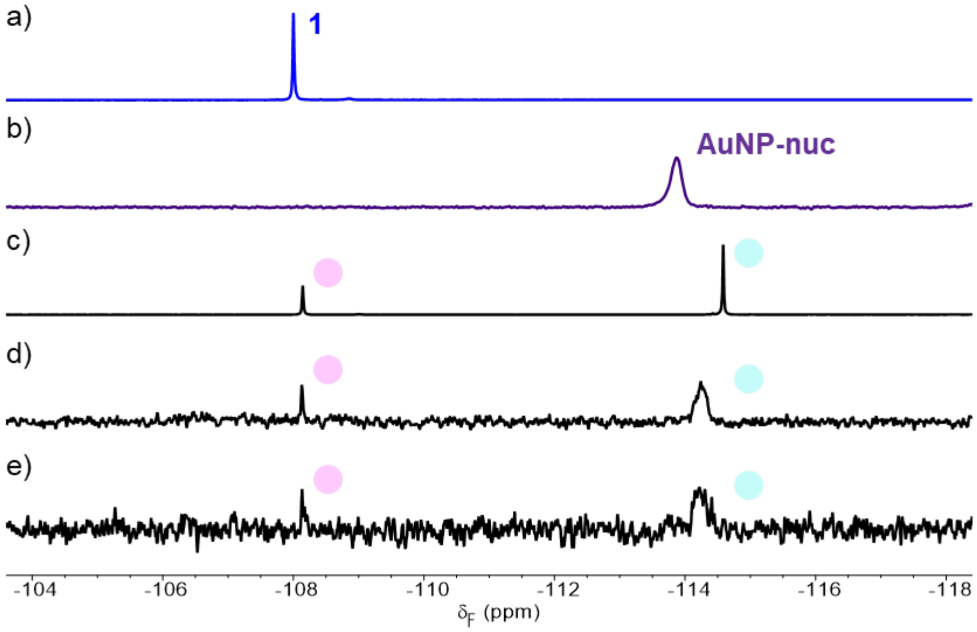

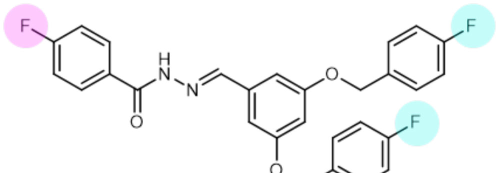

7

Figure S91 ^{19}F NMR spectra (376.7 MHz) of i) authentic hydrazone **1** (10% v/v D_2O/DMF), ii) authentic hydrazone **AuNP-nuc** (Au–**S16**, CD_2Cl_2), iii) authentic hydrazone **7** (10% v/v D_2O/DMF), iv) supernatant after heteromaterial formation (experiment **A**) (10% v/v D_2O/DMF), v) aliquot of supernatant after heteromaterial formation (experiment **B**) (10% v/v D_2O/DMF).

22.2 Characterisation UiO-66_Au heteromaterials

*Electron microscopy analysis – experiment **A** (Au:UiO-66 14:1)*

One week after combining a 14-fold excess of **AuNP-nuc** with **UiO-66-elec-4F** (corresponding to an approximate ratio of hydrazones **S16**:**1** of 11:1), TEM imaging (Figure S92) and EDX mapping (Figure S93) confirmed the formation of planet–satellite structures where **AuNP-nuc** decorated the surface of **UiO-66-elec-4F** with minimal excess **AuNP-nuc** present in the washed sample. The EDX elemental maps (Figure S93) also confirmed that hydrazones were still present on the external surfaces of the aggregates.

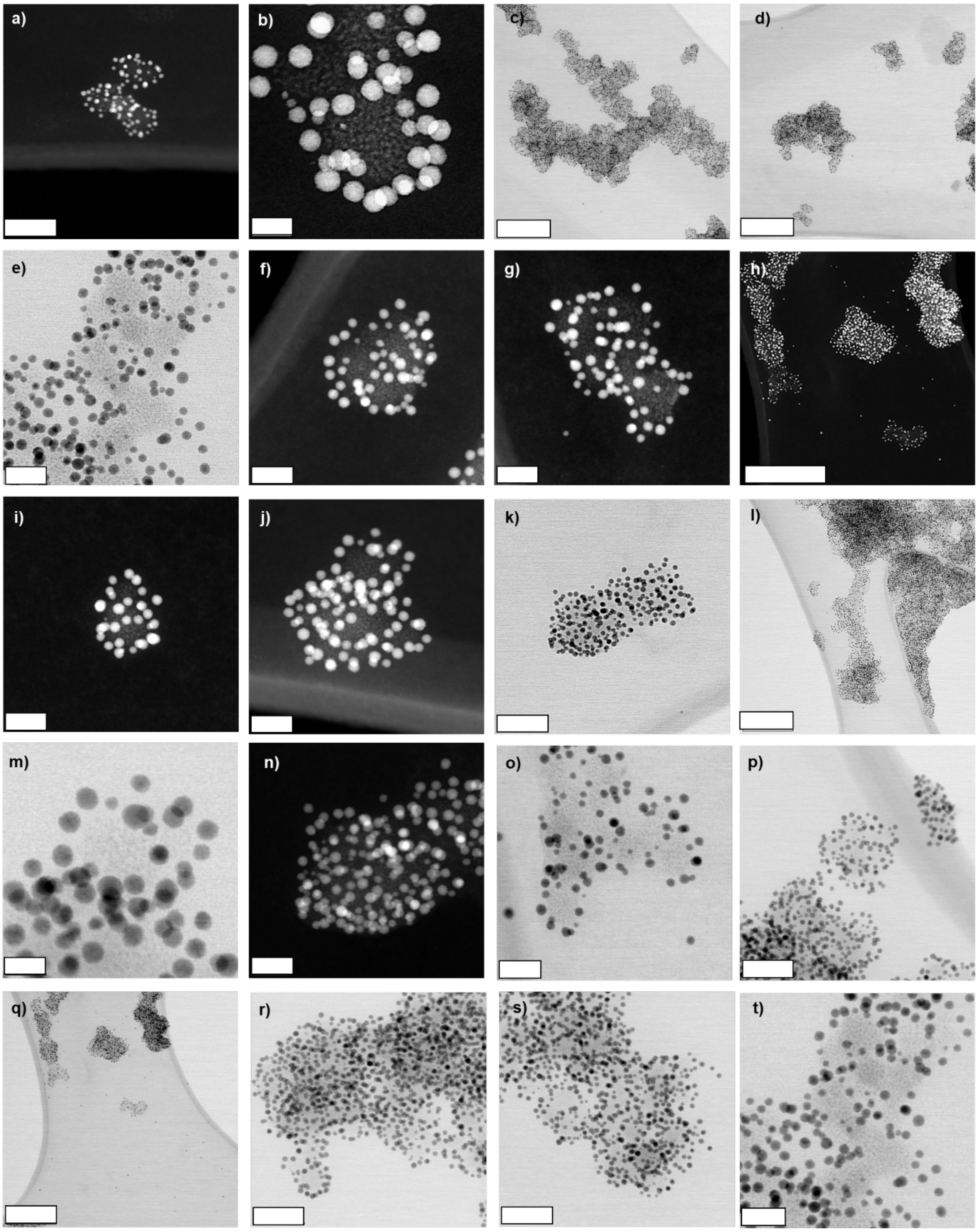

Figure S92 STEM-HAADF images of heteromaterials formed at **AuNP-nuc**:**UiO-66-elec-4F** 14:1 (experiment **A**). Scale bars: a) 50 nm; b) 10 nm; c) 200 nm; d) 200 nm; e) 20 nm; f) 20 nm; g) 20 nm; h) 200 nm; i) 20 nm; j) 20 nm; k) 50 nm; l) 200 nm; m) 10 nm; n) 20 nm; o) 20 nm; p) 50 nm; q) 200 nm; r) 50 nm; s) 50 nm; t) 20 nm.

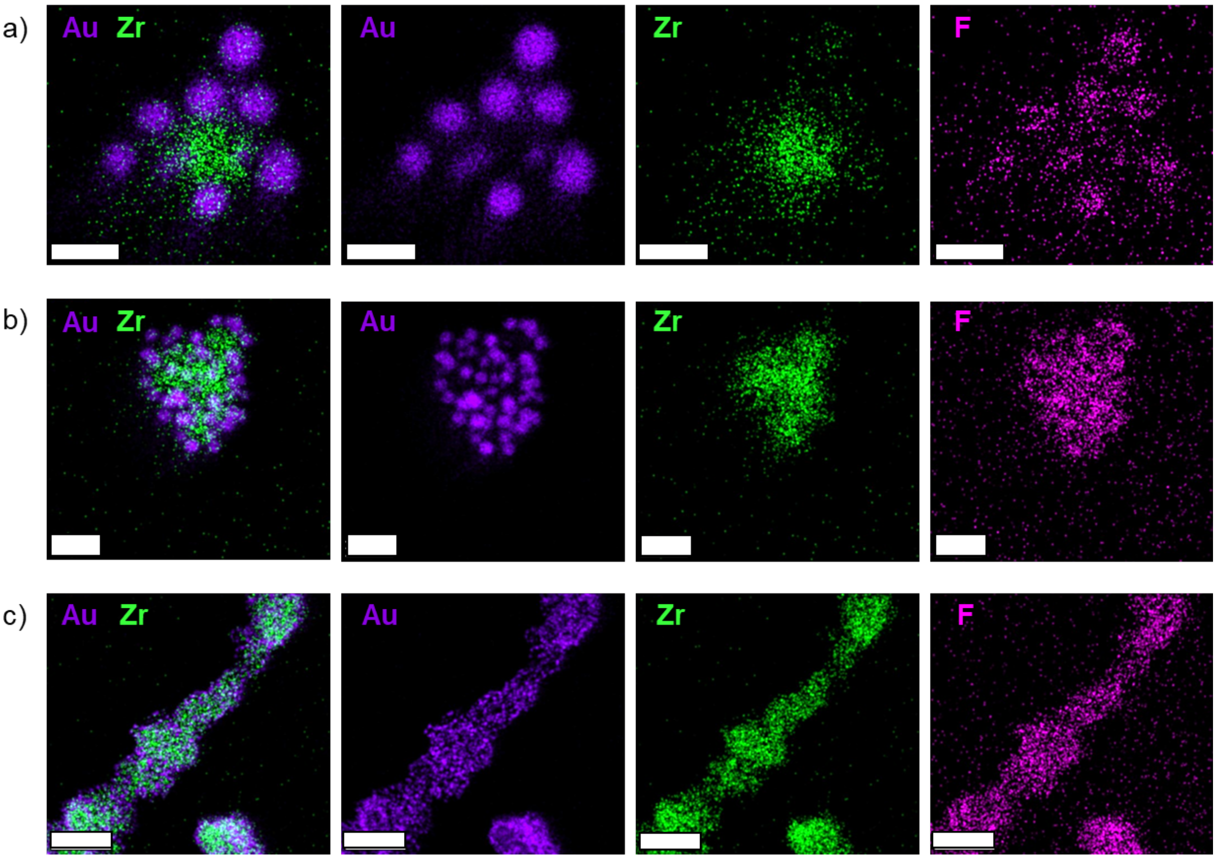

Figure S93 STEM-EDX of heteromaterials formed at **AuNP-nuc**:**UiO-66-elec-4F** 14:1 (experiment **A**). Green = Zr, purple = Au, pink = F. Scale bars: a) 10 nm; b) 20 nm; c) 100 nm.

*Electron microscopy analysis – experiment **B** (Au:UiO-66 1.4:1)*

One week after combining a 1.4-fold excess of **AuNP-nuc** with **UiO-66-elec-4F** (corresponding to an approximately equimolar ratio of hydrazones **S16**:**1** of 1.1:1), TEM imaging (Figure S94) indicated that the MOF NPs are more sparsely decorated with AuNPs compared to at higher stoichiometric excesses, resulting in a higher proportion of extended aggregates in which both AuNPs and MOF NPs link between more than one partner.

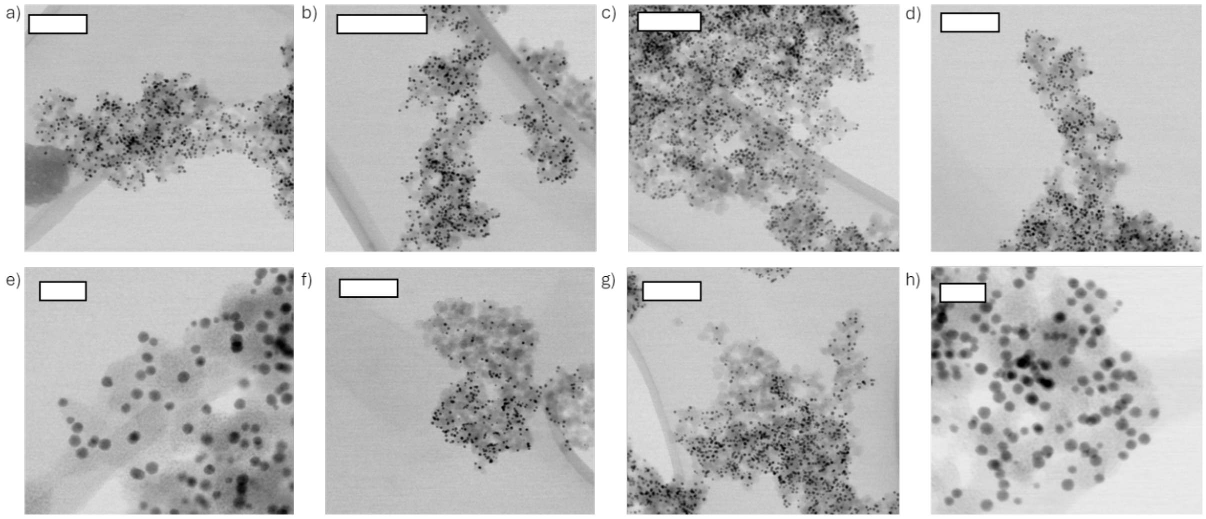

Figure S94 STEM-HAADF images of heteromaterials formed at **AuNP-nuc**:**UiO-66-elec-4F** 1.4:1 (experiment **B**). Scale bars: a)-d) 100 nm; e) 20 nm; f) 100 nm; g) 100 nm; h) 20 nm.

*Material characterisation of UiO-66_Au heteromaterial (experiment **B**)*

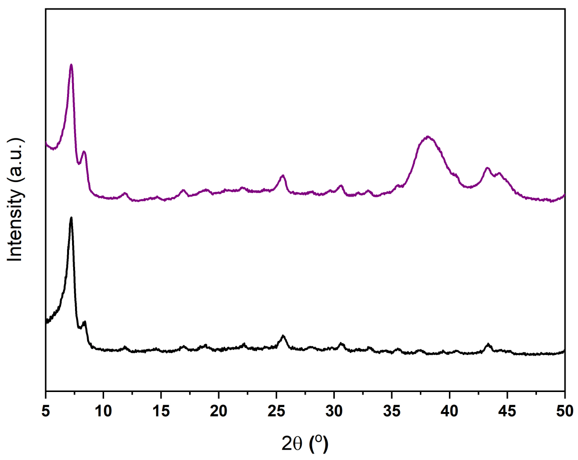

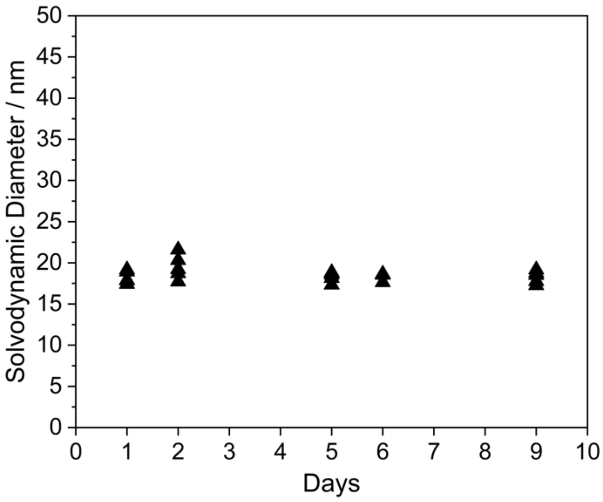

Figure S95 (Top) PXRD patterns of **UiO-66-elec-4F** (black, bottom) and **UiO-66_Au heteromaterial** (purple, top) and (Bottom) Variation of solvodynamic diameter with time measured by DLS of the dispersed **UiO-66_Au heteromaterial** showing that the suspensions are stable for at least nine days and show no aggregation.

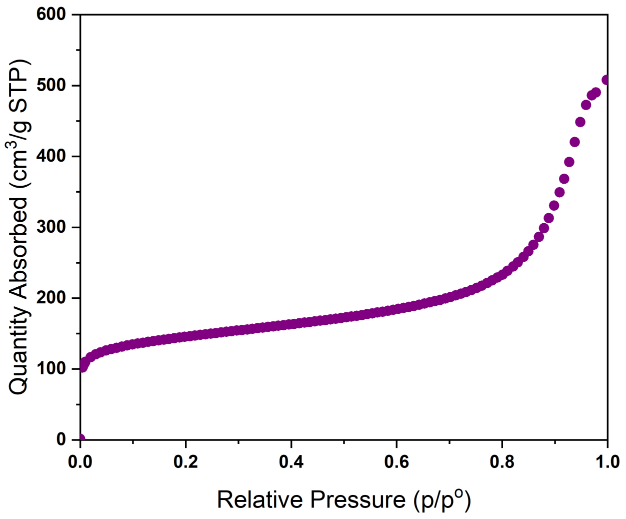

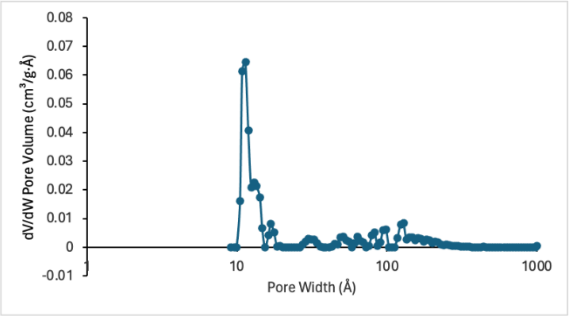

Figure S96 (Top) N_2 adsorption (77 K) isotherm of **UiO-66_Au heteromaterial** after activation at 150 °C for 16 hours and (bottom) the pore size distribution (NLDFT carbon slit pores) based on these adsorption data.

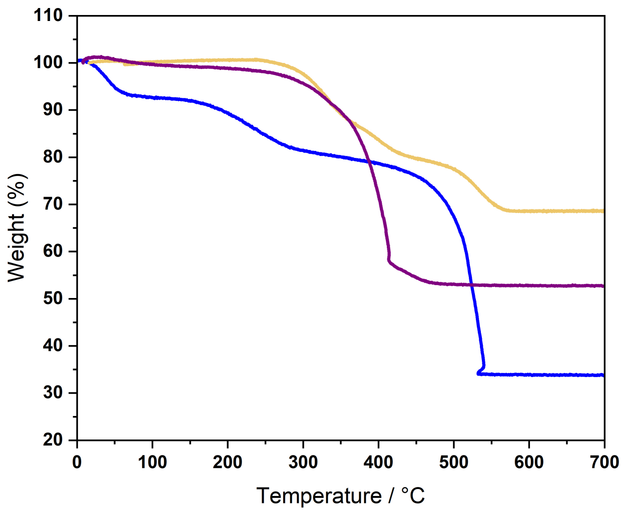

Figure S97 TGA plots of **AuNP-nuc** (yellow), **UiO-66_Au heteromaterial** (purple) and **UiO-66-elec-4F** (blue).

22.3 Heteromaterial assembly control – non-complementary building blocks

Two building blocks both with nucleophilic surface functionality were combined, following the general procedure described in Section 22.1: **UiO-66-nuc-4F** (0.2 mg, ~0.024 mM in terms of **2**) and **AuNP-nuc** (2 mg, ~0.3 mM in terms of **S16**).

Imaging by TEM (Figure S98) revealed no well-defined heteromaterial assemblies. Instead, the two different nanoscale building blocks tend to self-sort into spatially distinct clusters of each component, with AuNP aggregates appearing to deposit on top of MOF NP aggregates during drying on the TEM grid.

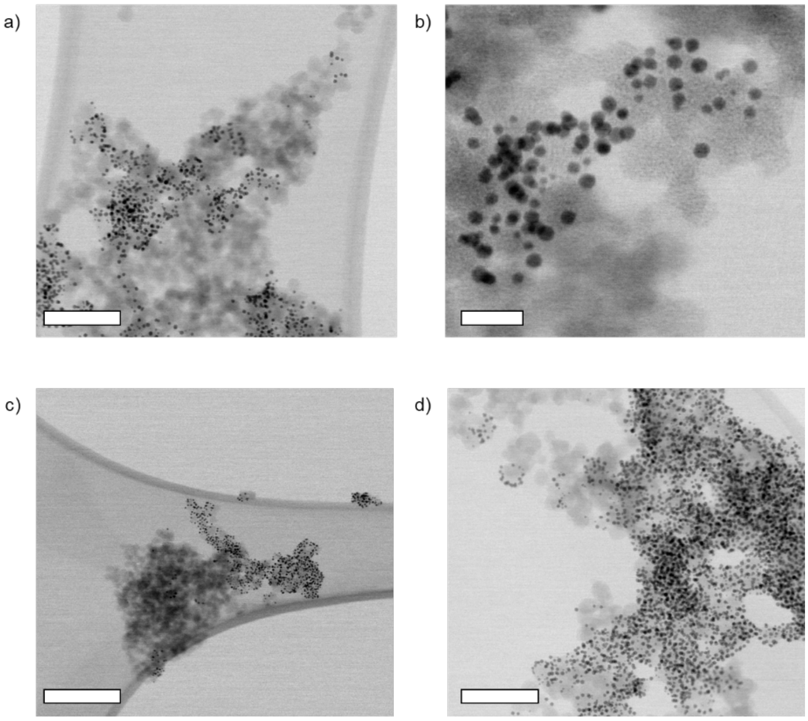

Figure S98 STEM images of heteromaterial control: **UiO-66-nuc-4F** and **AuNP-nuc**. Scale bars: a) 100 nm; b) 20 nm; c) 200 nm; d) 100 nm

22.4 Al-fum_Au heteromaterial

The general procedure described in Section 22.1 was adapted, replacing UiO-66-elec-4F with **Al-fum-elec-4F**, which was combined with AuNP-nuc at the stoichiometries shown in Table S20. After recovery by centrifugation, the solid product was washed with DMF followed by EtOH (collection by centrifugation at 14,800 rpm, 20 min each time). The assembly product was kept in solution until further analysis.

Table S20 Molecular stoichiometries used in forming heteromaterial assembly of **Al-fum-elec-4F** and **AuNP-nuc.**

Total volume / mL	Mass **Al-fum-elec-4F** / mg	[**1**] / mM [a]	Mass **AuNP-nuc** / mg	[**AuNP-nuc**] / μM	[**S16**] / mM	Molecular molar ratio (**S16**:**1**)
1.25	1.0	0.048	1.0	0.64	0.30	6.3:1

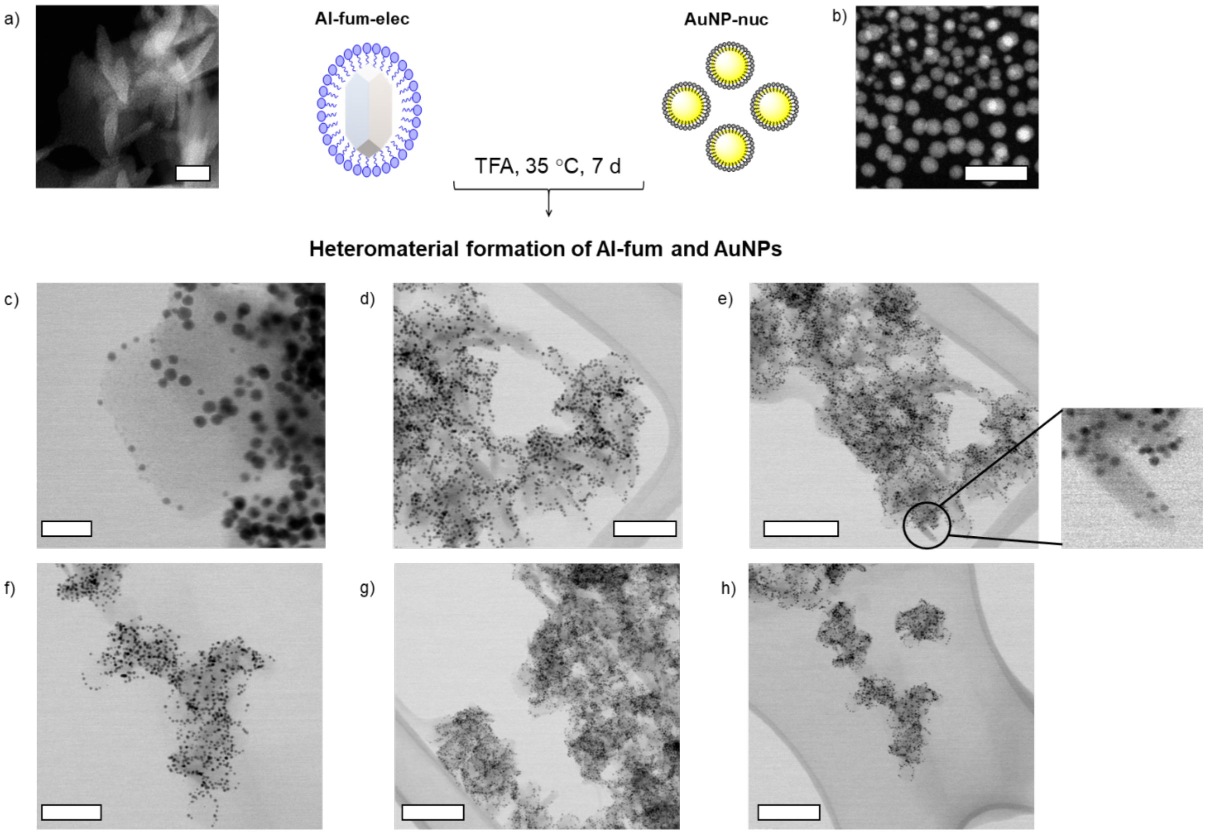

Figure S99 a) STEM-HAADF image of **Al-fum-elec-4F**: scale bar = 20 nm; b) TEM image of **AuNP-nuc**; scale bar = 20 nm. c)-h) STEM-HAADF images of **Al-fum_Au** heteromaterial, scale bars: c) 20 nm; d) 100 nm; e) 200 nm, with close up image of individual Al-fum NP decorated with AuNP at the edges; f) 100 nm; g) 200 nm; h) 200 nm.

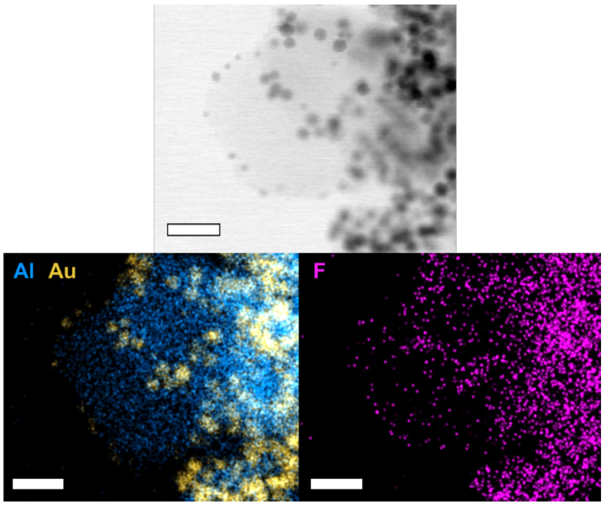

Figure S100 STEM-EDX of **Al-fum_Au** heteromaterial. Blue = Al, gold = Au, pink = F. Scale bar: 20 nm.

23. ^{1}H, ^{19}F and ^{13}C NMR spectra and mass spectra of organic compounds

23.1 Spectral data for 1

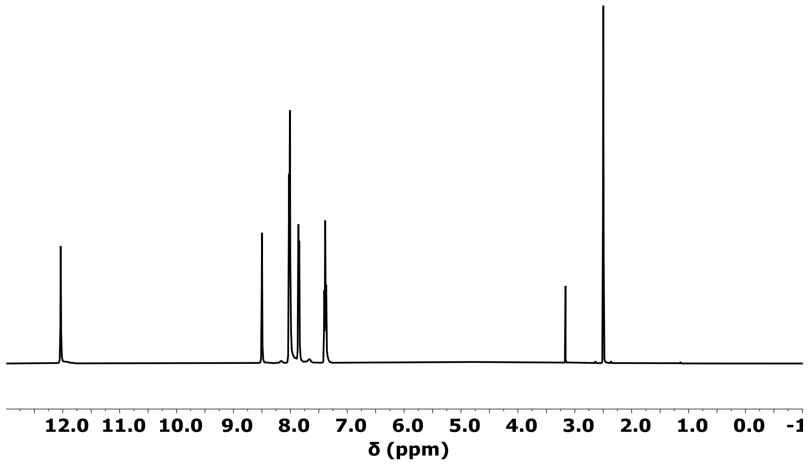

Figure S101 ^{1}H NMR spectrum (500.1 MHz, DMSO-d_6) of **1.**

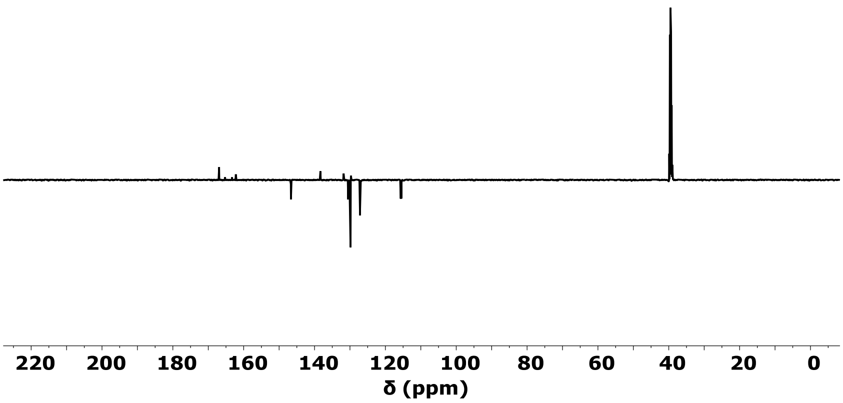

Figure S102 ^{13}C NMR spectrum (125.8 MHz, DMSO-d_6) of **1.**

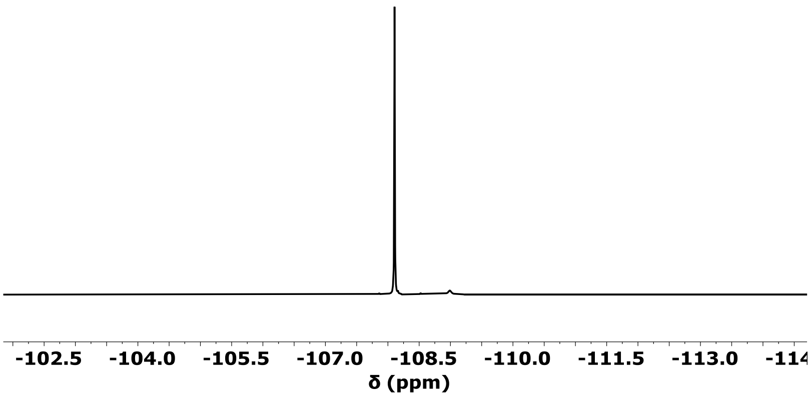

Figure S103 ^{19}F NMR spectrum (376.5 MHz, DMSO-d_6) of **1.**

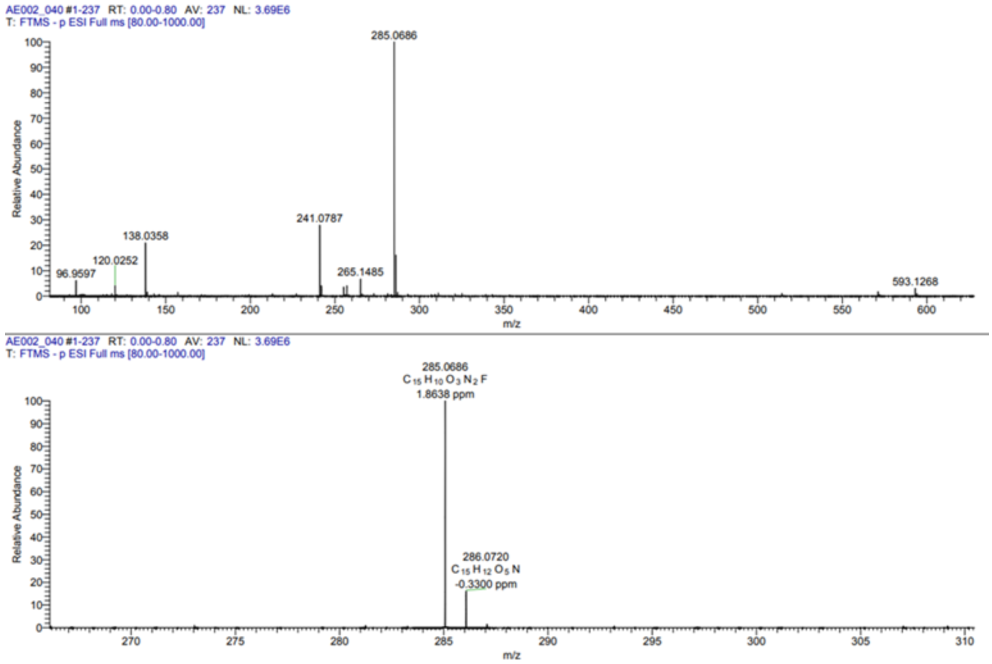

Figure S104 Mass spectrum of **1.**

23.2 Spectral data for 2

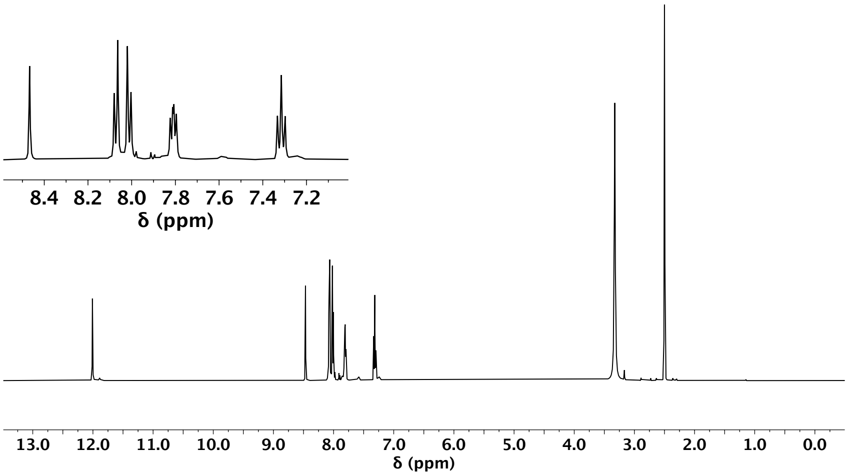

Figure S105 ^{1}H NMR Spectrum (499.9 MHz, DMSO-d_6) of **2.**

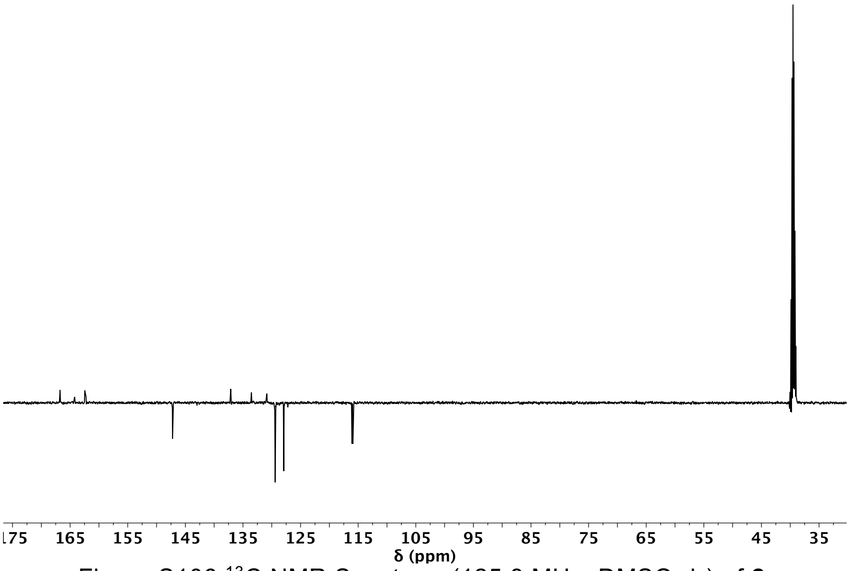

Figure S106 ^{13}C NMR Spectrum (125.8 MHz, DMSO-d_6) of **2.**

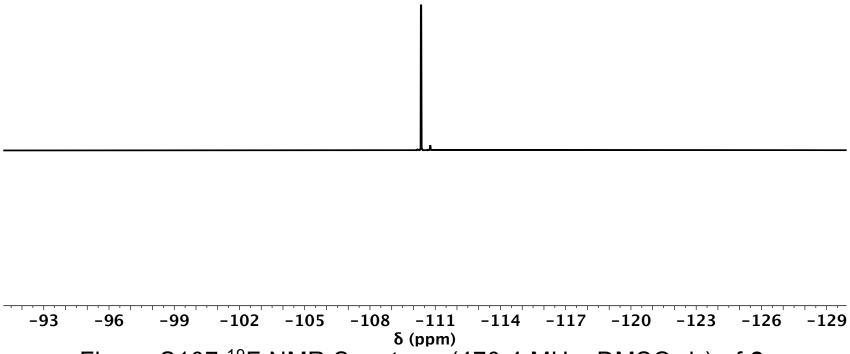

Figure S107 ^{19}F NMR Spectrum (470.4 MHz, DMSO-d_6) of **2.**

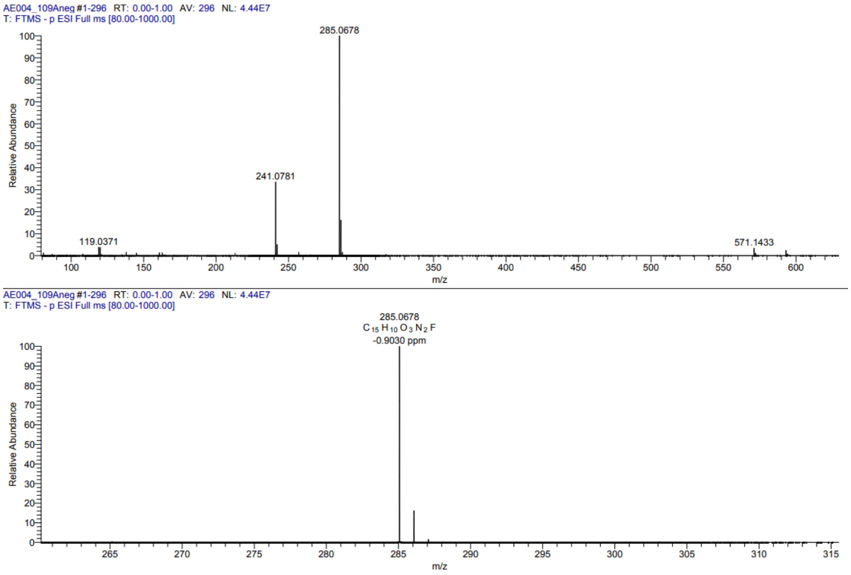

Figure S108 Mass spectrum of **2**.

23.3 Spectral data for S3

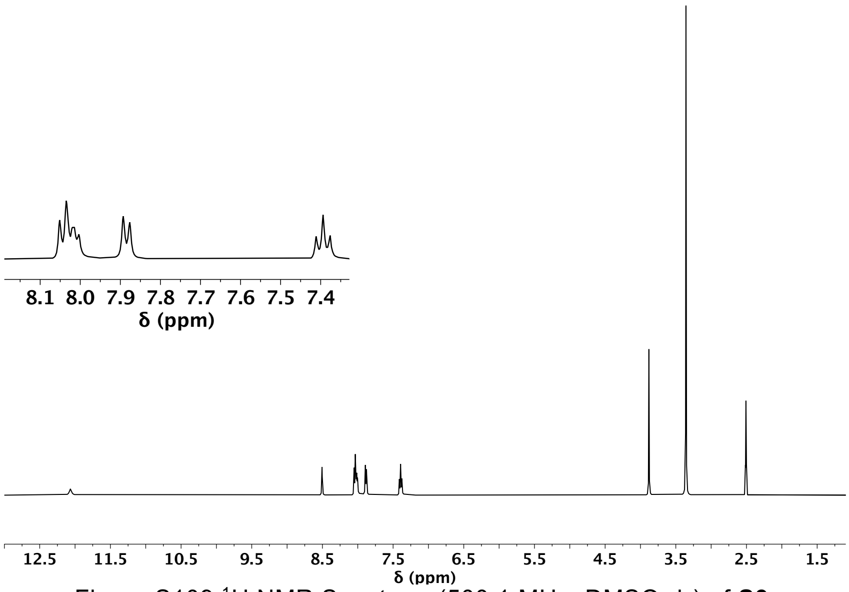

Figure S109 ^{1}H NMR Spectrum (500.1 MHz, DMSO-d_6) of **S3.**

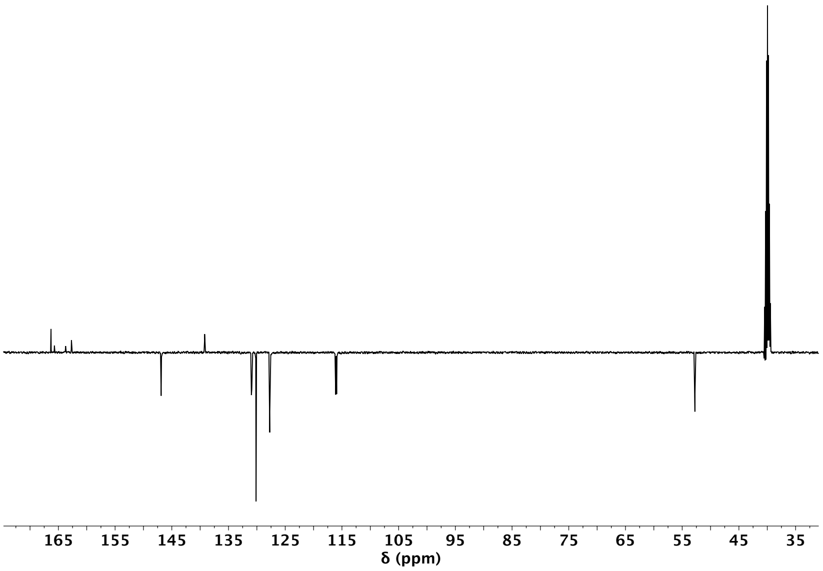

Figure S110 ^{13}C NMR Spectrum (125.8 MHz, DMSO-d_6) of **S3.**

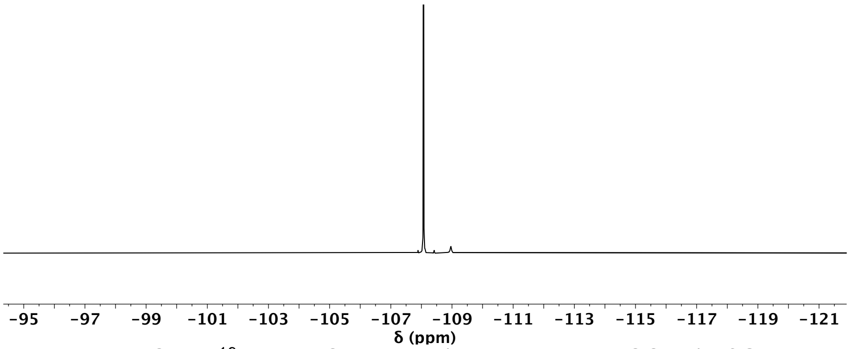

Figure S111 ^{19}F NMR Spectrum (470.4 MHz, DMSO-d_6) of **S3.**

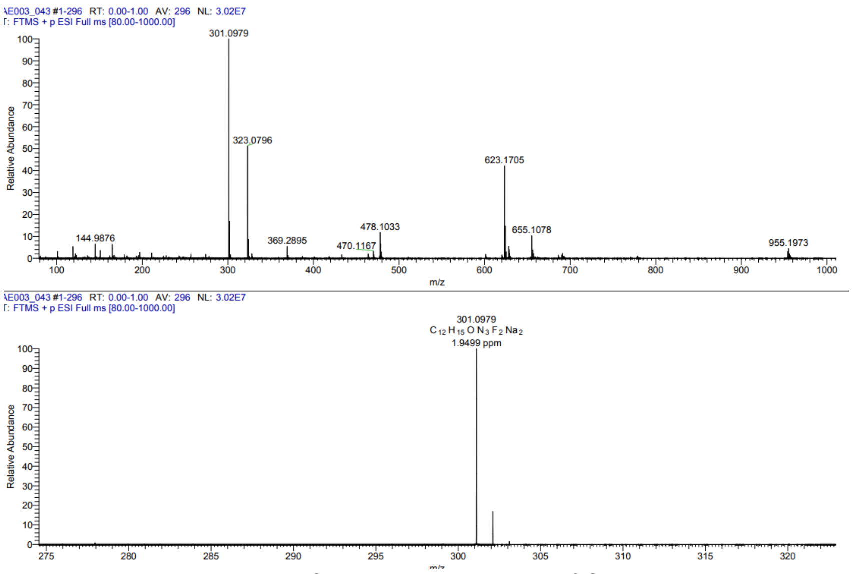

Figure S112 Mass spectrum of **S3**.

23.4 Spectral data for S4

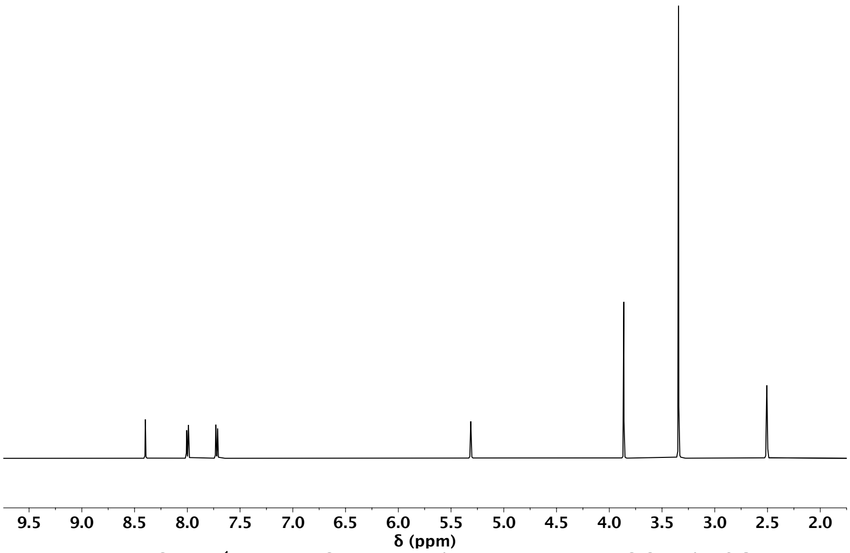

Figure S113 ¹H NMR Spectrum (500.1 MHz, DMSO-d_6) of **S4.**

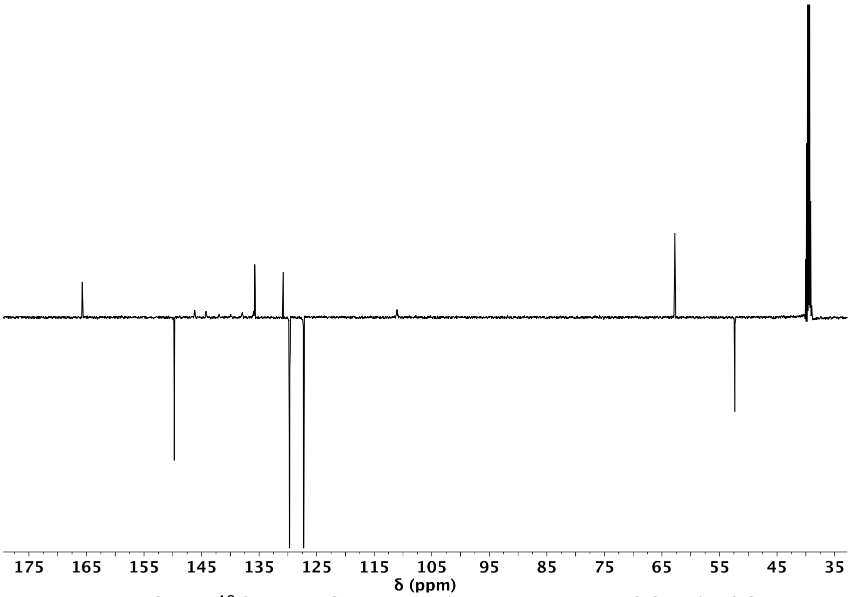

Figure S114 ^{13}C NMR Spectrum (125.8 MHz, DMSO-d_6) of **S4.**

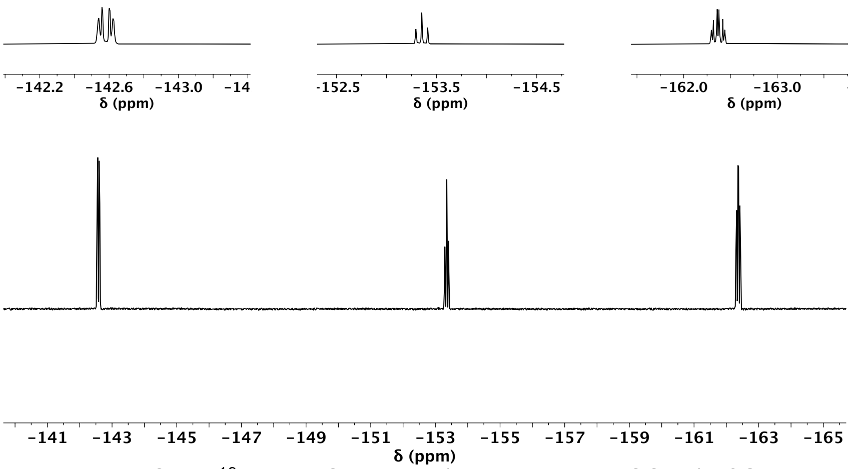

Figure S 115^{19}F NMR Spectrum (376.7 MHz, DMSO-d_6) of **S4.**

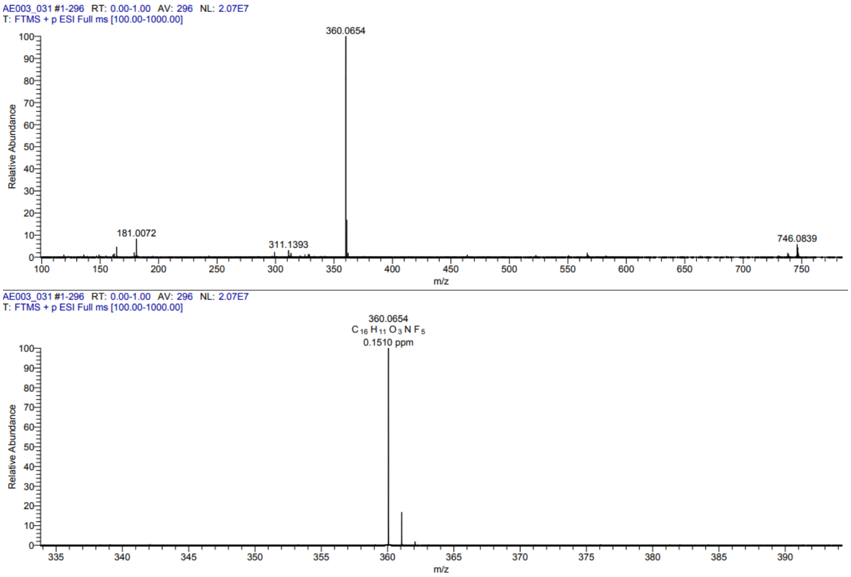

Figure S116 Mass spectrum of **S4**.

23.5 Spectral data for S5

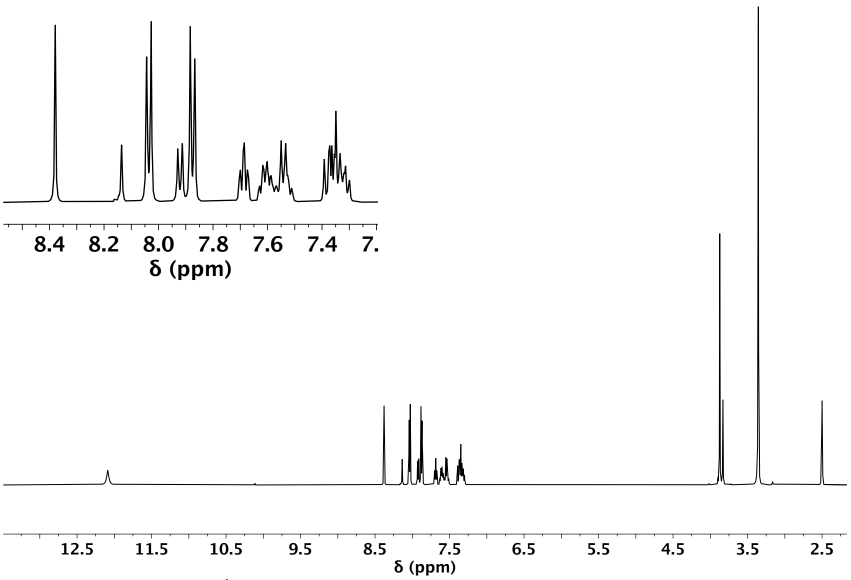

Figure S117 ^{1}H NMR Spectrum (499.9 MHz, DMSO-d_6) of **S5**.

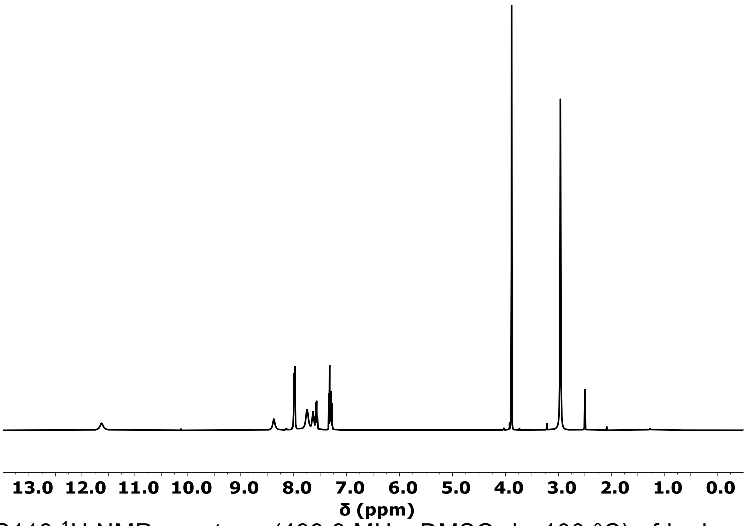

Figure S118 ^{1}H NMR spectrum (499.9 MHz, DMSO-d_6, 100 °C) of hydrazone **S5.**

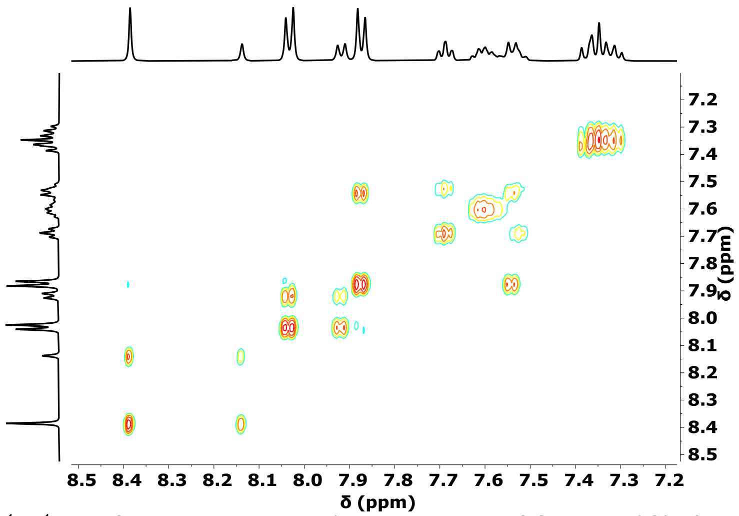

Figure S119 ^{1}H-^{1}H EXSY NMR spectrum (499.9 MHz, DMSO-d_6, 25 °C) of hydrazone **S5**.

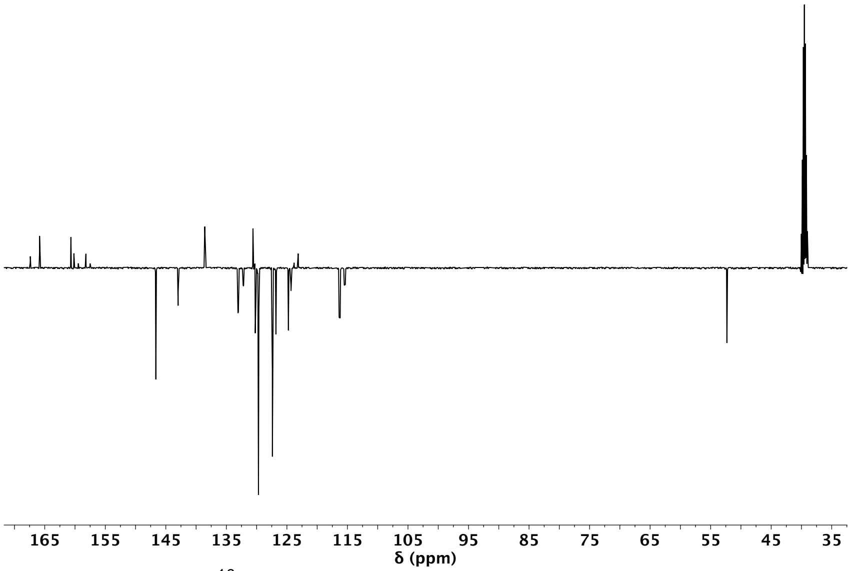

Figure S120 ^{13}C NMR Spectrum (125.8 MHz, DMSO-d_6) of **S5.**

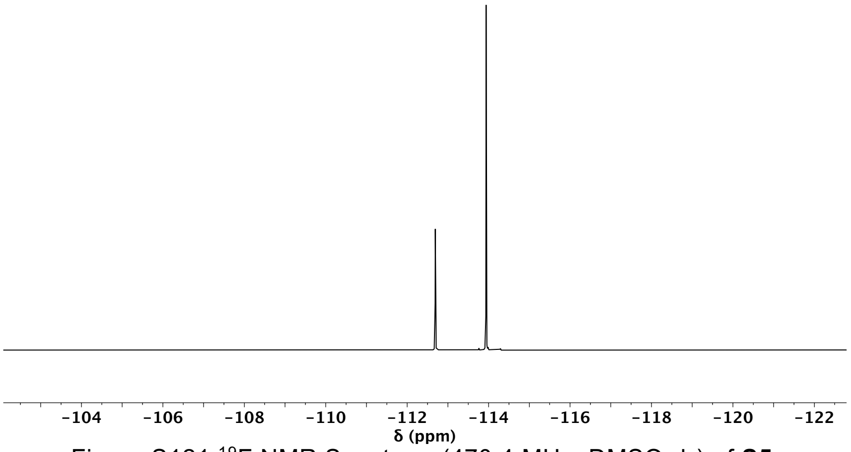

Figure S121 ^{19}F NMR Spectrum (470.4 MHz, DMSO-d_6) of **S5.**

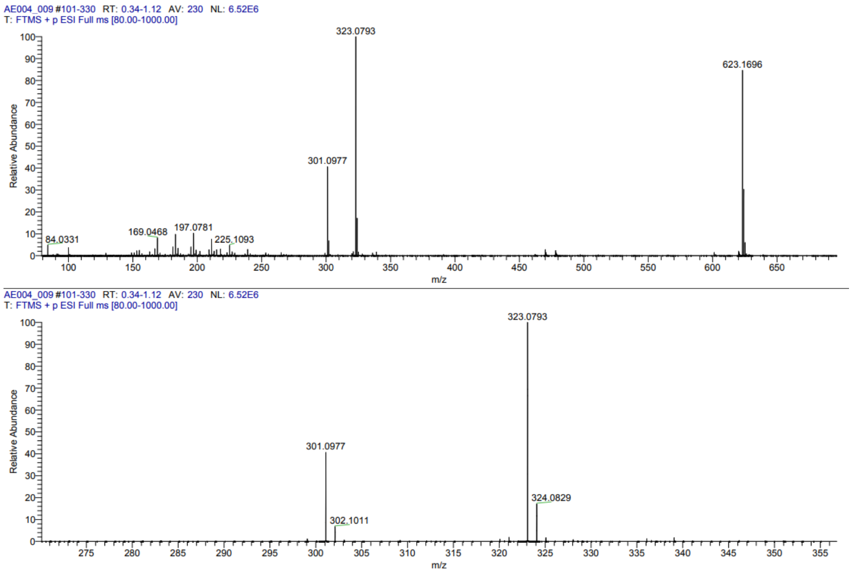

Figure S122 Mass spectrum for **S5**.

23.6 Spectral data for 3

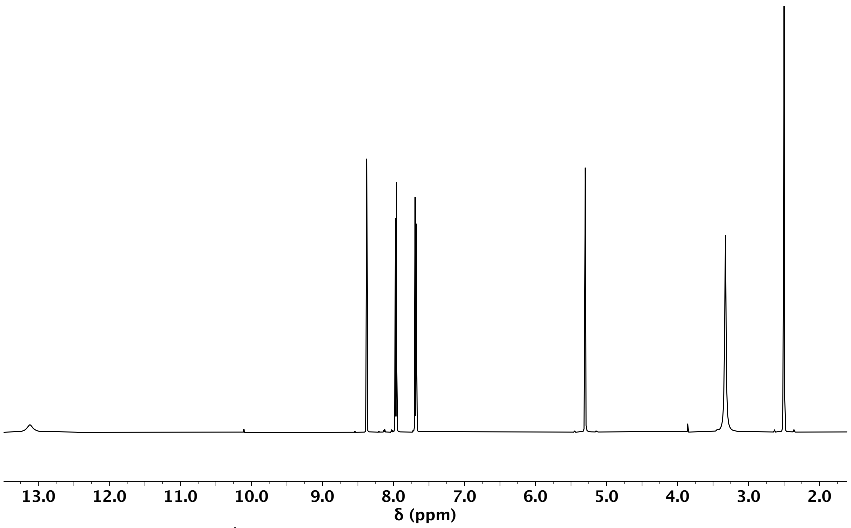

Figure S123 ^{1}H NMR Spectrum (499.9 MHz, DMSO-d_6) of **3.**

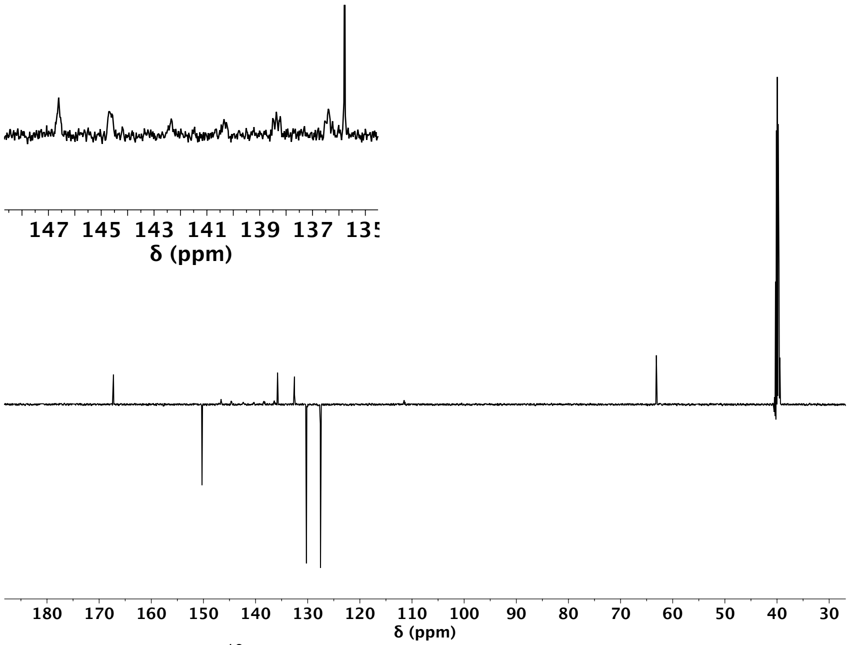

Figure S124 ^{13}C NMR Spectrum (125.8 MHz, DMSO-d_6) of **3.**

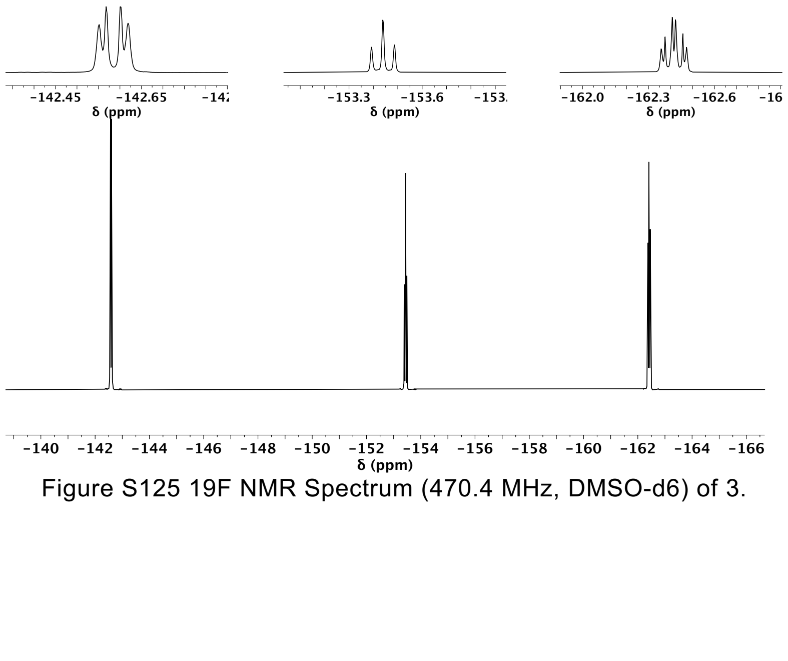

Figure S125 19F NMR Spectrum (470.4 MHz, DMSO-d6) of 3.

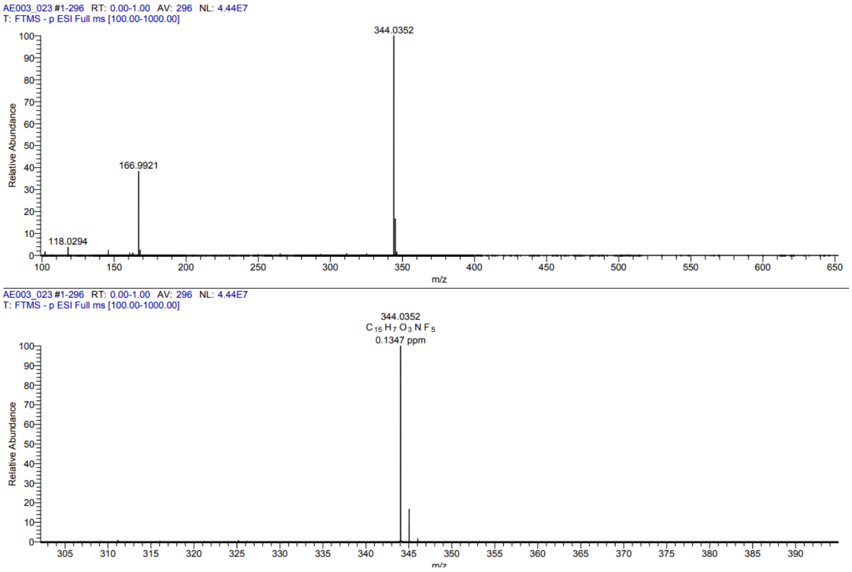

Figure S126 Mass spectrum of 3.

23.7 Spectral data for S6

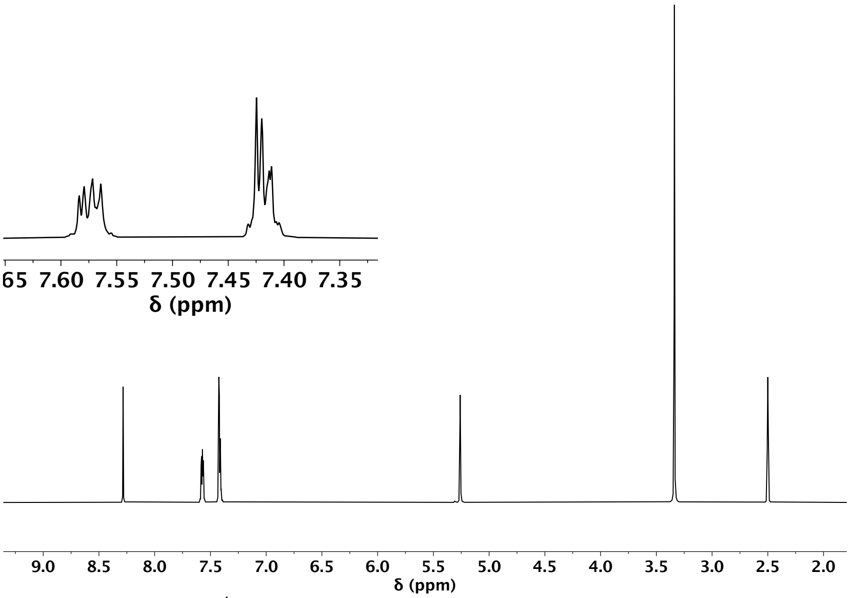

Figure S127 ^{1}H NMR Spectrum (500.1 MHz, DMSO-d_6) of **S6.**

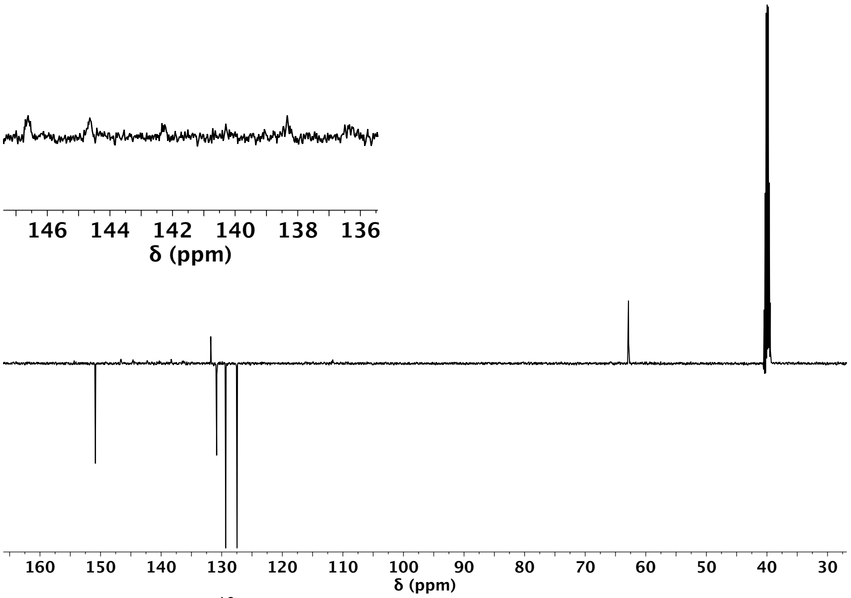

Figure S128 ^{13}C NMR Spectrum (125.8 MHz, DMSO-d_6) of **S6.**

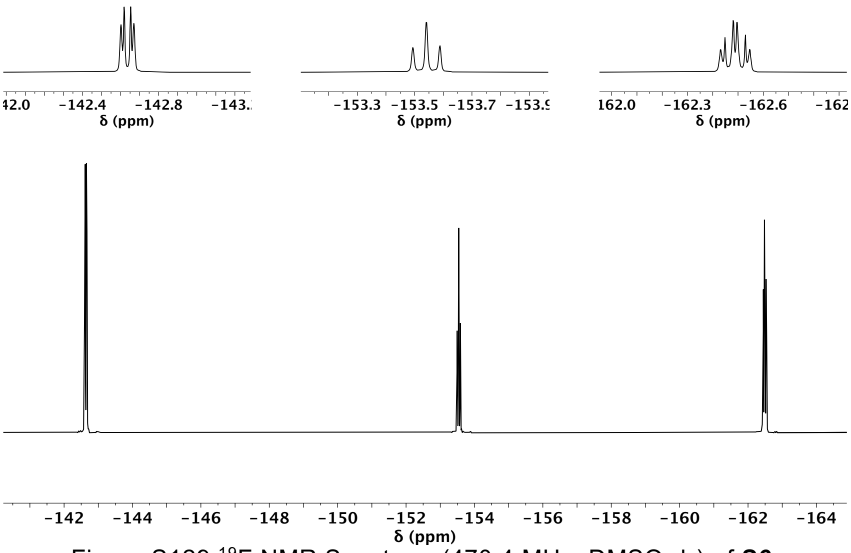

Figure S129 ^{19}F NMR Spectrum (470.4 MHz, DMSO-d_6) of **S6.**

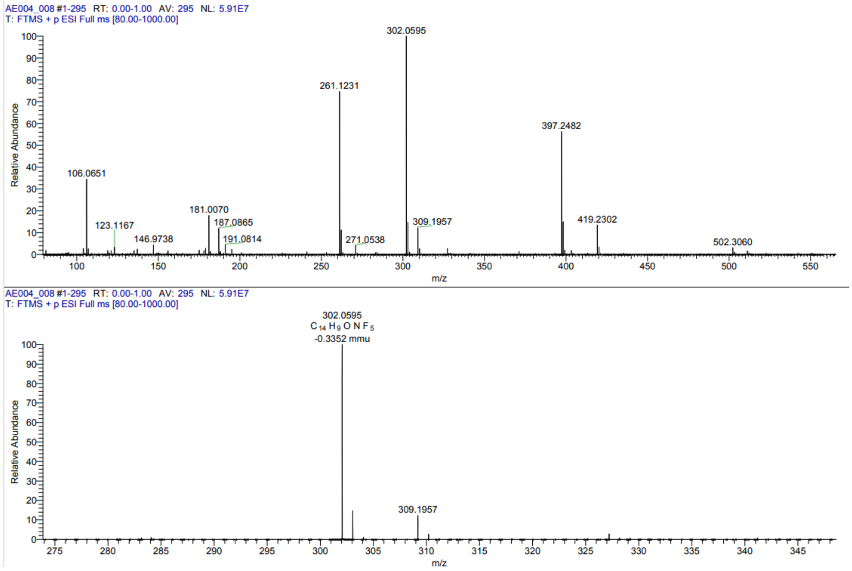

Figure S130 Mass spectrum of **S6**.

23.8 Spectral data for 4

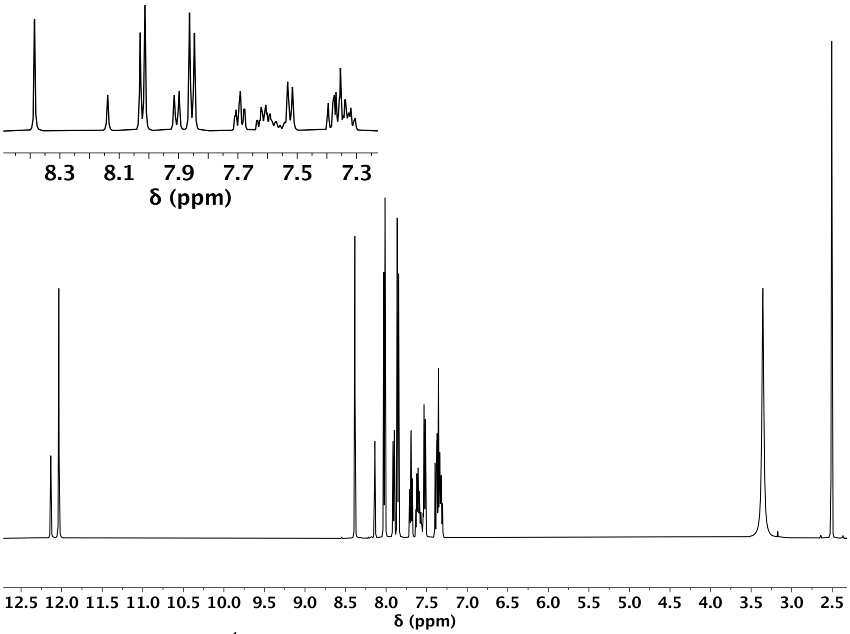

Figure S131 ^{1}H NMR Spectrum (500.1 MHz, DMSO-d_6) of **4.**

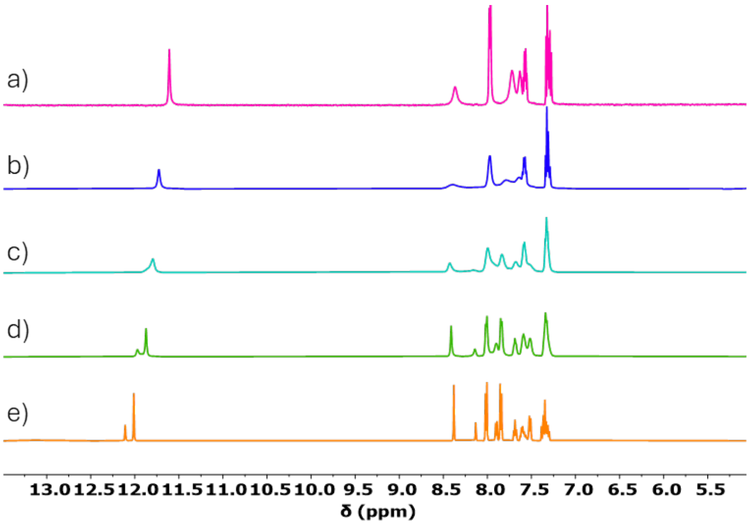

Figure S132 ^{1}H NMR spectrum (499.9 MHz, DMSO-d_6) of hydrazone **4** at a) 100 °C b) 80 °C, c) 65 °C, d) 50 °C and e) 25 °C.

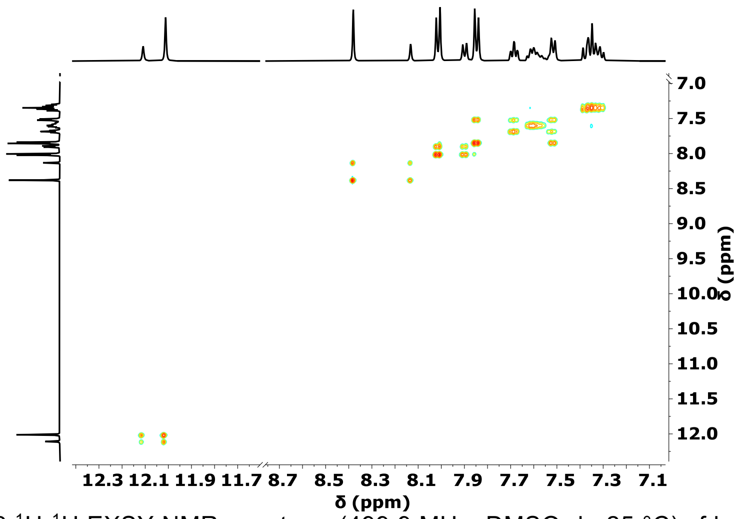

Figure S133 ^{1}H-^{1}H EXSY NMR spectrum (499.9 MHz, DMSO-d_6 ,25 °C) of hydrazone **4.**

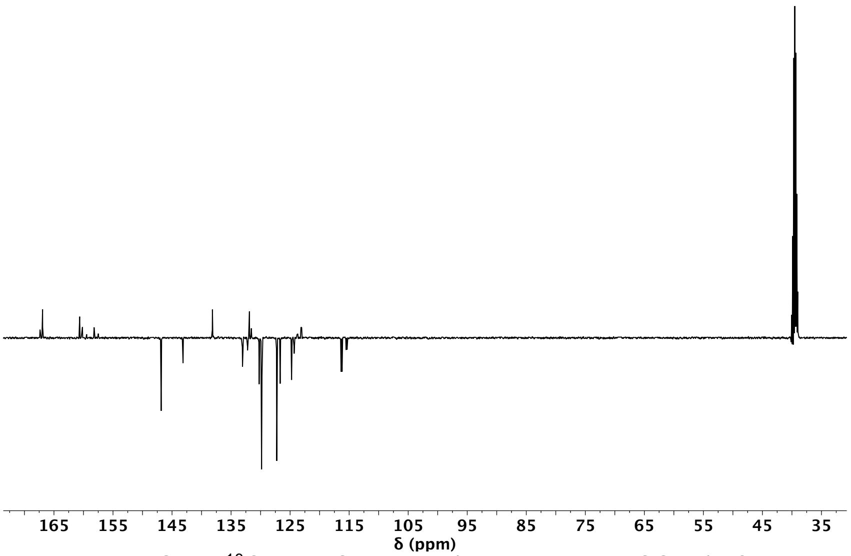

Figure S134 ^{13}C NMR Spectrum (125.8 MHz, DMSO-d_6) of **4.**

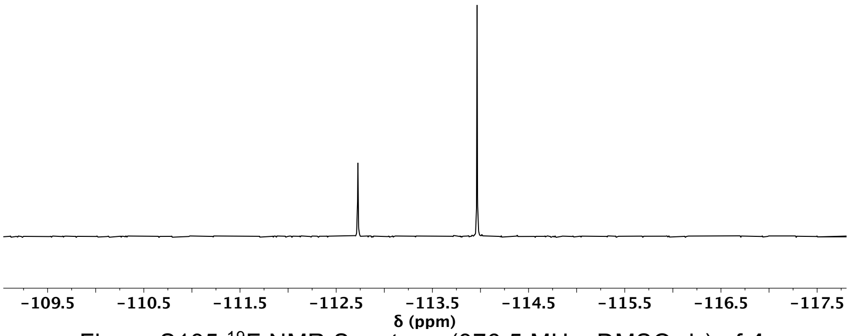

Figure S135 ^{19}F NMR Spectrum (376.5 MHz, DMSO-d_6) of **4.**

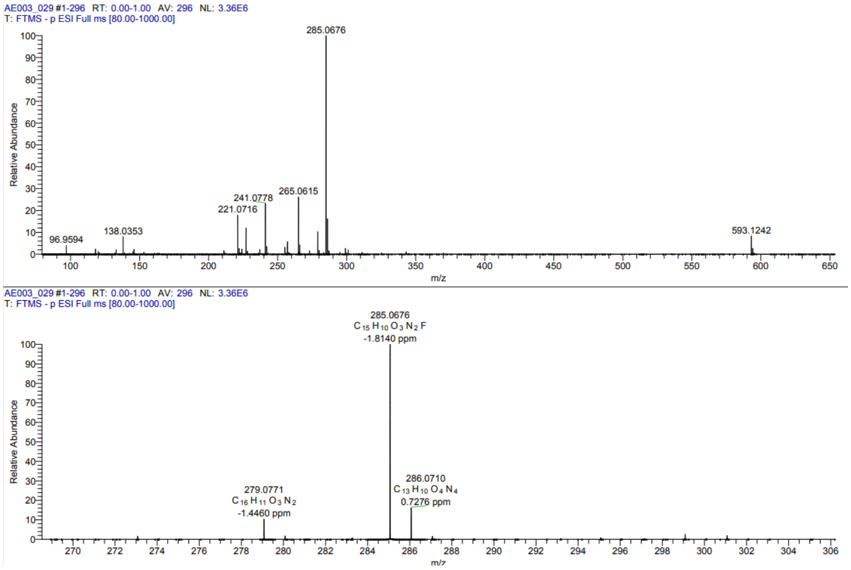

Figure S136 Mass spectrum of **4**.

23.9 Spectral data for S10

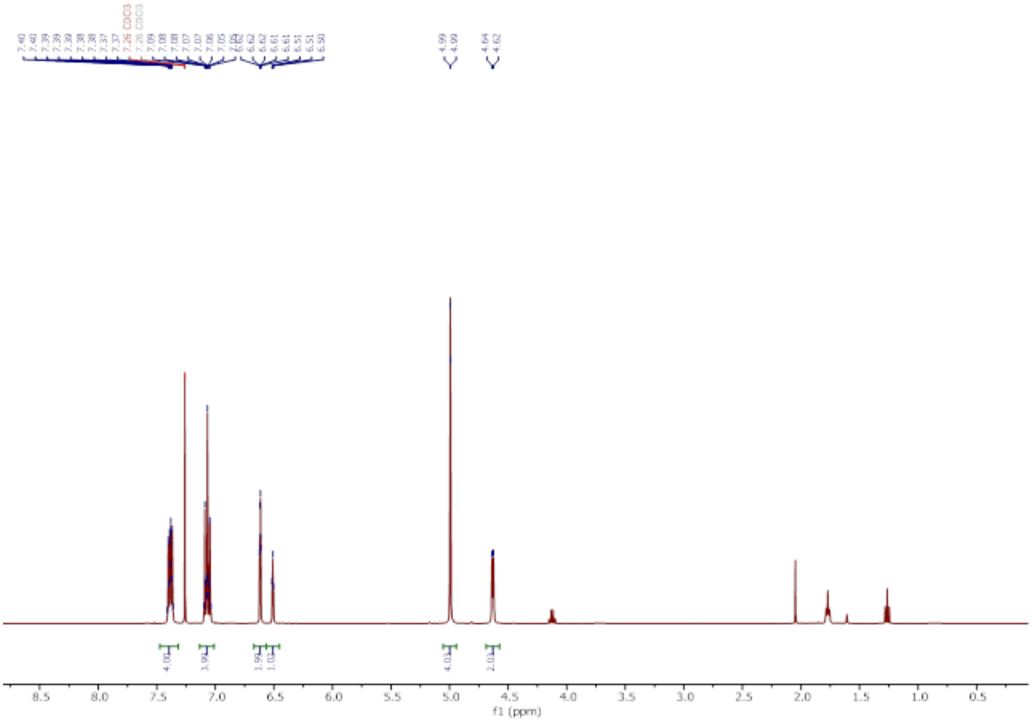

Figure S137 ^{1}H NMR Spectrum (400.1 MHz, $CDCl_3$) of **S10.**

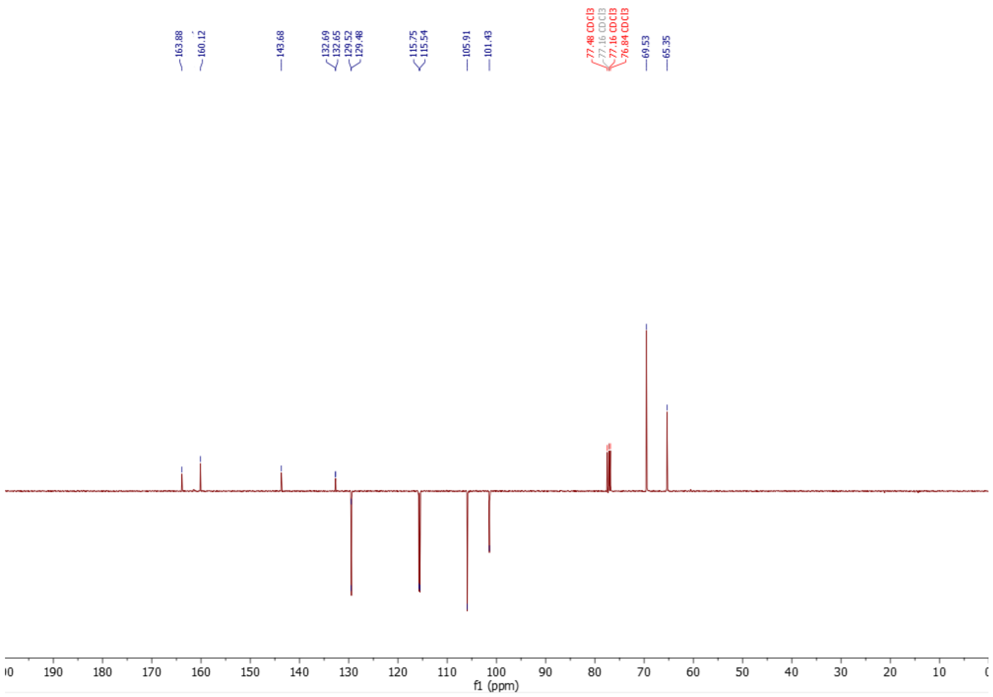

Figure S138 ^{13}C NMR Spectrum (100.6 MHz, $CDCl_3$) of **S10.**

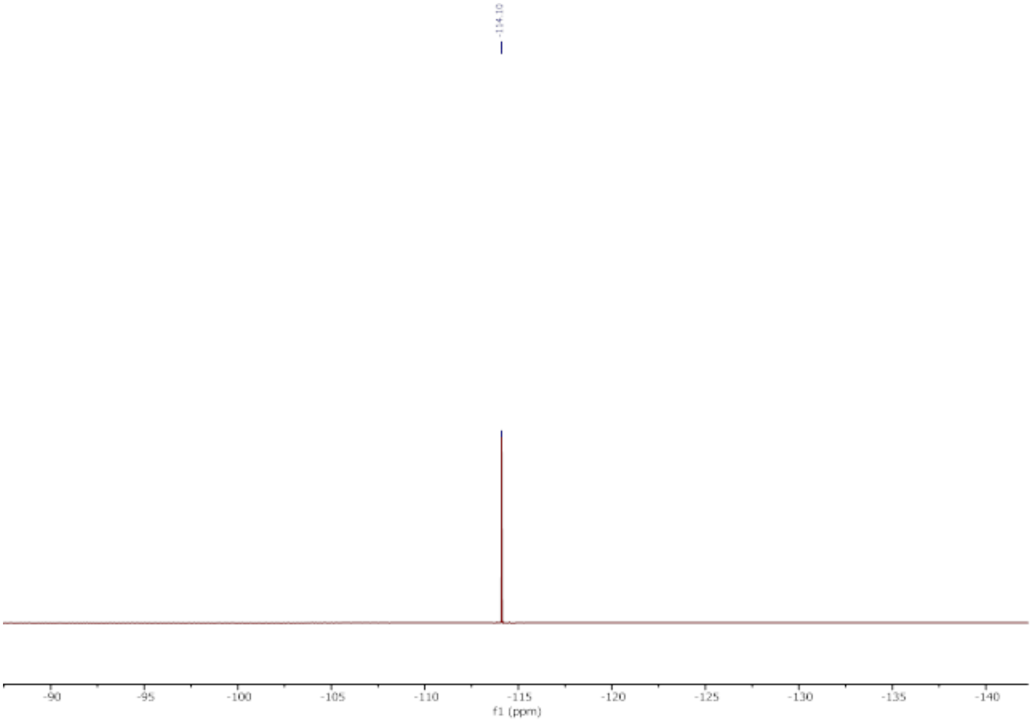

Figure S139 ^{19}F NMR Spectrum (376.5 MHz, $CDCl_3$) of **S10.**

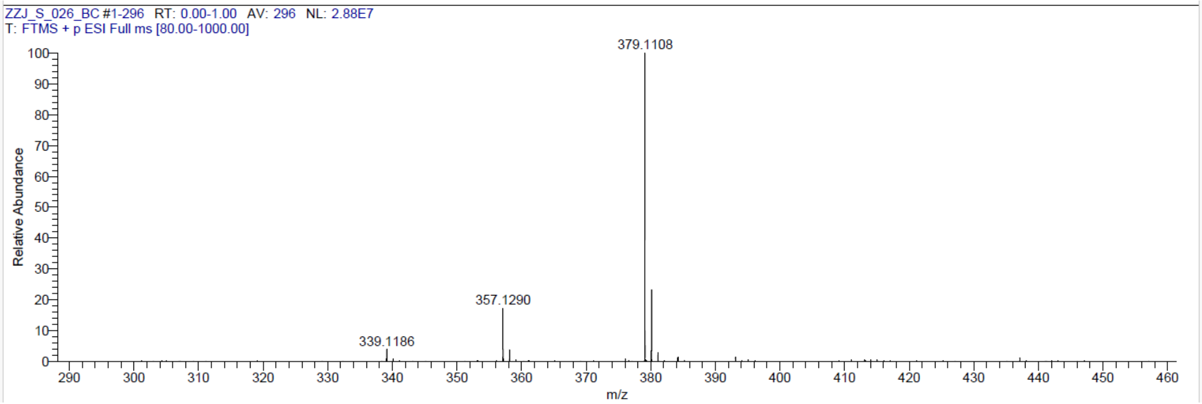

Figure S140 Mass spectrum of S10.

23.10 Spectral data for S11

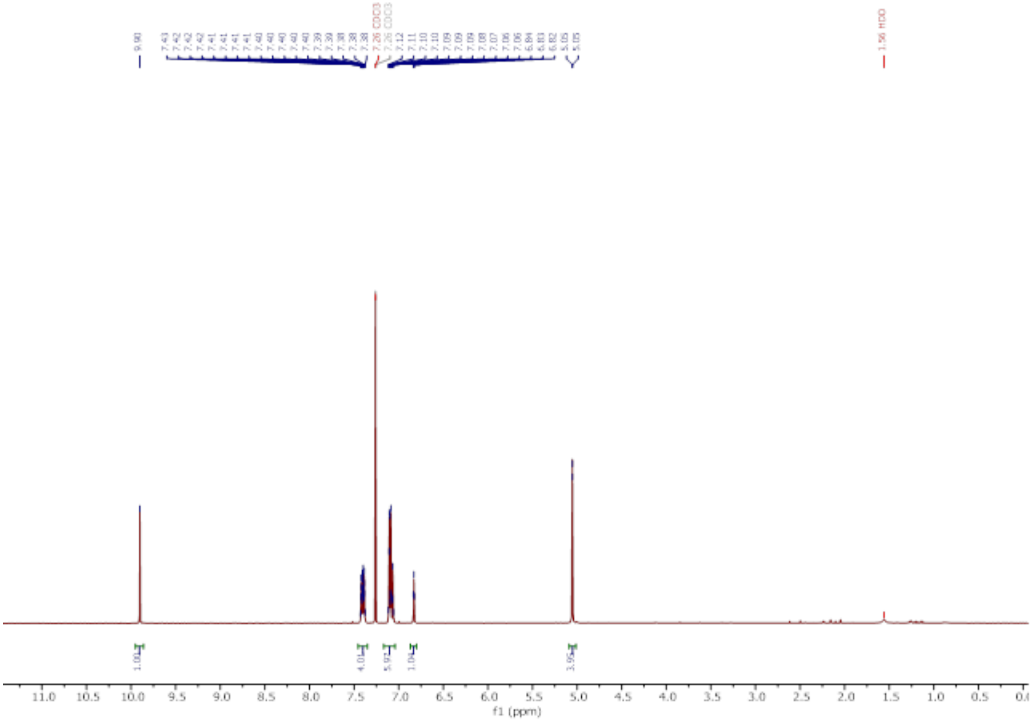

Figure S141 ^{1}H NMR Spectrum (400.1 MHz, $CDCl_3$) of **S11.**

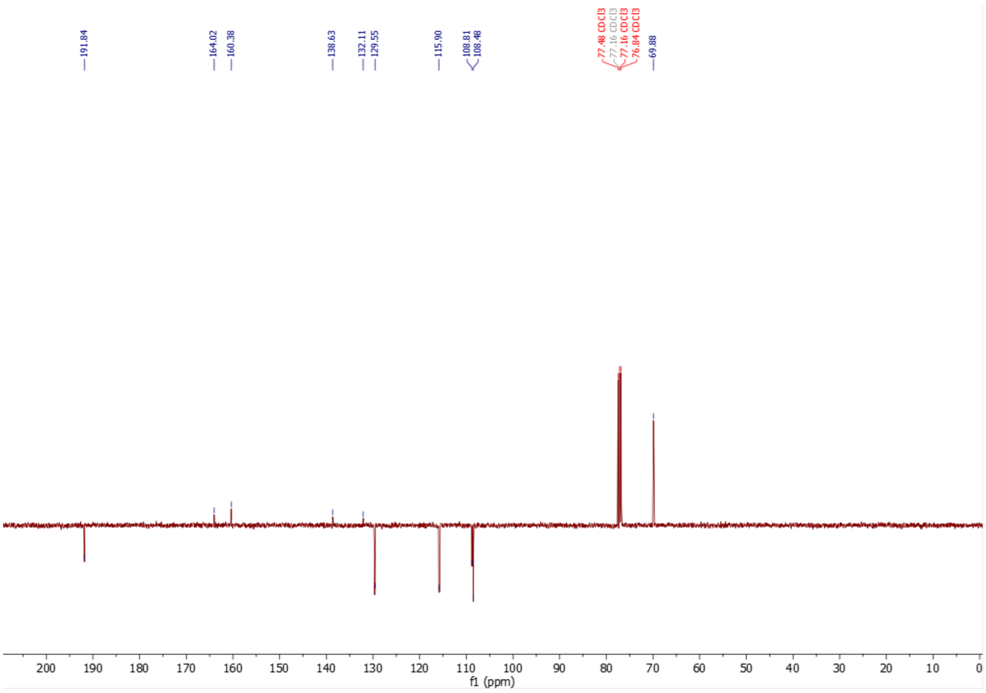

Figure S142 ^{13}C NMR Spectrum (100.6 MHz, $CDCl_3$) of **S11.**

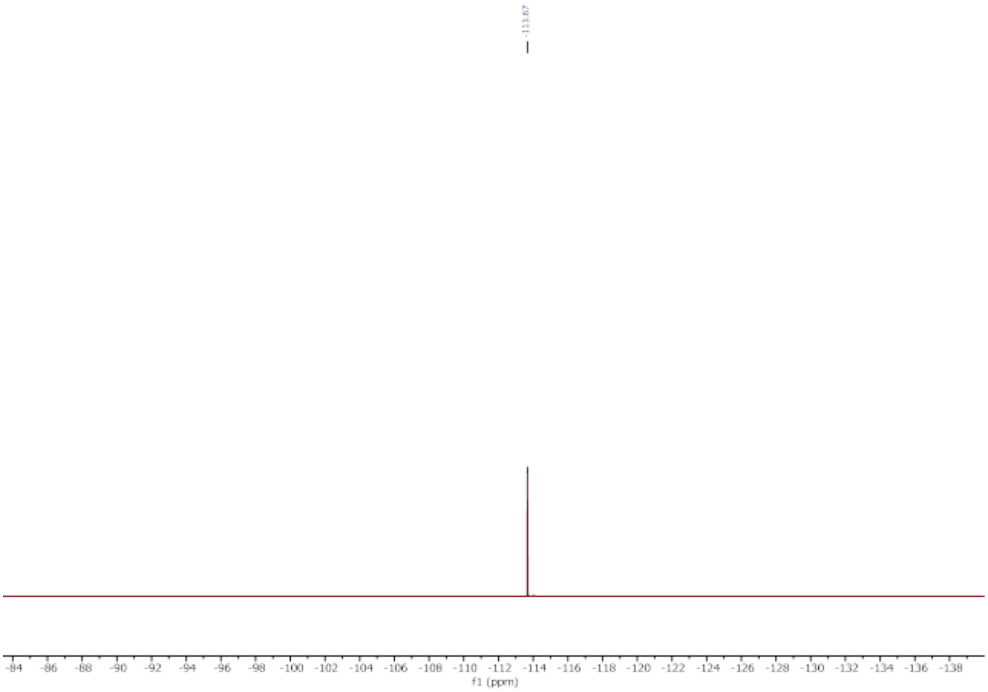

Figure S143 ^{1}H NMR Spectrum (376.5 MHz, $CDCl_3$) of **S11.**

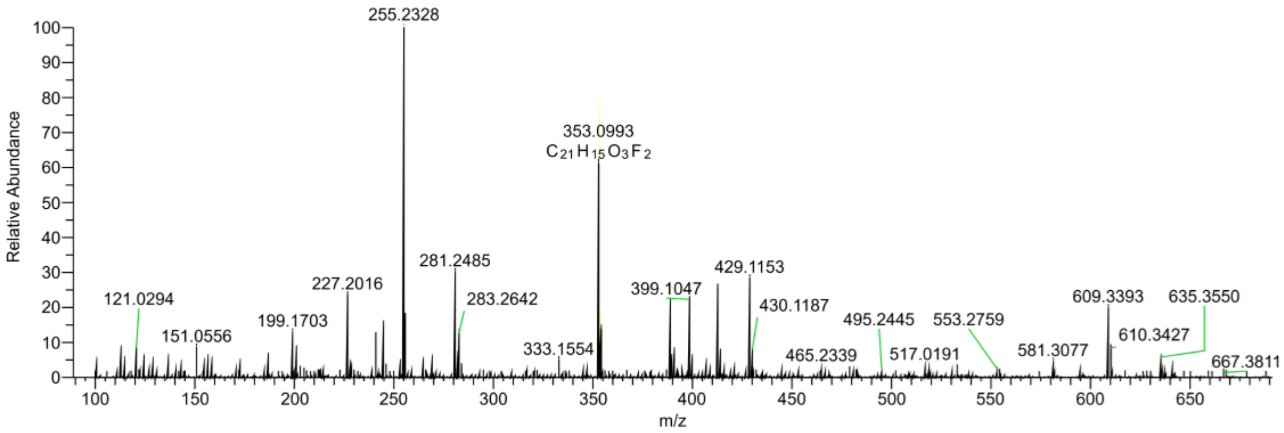

Figure S144 Mass spectrum of **S11.**

23.11 Spectral data for 5

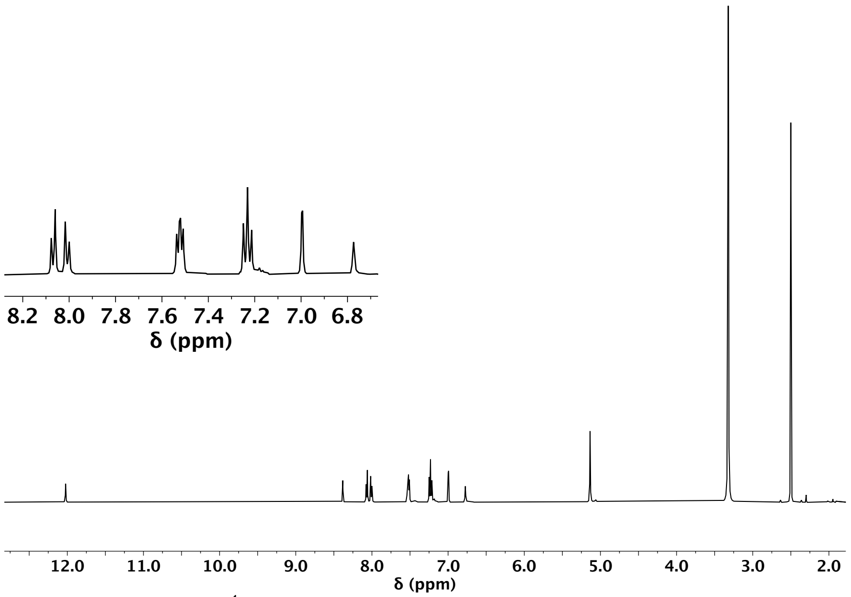

Figure S145 ^{1}H NMR Spectrum (499.9 MHz, DMSO-d_6) of **5.**

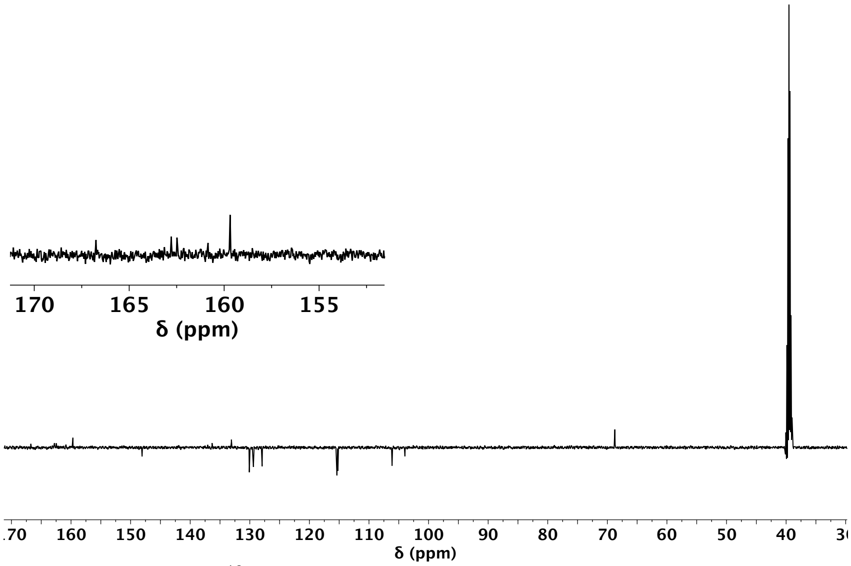

Figure S146 ^{13}C NMR Spectrum (125.8 MHz, DMSO-d_6) of **5.**

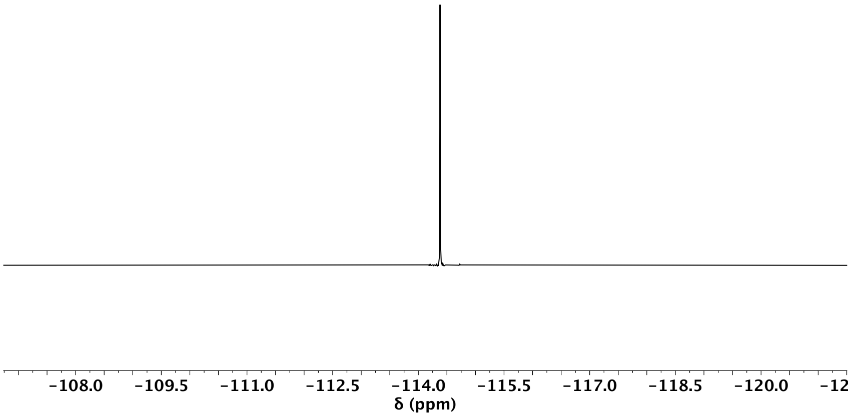

Figure S147 ^{19}F NMR Spectrum (470.4 MHz, DMSO-d_6) of **5.**

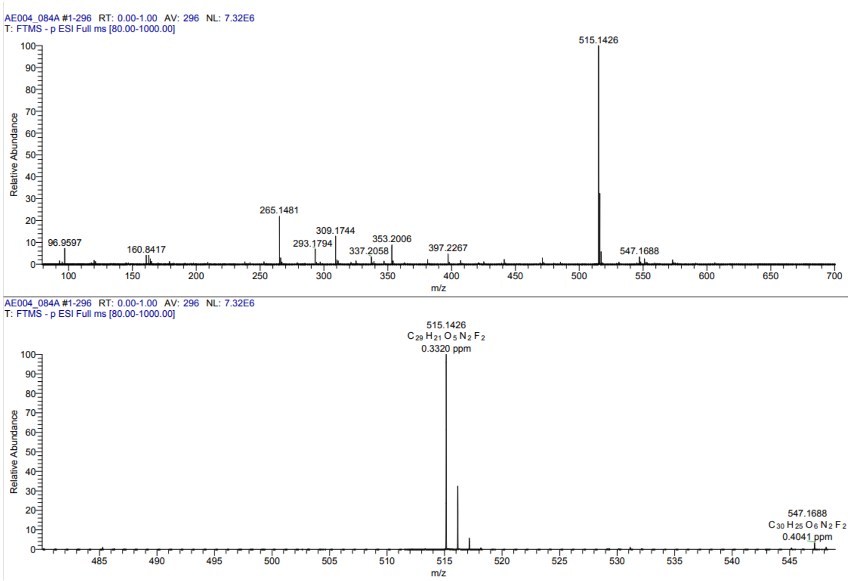

Figure S148 Mass spectrum of **5**.

23.12 Spectral data for 7

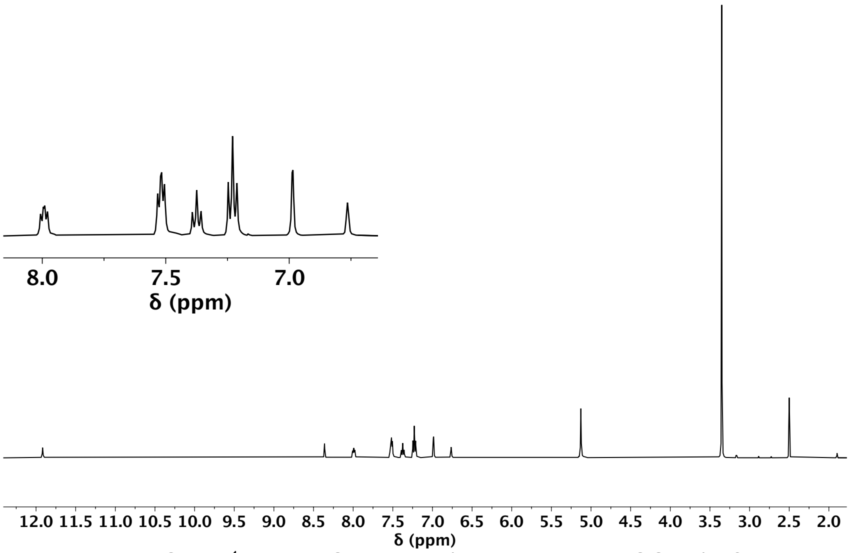

Figure S149 ^{1}H NMR Spectrum (500.1 MHz, DMSO-d_6) of **7.**

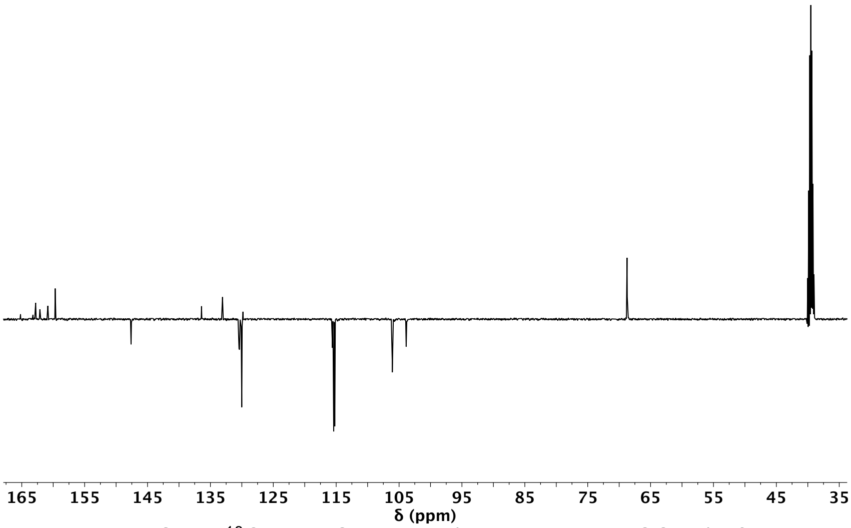

Figure S150 ^{13}C NMR Spectrum (125.8 MHz, DMSO-d_6) of **7.**

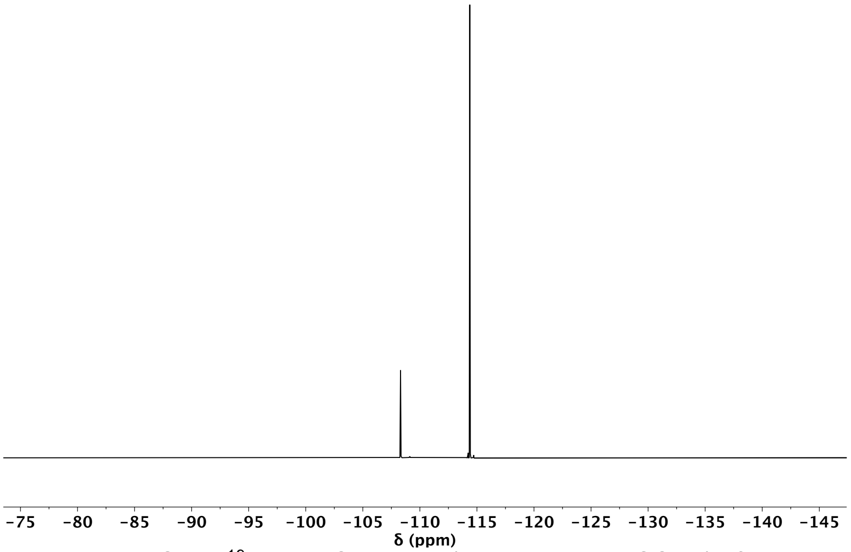

Figure S151 ^{19}F NMR Spectrum (470.4 MHz, DMSO-d_6) of **7.**

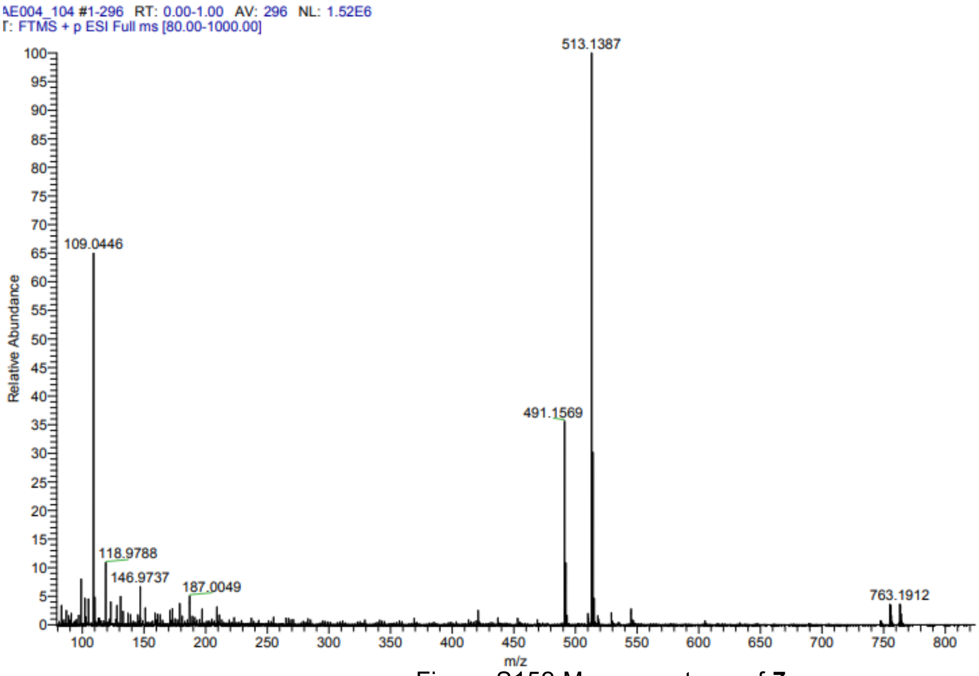

Figure S152 Mass spectrum of **7**.

23.13 Spectral data for S12

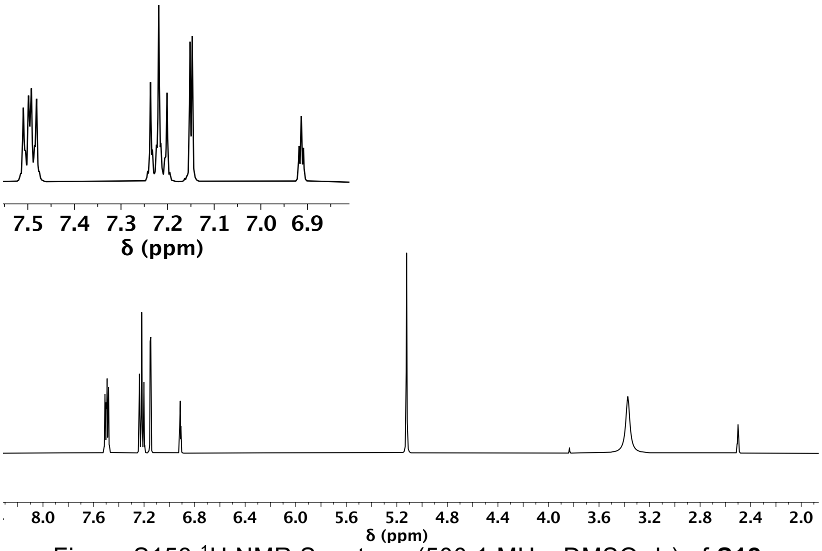

Figure S153 ^{1}H NMR Spectrum (500.1 MHz, DMSO-d_6) of **S12.**

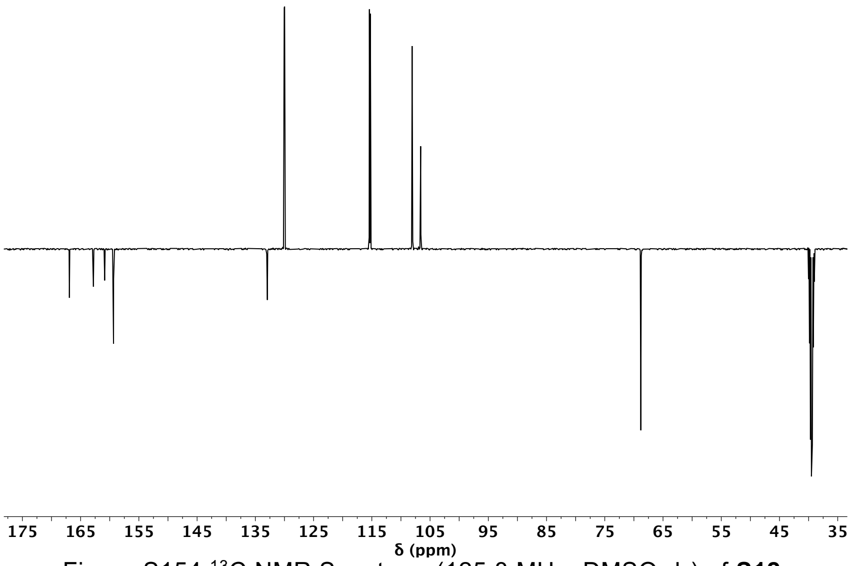

Figure S154 ^{13}C NMR Spectrum (125.8 MHz, DMSO-d_6) of **S12.**

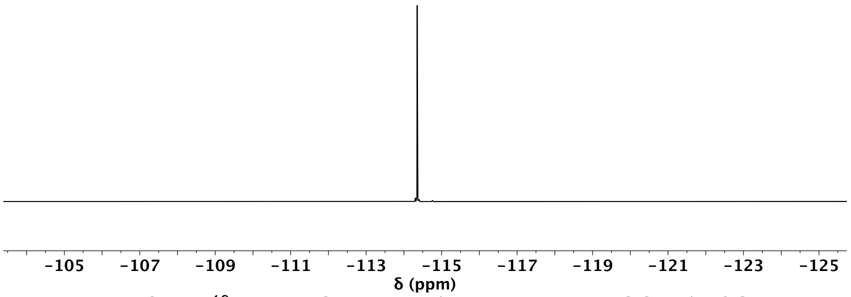

Figure S155 ^{19}F NMR Spectrum (376.5 MHz, DMSO-d_6) of **S12.**

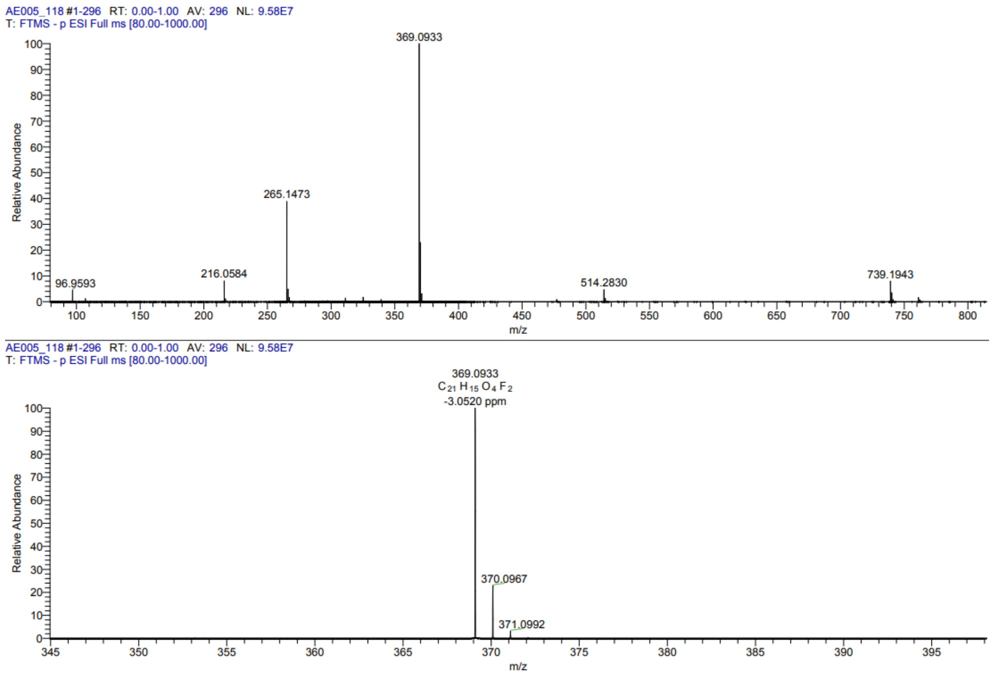

Figure S156 Mass spectrum of **S12**.

23.14 Spectral data for S14

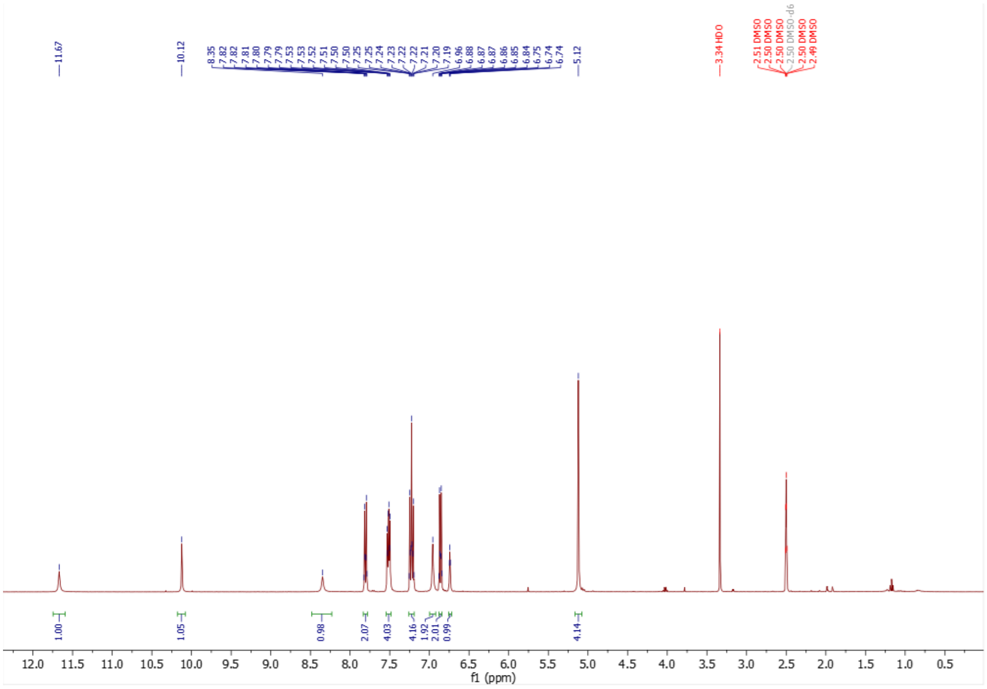

Figure S157 ^{1}H NMR Spectrum (400.1 MHz, DMSO-d_6) of **S14.**

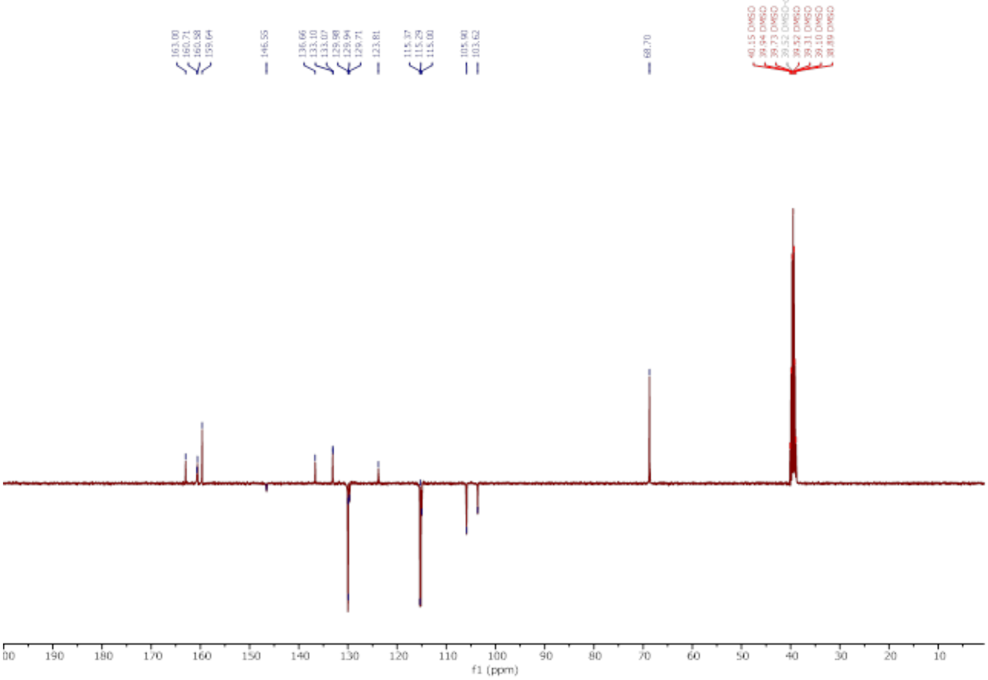

Figure S158 ^{13}C NMR Spectrum (100.6 MHz, DMSO-d_6) of **S14.**

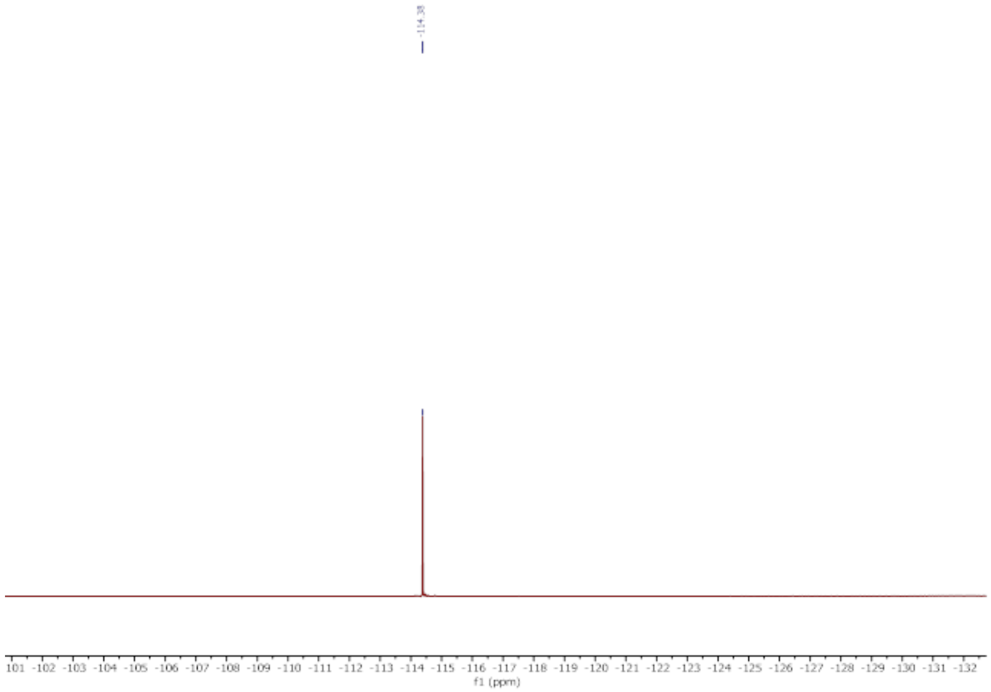

Figure S159 ^{19}F NMR Spectrum (376.5 MHz, DMSO-d_6) of **S14.**

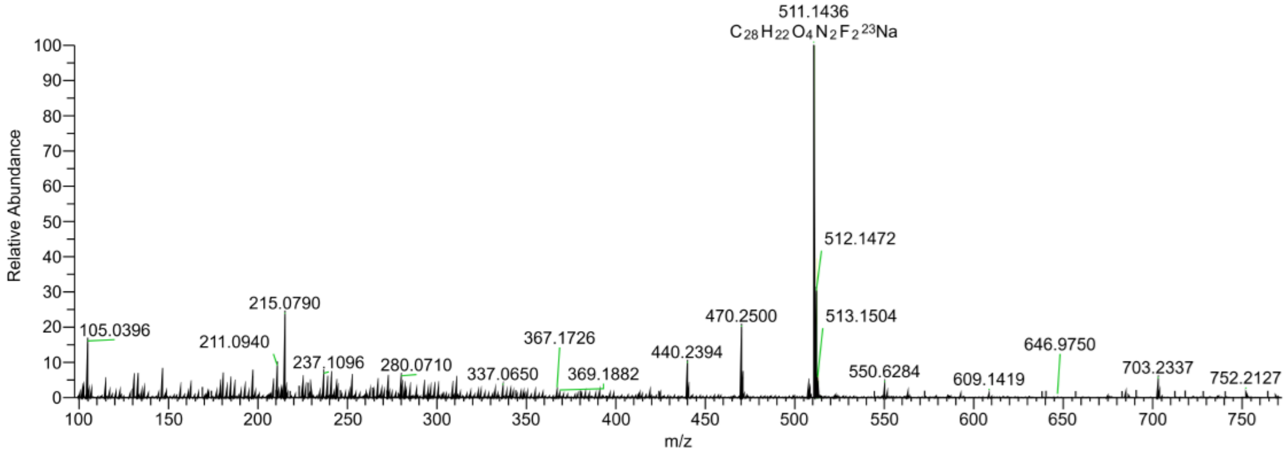

Figure S160 Mass spectrum of **S14.**

23.15 Spectral data for S15

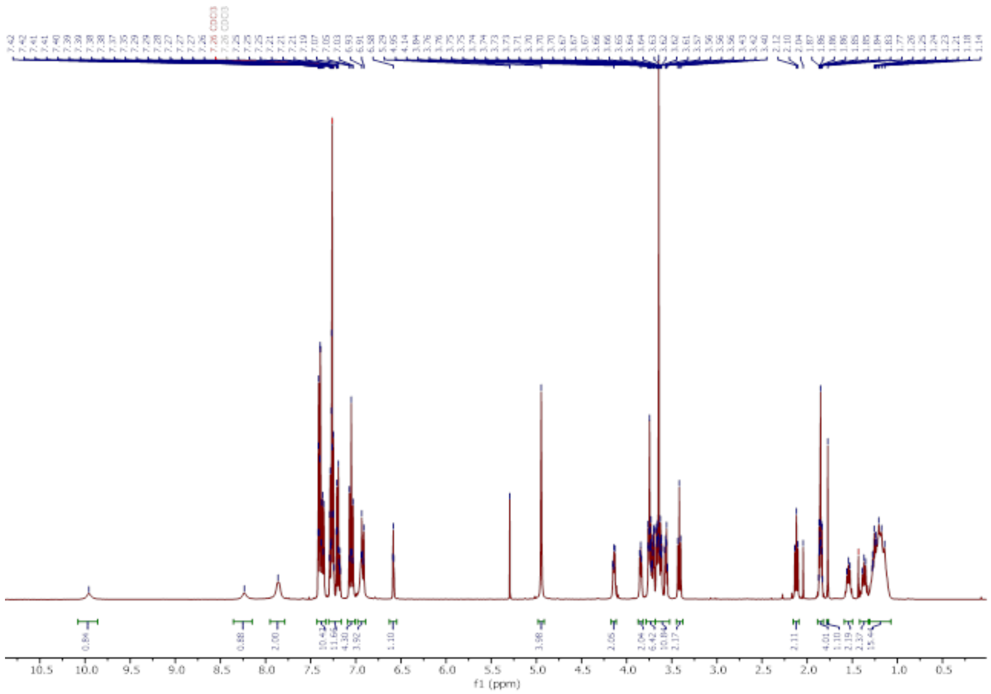

Figure S161 ^{1}H NMR Spectrum (400.1 MHz, $CDCl_6$) of **S15.**

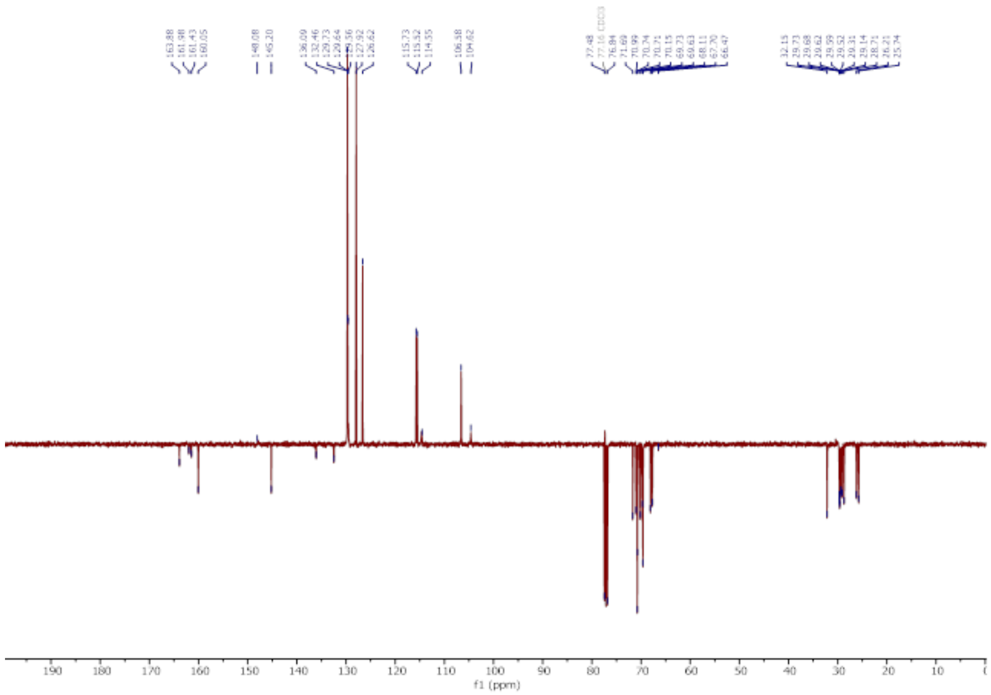

Figure S162 ^{13}C NMR Spectrum (100.6 MHz, $CDCl_3$) of **S15.**

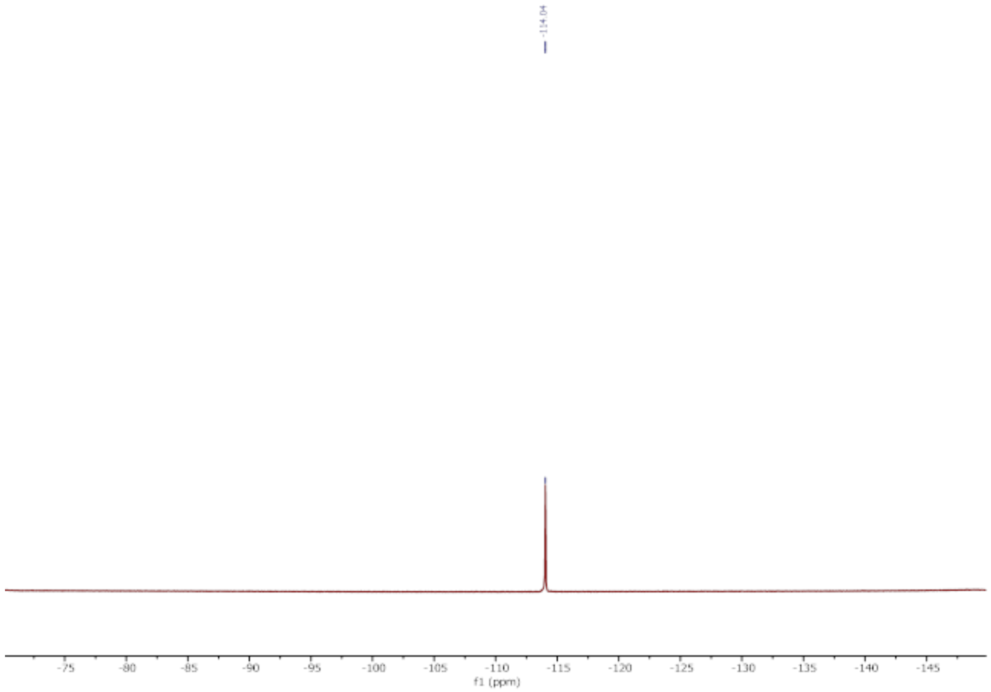

Figure S163 ^{19}F NMR Spectrum (376.5 MHz, DMSO-d_6) of **S15.**

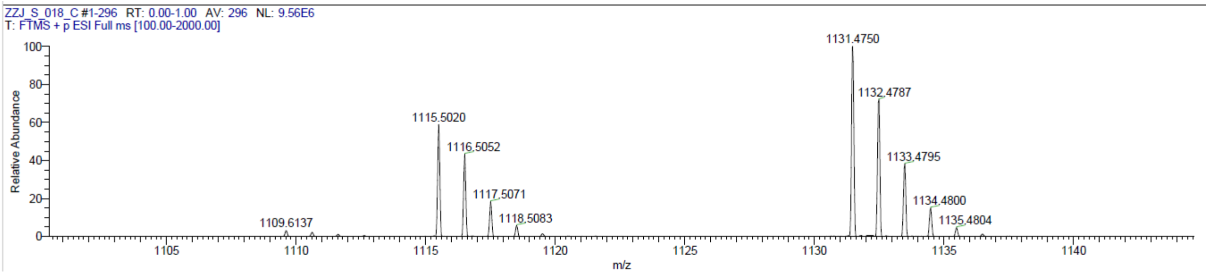

Figure S164 Mass spectrum of **S15.**

23.16 Spectral data for S16₂

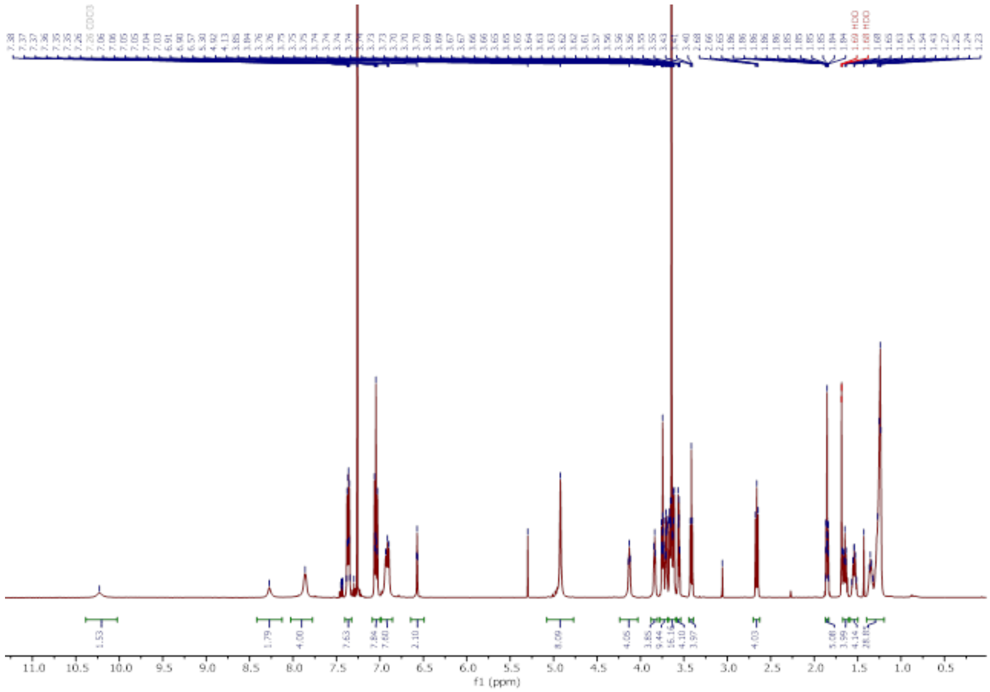

Figure S165 ^{1}H NMR Spectrum (400.1 MHz, $CDCl_3$) of **S16**$_2$.

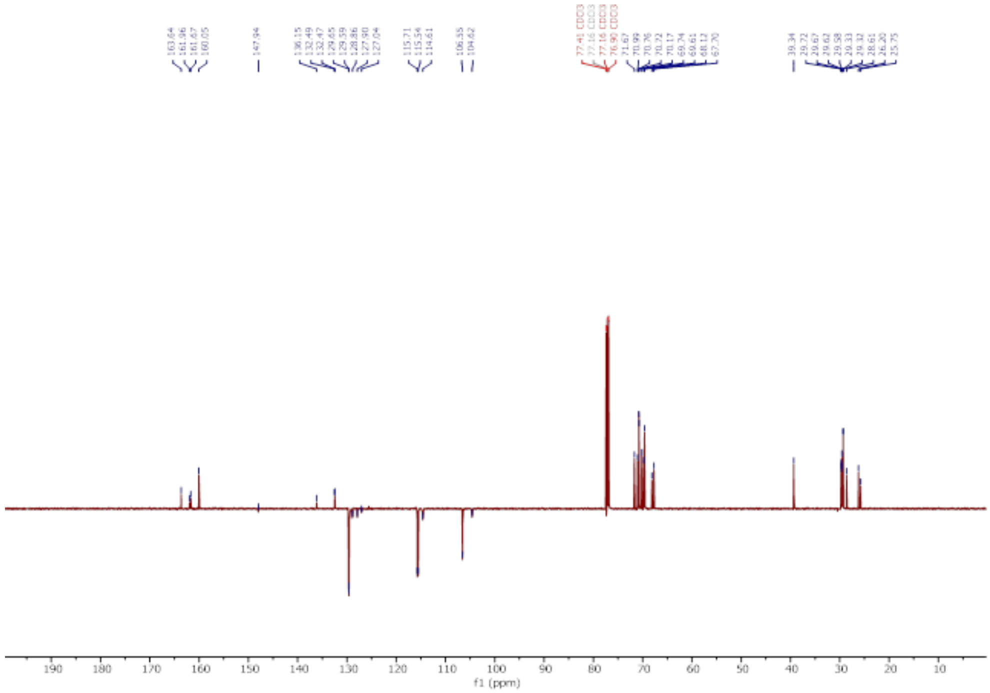

Figure S166 ^{13}C NMR Spectrum (100.6 MHz, $CDCl_3$) of **S16**$_2$.

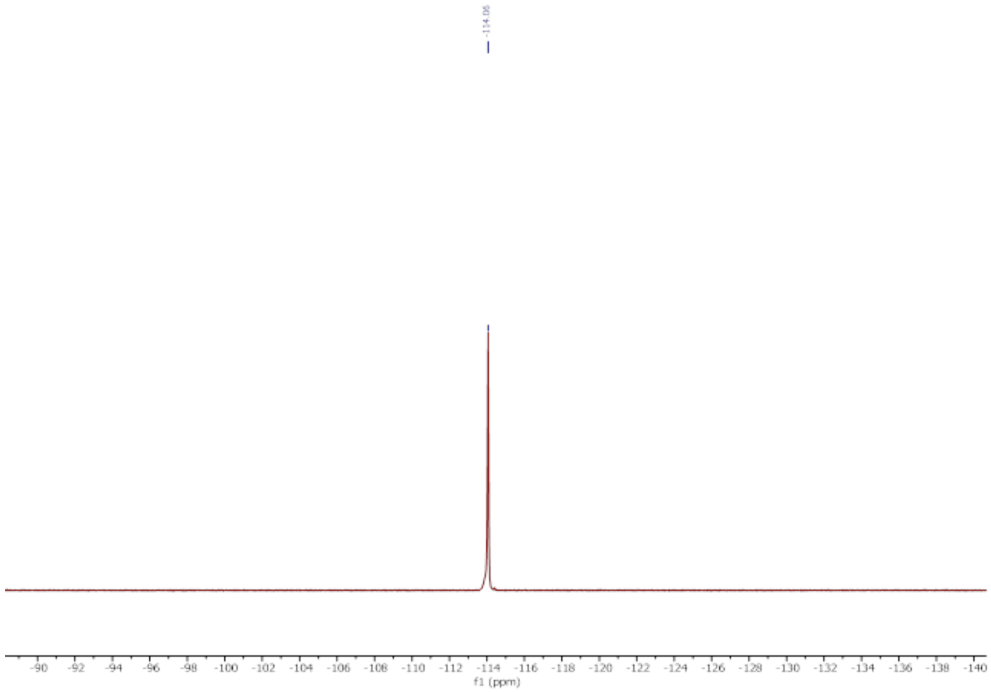

Figure S167 ^{19}F NMR Spectrum (376.5 MHz, DMSO-d$_6$) of **S16**$_2$.

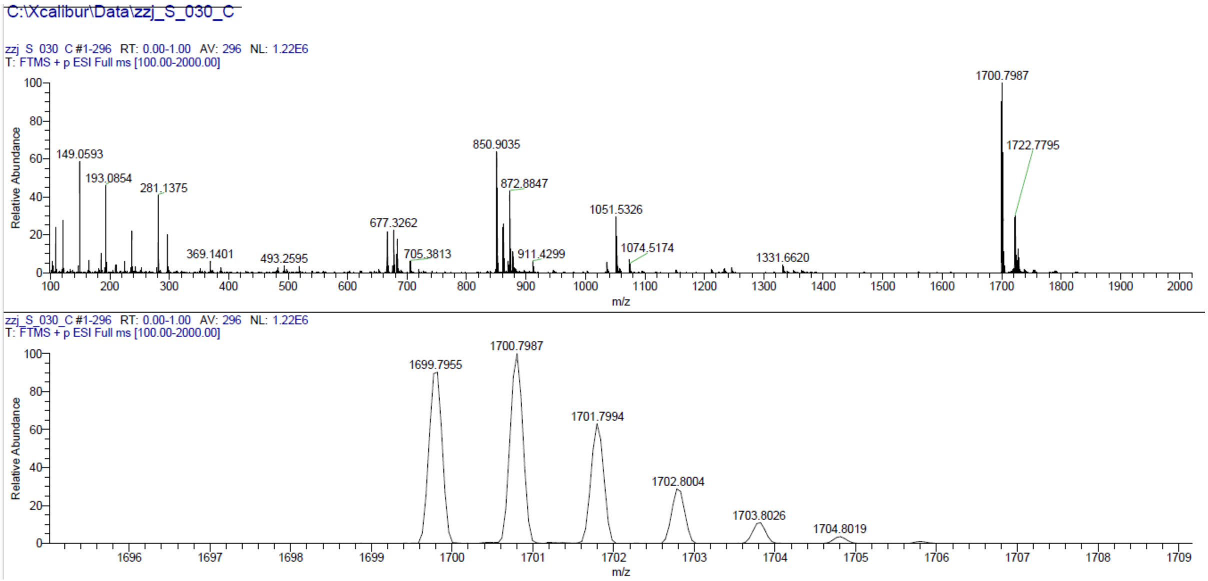

Figure S168 Mass spectrum of **S16**$_2$.

24. References

1. Rouquerol, J., Llewellyn, P. & Rouquerol, F. Is the BET equation applicable to microporous adsorbents? In *Studies in Surface Science and Catalysis* Vol. 160, 49-56 (2006).
2. Luo, S. Y. *et al.* Autotandem catalyst: from acylhydrazones to *N*',*N*'-methylaliphatic acylhydrazides via transfer hydrogenation/*N*-methylation with methanol catalyzed by a Cp* iridium complex bearing a functional ligand. *Org. Lett.* **24**, 9081-9085 (2022).
3. Delgado-Maldonado, T. *et al.* Synthesis and biological evaluation in vitro and in silico of *N*-propionyl-*N*-benzeneacylhydrazone derivatives as cruzain inhibitors of *Trypanosoma cruzi*. *Mol. Diversity* **26**, 39-50 (2022).
4. Arya, N., Mishra, S. K. & Suryaprakash, N. Intramolecular hydrogen bond directed distribution of conformational populations in the derivatives of *N*-benzylidenebenzohydrazide. *New J. Chem.* **43**, 13134-13142 (2019).
5. Geske, L., Baier, J., Boulos, J. C., Efferth, T. & Opatz, T. Xylochemical synthesis and biological evaluation of the orchidaceous natural products isoarundinin I, bleochrin F, blestanol K, and pleionol. *J. Nat. Prod.* **86**, 131-137 (2022).
6. Zhou, Y. H., Gao, G. C., Li, H. & Qu, J. P. A convenient method to reduce hydroxyl-substituted aromatic carboxylic acid with $NaBH_4/Me_2SO_4/B(OMe)_3$. *Tetrahedron Lett.* **49**, 3260-3263 (2008).
7. Bedia, K. K. *et al.* Synthesis and characterization of novel hydrazide-hydrazones and the study of their structure-antituberculosis activity. *Eur. J. Med. Chem.* **41**, 1253-1261 (2006).
8. Diez-Castellnou, M., Suo, R., Matthew, S. A. L., Marro, N. & Kay, E. R. Rapidly adaptive all-covalent nanoparticle surface engineering. *Chem. - Eur. J.* **27**, 9948-9953 (2021).
9. Morris, W. *et al.* Role of modulators in controlling the colloidal stability and polydispersity of the UiO-66 metal-organic framework. *ACS Appl. Mater. Interfaces* **9**, 33413-33418 (2017).
10. Cavka, J. H. *et al.* A new zirconium inorganic building brick forming metal organic frameworks with exceptional stability. *J. Am. Chem. Soc.* **130**, 13850-13851 (2008).
11. Connolly, B. M. *et al.* Tuning porosity in macroscopic monolithic metal-organic frameworks for exceptional natural gas storage. *Nat. Commun.* **10**, 2345 (2019).
12. Alvarez, E. *et al.* The structure of the aluminum fumarate metal-organic framework A520. *Angew. Chem. Int. Ed.* **54**, 3664-3668 (2015).
13. Rastrelli, F., Jha, S. & Mancin, F. Seeing through macromolecules: *T*2-filtered NMR for the purity assay of functionalized nanosystems and the screening of biofluids. *J. Am. Chem. Soc.* **131**, 14222-14224 (2009).
